# Prevalence of vaccine hesitancy in Italy: a cross-sectional study

**DOI:** 10.1016/j.lanepe.2026.101603

**Published:** 2026-01-31

**Authors:** Giuseppina Lo Moro, Fabrizio Bert, Giovanna Elisa Calabrò, Mauro Giovanni Carta, Giulia Cossu, Corrado De Vito, Manuela Martella, Azzurra Massimi, Anna Odone, Paolo Ragusa, Giacomo Pietro Vigezzi, Walter Ricciardi, Roberta Siliquini

**Affiliations:** a Department of Public Health and Pediatric Sciences, University of Turin, Turin, Italy; b Department of Human Sciences, Society and Health, University of Cassino and Southern Lazio, Cassino, Italy; c Department of Medical Sciences and Public Health, University of Cagliari, Cagliari, Italy; d Department of Public Health and Infectious Diseases, Sapienza University of Rome, Rome, Italy; e Department of Public Health, Experimental and Forensic Medicine. University of Pavia, Pavia, Italy; f Medical Direction, Fondazione IRCCS Policlinico San Matteo, Pavia, Italy; g Section of Hygiene, Department of Life Sciences and Public Health, Università Cattolica del Sacro Cuore, Rome, Italy; h AOU City of Health and Sciences, Turin, Italy

**Keywords:** Vaccine hesitancy, Italy, General population, Survey

## Abstract

**Background:**

Vaccine hesitancy (VH) remains a global threat, exacerbated by socio-political uncertainty. We aimed primarily to estimate VH prevalence in Italy, identifying the most susceptible subgroups, and secondarily to assess whether these patterns varied across VH dimensions.

**Methods:**

Cross-sectional survey (web/telephone) among adults in Italy (September 2024–March 2025). The sample (n = 52,094) was nationally representative by age, gender, education, area, municipality size. The primary outcome was VH (score ≥25, adult Vaccine Hesitancy Scale, aVHS). The secondary outcomes were aVHS subscales “Lack of trust” and “Risk perception”. Post-stratification weighting for age, area, and municipality size was applied.

**Findings:**

VH prevalence was 46.09% (95% CI: 45.65–46.53%). Multivariable models showed several associations with VH, e.g., gender, sexual orientation, ethnicity, health literacy, political and religious orientation, personal experiences, and vaccination support from community figures. Among many subgroups significant after multiple-comparison correction, the strongest differences in VH predicted probability (PP) were estimated among individuals using complementary/alternative medicine (PP = 58.5%), right-aligned (PP = 47.0%) or politically unaffiliated participants (PP = 48.4%), individuals with middle school education (PP = 48.3%), people aged 60–74 (PP = 49.0%), and participants uncertain about healthcare workers' pro-vaccination support (PP = 52.8%). While some groups, e.g., individuals with chronic conditions, inadequate health literacy, or religious participants reported higher perceived risk, others, e.g., non-binary respondents, showed higher lack of trust.

**Interpretation:**

This study highlighted the importance of granular data to inform inclusive strategies. Key figures and politics emerged as relevant, deserving further exploration. Future research should evaluate tailored interventions for identified at-risk groups.

**Funding:**

NextGenerationEU funding within the Italian Ministry of University and Research PNRR Extended Partnership initiative on Emerging Infectious Diseases.

**Research in context**

**Evidence before this study**

Vaccine hesitancy (VH) remains a major threat to immunisation programmes. Recent work has proposed conceptualising VH as a state of indecision about vaccination, regardless of the final behaviour. VH is complex, context-dependent, and highly sensitive to time, place, and population, making timely and representative data essential for public health planning. This need has become even more urgent with the global spread of misinformation and socio-political uncertainty. To provide updated data on the Italian context, we searched PubMed up to November 26, 2023, without language restrictions. The search combined free-text and controlled vocabulary terms for “vaccine”, “hesitancy”, and “Italy”. Eligible studies included systematic reviews on VH and observational studies on VH in the general population based on representative samples. Latest reviews largely focused on COVID-19 vaccination and reported fragmented, sometimes conflicting findings. National data stratified by demographic and social characteristics were scarce, and most studies relied on small or non-representative samples or examined vaccination uptake rather than attitudinal VH. Even the largest surveys to date (up to 10,000 participants) mainly assessed vaccination behaviour, underscoring the need for updated, representative evidence to identify high-risk subgroups for VH.

**Added value of this study**

This study used one of the largest nationally representative samples (n = 52,094) ever collected to investigate VH in Italy, ensuring demographic alignment with the adult population. Nearly half of participants reported VH according to the adult Vaccine Hesitancy Scale (aVHS). Our findings highlighted differences in VH across demographic and social profiles and showed that personal experiences, political and religious orientation, and perceived support from community figures may substantially shape hesitancy. We found differences in VH by gender, sexual identity, and ethnicity, underscoring the need to include these variables in studies of hesitancy, which have generally been understudied in the Italian population. Among the findings with limited prior evidence, we observed that lacking or being unaware of pro-vaccination support from health-care workers, teachers, or religious leaders was associated with higher VH, whereas lack of awareness of local politicians’ positions was associated with lower VH. Political orientation also mattered: although right-wing affiliation was linked to higher VH, hesitancy was also pronounced among centrists and the politically unaffiliated, suggesting the need for depoliticised health communication. While based in Italy, the study highlights issues common to many countries, including institutional mistrust, political polarisation, and subgroup-specific hesitancy, with relevance for international public health planning.

**Implications of all the available evidence**

Interventions should be tailored to profiles underserved by standard approaches, and may need to extend beyond traditional health-care settings by engaging trusted community professionals. Strengthening service accessibility, quality, and responsiveness, alongside efforts to rebuild institutional trust, remains essential. More granular data, including information on gender, sexual identity, and ethnicity, are needed to inform inclusive strategies. The role of community leaders should be further investigated to understand how engagement through local networks can build trust. Additionally, the influence of political and ideological signals on VH, and the potential of coordinated communication to reduce polarisation, should be carefully evaluated. Future research should monitor changes in VH over time and assess the impact of targeted interventions for diverse and marginalised groups.

## Introduction

Vaccine hesitancy (VH), defined as the delay in acceptance or refusal of vaccines despite the availability of services,^1^ remains a major threat to immunisation programmes. Interestingly, recent research highlighted the need to distinguish VH from vaccination behaviour, conceptualising VH as a state of indecision about vaccination, regardless of the final behaviour.^2^

Although the present study is grounded in conceptual frameworks long proposed to guide research on VH, such as the 3C model and the SAGE matrix of determinants,^1^ VH remains, by definition, a complex and context-dependent phenomenon. Its determinants vary across time, place, and population, requiring timely data to inform public health planning.^1^ The importance of up-to-date and evidence-based data is underscored by recent debates suggesting that the VH challenge may be amplified by the global spread of misinformation and political interference in scientific decision-making.^3^^,^^4^ The USA, traditionally a cornerstone of health leadership, has become a driver of mistrust through the international diffusion of vaccine falsehoods, undermining confidence worldwide.^3^ Similarly, in Italy, a controversy surrounding the National Immunization Technical Advisory Group (NITAG) raised concerns that politicised appointments could weaken immunisation policies and public trust.^4^

Regarding Italy, the most recent evidence synthesis on VH in the general population focused on COVID-19, highlighting various predisposing factors but also reporting fragmented findings.^5^ Some authors have highlighted substantial geographic heterogeneity in vaccine attitudes across Italy, with higher hesitancy in northern regions and mixed evidence regarding urban versus rural settings.^5^, ^6^, ^7^ National data stratified by key characteristics remain limited. Existing research often relied on small or non-representative samples or focused primarily on vaccination behaviour rather than attitudinal aspects. Even a recent large-scale survey with 10,000 participants has concentrated mostly on behavioural uptake,^7^ underscoring the need for updated, representative data to identify high-risk subgroups for VH, particularly in a situation of political and societal uncertainty.

In this context, the INF-ACT project (https://www.inf-act.it/) was established as a national collaboration among Italian universities and institutions, addressing the unmet needs of emerging infectious diseases. A Work Package aimed to investigate VH in the Italian adult population. Thus, the present study had the main goal to provide a comprehensive and updated picture of VH in a large representative sample of the Italian adult general population, thereby supporting the design of focused and targeted interventions. The primary objective was to estimate the prevalence of VH and to identify the most susceptible subgroups of the population. A secondary objective was to examine whether these patterns varied across different dimensions of VH. The central research question guiding this study was: which subgroups within the Italian adult population are most likely to exhibit VH?

## Methods

### Study design and participants

This nationwide cross-sectional study was based on a questionnaire developed by the research team.

The survey was conducted in Italy between September 2024 and March 2025, with data collection managed by a professional polling agency using a non-probability, quota-based sampling strategy. Computer-Assisted Web Interviewing (CAWI) was the primary method, supplemented by Computer-Assisted Telephone Interviewing (CATI) to ensure quota completion. Quotas were established to reflect the adult Italian population by age group, gender, geographic area, education, and municipality size. Eligibility criteria included being 18 years or older, residing in Italy, and having a sufficient understanding of the Italian language.

Survey dissemination, participant invitation, informed consent procedures, and data privacy compliance were managed by the polling agency, under oversight from the research team. All items were mandatory to complete the survey. To estimate the prevalence of VH with a 1% margin of error and 95% confidence level, a minimum sample of approximately 10,000 adults was required considering the Italian adult population (http://www.raosoft.com/samplesize.html). We targeted a larger sample (∼50,000) to enable robust subgroup and multivariable analyses, ensure adequate representation of minority groups, and improve the precision of estimates (Supplementary Methods M1).

Considering the recruitment by the polling agency, participants in the CAWI component were drawn a large, pre-existing national web panel managed by the agency. This panel is continuously updated through agreements with partner organisations and targeted online advertisements on social media platforms. All members of the panel have voluntarily consented to participate in surveys and provided sociodemographic information used to ensure quota alignment. Invitations containing a unique link to the online questionnaire were sent via e-mail. Participation was voluntary, and respondents could complete the questionnaire on a computer, tablet, or smartphone. Informed consent was obtained digitally before accessing the survey. Among 209,448 CAWI contacts (i.e., the panel), 36,644 respondents completed the questionnaire (completion rate 17.5%), 6454 interrupted it, 12,125 declined participation after opening the link (without starting the questionnaire), and 154,225 did not open the invitation.

To improve coverage of subgroups typically underrepresented in online panels (e.g., older adults or without internet access) and guarantee the quota completion, a CATI component was used. Telephone numbers (both mobile and landline) were drawn randomly from databases provided by an external supplier compliant with the “Registro delle Opposizioni” (the national opt-out registry that regulates the use of personal contact data for research and marketing purposes), ensuring inclusion of individuals who had given prior consent to be contacted for surveys. CATI respondents were contacted directly by trained interviewers (no automated calls or digital operators). Each number was generally attempted once; calls ended with either refusal, immediate interview, or acceptance followed by non-completion. A total of 56,019 potential respondents were contacted by phone: 15,450 completed the interview (completion rate 27.6%), 531 interrupted it, and 40,038 refused participation. Informed consent was obtained verbally at the beginning of the call, with the acceptance portion of the conversation recorded in accordance with privacy regulations.

Differences between CAWI and CATI respondents were expected and consistent with the recruitment method, as CATI interviews were specifically used to reach individuals with limited internet access and to refine quota coverage. A comparison of the two subsamples by key sociodemographic characteristics is reported in Supplementary Methods M1.

To mitigate selection bias that may arise from non-probability sampling, quotas were aligned with national demographic distributions. Social desirability bias was minimised by using self-administered CAWI as the primary mode, and avoiding the collection of identifying data. Recall bias was considered minimal given the nature of the questions, focussing on current attitudes and recent experiences.

### Procedures

#### Outcomes

We measured VH using the adult Vaccine Hesitancy Scale (aVHS),^8^ which consists of 10 items rated on a 5-point Likert scale with a total score ranging from 10 to 50, where higher scores indicate greater hesitancy.^8^ In our sample, aVHS internal consistency was high (Cronbach's α = 0.894). Participants scoring 25 or above were classified as vaccine-hesitant, in line with the original validation study^8^ and subsequent research using the aVHS.^9^, ^10^, ^11^ This binary classification was used as the primary outcome.

As secondary outcomes, we used the aVHS subscales: “Lack of trust” dimension (items 1–4, 6–8; score range 7–35, Cronbach's α = 0.955) and “Risk perception” dimension (items 5, 9, 10; score range 3–15, Cronbach's α = 0.764). Supplementary Methods M2 show details.

#### Independent variables

To characterise vaccine-hesitant adults in Italy, we included a comprehensive set of variables, selected on the basis of previous literature. The variables were organised in the following conceptual blocks: sociodemographic and socioeconomic characteristics (e.g., age, gender, occupation, socioeconomic status, nationality, ethnic group, sexual orientation); health-related characteristics and personal experience (e.g., chronic conditions, health literacy (HL), living arrangements, experiences with vaccine-preventable diseases (VPD) or adverse events following immunisation (AEFI), barriers when trying to access vaccination services); information sources; external influences (e.g., religious leaders, political figures, teachers, and healthcare workers (HCWs)); beliefs and attitudes (e.g., use of complementary and alternative medicine (CAM), political orientation, religion, perceptions of the Italian National Health Service (NHS)); vaccine conspiracy beliefs (Vaccine Conspiracy Beliefs Scale (VCBS)^12^ where a higher average score reflects a stronger endorsement of conspiracy beliefs), which we hypothesised may contribute to VH and account for part of its variance. The variables in Tables 1 and 2 reflect the original response options from the questionnaire, unless otherwise specified in the Supplementary Methods M3, where definitions, rationale, sources, and recategorizations are provided. “Prefer not to answer” options were included for gender, sexual orientation, political orientation, and religion, following consultation with the Ethics Committee, as these variables were considered sensitive.

**Table 1:** Sociodemographic, socioeconomic, health-related characteristics and personal experience: analyses with vaccine hesitancy as outcome.

	Overall sample n = 52,094	Prevalence of vaccine hesitancy^a^	Univariable regression		Multivariable regression^b^	
	(%)	% (95% CI)	OR (95% CI)	p	adjOR (95% CI)	p
**Block 1–Sociodemographic and socioeconomic characteristics**						
**Age group**		<0.0001				
18–29	14.39%	45.59% (44.44–46.75)	Ref.			
30–44	20.26%	53.6% (52.64–54.57)	1.38 (1.3–1.46)	<0.0001	1.46 (1.34–1.58)	<0.0001
45–59	27.46%	49.6% (48.8–50.41)	1.17 (1.11–1.24)	<0.0001	1.57 (1.44–1.72)	<0.0001
60–74	22.8%	42.07% (41.15–43)	0.87 (0.82–0.92)	<0.0001	1.75 (1.57–1.96)	<0.0001
75+	15.1%	36.17% (34.91–37.44)	0.68 (0.63–0.73)	<0.0001	1.47 (1.29–1.68)	<0.0001
**Gender**		<0.0001				
Male	48.31%	44.96% (44.32–45.6)	Ref.			
Female	50.68%	46.78% (46.16–47.4)	1.08 (1.04–1.12)	<0.0001	1.02 (0.97–1.07)	0.44
Non-binary/Other	0.94%	66.76% (62.23–70.99)	2.46 (2.01–3)	<0.0001	2.03 (1.6–2.58)	<0.0001
Prefer not to answer	0.07%	51.7% (36.25–66.82)	1.31 (0.7–2.47)	0.403	1.02 (0.46–2.25)	0.97
**Marital status**		<0.0001				
Single	22.99%	49.74% (48.82–50.65)	Ref.			
Married	53.17%	43.31% (42.7–43.91)	0.77 (0.74–0.81)	<0.0001	0.91 (0.84–0.98)	0.0103
Separated/Divorced	7.04%	53.89% (52.2–55.56)	1.18 (1.09–1.28)	<0.0001	1.13 (1.02–1.26)	0.025
Cohabiting	12.15%	51.71% (50.42–52.99)	1.08 (1.02–1.15)	0.014	1.05 (0.96–1.14)	0.28
Widowed	4.65%	33.43% (31.42–35.49)	0.51 (0.46–0.56)	<0.0001	0.79 (0.69–0.9)	0.00064
**Children**		<0.0001				
No children	35.83%	49.88% (49.15–50.61)	Ref.			
Only children ≤11 years	11.86%	53.52% (52.26–54.78)	1.16 (1.09–1.23)	<0.0001	0.93 (0.86–1.01)	0.093
Only children 12–18 years	6.53%	53.59% (51.92–55.26)	1.16 (1.08–1.25)	<0.0001	0.88 (0.8–0.98)	0.015
Only children >18 years	40.77%	39.3% (38.6–40.01)	0.65 (0.62–0.68)	<0.0001	0.79 (0.73–0.85)	<0.0001
Children of various ages	5.01%	46.88% (44.96–48.81)	0.89 (0.82–0.96)	0.0045	0.76 (0.68–0.84)	<0.0001
**Sexual orientation**		<0.0001				
Heterosexual	88.95%	45.68% (45.21–46.15)	Ref.			
Homosexual	1.63%	46.25% (42.78–49.76)	1.02 (0.89–1.18)	0.75	0.82 (0.68–0.98)	0.032
Bisexual	2.14%	47.24% (44.21–50.29)	1.06 (0.94–1.2)	0.32	0.79 (0.68–0.92)	0.00296
Pansexual	0.7%	65.79% (60.67–70.56)	2.29 (1.83–2.85)	<0.0001	0.92 (0.71–1.18)	0.5002
Ace spectrum	0.98%	69.09% (64.75–73.12)	2.66 (2.18–3.24)	<0.0001	0.86 (0.66–1.11)	0.24
Prefer not to answer	5.59%	45.67% (43.85–47.49)	1 (0.93–1.08)	0.99	0.66 (0.59–0.73)	<0.0001
**Municipality size (inhabitants)**		<0.0001				
≤10,000	30.57%	47.04% (46.26–47.83)	Ref.			
10,001–25,000	20.76%	44.34% (43.4–45.28)	0.9 (0.85–0.94)	<0.0001	0.97 (0.91–1.03)	0.35
25,001–50,000	14.78%	43.4% (42.29–44.51)	0.86 (0.82–0.91)	<0.0001	0.98 (0.91–1.05)	0.55
50,001–100,000	10.64%	47.2% (45.84–48.56)	1.01 (0.94–1.07)	0.85	1.07 (0.96–1.19)	0.23
100,001–250,000	8.47%	50.64% (49–52.27)	1.15 (1.07–1.24)	0.0001	1.11 (0.98–1.26)	0.104
>250,000	14.77%	45.87% (44.65–47.1)	0.95 (0.9–1.01)	0.12	0.997 (0.89–1.12)	0.96
**Geographic macro-area**		0.0025				
North-West	27.08%	46.28% (45.44–47.13)	Ref.			
North-East	19.69%	45.65% (44.68–46.62)	0.97 (0.93–1.03)	0.33	1.04 (0.98–1.11)	0.23
Centre	19.96%	44.78% (43.77–45.8)	0.94 (0.89–0.99)	0.026	1.02 (0.96–1.09)	0.55
South	22.53%	47.51% (46.59–48.43)	1.05 (1–1.1)	0.054	0.94 (0.88–1)	0.052
Islands	10.74%	45.87% (44.46–47.28)	0.98 (0.92–1.05)	0.62	0.88 (0.8–0.95)	0.0026
**Degree of urbanisation**		0.0044				
Pole	35.78%	46.53% (45.76–47.3)	Ref.			
Intermunicipal pole	2.49%	44.41% (41.67–47.17)	0.92 (0.82–1.03)	0.15	0.99 (0.86–1.15)	0.92
Belt	38.09%	46.51% (45.8–47.22)	1 (0.96–1.04)	0.98	1.08 (0.99–1.18)	0.098
Intermediate	14.13%	45.76% (44.61–46.91)	0.97 (0.92–1.03)	0.28	1.09 (0.98–1.21)	0.106
Peripheral	8.14%	43.33% (41.83–44.83)	0.88 (0.82–0.94)	0.00021	1 (0.88–1.12)	0.96
Ultra-peripheral	1.38%	45.91% (42.31–49.55)	0.98 (0.84–1.13)	0.74	0.96 (0.79–1.18)	0.72
**Education level**		<0.0001				
Upper secondary	52.67%	48.49% (47.87–49.1)	Ref.			
Primary/None	2.58%	33.36% (30.83–35.99)	0.53 (0.47–0.6)	<0.0001	0.53 (0.46–0.61)	<0.0001
Lower secondary	14.75%	48.21% (47.06–49.36)	0.99 (0.94–1.04)	0.68	1.07 (1–1.14)	0.0597
University	23.91%	42.24% (41.34–43.15)	0.78 (0.74–0.81)	<0.0001	0.82 (0.78–0.87)	<0.0001
Postgraduate	6.1%	40.77% (39.01–42.55)	0.73 (0.68–0.79)	<0.0001	0.7 (0.63–0.77)	<0.0001
**Occupational status**		<0.0001				
Non-healthcare worker	45.96%	49.41% (48.75–50.07)	Ref.			
Healthcare worker	4.89%	42.56% (40.6–44.55)	0.76 (0.7–0.83)	<0.0001	0.7 (0.63–0.77)	<0.0001
Homemaker	8.03%	52.69% (51.11–54.26)	1.14 (1.06–1.22)	0.00025	1.09 (0.99–1.19)	0.067
Retired	27%	36.72% (35.87–37.57)	0.59 (0.57–0.62)	<0.0001	0.83 (0.76–0.91)	<0.0001
Student (non-health field)	4.07%	38.83% (36.71–40.99)	0.65 (0.59–0.71)	<0.0001	0.65 (0.57–0.74)	<0.0001
Student (health field)	1.56%	35.86% (32.53–39.34)	0.57 (0.49–0.67)	<0.0001	0.54 (0.44–0.65)	<0.0001
Job seeker	3.70%	55.73% (53.39–58.04)	1.29 (1.16–1.42)	<0.0001	1.02 (0.91–1.16)	0.69
Unemployed	4.66%	58.1% (56.04–60.14)	1.42 (1.3–1.55)	<0.0001	1.02 (0.91–1.14)	0.73
Other	0.14%	45.88% (34.22–58.02)	0.87 (0.53–1.44)	0.59	0.77 (0.43–1.37)	0.37
**Continent of citizenship**		0.49				
Italy	98.08%	46.05% (45.6–46.49)	Ref.			
Europe (non-Italy)	1.16%	49.01% (44.88–53.16)	1.13 (0.95–1.33)	0.16	0.97 (0.79–1.2)	0.801
Africa	0.28%	50.52% (42.15–58.87)	1.2 (0.85–1.68)	0.299	1.19 (0.75–1.91)	0.46
America	0.29%	45.53% (37.56–53.73)	0.98 (0.7–1.36)	0.901	0.87 (0.54–1.39)	0.55
Asia	0.19%	44.41% (34.87–54.38)	0.94 (0.63–1.4)	0.75	1.02 (0.59–1.78)	0.94
Oceania	0.002%	100%	empty			
**Self-identified ethnicity**		<0.0001				
European	96.67%	45.6% (45.15–46.05)	Ref.			
Multi-ethnic	0.77%	65.65% (60.66–70.32)	2.28 (1.84–2.83)	<0.0001	1.24 (0.95–1.61)	0.107
North American/Australian	0.31%	70.47% (62.43–77.4)	2.85 (1.98–4.09)	<0.0001	1.44 (0.92–2.25)	0.11
Arab-Middle Eastern	0.43%	64.85% (58.07–71.08)	2.2 (1.65–2.93)	<0.0001	0.95 (0.64–1.4)	0.78
North African	0.46%	57.48% (50.9–63.82)	1.61 (1.24–2.11)	0.00044	1.25 (0.86–1.82)	0.24
Latino-American	0.67%	55.22% (49.82–60.5)	1.47 (1.18–1.83)	0.00051	0.88 (0.64–1.22)	0.46
African American	0.08%	78.63% (63.39–88.66)	4.39 (2.07–9.33)	0.00012	1.73 (0.74–4.04)	0.24
Black African	0.19%	47.95% (37.87–58.2)	1.1 (0.73–1.66)	0.65	0.84 (0.49–1.43)	0.52
Asian	0.31%	51.66% (43.78–59.45)	1.27 (0.93–1.75)	13	0.99 (0.63–1.56)	0.96
Pacific Islands	0.00%	51.75% (37.69–65.53)	1.28 (0.72–2.27)	0.399	0.58 (0.26–1.32)	0.197
**Material deprivation**		<0.0001				
No deprivation	95.72%	45.8% (45.35–46.26)	Ref.			
Severe deprivation	4.28%	52.54% (50.4–54.68)	1.31 (1.2–1.43)	<0.0001	1.01 (0.9–1.13)	0.88
**Block 2–Health-related characteristics and personal experience**						
**Chronic conditions**		<0.0001				
No chronic disease	54.03%	46.95% (46.35–47.55)	1 (0.96–1.05)	0.88	0.93 (0.89–0.98)	0.011
One chronic disease	28.43%	47.03% (46.19–47.86)	0.82 (0.78–0.86)	<0.0001	0.85 (0.8–0.91)	<0.0001
More than one chronic disease	17.53%	41.94% (40.88–42.99)				
**Living with a person with disability**		<0.0001				
No	82.56%	44.92% (44.43–45.41)	Ref.			
Yes	17.44%	51.64% (50.57–52.7)	1.31 (1.25–1.37)	<0.0001	1.01 (0.95–1.07)	0.71
**Inadequate health literacy**		<0.0001				
No	59.94%	44.21% (43.64–44.78)	Ref.			
Yes	40.06%	48.91% (48.21–49.6)	1.21 (1.16–1.25)	<0.0001	1.14 (1.09–1.2)	<0.0001
**Knowing someone who had AEFI**		<0.0001				
No	68.12%	36.06% (35.54–36.58)	Ref.			
Yes	31.88%	67.53% (66.79–68.26)	3.69 (3.54–3.84)	<0.0001	3.44 (3.27–3.62)	<0.0001
**Knowing someone who had VPD**		<0.0001				
No	77.47%	46.71% (46.21–47.22)	Ref.			
Yes	22.53%	43.96% (43.03–44.89)	0.89 (0.86–0.93)	<0.0001	0.52 (0.49–0.55)	<0.0001
**Reported barriers to vaccination**		<0.0001				
No	53.58%	37.97% (37.38–38.56)	Ref.			
Yes	46.42%	55.47% (54.82–56.11)	2.03 (1.96–2.11)	<0.0001	1.35 (1.29–1.41)	<0.0001

(post-stratification weights for age group, geographic area, and municipality size applied).

Abbreviations: adjOR, adjusted Odds Ratio; AEFI, Adverse Event Following Immunisation; CI, Confidence Interval; OR, Odds Ratio; VPD, Vaccine Preventable Disease.

a p-value in this column were obtained via Chi-squared tests.

b The multivariable regression model reported in Table 1 was adjusted for all the variables presented in Tables 1 and 2 (Block 1–6). Post-stratification weights for age group, geographic area, and municipality size are applied (unweighted model in Tables S1 and S2, comparison between weighted and unweighted models in Table S3). Note: Regression estimates should not be interpreted as causal effects.

**Table 2:** Information sources and trust, external influences, beliefs, attitudes and survey mode: analyses with vaccine hesitancy as outcome.

	Overall sample n = 52,094	Prevalence of vaccine hesitancy^a^	Univariable regression		Multivariable regression^b^	
	(%)	% (95% CI)	OR (95% CI)	p	adjOR (95% CI)	p
**Block 3–Information sources and trust**						
**Information source cluster**		<0.0001				
Diversified sources	62.8%	53.45% (52.89–54.01)	Ref.			
Professional-only sources	37.2%	33.67% (32.99–34.37)	0.44 (0.43–0.46)	<0.0001	0.73 (0.7–0.77)	<0.0001
**Trust in sources**^c^ (from 1 = not at all to 4 = very much)	2.98 (0.003)	–	0.3 (0.29–0.31)	<0.0001	0.45 (0.43–0.47)	<0.0001
**Block 4—External influences (perceived vaccination endorsement in the respondent's community by:)**						
**By religious leaders**		<0.0001				
Yes	27.61%	40.68% (39.84–41.53)	Ref.			
No	19.38%	62.43% (61.45–63.4)	2.42 (2.29–2.56)	<0.0001	1.31 (1.21–1.42)	<0.0001
Don't know	53.00%	42.94% (42.33–43.54)	1.1 (1.05–1.14)	<0.0001	1.01 (0.94–1.07)	0.86
**By political leaders**		<0.0001				
Yes	40.04%	42.4% (41.7–43.1)	Ref.			
No	17.62%	63.49% (62.46–64.51)	2.36 (2.24–2.49)	<0.0001	1.06 (0.98–1.14)	0.18
Don't know	42.34%	42.34% (41.67–43.02)	1 (0.96–1.04)	0.913	0.69 (0.65–0.74)	<0.0001
**By teachers**		<0.0001				
Yes	44.95%	38.13% (37.48–38.77)	Ref.			
No	14.99%	67.68% (66.58–68.75)	3.4 (3.21–3.6)	<0.0001	1.4 (1.28–1.52)	<0.0001
Don't know	40.06%	46.95% (46.25–47.65)	1.44 (1.38–1.49)	<0.0001	1.33 (1.24–1.42)	<0.0001
**By health professionals**		<0.0001				
Yes	61.73%	37.72% (37.17–38.27)	Ref.			
No	12.32%	69.96% (68.78–71.11)	3.85 (3.62–4.09)	<0.0001	1.66 (1.52–1.81)	<0.0001
Don't know	25.94%	54.68% (53.82–55.55)	1.99 (1.91–2.08)	<0.0001	1.92 (1.79–2.05)	<0.0001
**Block 5—Beliefs and attitudes**						
**Use of non-conventional medicine**		<0.0001				
No	67.73%	40.63% (40.1–41.16)	Ref.			
Yes, integrated with conventional medicine	22.92%	52.03% (51.1–52.96)	1.58 (1.52–1.65)	<0.0001	1.21 (1.15–1.28)	<0.0001
Yes, as alternative to conventional medicine	9.35%	71.09% (69.76–72.4)	3.59 (3.36–3.85)	<0.0001	2.24 (2.06–2.45)	<0.0001
**Political orientation**		<0.0001				
Right (7–9)	21.09%	47.72% (46.74–48.69)	Ref.			
Centre (4–6)	31.04	50.81% (50.01–51.61)	1.13 (1.08–1.19)	<0.0001	0.96 (0.9–1.02)	0.17
Extreme left (0)	4.25%	44.35% (42.17–46.55)	0.87 (0.79–0.96)	0.0061	0.65 (0.57–0.73)	<0.0001
Left (1–3)	13.93%	34% (32.86–35.15)	0.56 (0.53–0.6)	<0.0001	0.56 (0.52–0.61)	<0.0001
Extreme right (10)	3.97%	52.52% (50.27–54.75)	1.21 (1.1–1.34)	0.00013	1.11 (0.98–1.26)	0.098
Non-aligned with traditional parties	17.4%	50.22% (49.17–51.27)	1.11 (1.04–1.17)	0.00063	1.03 (0.96–1.11)	0.41
Prefer not to answer	8.32%	33.8% (32.36–35.27)	0.56 (0.52–0.6)	<0.0001	0.9 (0.81–0.99)	0.026
**Religion**		<0.0001				
Catholic	71.78%	45.03% (44.5–45.55)	Ref.			
Orthodox	2.79%	42.47% (39.95–45.02)	0.9 (0.81–1)	0.055	0.93 (0.81–1.08)	0.34
Protestant	0.87%	62.82% (58.05–67.35)	2.06 (1.69–2.52)	<0.0001	1.35 (1.06–1.73)	0.015
Jewish	0.34%	69.86% (61.67–76.96)	2.83 (1.96–4.08)	<0.0001	1.87 (1.2–2.92)	0.0055
Muslim	1.33%	58.07% (54.21–61.83)	1.69 (1.44–1.98)	<0.0001	1.24 (0.99–1.56)	0.066
Jehovah's Witness	0.94%	66.3% (61.9–70.44)	2.4 (1.98–2.91)	<0.0001	1.51 (1.18–1.92)	0.00088
Atheist	10.68%	41.53% (40.19–42.88)	0.87 (0.82–0.92)	<0.0001	0.96 (0.88–1.06)	0.44
Agnostic	3.37%	39.21% (36.88–41.6)	0.79 (0.71–0.87)	<0.0001	0.96 (0.84–1.1)	0.54
Buddhist	0.56%	62.54% (56.66–68.06)	2.04 (1.6–2.6)	<0.0001	1.27 (0.93–1.72)	0.13
Hindu	0.14%	76.57% (64.75–85.32)	3.99 (2.24–7.1)	<0.0001	2.24 (1.2–4.16)	0.011
Other	1.82%	63.35% (60.15–66.43)	2.11 (1.84–2.42)	<0.0001	1.43 (1.21–1.68)	<0.0001
Prefer not to answer	5.37%	56.52% (54.57–58.45)	1.59 (1.46–1.72)	<0.0001	1.28 (1.15–1.43)	<0.0001
**Importance of religion**		<0.0001				
Not at all (0)	14.37%	42.63% (41.47 to 43.79)	Ref.			
Slightly (1–3)	14.11%	46.23% (45.04–47.43)	1.16 (1.08–1.24)	<0.0001	1.17 (1.07–1.28)	0.00072
Somewhat important (4–6)	27.7%	52.98% (52.13–53.83)	1.52 (1.43–1.61)	<0.0001	1.3 (1.19–1.42)	<0.0001
Very (7–9)	30.43%	41.52% (40.74–42.31)	0.96 (0.9–1.01)	0.12	1.22 (1.11–1.35)	<0.0001
Extremely (10)	8.32%	42.97% (41.44–44.51)	1.01 (0.94–1.1)	0.73	1.06 (0.94–1.19)	0.33
Prefer not to answer	5.06%	50.42% (48.37–52.47)	1.37 (1.24–1.5)	<0.0001	1.6 (1.39–1.84)	<0.0001
**Perceived NHS quality**^c^ (from 0 = worst to 10 = best)	5.73 (0.009)	–	0.78 (0.77–0.79)	<0.0001	0.9 (0.89–0.92)	<0.0001
**Perceived NHS access**^c^ (from 0 = least accessible to 10 = most accessible)	6.01 (0.010)	–	0.79 (0.79–0.8)	<0.0001	0.91 (0.9–0.92)	<0.0001
**Block 6—Survey mode**						
**Survey mode**		<0.0001				
CAWI	71.42%	50.61% (50.09–51.14)	Ref.			
CATI	28.58%	34.79% (34.01–35.57)	0.52 (0.5–0.54)	<0.0001	0.77 (0.72–0.83)	<0.0001

(post-stratification weights for age group, geographic area, and municipality size applied).

Abbreviations: adjOR, adjusted Odds Ratio; CATI, Computer-Assisted Telephone Interviewing; CAWI, Computer-Assisted Web Interviewing; CI, Confidence Interval; NHS, National Health Service; OR, Odds Ratio.

a p-value in this column were obtained via Chi-squared tests.

b The multivariable regression model reported in Table 1 was adjusted for all the variables presented in Tables 1 and 2 (Block 1–6). Post-stratification weights for age group, geographic area, and municipality size are applied (unweighted model in Tables S1 and S2, comparison between weighted and unweighted models in Table S3). Note: Regression estimates should not be interpreted as causal effects.

c Variables expressed as mean and standard error in brackets. Group differences were assessed using independent samples t-tests (all comparisons: p < 0.001).

### Statistical analysis

Descriptive analyses were executed for all variables.

The primary outcome (VH) prevalence was described with the corresponding 95% confidence interval (CI). Prevalence of VH was reported across all independent variables, and chi-squared tests (or t-tests for quantitative variables) were used to examine associations. Univariable logistic regressions were also calculated to estimate crude odds ratios (ORs). Post-stratification weights were applied to align the sample with the Italian adult population: we constructed post-strata based on the joint distribution of age, geographical macro-area, and municipality size. Gender and education were not included in post-stratification due to lack of harmonised public benchmarks matching our survey categories. Details of post-stratification weighting in Supplementary Methods M4.

We computed a multivariable logistic regression model, with the primary analytical aim of assessing the strength of association of each determinant with VH, while accounting for the influence of the others. We chose this approach to quantify the unique association of each variable category with VH, reflecting its multidimensional nature and supporting the identification of subgroups that may require prioritisation in targeted public health strategies. For this reason, all domains identified a priori based on existing literature (Supplementary Methods 3) were included in the final model. No causal ordering among determinants was assumed, as they were conceptualised as parallel contributors to hesitancy rather than as variables lying on a causal pathway between each other. The VCBS was the only exception: it was included only in the sensitivity analysis to avoid adjusting for a possible mediator and to evaluate the extent to which associations were independent of conspiratorial thinking.

Thus, a multivariable logistic regression model was developed using a hierarchical approach in six blocks: (1) sociodemographic and socioeconomic characteristics, (2) health-related characteristics and personal experiences, (3) information sources, (4) external influences, (5) beliefs and attitudes, and (6) survey mode. Collinearity was excluded using the Variance Inflation Factor (VIF): the mean VIF was 1.67 (all values < 5, Supplementary Methods 5.1). Model fit was evaluated using the log-likelihood, likelihood ratio chi-square statistics, degrees of freedom, and pseudo R^2^. Likelihood ratio tests were used to compare adjacent models. The final model showed good performance, supported by multiple diagnostics, including influential observation analysis (Pregibon's delta-beta), discrimination, calibration, and bootstrap internal validation, with full methods and results reported in the Supplementary Methods M4 and M5. Post-stratification weights were applied to the final model.

Predicted probabilities of VH were computed from the final model using predictive margins. These were visualised graphically to aid interpretation. Predicted probabilities were estimated as model-adjusted, population-averaged risks (Supplementary Methods M4). For categorical variables with more than two levels, pairwise comparisons of predicted probabilities were performed: pairwise contrasts quantified absolute differences between categories, with p-values adjusted using the Bonferroni correction (details in Supplementary Methods M4). Post-stratification weights were applied also to predicted probabilities and pairwise contrasts.

For secondary outcomes (aVHS subscales), separate univariable and multivariable linear regression models were performed. Multivariable models, including all the above-mentioned blocks, were estimated both with and without VCBS. In linear regressions partial eta-squared (η^2^) was calculated as a measure of effect size. Assumptions for linear regression were evaluated, including linearity, residual normality, and homoscedasticity. Minor deviations from normality were observed, while heteroskedasticity was detected and addressed by using robust standard errors. Full diagnostics are reported in the Supplementary Methods (M4 and M5). Post-stratification weights were applied to the aVHS subscales multivariable models.

Two sensitivity analyses were conducted for the primary outcome. First, a seventh block including the VCBS was added to the regression model to assess which groups had a higher probability of VH independently of conspiracy beliefs, treating these beliefs as one component of hesitancy. Second, the aVHS score was analysed as a continuous outcome using linear regression models, with and without the inclusion of VCBS, to assess the robustness of our results and to preserve the full informational content of the scale. Linear regression diagnostics were performed using the same procedures applied to the aVHS subscales (Supplementary Methods M5). Post-stratification weights were applied to the multivariable regression models used in the sensitivity analyses.

Overall, variables were entered in the regression models by forced entry, and missing data were handled by listwise deletion. Post-stratification weighted results are presented as the main analyses in the text, while complete unweighted analyses are provided in the Supplementary Materials. Weighted and unweighted multivariable regression estimates were compared, with percentage differences between adjusted OR (or coefficients) computed to describe consistency.

All analyses were conducted using Stata (Versions 18 and 19). Figures were created with Excel 2019. A two-sided p-value <0.050 was considered statistically significant.

### Ethical approval

The National Ethics Committee for Research Institutions and other national public bodies approved the study (Protocol No. 0023087, 28th of May 2024). Online written informed consent (CAWI) or recorded verbal consent (CATI) was obtained from all participants.

### Role of the funding source

The funder of the study had no role in study design, data collection, data analysis, data interpretation, or writing of the report.

## Results

The survey agency provided a dataset including only completed questionnaires, for a total of 52,094 respondents (70.3% CAWI). The descriptive analyses (with post-stratification weighting) of Blocks 1 and 2 are presented in the first column of Table 1, Blocks 3–6 in Table 2.

A total of 23,844 participants (poststratification weighted prevalence: 46.09% (95% CI: 45.65%–46.53%); unweighted prevalence: 45.77%, 95% CI: 45.34–46.20%) were labelled as “vaccine hesitant”. The weighted mean aVHS score was 24 (unweighted: 24, SD = 8.92). The second columns of Tables 1 and 2 reports the weighted prevalence of VH across categories. Chi-square tests and univariable logistic regressions showed significant associations between VH and all variables, except for continent of citizenship (Tables 1 and 2). Unweighted results corresponding to Tables 1 and 2 are presented in Tables S1 and S2. A comparison of unweighted and post-stratification weighted VH prevalence is presented in Table S3. Only three categories showed percentage differences higher than 5% between weighted and unweighted estimates: reported barriers to vaccination (+18.2%), age ≥75 years (+6.4%), and African American ethnicity (+6.3%).

The unweighted hierarchical logistic regression model (Table S4) showed progressive and significant improvement in fit across blocks (fit statistics in Table S5). The addition of Block 2 produced the largest gain in explanatory power (Pseudo R^2^ from 0.034 to 0.116). Blocks 3–5 improved the model, bringing the Pseudo R^2^ to 0.233. Block 6 had limited influence. The weighted final model is reported in Tables 1 and 2. The strongest associations with VH were observed for knowing someone who had experienced an AEFI (adjOR = 3.44), using CAM as an alternative to conventional medicine (adjOR = 2.24), identifying as Hindu (adjOR = 2.24), and reporting a non-binary/other gender identity (adjOR = 2.03).

When comparing the final unweighted and weighted models (Table S6), adjOR were largely consistent across all determinants. Percentage differences between the adjORs were below 10% (exception: African American ethnicity, +49.8%). Associations between variables and VH showed the same direction and statistical significance in both models, except for: living in Southern Italy (lower VH) and being a homemaker (higher VH) were significant only in the unweighted model, whereas having at least one chronic condition (lower VH) reached significance only in the weighted model.

**Fig. 1:**
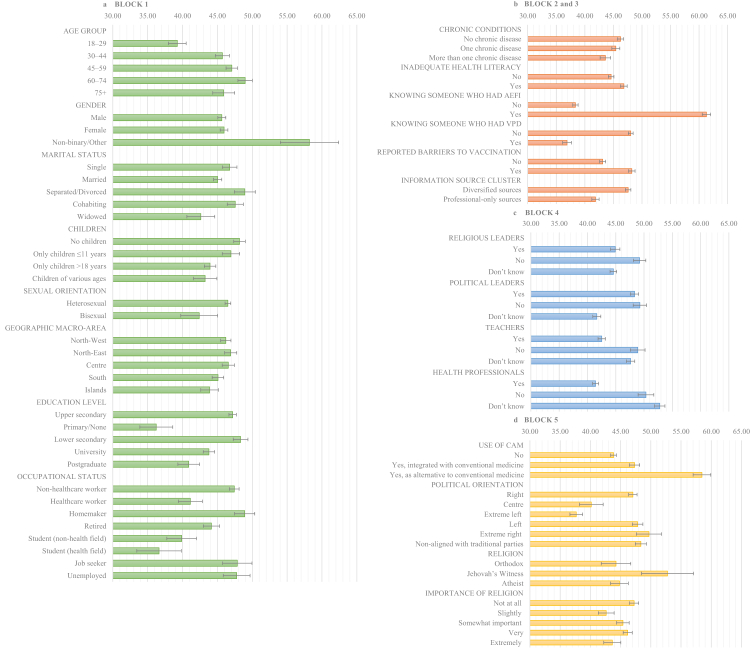
**Poststratification weighted predicted probabilities of vaccine hesitancy (95% CI)**. Fig. 1 presents the poststratification weighted predicted probabilities of vaccine hesitancy (percentage and 95% Confidence Interval, CI) for variables of blocks 1 (a) (Sociodemographic and socioeconomic characteristics), 2 and 3 (b)/Health-related characteristics and personal experience; information sources), 4 (c) (External influences: perceived vaccination endorsement in the respondent's community), 5 (d) (Beliefs and attitudes) based on the final multivariable logistic regression model with Block 1–6. To enhance clarity and readability, displayed categories are limited to categories involved in at least one pairwise contrast that was statistically significant after Bonferroni correction (for variables with >2 levels) (Table S9), or with a significant overall association if binary (Tables 1 and 2). Continuous variables, survey mode, and “Prefer not to answer”, “Other” response options were excluded from the graphs. All poststratification weighted predicted probabilities are reported in Tables S7. Abbreviations: AEFI, Adverse Event Following Immunization; CI, Confidence Interval; VPD, Vaccine-Preventable Disease.

Predicted probabilities of VH based on the final weighted model are in Table S7 (based on the unweighted model: Table S8). The ten subgroups with the highest probability (from 61.90% to 50.85%) included those who reported knowing someone who experienced AEFI, Hindu individuals, respondents using CAM in place of conventional care, those identifying as non-binary/other, Jewish participants, African American individuals, those unaware of any pro-vaccine HCWs, Jehovah's Witnesses, North American/Australian individuals, and Protestants. Fig. 1 presents the weighted VH predicted probabilities showing only categories involved in at least one significant pairwise contrast after Bonferroni correction (variables with >2 levels) (weighted comparisons: Table S9; unweighted comparisons: Table S10), or with a significant association at the regression model (binary variables) (Tables 1 and 2). The highest increase was observed among those using CAM as a replacement for conventional care, with a 14.6-point difference from non-users. Focussing on the largest significant differences (>10 percentage points), lower hesitancy was observed among health students compared with non-HCWs, homemakers, unemployed, and job seekers. Individuals identifying as non-binary showed higher hesitancy than men and women. Respondents aligned with the extreme right, right, or non-aligned exhibited hesitancy higher than left-aligned participants. Individuals unsure whether HCWs in their community support vaccination showed greater hesitancy than those perceiving support. People with middle school education and upper secondary education had higher hesitancy than those with elementary education.

Considering our secondary outcomes, the “Lack of trust” subscale showed a weighted mean of 15.29 (unweighted: 15.26, SD = 7.41), while the “Risk perception” subscale had a mean of 8.97 (unweighted: 8.97, SD = 3.37). The aVHS total score was strongly correlated with “Lack of trust” (r = 0.931) and moderately with “Risk perception” (r = 0.599). VCBS was correlated with “Lack of trust” (r = 0.613) and “Risk perception” (r = 0.487) (all correlations: p < 0.0001).

Tables 3 and 4 shows the multivariable linear regression models of the two aVHS subscales with poststratification weights. Tables S11 and S12 report univariable regressions and details of weighted multivariable models (unweighted models: Tables S13 and S14; comparisons between weighted and unweighted models: Table S15). Most variables were significantly associated with the subscales showing similar relationships, although effect sizes were generally small or lower than small. Some variables showed significant associations in opposite directions across the two models (both considering weighted and unweighted analyses). For instance, respondents identifying as non-binary/other reported greater distrust but lower perceived risk. Individuals with multiple chronic illnesses were less likely to report a lack of trust, yet more likely to perceive risk. Inadequate HL was inversely associated with lack of trust but positively associated with perceived risk. Religious importance was not significantly associated with lack of trust, but positively associated with the risk subscale. Only in the weighted model, being from Southern Italy was negatively associated with lack of trust and positively associated with risk perception. The unweighted models also highlighted that female gender was associated with a lower lack of trust, but with a higher perceived risk; living with someone frail was associated with lower lack of trust but higher risk perception. When the VCBS was added to both models (Tables S11–S14), it emerged as the strongest predictor for both subscales, with large effect sizes (“Lack of trust”: η^2^ = 0.220; “Risk perception”: η^2^ = 0.171).

**Table 3:** Sociodemographic, socioeconomic, health-related characteristics and personal experience: linear regression models for “Lack of trust” and “Risk perception” subscales of the adult Vaccine Hesitancy Scale (aVHS).

	Lack of trust subscale model^a^		Risk perception subscale model^a^	
	adjCoef (95% CI)	p	adjCoef (95% CI)	p
**Block 1–Sociodemographic and socioeconomic characteristics**				
**Age group**				
18–29	Ref.		Ref.	
30–44	1.23 (1.02–1.44)	<0.0001	0.29 (0.19–0.4)	<0.0001
45–59	1.5 (1.28–1.72)	<0.0001	0.44 (0.34–0.55)	<0.0001
60–74	1.74 (1.45–2.03)	<0.0001	0.61 (0.47–0.75)	<0.0001
75+	1.06 (0.72–1.4)	<0.0001	0.82 (0.65–0.99)	<0.0001
**Gender**				
Male	Ref.		Ref.	
Female	−0.11 (−0.22 to 0.01)	0.071	0.34 (0.28–0.4)	<0.0001
Non-binary/Other	1.73 (1.13–2.32)	<0.0001	−0.53 (−0.82 to −0.24)	0.00035
Prefer not to answer	1.29 (−0.45 to 3.03)	0.15	−1.34 (−2.28 to −0.4)	0.0052
**Marital status**				
Single	Ref.		Ref.	
Married	−0.32 (−0.51 to −0.12)	0.0014	−0.03 (−0.12 to 0.07)	0.595
Separated/Divorced	0.55 (0.26–0.85)	0.00022	0.01 (−0.13 to 0.14)	0.93
Cohabiting	0.1 (−0.11 to 0.32)	0.35	0.15 (0.05–0.25)	0.0044
Widowed	−0.42 (−0.76 to −0.08)	0.015	−0.09 (−0.27 to 0.09)	0.31
**Children**				
No children	Ref.		Ref.	
Only children ≤11 years	−0.34 (−0.55 to −0.14)	0.0012	0.07 (−0.03 to 0.17)	0.16
Only children 12–18 years	−0.55 (−0.81 to −0.29)	<0.0001	0.11 (−0.01 to 0.24)	0.077
Only children >18 years	−0.63 (−0.84 to −0.42)	<0.0001	−0.25 (−0.35 to −0.15)	<0.0001
Children of various ages	−0.75 (−1.03 to −0.47)	<0.0001	0.03 (−0.11 to 0.17)	0.68
**Sexual orientation**				
Heterosexual	Ref.		Ref.	
Homosexual	−0.4 (−0.86 to 0.06)	0.088	−0.27 (−0.48 to −0.05)	0.016
Bisexual	−0.72 (−1.11 to −0.34)	0.00024	−0.21 (−0.4 to −0.02)	0.032
Pansexual	−0.66 (−1.26 to −0.06)	0.031	−0.47 (−0.76 to −0.17)	0.00199
Ace spectrum	−0.81 (−1.41 to −0.21)	0.0082	−0.11 (−0.39 to 0.17)	0.45
Prefer not to answer	−1.54 (−1.83 to −1.26)	<0.0001	1.16 (1.02–1.29)	<0.0001
**Municipality size (inhabitants)**				
≤10,000	Ref.		Ref.	
10,001–25,000	−0.08 (−0.23 to 0.08)	0.35	−0.01 (−0.08 to 0.07)	0.88
25,001–50,000	−0.12 (−0.31 to 0.07)	0.21	0.01 (−0.08 to 0.1)	0.84
50,001–100,000	0.11 (−0.15 to 0.38)	0.41	0.11 (−0.02 to 0.25)	0.094
100,001–250,000	0.1 (−0.22 to 0.42)	0.55	0.14 (−0.02 to 0.3)	0.094
>250,000	−0.11 (−0.4 to 0.18)	0.47	0.09 (−0.06 to 0.23)	0.25
**Geographic macro-area**				
North-West	Ref.		Ref.	
North-East	0.28 (0.11–0.44)	0.001	−0.07 (−0.15 to 0.01)	0.09
Centre	−0.05 (−0.22 to 0.11)	0.52	0.21 (0.13–0.29)	<0.0001
South	−0.43 (−0.6 to −0.27)	<0.0001	0.09 (0.01–0.17)	0.036
Islands	−0.57 (−0.79 to −0.35)	<0.0001	−0.29 (−0.4 to −0.18)	<0.0001
**Degree of urbanisation**				
Pole	Ref.		Ref.	
Intermunicipal pole	−0.19 (−0.57 to 0.18)	0.31	0.12 (−0.07 to 0.31)	0.22
Belt	0.22 (−0.01 to 0.44)	0.061	0 (−0.11 to 0.12)	0.98
Intermediate	0.16 (−0.1 to 0.43)	0.22	−0.08 (−0.22 to 0.05)	0.22
Peripheral	0.16 (−0.14 to 0.46)	0.302	−0.01 (−0.16 to 0.15)	0.94
Ultra-peripheral	0.07 (−0.44 to 0.58)	0.79	−0.02 (−0.28 to 0.25)	0.89
**Education level**				
Upper secondary	Ref.		Ref.	
Primary/None	−0.94 (−1.32 to −0.56)	<0.0001	−0.51 (−0.73 to −0.28)	<0.0001
Lower secondary	0.27 (0.09–0.45)	0.0033	0.33 (0.24–0.42)	<0.0001
University	−0.41 (−0.55 to −0.27)	<0.0001	−0.36 (−0.42 to −0.29)	<0.0001
Postgraduate	−0.57 (−0.81 to −0.33)	<0.0001	−0.37 (−0.49 to −0.25)	<0.0001
**Occupational status**				
Non-healthcare worker	Ref.		Ref.	
Healthcare worker	−1.22 (−1.46 to −0.98)	<0.0001	−0.28 (−0.41 to −0.15)	<0.0001
Homemaker	0.22 (−0.01 to 0.46)	0.065	−0.08 (−0.19 to 0.03)	0.18
Retired	−0.48 (−0.71 to −0.24)	<0.0001	0.03 (−0.08 to 0.14)	0.56
Student (non-health field)	−0.81 (−1.11 to −0.51)	<0.0001	−0.62 (−0.77 to −0.47)	<0.0001
Student (health field)	−1.42 (−1.82 to −1.02)	<0.0001	−0.83 (−1.04 to −0.62)	<0.0001
Job seeker	0.1 (−0.21 to 0.42)	0.52	−0.02 (−0.17 to 0.13)	0.79
Unemployed	0.21 (−0.09 to 0.51)	0.17	0 (−0.14 to 0.14)	0.99
Other	0.44 (−1.14 to 2.01)	0.59	−0.12 (−0.72 to 0.49)	0.71
**Continent of citizenship**				
Italy	Ref.		Ref.	
Europe (non-Italy)	0.11 (−0.42 to 0.64)	0.69	−0.41 (−0.67 to −0.14)	0.0032
Africa	2.11 (0.82–3.39)	0.0013	−0.38 (−1.01 to 0.25)	0.24
America	−0.12 (−1.28 to 1.04)	0.84	−0.33 (−1 to 0.34)	0.34
Asia	1.08 (−0.35 to 2.51)	0.14	0.53 (−0.11 to 1.17)	0.11
Oceania	−9.14 (−10.62 to −7.66)	<0.0001	−2.04 (−2.74 to −1.35)	<0.0001
**Self-identified ethnicity**				
European	Ref.		Ref.	
Multi-ethnic	0.12 (−0.53 to 0.77)	0.71	−0.45 (−0.75 to −0.14)	0.0045
North American/Australian	1.04 (−0.09 to 2.18)	0.072	−0.4 (−0.95 to 0.15)	0.15
Arab-Middle Eastern	−0.37 (−1.22 to 0.47)	0.39	−0.57 (−1.01 to −0.14)	0.01001
North African	−0.7 (−1.59 to 0.18)	0.12	−0.29 (−0.73 to 0.14)	0.18
Latino-American	−0.1 (−0.89 to 0.69)	0.804	−0.32 (−0.72 to 0.09)	0.12
African American	−0.11 (−2.03 to 1.81)	0.91	−0.35 (−1.2 to 0.49)	0.41
Black African	−0.88 (−2.24 to 0.48)	0.21	−0.24 (−0.96 to 0.47)	0.51
Asian	−0.78 (−1.91 to 0.34)	0.17	−0.7 (−1.29 to −0.11)	0.0195
Pacific Islands	−0.76 (−2.93 to 1.41)	0.49	−1.33 (−2.49 to −0.18)	0.024
**Material deprivation**				
No deprivation	Ref.		Ref.	
Severe deprivation	0.2 (−0.1 to 0.51)	0.19	0.03 (−0.11 to 0.18)	0.65
**Block 2–Health-related characteristics and personal experience**				
**Chronic conditions**				
No chronic disease	Ref.		Ref.	
One chronic disease	−0.27 (−0.4 to −0.14)	<0.0001	−0.06 (−0.12 to 0.01)	0.095
More than one chronic disease	−0.52 (−0.7 to −0.35)	<0.0001	0.58 (0.5–0.67)	<0.0001
**Living with a person with disability**				
No	Ref.		Ref.	
Yes	−0.19 (−0.34 to −0.03)	0.017	0.05 (−0.02 to 0.12)	0.17
**Inadequate health literacy**				
No	Ref.		Ref.	
Yes	−0.38 (−0.5 to −0.26)	<0.0001	0.58 (0.52–0.63)	<0.0001
**Knowing someone who had AEFI**				
No	Ref.		Ref.	
Yes	4.2 (4.05–4.34)	<0.0001	1.44 (1.38–1.51)	<0.0001
**Knowing someone who had VPD**				
No	Ref.		Ref.	
Yes	−1.95 (−2.09 to −1.8)	<0.0001	−0.42 (−0.49 to −0.35)	<0.0001
**Reported barriers to vaccination**				
No	Ref.		Ref.	
Yes	0.33 (0.21–0.45)	<0.0001	0.45 (0.39–0.51)	<0.0001

(post-stratification weights for age group, geographic area, and municipality size applied).

Abbreviations: adjOR, adjusted Odds Ratio; AEFI, Adverse Event Following Immunisation; CI, Confidence Interval; OR, Odds Ratio; VPD, Vaccine Preventable Disease.

a The multivariable regression model reported in Table 3 was adjusted for all the variables presented in Tables 3 and 4 (Block 1–6). Post-stratification weights for age group, geographic area, and municipality size are applied (unweighted model in Tables S13 and S14, comparison between weighted and unweighted models in Table S15). Note: Regression estimates should not be interpreted as causal effects.

**Table 4:** Information sources and trust, external influences, beliefs, attitudes and survey mode: linear regression models for “Lack of trust” and “Risk perception” subscales of the adult Vaccine Hesitancy Scale (aVHS).

	Lack of Trust subscale model^a^		Risk Perception subscale model^a^	
	adjCoef (95% CI)	p	adjCoef (95% CI)	p
**Block 3–Information sources and trust**				
**Information source cluster**				
Diversified sources	Ref.		Ref.	
Professional-only sources	−0.47 (−0.59 to −0.34)	<0.0001	−0.51 (−0.57 to −0.45)	<0.0001
**Trust in sources** (from 1 = not at all to 4 = very much)	−2.38 (−2.49 to −2.28)	<0.0001	−0.47 (−0.52 to −0.42)	<0.0001
**Block 4—External influences (perceived vaccination endorsement in the respondent's community by:)**				
**By religious leaders**				
Yes	Ref.		Ref.	
No	0.24 (0.05–0.44)	0.015	0.34 (0.25–0.44)	<0.0001
Don't know	−0.18 (−0.34 to −0.02)	0.026	0.22 (0.14 to 0.3)	<0.0001
**By political leaders**				
Yes	Ref.		Ref.	
No	−0.38 (−0.58 to −0.19)	0.00014	−0.26 (−0.35 to −0.16)	<0.0001
Don't know	−1.18 (−1.34 to −1.02)	<0.0001	−0.48 (−0.56 to −0.4)	<0.0001
**By teachers**				
Yes	Ref.		Ref.	
No	0.89 (0.68–1.11)	<0.0001	−0.02 (−0.12 to 0.08)	0.74
Don't know	1.03 (0.87–1.19)	<0.0001	−0.26 (−0.34 to −0.18)	<0.0001
**By health professionals**				
Yes	Ref.		Ref.	
No	1.21 (0.99–1.43)	<0.0001	0.32 (0.21–0.42)	<0.0001
Don't know	1.48 (1.31–1.64)	<0.0001	0.74 (0.66–0.82)	<0.0001
**Block 5—Beliefs and attitudes**				
**Use of non-conventional medicine**				
No	Ref.		Ref.	
Yes, integrated with conventional medicine	0.26 (0.12–0.4)	0.00028	−0.02 (−0.09 to 0.05)	0.56
Yes, as alternative to conventional medicine	2.4 (2.17–2.62)	<0.0001	0.67 (0.57–0.77)	<0.0001
**Political orientation**				
Right (7–9)	Ref.		Ref.	
Centre (4–6)	−0.1 (−0.25 to 0.06)	0.21	−0.35 (−0.42 to −0.27)	<0.0001
Extreme left (0)	−0.76 (−1.1 to −0.42)	<0.0001	−0.79 (−0.95 to −0.62)	<0.0001
Left (1–3)	−1.12 (−1.31 to −0.93)	<0.0001	−0.89 (−0.99 to −0.79)	<0.0001
Extreme right (10)	0.89 (0.53–1.26)	<0.0001	0.56 (0.39–0.72)	<0.0001
Non-aligned with traditional parties	0.67 (0.48–0.86)	<0.0001	0.33 (0.24–0.43)	<0.0001
Prefer not to answer	−0.15 (−0.38 to 0.08)	0.21	−0.32 (−0.44 to −0.19)	<0.0001
**Religion**				
Catholic	Ref.		Ref.	
Orthodox	−0.61 (−0.95 to −0.28)	0.00032	0.64 (0.47–0.8)	<0.0001
Protestant	0.86 (0.23–1.49)	0.0071	−0.22 (−0.51 to 0.06)	0.13
Jewish	1.58 (0.64–2.51)	0.00096	−0.62 (−1.15 to −0.09)	0.022
Muslim	0.84 (0.27–1.42)	0.00395	0.29 (0.01–0.56)	0.042
Jehovah's Witness	0.56 (−0.05 to 1.18)	0.073	0.08 (−0.18 to 0.34)	0.55
Atheist	−0.01 (−0.26 to 0.23)	0.92	−0.16 (−0.28 to −0.04)	0.0066
Agnostic	−0.1 (−0.43 to 0.22)	0.53	−0.14 (−0.3 to 0.01)	0.067
Buddhist	1.43 (0.64–2.23)	0.0004	−0.06 (−0.42 to 0.3)	0.74
Hindu	2.39 (0.85–3.93)	0.0023	0.41 (−0.29 to 1.11)	0.25
Other	1.75 (1.28–2.21)	<0.0001	0.29 (0.08–0.5)	0.0069
Prefer not to answer	0.82 (0.54–1.11)	<0.0001	−0.09 (−0.24 to 0.05)	0.198
**Importance of religion**				
Not at all (0)	Ref.		Ref.	
Slightly (1–3)	0.25 (0.01–0.49)	0.041	0.2 (0.09–0.32)	0.00056
Somewhat important (4–6)	−0.08 (−0.33 to 0.16)	0.501	0.47 (0.35–0.59)	<0.0001
Very (7–9)	−0.14 (−0.4 to 0.11)	0.27	0.43 (0.3–0.55)	<0.0001
Extremely (10)	−0.16 (−0.48 to 0.16)	0.34	0.45 (0.3–0.6)	<0.0001
Prefer not to answer	0.17 (−0.21 to 0.54)	0.38	1.41 (1.22–1.6)	<0.0001
**Perceived NHS quality** (from 0 = worst to 10 = best)	−0.45 (−0.5 to −0.41)	<0.0001	−0.04 (−0.06 to −0.02)	<0.0001
**Perceived NHS access** (from 0 = least accessible to 10 = most accessible)	−0.3 (−0.34 to −0.26)	<0.0001	0.03 (0.01–0.05)	0.0071
**Block 6—Survey mode**				
**Survey mode**				
CAWI	Ref.		Ref.	
CATI	−0.87 (−1.05 to −0.7)	<0.0001	−0.19 (−0.27 to −0.1)	<0.0001

(post-stratification weights for age group, geographic area, and municipality size applied).

Abbreviations: adjOR, adjusted Odds Ratio; CATI, Computer-Assisted Telephone Interviewing; CAWI, Computer-Assisted Web Interviewing; CI, Confidence Interval; NHS, National Health Service; OR, Odds Ratio.

a The multivariable regression model reported in Table 4 was adjusted for all the variables presented in Tables 3 and 4 (Block 1–6). Post-stratification weights for age group, geographic area, and municipality size are applied (unweighted model in Tables S13 and S14, comparison between weighted and unweighted models in Table S15). Note: Regression estimates should not be interpreted as causal effects.

Table S4 presents also the unweighted sensitivity analysis adjusting for the VCBS (Block 7, Pseudo R^2^ = 0.361), which showed a relationship between VH and VCBS (adjOR = 2.08, 95% CI = 2.05–2.12). Table S16 shows the weighted model, confirming a strong relationship (adjOR = 2.08, 95% CI = 2.04–2.12) (comparison between models in Table S17). Most variables retained similar directions and significance levels compared with the main model. Some significant changes emerged: living in belt or intermediate areas became associated with higher hesitancy; both centrists and non-aligned individuals became more hesitant than those on the right, while extreme right participants were less hesitant; being extremely religious became significantly protective.

The sensitivity analysis using the continuous aVHS score is in Tables S18–S20. Most associations had the same direction as those observed in the main model, although some changed in strength or statistical significance. Effect sizes were generally small, except for the VCBS score (η^2^ = 0.325).

## Discussion

This study primarily aimed to provide an updated estimate of VH in the Italian adult population. Using a large and representative sample, nearly half of respondents were vaccine hesitant. Our findings highlighted the complexity of VH, helping to identify priority groups.

Our VH prevalence was generally higher than VH found using the same cut-off in the general population. Prior research found 27.5% in post-pandemic Italy,^9^ and, during the pandemic, 37.7% in Saudi Arabia,^10^ and 22–59% across China, the USA, and Taiwan.^11^ These differences reflect the dynamic, context-dependent nature of VH,^1^ likely influenced by timing, sample representativeness, and socio-political context. The increase in VH of our sample may be partly attributable to differences in methodology and sample size, but may also reflect a shift in attitudes following the pandemic, consistently with European trends. Indeed, this pattern aligns with the 2022 State of Vaccine Confidence report.^13^ Compared with 2020, this report documented a decline in agreement regarding importance, effectiveness, and compatibility of vaccines with personal beliefs.^13^ In Italy, confidence in vaccine importance declined, while perceptions on safety remained stable, suggesting that the VH rise may be more associated with increasing complacency rather than reduced trust in safety.^14^ Nevertheless, our data highlighted that trust (particularly institutional) remains a key determinant. For instance, lower perceived NHS quality was significantly associated with hesitancy, suggesting VH currently may stem less from doubts about vaccine safety and more from a lack of trusted messengers able to convey the importance of vaccination. Therefore, VH remains a challenge in Italy, needing continuous monitoring.

Unexpectedly, VH was higher among adults over 30, especially in the 60–74 age group, an important vaccination target. The association emerged after adjusting for variables like health conditions, suggesting that the lower hesitancy in older adults in previous research^15^ may reflect other protective factors. Our findings on marital status aligned with most works.^15^ Marital status may influence vaccination attitudes by shaping perceived benefits, barriers, and social cues.^16^ Parenthood appeared protective: vaccine attitudes can evolve with parenting experience, as confidence in decision-making and trust in vaccines may increase over time.^17^ Lower hesitancy was found in Southern Italy and the Islands. Given the structural disadvantages in the South,^18^ a possible limited uptake may reflect systemic and organisational barriers rather than attitudes. Moreover, prior reviews reported higher VH in Northern Italy.^5^^,^^6^ Specific areas as South Tyrol (North-East), which records the lowest vaccination coverage nationwide, present unique contextual factors: VH has been linked to low institutional trust, strong individualistic values, widespread CAM use, and language-based differences in access to health information.^19^^,^^20^ These findings highlight the importance of considering sub-national heterogeneity and the need for context-sensitive strategies. Education showed a U-shaped pattern: hesitancy was lower among the least and most educated. Light et al.^21^ showed that individuals with more years of education tend to overestimate their knowledge, and this may explain higher hesitancy among those with intermediate education. Higher education and HCW status were linked to lower hesitancy,^15^ possibly indicating exposure to environments promoting critical thinking and trust in evidence-based sources. Gender and sexual identity were associated with VH. Consistent with prior research,^22^ individuals identifying as non-binary/other gender reported one of the highest VH, possibly due to mistrust in healthcare linked to marginalisation, while bisexual participants were less hesitant, potentially reflecting stronger engagement with health-promoting networks. Subscale analyses supported these interpretations, with non-binary respondents showing higher lack of trust but lower perceived risk. However, non-binary data should be interpreted cautiously, as many responses came from older individuals, suggesting possible misclassification.

Individuals with chronic conditions were less hesitant, as previously reported,^15^ but showed higher risk perception, highlighting the need to address safety concerns. Participants with inadequate HL showed higher VH, which was driven more by fear than by institutional distrust: higher HL may reduce belief in misinformation,^23^ supporting efforts to target false beliefs in low-literacy groups. Findings on personal experiences aligned with the “3C” model.^1^ Perceived barriers and the opinion on the NHS reflected convenience issues, highlighting the need to address service-related determinants. Knowing someone affected by VPD may reduce complacency, whereas knowing someone who experienced AEFI strongly increased hesitancy. This may result from cognitive biases, which could also explain the higher hesitancy among participants using non-professional sources, such as social media.^24^

No perceived pro-vaccination support, or lack of awareness of support, from HCWs, teachers, and religious leaders was associated with VH. HCW recommendation plays a well-established role,^25^ with participants perceiving no support from HCWs showing one of the highest predicted hesitancy. While teachers are key figures, evidence on their role is limited; training them to address misinformation and promote vaccine literacy may be beneficial.^26^ Religious leaders’ role reinforces evidence that faith-based support can improve vaccination rates.^27^ Hesitancy followed a non-linear pattern, being higher among moderately religious individuals, possibly reflecting greater trust in science among non-religious^28^ or stronger alignment with community norms and religious leadership among the highly religious.^27^ Indeed, higher religious importance was associated with risk perception, not distrust. Though not all associations remained significant after Bonferroni correction, predicted hesitancy was higher among many religious groups known to face cultural or theological barriers.^29^

Not knowing local politicians’ stance was associated with lower hesitancy, possibly indicating political disengagement or rejection of politicised health messages. The influence of politics on VH is context-dependent, with studies showing divergent patterns.^30^^,^^31^ In Italy, right-wing orientation was associated with lower trust in science and higher hesitancy.^32^ We confirmed this and found high hesitancy among centrists and the politically unaffiliated, suggesting that depoliticised and unified health communication may help reduce hesitancy in polarised contexts.

Individuals using CAM instead of conventional care had one of the highest predicted probabilities. Previous research linked this to distrust in biomedicine rather than confidence in CAM.^33^ Reaching these groups may require engaging CAM-sensitive professionals and offering counselling in trusted settings, warranting further investigation.

Although several associations lost significance after Bonferroni correction, many ethnic groups showed high predicted hesitancy. Despite very small samples, these patterns are relevant, as ethnic disparities in vaccine attitudes remain underexplored in Italy, with Italian data absent from relevant literature.^34^

Adjusting for VCBS revealed profiles less influenced by conspiracy beliefs. As expected, VCBS was a strong VH predictor,^12^ particularly for lack of trust. Most associations remained consistent, but new patterns emerged. Hesitancy became higher in belt/intermediate areas, suggesting geographic barriers related to distance to immunization sites^35^ previously masked by VCBS. The effect of knowing someone with AEFI largely decreased, indicating that conspiracy beliefs may amplify perceived severity and shape how such events are remembered. Hesitancy was higher among centrists and the politically unaffiliated than right-wing participants, possibly reflecting broader disengagement or mistrust in institutions rather than conspiracy beliefs. The extreme right showed lower hesitancy than the broader right-wing group, suggesting that their hesitancy was largely explained by VCBS. Being extremely religious appeared protective, possibly indicating strong community support and guidance from religious institutions.

Considering the strength of associations, several variables showed adjORs >2 (e.g., knowing someone with AEFI, CAM, non-binary identity, and conspiracy beliefs) supporting their relevance. For the aVHS subscales, most effect sizes were small/lower than small, while conspiracy beliefs emerged as the strongest predictor across subscales. However, public health relevance should not be judged solely by effect magnitude: even small effects, especially when linked to widespread factors, can have meaningful implications for vaccination uptake at the population level.

This study had several limitations. The cross-sectional design precludes any inference about causality. Although quotas reflected national demographics, non-probability sampling limits generalisability; post-stratification weighting improved population alignment, but residual imbalances in population representativeness may remain due to unavailable benchmarks for gender and education. Several subgroups defined by self-reported nationality or ethnicity were small and non-representative, limiting estimate precision. Subgroups such as gender-diverse individuals and certain ethnic or religious minorities represented less than 1% of the sample: related findings should be interpreted cautiously, and targeted research is warranted.

The mixed-mode recruitment may have methodological limitations: CAWI relied on a voluntary web panel, potentially affected by self-selection and variable engagement, while CATI may have introduced mode-related effects. Response rates, although compatible with large-scale web surveys, suggest possible non-response bias, particularly if participation varied by vaccination attitudes. Despite measures to reduce bias, residual effects cannot be excluded. Non-probability online sampling may have introduced selection bias, partly mitigated by CATI, and differential participation by vaccination attitudes may have affected VH prevalence in uncertain directions. The reversal of the survey–mode association after adjustment for VCBS suggests possible mode effects and social desirability bias. Recall bias was likely minimal given the focus on current attitudes and recent experiences; however, recall bias and reverse causality cannot be excluded for retrospective self-reports (e.g., knowing someone with an AEFI or VPD). The “prefer not to answer” option may have increased acceptability but could reduce the interpretability of subgroup analyses or signal disengagement associated with vaccine attitudes.

A limitation of the modelling strategy is that mutually adjusted associations do not imply causality, as determinants were included without a specified causal ordering. Alternative modelling assumptions could show different estimates. This approach nonetheless allowed comprehensive identification of subgroups with higher adjusted likelihood of VH. Additionally, although the aVHS cut-off was supported by prior validation^8^ and complemented by continuous-score analyses, no independent gold standard was available for further calibration. While several components used validated instruments (e.g., aVHS, VCBS), the questionnaire was not validated as a single psychometric tool. Last, the absence of qualitative interviews limited deeper exploration of some associations.

Despite these limitations, this study draws on one of the largest samples used to investigate VH, with quotas ensuring demographic alignment with the Italian adult population and validated instruments assessing hesitancy and its subdimensions. Although conducted in Italy, the study addresses issues common to many high-income countries, e.g., institutional mistrust, political polarisation, and subgroup-specific hesitancy, making the results relevant to international public health planning.

In conclusion, this study identified groups most at risk of VH, with implications for public health strategies. Reaching these groups may require moving beyond traditional settings. Communication should be tailored to specific subgroups by addressing complacency and mistrust using trusted messengers. Local HCWs involvement should be central. Teachers and religious leaders can also be engaged as community amplifiers. In culturally diverse or alternative health-oriented groups, communication may be more effective if delivered by trusted, culturally sensitive intermediaries. Strengthening accessibility and quality of services, while rebuilding institutional trust, remains a priority, as does emphasising the vaccination importance to address complacency. The need for more granular data (e.g., on gender, sexual identity, and ethnicity) to inform truly inclusive strategies emerged.

Several areas warrant investigation. The role of key community figures deserves exploration, especially within minority communities. Political influence on VH remains complex, and the potential of coordinated messaging to mitigate polarisation should be examined. Future research should track hesitancy over time and evaluate targeted interventions designed for diverse and marginalised groups, supported by qualitative studies to clarify underlying motivations.

## Contributors

Conceptualization: FB, MGC, CDV, AO, WR, RS; Methodology: GLM, FB, GEC, MGC, GC, CDV, MM, AM, AO, PR, GPV, WR, RS; Formal analysis: GLM, FB; Data curation: GLM, FB, PR, MM; Writing—Original Draft: GLM, FB, PR; Writing—Review & Editing: GLM, FB, GEC, MGC, GC, CDV, MM, AM, AO, GPV, WR, RS; Visualization: GLM; Supervision: FB, RS; Project Administration: GLM, FB, RS; Funding acquisition: RS. GLM and FB have accessed and verified the data. All authors had final responsibility for the decision to submit for publication.

## Data sharing statement

According to the protocol approved by the Ethics Committee, individual participant data cannot be shared with third parties. The data dictionary and derived data may be made available upon reasonable request to the corresponding author, subject to approval by the study investigators. Requests should be sent to the corresponding author at fabrizio.bert@unito.it.

## Declaration of interests

All authors have completed the ICMJE uniform disclosure form at www.icmje.org/disclosure-of-interest/ and declare support from NextGenerationEU funding within the MUR (Italian Ministry of University and Research) PNRR Extended Partnership initiative on Emerging Infectious Diseases (Project no. PE00000007, INF-ACT) for the submitted work. The authors report the following relationships with private companies and industries in the past 36 months: GLM provided consultancy for Moderna; FB provided consultancy for Sanofi; GEC provided consultancy for GSK, lectures and presentations for GSK, MSD, and Pfizer, and served on an advisory board for GSK and MSD; MM reports accommodation and travel support from GSK; WR provided consultancy for Atheneum, lectures and presentations for Triumph Italy, GSK, the European House-Ambrosetti, Sanofi, I&C srl, Aristea International srl, Italia Longeva, Value Relations srl, and served on an advisory board for Sanofi, MSD, Medistrava the European House-Ambrosetti, and Dedalus; RS received research grants and donations from Pfizer, GSK, Seqirus, Moderna, provided lectures and presentations for GSK and Pfizer, and served on an advisory board for GSK, MSD, Moderna, Pfizer, Sanofi, Novavax, Seqirus, and Astrazeneca. In addition, GEC is scientific director of VIHTALI (spin off of Università Cattolica del Sacro Cuore, Rome, Italy), WR is scientific consultant for VIHTALI and served on an advisory board for The Mission Board on vaccination in Europe, RS was President of the Italian Society of Hygiene, Preventive Medicine and Public Health. No other relationships with industry or activities that could appear to have influenced the submitted work are reported. MGC reports being President of the Società Mediterranea di Salute Mentale, an Honorary Member of the Brazilian Academy of Medicine, and having led projects funded by the World Health Organization, the European Union (DG DEVCO), the Italian Ministry of University and Research, and the Italian Decentralized Cooperation.

## Appendix A Supplementary data

Supplementary File

Translated Abstract
