# Supplementary material for: Prevalence of vaccine hesitancy in Italy: a cross-sectional study
Source: Lancet Reg Health Eur. 2026 Jan 31;63:101603. doi: 10.1016/j.lanepe.2026.101603 (PMC12882703; doi:10.1016/j.lanepe.2026.101603)
Supplement: Supplementary File [file mmc1.docx]

**SUPPLEMENTARY FILE:**

**Prevalence of vaccine hesitancy in Italy: a cross-sectional study**

**Authors**

Giuseppina Lo Moro°^1^, Fabrizio Bert*°^1^, Giovanna Elisa Calabrò^2^, Mauro Giovanni Carta^3^, Giulia Cossu^3^, Corrado De Vito^4^, Manuela Martella^1^, Azzurra Massimi^4^, Anna Odone^5,6^, Paolo Ragusa^1^, Giacomo Pietro Vigezzi^5^, Walter Ricciardi^7^, Roberta Siliquini^1,8^

° Joint first authorship.

**Affiliations**

^1^ Department of Public Health and Pediatric Sciences, University of Turin, Turin, Italy

^2^ Department of Human Sciences, Society and Health, University of Cassino and Southern Lazio, Cassino, Italy

^3^ Department of Medical Sciences and Public Health, University of Cagliari, Cagliari, Italy

^4^ Department of Public Health and Infectious Diseases, Sapienza University of Rome, Rome, Italy

^5^ Department of Public Health, Experimental and Forensic Medicine. University of Pavia, Pavia, Italy

^6^ Medical Direction, Fondazione IRCCS Policlinico San Matteo, Pavia, Italy

^7^ Section of Hygiene, Department of Life Sciences and Public Health, Università Cattolica del Sacro Cuore, Rome, Italy

^8^ AOU City of Health and Sciences, Turin, Italy.

***Corresponding author: Fabrizio Bert**

Department of Public Health and Pediatric Sciences, University of Turin, Turin, Italy

Via Santena 5 bis, 10126, Turin, Italy

Email: [fabrizio.bert@unito.it](mailto:fabrizio.bert@unito.it)

Table of contents

[Supplementary Methods 4](#_Toc213693604)

[M1. Study design and participants 4](#_Toc213693605)

[M2. Procedures: Outcomes 5](#_Toc213693606)

[M3. Procedures: Independent variables 5](#_Toc213693607)

[M4. Statistical analysis 8](#_Toc213693608)

[M5. Regression diagnostics 9](#_Toc213693609)

[M6. References Supplementary Methods 22](#_Toc213693610)

[Supplementary Tables 26](#_Toc213693611)

[Table S1. Unweighted analysis: Sociodemographic, socioeconomic, health-related characteristics and personal experience: analyses with Vaccine Hesitancy as outcome. 26](#_Toc213693612)

[Table S2. Unweighted analysis: Information sources and trust, external influences, beliefs, attitudes and survey mode: analyses with Vaccine Hesitancy as outcome. 28](#_Toc213693613)

[Table S3. Comparison of unweighted and post-stratification weighted prevalence of vaccine hesitancy by study variables (percentage difference). 30](#_Toc213693614)

[Table S4. Unweighted analysis: Hierarchical logistic regression model for vaccine hesitancy 33](#_Toc213693615)

[Table S5. Unweighted analysis: Hierarchical logistic regression model for vaccine hesitancy: model fit statistics by block 38](#_Toc213693616)

[Table S6. Comparison of unweighted and post-stratification weighted estimates in the final multivariable model for vaccine hesitancy (Blocks 1–6). 39](#_Toc213693617)

[Table S7. Post-stratification weighted analysis: Predicted probabilities of vaccine hesitancy, calculated after the multiple logistic regression model with Blocks from 1 to 6 42](#_Toc213693618)

[Table S8. Unweighted analysis: Predicted probabilities of vaccine hesitancy, calculated after the multiple logistic regression model with Blocks from 1 to 6 45](#_Toc213693619)

[Table S9. Post-stratification weighted analysis: Pairwise comparisons of predicted probabilities of vaccine hesitancy, calculated after the multiple logistic regression model with Blocks from 1 to 6 (Bonferroni correction) 48](#_Toc213693620)

[Table S10. Unweighted analysis: Pairwise comparisons of predicted probabilities of vaccine hesitancy, calculated after the multiple logistic regression model with Blocks from 1 to 6 (Bonferroni correction) 54](#_Toc213693621)

[Table S11. Post-stratification weighted analysis: Hierarchical linear regression model for “Lack of trust” subscale 60](#_Toc213693622)

[Table S12. Post-stratification weighted analysis: Hierarchical linear regression model for “Risk perception” subscale 63](#_Toc213693623)

[Table S13. Unweighted analysis: Hierarchical linear regression model for “Lack of trust” subscale 66](#_Toc213693624)

[Table S14. Unweighted analysis: Hierarchical linear regression model for “Risk perception” subscale 69](#_Toc213693625)

[Table S15. Comparison of unweighted and post-stratification weighted estimates in the multivariable models for the secondary outcomes (“Lack of trust” and “Risk perception” subscales) 72](#_Toc213693626)

[Table S16. Post-stratification weighted analysis: Hierarchical linear regression model for vaccine hesitancy (Block 7, adjusted for Vaccine Conspiracy Belief Scale) 77](#_Toc213693627)

[Table S17. Comparison of unweighted and post-stratification weighted estimates logistic regression model for vaccine hesitancy adjusted for Vaccine Conspiracy Belief Scale (Block 7) 80](#_Toc213693628)

[Table S18. Post-stratification weighted analysis: Sensitivity analysis: linear regression models with adult Vaccine Hesitancy Scale (aVHS) score as outcome. 83](#_Toc213693629)

[Table S19. Unweighted analysis: Sensitivity analysis: linear regression models with adult Vaccine Hesitancy Scale (aVHS) score as outcome. 86](#_Toc213693630)

[Table S20. Comparison of unweighted and post-stratification weighted estimates linear regression model for adult Vaccine Hesitancy Scale (aVHS) score. 89](#_Toc213693631)

# Supplementary Methods

## M1. Study design and participants

The survey was conducted in Italy between September 2024 and March 2025, with data collection managed by a professional polling agency using a non-probability, quota-based sampling strategy. Computer-Assisted Web Interviewing (CAWI) was the primary method, supplemented by Computer-Assisted Telephone Interviewing (CATI) to ensure quota completion.

Quotas were established to reflect the adult Italian population by age group, gender, geographic area, and municipality size, based on data from the Italian National Institute of Statistics (ISTAT).^1^ Quotas for educational attainment were estimated using the agency’s proprietary sociocultural profiling system, based on a cumulative database of over 250,000 cases. To enhance inclusiveness, a minimum quota of 1% was set for participants identifying as non-binary or other gender identities, in addition to quotas based on binary gender from ISTAT data.

To estimate the prevalence of Vaccine Hesitancy (VH) with a 1% margin of error and 95% confidence level, a minimum sample of approximately 10,000 adults was required for the Italian population (49,786,127 residents aged ≥18 on the 1^st^ of January 2023, ISTAT^1^), based on Raosoft calculations (http://www.raosoft.com/samplesize.html). We targeted a larger sample (~50,000) to enable robust subgroup and multivariable analyses, ensure adequate representation of minority groups, and improve the precision of estimates.

Differences between CAWI and CATI respondents were expected and consistent with the recruitment method, as CATI interviews were specifically used to reach individuals with limited internet access and to refine quota coverage. A comparison of the two subsamples by key sociodemographic characteristics is reported in the following Table M1.

Table M1. Main sociodemographic characteristics stratified by survey mode.

|  | **CATI** | **CAWI** | p |
| --- | --- | --- | --- |
| **Age group** |  |  | <0·0001 |
| 18–29 | 771 (10·36%) | 6672 (89·64%) |  |
| 30–44 | 923 (8·62%) | 9790 (91·38%) |  |
| 45–59 | 2405 (16·1%) | 12532 (83·9%) |  |
| 60–74 | 5045 (44·8%) | 6217 (55·2%) |  |
| 75+ | 6306 (81·48%) | 1433 (18·52%) |  |
| **Gender** |  |  | <0·0001 |
| Male | 7881 (31·34%) | 17264 (68·66%) |  |
| Female | 7408 (28·03%) | 19020 (71·97%) |  |
| Non-binary/Other | 157 (32·64%) | 324 (67·36%) |  |
| Prefer not to answer | 4 (10%) | 36 (90%) |  |
| **Geographic macro-area** |  |  | <0·0001 |
| North-West | 3410 (24·23%) | 10663 (75·77%) |  |
| North-East | 3642 (35·54%) | 6607 (64·46%) |  |
| Centre | 3718 (35·74%) | 6686 (64·26%) |  |
| South | 3115 (26·49%) | 8646 (73·51%) |  |
| Islands | 1565 (27·91%) | 4042 (72·09%) |  |
| **Marital status** |  |  | <0·0001 |
| Single | 1337 (11·22%) | 10583 (88·78%) |  |
| Married | 10616 (38·27%) | 17125 (61·73%) |  |
| Separated/Divorced | 852 (23·5%) | 2773 (76·5%) |  |
| Cohabiting | 809 (12·82%) | 5499 (87·18%) |  |
| Widowed | 1836 (73·44%) | 664 (26·56%) |  |
| **Children** |  |  | <0·0001 |
| Yes | 12856 (38·53%) | 20511 (61·47%) |  |
| No | 2594 (13·85%) | 16133 (86·15%) |  |
| **Education level** |  |  | <0·0001 |
| Upper secondary | 7477 (27·63%) | 19584 (72·37%) |  |
| Primary/None | 1081 (74·14%) | 377 (25·86%) |  |
| Lower secondary | 4050 (51·59%) | 3800 (48·41%) |  |
| University | 2545 (20·35%) | 9963 (79·65%) |  |
| Postgraduate | 297 (9·23%) | 2920 (90·77%) |  |
| **Continent of citizenship** |  |  | <0·0001 |
| Italy | 15222 (29·79%) | 35871 (70·21%) |  |
| Europe (non-Italy) | 146 (24·7%) | 445 (75·3%) |  |
| Africa | 36 (25·53%) | 105 (74·47%) |  |
| America | 25 (16·67%) | 125 (83·33%) |  |
| Asia | 20 (18·87%) | 86 (81·13%) |  |
| Oceania | 1 (100%) | 0 (0%) |  |

p-value obtained by Chi-squared test.

Abbreviations: CATI Computer Assisted Telephone Interviewing, CAWI Computer Assisted Web Interviewing

## M2. Procedures: Outcomes

The primary outcome was VH, measured using the Italian validated version^2^ of the adult Vaccine Hesitancy Scale (aVHS).^3^ We used the validated Italian version of the adult Vaccine Hesitancy Scale (aVHS), ^2^ which underwent full cross-cultural adaptation and psychometric testing: the Italian version demonstrated excellent internal consistency (Cronbach’s α=0·94), test–retest reliability (intra-class correlation coefficient=0·87), and content validity (scale content of validity index=0·97). ^2^ The aVHS consists of 10 items rated on a 5-point Likert scale (from “strongly disagree” to “strongly agree”), with each item scored from 1 to 5. Items 1–4 and 6–8 were reverse-coded prior to summing, resulting in a total score ranging from 10 to 50, with higher scores indicating greater hesitancy.^3^ In our sample, the aVHS internal consistency was high (Cronbach’s α=0.894).

Participants scoring 25 or above were classified as vaccine-hesitant, in line with the original validation study^3^ and subsequent research using the aVHS.^4–6^ This binary classification was used as the primary outcome.

As secondary outcomes, we used the aVHS subscales: “Lack of trust” dimension (items 1-4, 6-8; score range 7-35) and “Risk perception” dimension (items 5, 9, 10; score range 3-15), based on prior literature.^7,8^ A principal component analysis conducted with SPSS (Version 29.0.1.0) confirmed the two-component structure in our sample (Kaiser-Meyer-Olkin test=0.930; Bartlett’s test p<0.001), explaining 75.7% of the total variance. The “Lack of trust” and the “Risk perception” subscales had an internal consistency of 0.955 and 0.764, respectively.

## M3. Procedures: Independent variables

To characterise vaccine-hesitant adults in Italy, we included a comprehensive set of variables, selected on the basis of previous literature. For analytical clarity, the variables are presented below according to the conceptual blocks used in the analysis. The variables given in Table 1 and 2 (Main paper) reflect the original response options from the questionnaire, unless otherwise specified below. “Prefer not to answer” options were included for gender, sexual orientation, political orientation, and religion, following consultation with the Ethics Committee, as these variables were considered sensitive. Other sections of the questionnaire were designed to address secondary objectives of the survey and will be analysed separately in future publications.

*Sociodemographic and socioeconomic characteristics*

Basic characteristics, such as age, gender, marital status, and parenthood, have shown inconsistent associations with VH in the literature, suggesting the need for further investigation.^9–11^ About gender, evidence highlighted the importance of going beyond the binary categorisation, as non-binary and gender-diverse individuals may have distinct vaccination attitudes.^12^

As for measurements, participants were asked to report their age in years. Age was then categorised following a grouping used in an Italian report of the European Social Survey, ^13^ which also reflects age ranges relevant to the national vaccination programme.^14^ Gender and marital status were assessed using predefined response options. Parenthood was evaluated by a multiple-choice question on the presence of children in specific age groups (≤11, 12-18, >18 years). Responses were recoded into a mutually exclusive five-category variable.

The place of residence was considered relevant given documented structural disadvantages in Southern Italy and the Islands ^15^ and evidence from other contexts suggesting that geographic barriers, such as distance from urban health hubs, may influence vaccinations.^16,17^ Thus, residence was collected at the municipal level and used to derive three geographical variables: municipality size, classified by population size into six categories; ^18^ geographical macro-area;^18^ and internal area status (degree of urbanization), derived using the Italian “Aree Interne” classification (2021–2027), which identifies municipalities based on distance from essential services.^19^

Educational level and occupational status were also included, given the frequently reported association between lower education and higher VH, as well as the lower VH observed in certain professional categories such as healthcare workers (HCWs).^9,10,20^ Participants reported their highest level of education completed and occupation was recorded via a predefined list.

We also assessed socioeconomic status, as it has been frequently linked to higher VH, though findings are inconsistent across contexts.^21,22^ Socioeconomic vulnerability was assessed using a battery of nine items reflecting material deprivation. Following Eurostat guidelines,^23^ a binary indicator of severe material deprivation was created, identifying individuals reporting at least four of the nine conditions.

We collected data on nationality, ethnic group, and sexual orientation to capture potential inequalities affecting minority groups, as most studies on minority populations have been conducted outside of Europe.^24,25^ Exclusion of ethnic and LGBTQ+ communities from health research has been linked to mistrust, misinformation, and increased hesitancy.^26–28^ Nationality was used to derive a variable indicating continent of citizenship,^29^ with Italian nationals kept as a distinct category. Participants self-identified their ethnic group(s) from a predefined list, with the option to select multiple responses. Those reporting more than one group were classified as “multi-ethnic,” and all other categories were retained individually. Sexual orientation was assessed with multiple response options.

*Health-related characteristics and personal experience*

The presence of chronic conditions has been associated with lower VH.^30,31^ Participants were asked whether they suffered from one or more chronic conditions through a multiple-choice item listing a range of diseases categories. Responses were recoded into a three-level variable: no chronic condition, one chronic condition, or more than one. Living arrangements were also assessed^9^: participants were asked whether they lived with individuals with disabilities or chronic illnesses, as we hypothesised these conditions may affect attitudes toward vaccination.

Health literacy (HL) was included: although higher HL is expected to support vaccine uptake through improved informed decision-making, the relationship between HL and vaccination remains unclear.^32^ HL was assessed using the Single Item Literacy Screener (SILS), a tool developed to identify individuals who may have difficulty understanding written health information.^33,34^ Participants were asked how often they needed help reading materials such as instructions, pamphlets, or other written content from a doctor or pharmacy. Response options ranged from “Never” to “Always” on a five-point scale. Scores above 2 were classified as indicating inadequate HL.^34^

We assessed participants’ experiences with vaccine-preventable diseases (VPD) and adverse events following immunisation (AEFI), which may influence risk perception and attitudes toward vaccines.^35,36^ Participants were asked if they personally knew someone who had experienced AEFI, and whether they knew anyone who had developed VPD due to a lack of vaccination.

We investigated whether participants had encountered specific barriers when trying to access vaccination services, as such past experiences may contribute to hesitancy by reinforcing negative perceptions of the healthcare system or the vaccination process itself.^37^ Perceived barriers to vaccination were assessed through a multiple-choice question listing various barriers. A TwoStep Cluster analysis (SPSS, Version 29.0.1.0) was conducted to identify participant profiles based on reported barriers. The algorithm, guided by Schwarz’s Bayesian Criterion, identified only two clusters: those reporting no relevant barriers and those reporting at least one.

*Information sources*

We included variables on information sources and trust: prior studies among parents found that relying on the internet was associated with higher refusal rates,^38^ while those consulting HCWs showed lower hesitancy.^39^ Participants indicated up to three main sources they use for health and medical information, selecting from a predefined list of 13 sources. The list of possible sources included: general practitioners; medical specialists; other health professionals; institutional websites (e.g., Ministry of Health, Civil Protection, World Health Organization, scientific societies); printed or online newspapers and magazines; radio and/or television; non-institutional websites (e.g., Google, Wikipedia, blogs, and forums); institutional social media pages (e.g., Facebook, Instagram, X, TikTok, Telegram); non-institutional social media pages; religious leaders; political leaders; celebrities/influencers; and family members or acquaintances. As above, a TwoStep Cluster analysis identified two distinct profiles. In cluster 1 (diversified sources), participants used a wide range of sources, including media, websites, social networks, and HCWs. Some sources (e.g. printed media, TV, and social networks) were used exclusively by this group. In cluster 2 (professional-only sources), participants relied exclusively on general practitioners, specialists, and other health professionals, without reporting use of other media or online sources. Trust in the main information source was measured on a four-point Likert scale (1=not at all, 4=very much) and treated as a discrete quantitative variable.

*External influences*

We explored contextual influences^40^ by assessing participants’ perceptions of the vaccination stance of key community figures. Participants were asked whether, to their knowledge, key figures in their community (religious leaders, political figures, teachers, and HCWs) were supportive of vaccination (possible answers: “Yes”, “No”, “Don’t know”).

*Beliefs and attitudes*

Use of complementary and alternative medicine (CAM) was assessed, as it may represent an attitude that has been associated with VH and greater distrust in conventional medicine.^41^ Participants were asked about their engagement with CAM through a multiple-choice question.

Political orientation was included given the potential impact of political ideology, as right-wing and populist positions have been associated with higher VH.^42,43^ Political orientation was assessed using an 11-point scale from 0 (far left) to 10 (far right), with additional options for “Non-aligned with traditional parties” and “Prefer not to answer.” Following recommendations for enriched scales,^44^ responses were recoded into seven categories: extreme left (0), left (1–3), centre (4–6), right (7–9), extreme right (10), non-aligned, and “Prefer not to answer”.

Religion was explored, as various faiths have been associated with vaccination refusal due to concerns over vaccine composition, safety, or interference with natural processes.^40,45,46^ Religious affiliation was reported by selecting from a list of options. The importance of religion in daily life was assessed on a 0–10 scale and recoded into six categories similarly to politics: not at all (0), slightly (1–3), moderately (4–6), very (7–9), extremely (10) important, and “Prefer not to answer”.

We also evaluated perceived quality and accessibility of the Italian National Health Service (NHS), since trust in healthcare institutions may influence VH.^10,40^ Participants rated the perceived quality and accessibility on 11-point scales (0=worst/least accessible, 10=best/most accessible). Both were treated as discrete quantitative variables.

*Vaccine conspiracy beliefs*

Given the association between conspiracy beliefs and VH,^47^ and the specific role of conspiratorial thinking about vaccinations in undermining vaccine intention,^48,49^ we hypothesised that such beliefs may contribute to VH and account for part of its variance. We used the Vaccine Conspiracy Beliefs Scale (VCBS), a validated 7-item instrument.^50^ Each item was rated on a 7-point Likert scale ranging from 1 (“strongly disagree”) to 7 (“strongly agree”). A higher average score reflects a stronger endorsement of vaccine conspiracy beliefs. The internal consistency of the VCBS in our sample was high (α=0·968).

## M4. Statistical analysis

Descriptive analyses were executed for all variables. Given the large sample size, quantitative variables were treated as normally distributed.^51^ The primary outcome (VH) prevalence was described with the corresponding 95% confidence interval (CI). Prevalence of VH was reported across all independent variables, and chi-squared tests (or t-tests for quantitative variables) were used to examine associations. Univariable logistic regression models were also calculated to estimate crude odds ratios (ORs).

To adjust for discrepancies between the sample and the target population, we applied post-stratification weighting using external population benchmarks provided by the Italian National Institute of Statistics (ISTAT). Specifically, we constructed post-strata based on the joint distribution of age group (18–29, 30–44, 45–59, 60–74, and 75+), geographical macro-area (North-West, North-East, Centre, South, and Islands), and municipality size (inhabitants: ≤10,000, 10,001–25,000, 25,001–50,000, 50,001–100,000, 100,001–250,000, and >250,000. Population totals for each post-stratum were retrieved from ISTAT’s official demographic estimates as of January 1st, 2025.^52^ Post-stratification was implemented in Stata using the svyset command with the poststrata() and postweight() options.

We opted not to include gender or educational attainment in the post-stratification scheme. Gender was excluded because of incomplete harmonization with population benchmarks: ISTAT provides only binary gender categories, while our survey included additional gender identities. Educational attainment was excluded due to the lack of recent, disaggregated and harmonized data from ISTAT corresponding to our survey categories. Additionally, education quotas in the original sampling frame were derived from proprietary estimates based on a large cumulative dataset, which cannot be directly reconciled with public benchmarks.

A multivariable logistic regression model was developed using a hierarchical approach in six blocks: (1) sociodemographic and socioeconomic characteristics, (2) health-related characteristics and personal experiences, (3) information sources, (4) external influences, (5) beliefs and attitudes, and (6) survey mode. Collinearity was assessed using the Variance Inflation Factor (VIF); the mean VIF was 1.67, with all values less than 5 (Supplementary Methods M5.1). Model fit was evaluated using the log-likelihood, likelihood ratio chi-square statistics, degrees of freedom, and pseudo R². Likelihood ratio tests were used to compare adjacent models.

The final model showed good performance, supported by multiple diagnostics, including influential observation analysis (Pregibon’s delta-beta), discrimination, calibration, and bootstrap internal validation. We evaluated the influence of individual observations on the final model estimates using Pregibon’s delta-beta (Δβ). Because no universal cutoff for Δβ exists, we visually inspected scatterplots of Δβ against predicted probabilities to identify extreme points (Supplementary Methods M5.2). Then, we refitted the model: excluding high-influence observations (Δβ > 0.20) did not materially change the direction or magnitude of the associations: estimates from the restricted model closely matched the full-sample model (Supplementary Methods M5.3), supporting the robustness of results. Discrimination was assessed using the area under the ROC curve (AUC=0.812). Calibration was evaluated by comparing observed and predicted probabilities across deciles of predicted risk (calibration plot) and using the Hosmer–Lemeshow goodness-of-fit test. Calibration was strong, with close agreement between observed and predicted probabilities throughout deciles of risk (Supplementary Methods M5.4). Although the Hosmer–Lemeshow test was statistically significant (p<0·001), this was expected given the large sample size (n>50,000) and was not supported by visual evidence of miscalibration.

Although the primary purpose of the multivariable model was to estimate adjusted associations between determinants and VH, internal validation was performed using bootstrap resampling (50 replications), estimating optimism-corrected discrimination (C-Statistic), calibration (calibration-in-the-large, expected:observed ratio slope), and overall accuracy (scaled Brier score), along with heuristic and bootstrap shrinkage factors (Stata user-developed bsvalidation command ^53^). Internal validation through bootstrap resampling (50 replications) showed excellent model performance with minimal overfitting (optimism-adjusted C-statistic: 0.811; calibration slope: 0.992). Calibration was accurate (E:O ratio: 1.00; CITL: 0.002), confirming that predicted probabilities closely matched observed outcomes. Shrinkage factors were near 1 (0.994 heuristic; 0.992 bootstrap), indicating negligible overfitting and no need for substantial penalization of regression coefficients (Supplementary Methods M5.5).

Post-stratification weights were applied to the final model.

Predicted probabilities of VH were computed from the final model using predictive margins. These were visualised graphically to aid interpretation. Predicted probabilities were estimated as model-adjusted, population-averaged risks. According to the Stata’s margins command, the predictive margins are the weighted average of the predicted probabilities for each observation in the estimation sample. Cell counts and 95% CI were provided for all predicted probability estimates.

For categorical variables with more than two levels, pairwise comparisons of predicted probabilities were performed: pairwise contrasts quantified absolute differences between categories, with p-values adjusted using the Bonferroni correction.

Indeed, to compare differences between categories within each factor-variable term, we performed post-estimation pairwise contrasts of adjusted predictions using the margins command with the pwcompare(effects) option. To account for multiple comparisons within each factor, p-values and confidence intervals were adjusted using Bonferroni’s correction, as implemented by Stata’s mcompare(bonferroni) option. This procedure adjusts the comparisonwise error rate (αc) to ensure that the experimentwise error rate (αe) does not exceed the prespecified threshold, according to the Bonferroni inequality (αe ≤ mαc), where m represents the number of pairwise comparisons for a given k-level factor (m = k(k−1)/2). For binary independent variables, no multiple-comparison correction was required, as only one contrast exists (m=1).

Post-stratification weights were applied also to predicted probabilities and pairwise contrasts.

For secondary outcomes (aVHS subscales), separate univariable and multivariable linear regression models were performed. Multivariable models, including all the above-mentioned blocks (blocks 1-6), were estimated both with and without VCBS. In linear regressions partial eta-squared (η²) was calculated as a measure of effect size. Assumptions for linear regression were evaluated, including linearity, residual normality, and homoscedasticity. Minor deviations from normality were observed, while heteroskedasticity was detected and addressed by using robust standard errors.

Linearity of discrete predictors modelled as continuous variables was assessed using added-variable (partial regression) plots (avplot). Visual inspection of added-variable plots showed no appreciable departures from linearity for the discrete predictors (Supplementary Methods M5.6-M5.13). Residual normality was assessed using kernel density estimation and normal Q-Q plots (Supplementary Methods M5.18-M5.21). Residuals showed an approximately normal distribution, with only minor deviations, unlikely to affect model inference. The assumption of constant residual variance was evaluated using the Breusch–Pagan/Cook–Weisberg test. Evidence of heteroskedasticity was found across the linear outcomes (Breusch–Pagan/Cook–Weisberg tests, all p<0·001). Thus, all unweighted linear models were fitted using robust standard errors (vce(robust) option in Stata). Weighted models were fitted using Stata’s svy commands, which apply Taylor-series linearisation to provide robust variance estimation.

Post-stratification weights were applied to the aVHS subscales multivariable regression models.

Two sensitivity analyses were conducted for the primary outcome. First, a seventh block including the VCBS was added to the regression model to assess which groups had a higher probability of VH independently of conspiracy beliefs, treating these beliefs as one component of hesitancy. Second, the aVHS score was analysed as a continuous outcome using linear regression models (with robust standard errors), with and without the inclusion of VCBS, to assess the robustness of our results and to preserve the full informational content of the scale. Linear regression diagnostics were performed using the same procedures applied to the aVHS subscales (Supplementary file M5.14-M5.17, M5.22-M5.23, Breusch–Pagan/Cook–Weisberg tests p<0·001). Post-stratification weights were applied to the multivariable regression models used in the sensitivity analyses.

Results from logistic regressions were expressed as crude or adjusted ORs (ORs/adjORs) with 95% CIs. Results from linear regressions were reported as crude or adjusted coefficients (Coef/adjCoef) with 95% CIs and partial eta-squared (η²) was calculated as a measure of effect size. Partial eta-squared values were interpreted as follows: 0.0099 as small, 0.0588 as medium, and 0.1379 as large effect.^54^

Overall, variables were entered in the regression models by forced entry, and missing data were handled by listwise deletion. Post-stratification weighted results are presented as the main analyses in the text, while complete unweighted analyses are provided in the Supplementary Materials. Weighted and unweighted multivariable regression estimates were compared, with percentage differences between adjusted OR (or coefficients) computed to describe consistency.

All analyses were conducted using Stata (Versions 18 and 19). Figures were created with Excel 2019. A two-sided p-value <0·050 was considered statistically significant.

## M5. Regression diagnostics

**M5.1 Variance Inflation Factor (VIF)**

| **Variable** | **VIF** | **1/VIF** |
| --- | --- | --- |
| **Age group** |  |  |
| 18–29 |  |  |
| 30–44 | 2·58 | 0·39 |
| 45–59 | 3·56 | 0·28 |
| 60–74 | 4·65 | 0·21 |
| 75+ | 4·67 | 0·21 |
| **Gender** |  |  |
| Male |  |  |
| Female | 1·15 | 0·87 |
| Non-binary/Other | 1·12 | 0·90 |
| Prefer not to answer | 1·01 | 0·99 |
| **Marital status** |  |  |
| Single |  |  |
| Married | 2·98 | 0·34 |
| Separated/Divorced | 1·58 | 0·63 |
| Cohabiting | 1·56 | 0·64 |
| Widowed | 1·66 | 0·60 |
| **Children** |  |  |
| No children |  |  |
| Only children ≤11 years | 1·56 | 0·64 |
| Only children 12-18 years | 1·37 | 0·73 |
| Only children >18 years | 3·15 | 0·32 |
| Children of various ages | 1·31 | 0·76 |
| **Sexual orientation** |  |  |
| Heterosexual |  |  |
| Homosexual | 1·03 | 0·97 |
| Bisexual | 1·06 | 0·94 |
| Pansexual | 1·08 | 0·93 |
| Ace spectrum | 1·11 | 0·90 |
| Prefer not to answer | 1·34 | 0·75 |
| **Municipality size (inhabitants)** |  |  |
| ≤10,000 |  |  |
| 10,001–25,000 | 1·43 | 0·70 |
| 25,001–50,000 | 1·6 | 0·62 |
| 50,001–100,000 | 2·35 | 0·43 |
| 100,001–250,000 | 2·5 | 0·40 |
| >250,000 | 3·79 | 0·26 |
| **Geographic macro-area** |  |  |
| North-West |  |  |
| North-East | 1·5 | 0·67 |
| Centre | 1·49 | 0·67 |
| South | 1·62 | 0·62 |
| Islands | 1·39 | 0·72 |
| **Degree of urbanisation** |  |  |
| Pole |  |  |
| Intermunicipal pole | 1·2 | 0·83 |
| Belt | 4·34 | 0·23 |
| Intermediate | 2·97 | 0·34 |
| Peripheral | 2·41 | 0·41 |
| Ultra-peripheral | 1·33 | 0·75 |
| **Education level** |  |  |
| Upper secondary |  |  |
| Primary/None | 1·15 | 0·87 |
| Lower secondary | 1·24 | 0·81 |
| University | 1·21 | 0·83 |
| Postgraduate | 1·13 | 0·89 |
| **Occupational status** |  |  |
| Non-healthcare worker |  |  |
| Healthcare worker | 1·09 | 0·92 |
| Homemaker | 1·27 | 0·79 |
| Retired | 3·2 | 0·31 |
| Student (non-health field) | 1·38 | 0·72 |
| Student (health field) | 1·13 | 0·88 |
| Job seeker | 1·12 | 0·90 |
| Unemployed | 1·13 | 0·89 |
| Other | 1·01 | 0·99 |
| **Continent of citizenship** |  |  |
| Italy |  |  |
| Europe (non-Italy) | 1·09 | 0·92 |
| Africa | 1·32 | 0·76 |
| America | 1·34 | 0·75 |
| Asia | 1·51 | 0·66 |
| Oceania | 1·01 | 0·99 |
| **Self-identified ethnicity** |  |  |
| European |  |  |
| Multi-ethnic | 1·08 | 0·93 |
| North American / Australian | 1·05 | 0·96 |
| Arab-Middle Eastern | 1·14 | 0·88 |
| North African | 1·24 | 0·81 |
| Latino-American | 1·36 | 0·74 |
| African American | 1·02 | 0·98 |
| Black African | 1·18 | 0·85 |
| Asian | 1·52 | 0·66 |
| Pacific Islands | 1·01 | 0·99 |
| **Material deprivation** |  |  |
| No deprivation |  |  |
| Severe deprivation | 1·06 | 0·94 |
| **Chronic conditions** |  |  |
| No chronic disease |  |  |
| One chronic disease | 1·22 | 0·82 |
| More than one chronic disease | 1·41 | 0·71 |
| **Living with a person with disability** |  |  |
| No |  |  |
| Yes | 1·1 | 0·91 |
| **Inadequate health literacy** |  |  |
| No |  |  |
| Yes | 1·13 | 0·89 |
| **Knowing someone who had AEFI** |  |  |
| No |  |  |
| Yes | 1·21 | 0·83 |
| **Knowing someone who had VPD** |  |  |
| No |  |  |
| Yes | 1·17 | 0·85 |
| **Reported barriers to vaccination** |  |  |
| No |  |  |
| Yes | 1·24 | 0·81 |
| **Information source cluster** |  |  |
| Diversified sources |  |  |
| Professional-only sources | 1·18 | 0·85 |
| **Trust in sources** | 1·28 | 0·78 |
| **By religious leaders** |  |  |
| Yes |  |  |
| No | 2·07 | 0·48 |
| Don’t know | 2·17 | 0·46 |
| **By political leaders** |  |  |
| Yes |  |  |
| No | 1·95 | 0·51 |
| Don’t know | 2·26 | 0·44 |
| **By teachers** |  |  |
| Yes |  |  |
| No | 1·92 | 0·52 |
| Don’t know | 2·16 | 0·46 |
| **By health professionals** |  |  |
| Yes |  |  |
| No | 1·67 | 0·60 |
| Don’t know | 1·82 | 0·55 |
| **Use of non-conventional medicine** |  |  |
| No |  |  |
| Yes, integrated with conventional medicine | 1·18 | 0·85 |
| Yes, as alternative to conventional medicine | 1·13 | 0·88 |
| **Political orientation** |  |  |
| Right (7–9) |  |  |
| Centre (4–6) | 1·78 | 0·56 |
| Extreme left (0) | 1·25 | 0·80 |
| Left (1–3) | 1·56 | 0·64 |
| Extreme right (10) | 1·18 | 0·85 |
| Non-aligned with traditional parties | 1·7 | 0·59 |
| Prefer not to answer | 1·55 | 0·64 |
| **Religion** |  |  |
| Catholic |  |  |
| Orthodox | 1·25 | 0·80 |
| Protestant | 1·04 | 0·96 |
| Jewish | 1·05 | 0·95 |
| Muslim | 1·36 | 0·73 |
| Jehovah’s Witness | 1·06 | 0·94 |
| Atheist | 1·76 | 0·57 |
| Agnostic | 1·19 | 0·84 |
| Buddhist | 1·03 | 0·97 |
| Hindu | 1·02 | 0·98 |
| Other | 1·05 | 0·95 |
| Prefer not to answer | 1·26 | 0·80 |
| **Importance of religion** |  |  |
| Not at all (0) |  |  |
| Slightly (1–3) | 2·18 | 0·46 |
| Somewhat important (4–6) | 3·6 | 0·28 |
| Very (7–9) | 4·15 | 0·24 |
| Extremely (10) | 2·15 | 0·46 |
| Prefer not to answer | 1·82 | 0·55 |
| **Perceived NHS quality** | 2·12 | 0·47 |
| **Perceived NHS access** | 2·06 | 0·49 |
| **Survey mode** |  |  |
| CAWI |  |  |
| CATI | 2·03 | 0·49 |

Abbreviations: AEFI Adverse Event Following Immunisation, CATI Computer-Assisted Telephone Interviewing, CAWI Computer-Assisted Web Interviewing, NHS National Health Service, VPD Vaccine Preventable Disease.

**M5.2 Pregibon’s delta-beta: scatterplot**


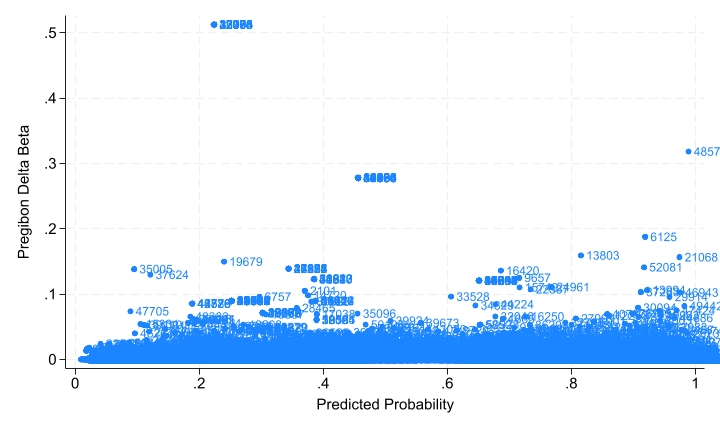


**M5.3 Vaccine hesitancy final logistic regression model (blocks 1-6): excluding high-influence observations (Pregibon’s delta-beta > 0.20)**

|  | **Model excluding high-influence observations** | | **Comparison** | | | | |
| --- | --- | --- | --- | --- | --- | --- | --- |
|  |  |  | **Main model** |  | **Model excluding high-influence observations** | |  |
|  | **adjOR (95%CI)** | **p** | **adjOR** | **SE** | **adjOR** | **SE** | **Percentage difference adjOR** |
| **Age group** |  |  |  |  |  |  |  |
| 18–29 | Ref. |  | Ref. |  |  |  |  |
| 30–44 | 1·47 (1·35 to 1·59) | <0·0001 | 0·384*** | -9·19 | 0·384*** | -9·18 | 0·00 |
| 45–59 | 1·57 (1·44 to 1·72) | <0·0001 | 0·458*** | -10·38 | 0·453*** | -10·27 | -1·09 |
| 60–74 | 1·79 (1·6 to 1·99) | <0·0001 | 0·582*** | -10·57 | 0·580*** | -10·53 | -0·34 |
| 75+ | 1·41 (1·25 to 1·6) | <0·0001 | 0·342*** | -5·39 | 0·344*** | -5·41 | 0·58 |
| **Gender** |  |  |  |  |  |  |  |
| Male | Ref. |  | Ref. |  |  |  |  |
| Female | 1·01 (0·97 to 1·06) | 0·627 | 0·013 | -0·58 | 0·011 | -0·49 | -15·38 |
| Non-binary/Other | 2·12 (1·67 to 2·69) | <0·0001 | 0·752*** | -6·17 | 0·751*** | -6·17 | -0·13 |
| Prefer not to answer | 1·02 (0·46 to 2·29) | 0·952 | 0·0258 | -0·06 | 0·0246 | -0·06 | -4·65 |
| **Marital status** |  |  |  |  |  |  |  |
| Single | Ref. |  | Ref. |  |  |  |  |
| Married | 0·9 (0·84 to 0·96) | 0·00301 | -0·106** | (-2·93) | -0·108** | (-2·97) | 1·89 |
| Separated/Divorced | 1·12 (1·01 to 1·24) | 0·034 | 0·111* | -2·14 | 0·110* | -2·13 | -0·90 |
| Cohabiting | 1·03 (0·95 to 1·11) | 0·502 | 0·0346 | -0·86 | 0·027 | -0·67 | -21·97 |
| Widowed | 0·76 (0·67 to 0·86) | <0·0001 | -0·271*** | (-4·28) | -0·271*** | (-4·28) | 0·00 |
| **Children** |  |  |  |  |  |  |  |
| No children | Ref. |  | Ref. |  |  |  |  |
| Only children ≤11 years | 0·94 (0·87 to 1·01) | 0·11 | -0·068 | (-1·69) | -0·0649 | (-1·61) | -4·56 |
| Only children 12-18 years | 0·89 (0·81 to 0·98) | 0·0203 | -0·117* | (-2·39) | -0·114* | (-2·32) | -2·56 |
| Only children >18 years | 0·78 (0·73 to 0·84) | <0·0001 | -0·249*** | (-6·60) | -0·247*** | (-6·52) | -0·80 |
| Children of various ages | 0·76 (0·68 to 0·85) | <0·0001 | -0·276*** | (-5·07) | -0·272*** | (-5·00) | -1·45 |
| **Sexual orientation** |  |  |  |  |  |  |  |
| Heterosexual | Ref. |  | Ref. |  |  |  |  |
| Homosexual | 0·8 (0·67 to 0·94) | 0·0078 | -0·228** | (-2·68) | -0·227** | (-2·66) | -0·44 |
| Bisexual | 0·77 (0·66 to 0·9) | 0·00074 | -0·264*** | (-3·40) | -0·262*** | (-3·37) | -0·76 |
| Pansexual | 0·92 (0·7 to 1·21) | 0·55 | -0·0832 | (-0·59) | -0·0836 | (-0·59) | 0·48 |
| Ace spectrum | 0·89 (0·7 to 1·13) | 0·35 | -0·127 | (-1·03) | -0·116 | (-0·94) | -8·66 |
| Prefer not to answer | 0·65 (0·59 to 0·72) | <0·0001 | -0·432*** | (-8·42) | -0·433*** | (-8·43) | 0·23 |
| **Municipality size (inhabitants)** |  |  |  |  |  |  |  |
| ≤10,000 | Ref. |  | Ref. |  |  |  |  |
| 10,001–25,000 | 0·98 (0·92 to 1·04) | 0·46 | -0·0203 | (-0·66) | -0·0229 | (-0·74) | 12·81 |
| 25,001–50,000 | 0·98 (0·91 to 1·06) | 0·63 | -0·0184 | (-0·48) | -0·0181 | (-0·48) | -1·63 |
| 50,001–100,000 | 1·06 (0·96 to 1·17) | 0·28 | 0·0553 | -1·07 | 0·0557 | -1·08 | 0·72 |
| 100,001–250,000 | 1·09 (0·96 to 1·23) | 0·18 | 0·086 | -1·37 | 0·0842 | -1·34 | -2·09 |
| >250,000 | 0·98 (0·88 to 1·1) | 0·74 | -0·0132 | (-0·23) | -0·019 | (-0·34) | 43·94 |
| **Geographic macro-area** |  |  |  |  |  |  |  |
| North-West | Ref. |  | Ref. |  |  |  |  |
| North-East | 1·02 (0·96 to 1·09) | 0·51 | 0·0208 | -0·64 | 0·0215 | -0·66 | 3·37 |
| Centre | 1 (0·93 to 1·06) | 0·88 | 0·00154 | -0·05 | -0·00483 | (-0·15) | -413·64 |
| South | 0·92 (0·86 to 0·98) | 0·0098 | -0·0832** | (-2·60) | -0·0827** | (-2·58) | -0·60 |
| Islands | 0·86 (0·79 to 0·93) | 0·00015 | -0·154*** | (-3·82) | -0·152*** | (-3·79) | -1·30 |
| **Degree of urbanisation** |  |  |  |  |  |  |  |
| Pole | Ref. |  | Ref. |  |  |  |  |
| Intermunicipal pole | 0·96 (0·83 to 1·11) | 0·56 | -0·0408 | (-0·56) | -0·0425 | (-0·58) | 4·17 |
| Belt | 1·09 (0·99 to 1·19) | 0·0704 | 0·0829 | -1·82 | 0·0824 | -1·81 | -0·60 |
| Intermediate | 1·07 (0·97 to 1·19) | 0·17 | 0·0768 | -1·48 | 0·0716 | -1·38 | -6·77 |
| Peripheral | 0·98 (0·87 to 1·1) | 0·75 | -0·0181 | (-0·30) | -0·0191 | (-0·32) | 5·52 |
| Ultra-peripheral | 0·97 (0·8 to 1·18) | 0·77 | -0·0281 | (-0·28) | -0·0303 | (-0·30) | 7·83 |
| **Education level** |  |  |  |  |  |  |  |
| Upper secondary | Ref. |  | Ref. |  |  |  |  |
| Primary/None | 0·57 (0·5 to 0·66) | <0·0001 | -0·561*** | (-7·71) | -0·559*** | (-7·68) | -0·36 |
| Lower secondary | 1·06 (0·99 to 1·12) | 0·097 | 0·0501 | -1·55 | 0·0537 | -1·66 | 7·19 |
| University | 0·84 (0·8 to 0·89) | <0·0001 | -0·174*** | (-6·38) | -0·171*** | (-6·30) | -1·72 |
| Postgraduate | 0·7 (0·64 to 0·77) | <0·0001 | -0·353*** | (-7·52) | -0·350*** | (-7·46) | -0·85 |
| **Occupational status** |  |  |  |  |  |  |  |
| Non-healthcare worker | Ref. |  | Ref. |  |  |  |  |
| Healthcare worker | 0·71 (0·64 to 0·78) | <0·0001 | -0·346*** | (-6·67) | -0·348*** | (-6·70) | 0·58 |
| Homemaker | 1·09 (1 to 1·19) | 0·049 | 0·0950* | -2·17 | 0·0864* | -1·97 | -9·05 |
| Retired | 0·83 (0·76 to 0·9) | <0·0001 | -0·181*** | (-4·29) | -0·187*** | (-4·43) | 3·31 |
| Student (non-health field) | 0·65 (0·58 to 0·74) | <0·0001 | -0·424*** | (-6·69) | -0·424*** | (-6·70) | 0·00 |
| Student (health field) | 0·54 (0·45 to 0·65) | <0·0001 | -0·618*** | (-6·60) | -0·618*** | (-6·60) | 0·00 |
| Job seeker | 1·02 (0·91 to 1·14) | 0·76 | 0·0188 | -0·32 | 0·018 | -0·3 | -4·26 |
| Unemployed | 1·02 (0·92 to 1·14) | 0·69 | 0·023 | -0·43 | 0·0216 | -0·4 | -6·09 |
| Other | 0·73 (0·43 to 1·25) | 0·25 | -0·316 | (-1·15) | -0·315 | (-1·14) | -0·32 |
| **Continent of citizenship** |  |  |  |  |  |  |  |
| Italy | Ref. |  | Ref. |  |  |  |  |
| Europe (non-Italy) | 1·02 (0·83 to 1·24) | 0·86 | 0·013 | -0·13 | 0·0175 | -0·17 | 34·62 |
| Africa | 1·17 (0·74 to 1·84) | 0·501 | 0·149 | -0·64 | 0·156 | -0·67 | 4·70 |
| America | 0·86 (0·54 to 1·35) | 0·505 | -0·155 | (-0·67) | -0·155 | (-0·67) | 0·00 |
| Asia | 0·95 (0·54 to 1·65) | 0·85 | -0·0535 | (-0·19) | -0·0557 | (-0·20) | 4·11 |
| Oceania | - | - | - | - | - | - | - |
| **Self-identified ethnicity** |  |  |  |  |  |  |  |
| European | Ref. |  | Ref. |  |  |  |  |
| Multi-ethnic | 1·35 (1·04 to 1·76) | 0·027 | 0·302* | -2·23 | 0·300* | -2·21 | -0·66 |
| North American / Australian | 1·44 (0·95 to 2·2) | 0·089 | 0·368 | -1·71 | 0·366 | -1·7 | -0·54 |
| Arab-Middle Eastern | 1·05 (0·73 to 1·5) | 0·79 | 0·0513 | -0·28 | 0·0493 | -0·27 | -3·90 |
| North African | 1·31 (0·92 to 1·87) | 0·14 | 0·271 | -1·49 | 0·271 | -1·49 | 0·00 |
| Latino-American | 0·89 (0·65 to 1·21) | 0·44 | -0·12 | (-0·76) | -0·12 | (-0·77) | 0·00 |
| African American | 1·4 (0·57 to 3·46) | 0·46 | 0·144 | -0·32 | 0·34 | -0·74 | 136·11 |
| Black African | 0·84 (0·5 to 1·41) | 0·52 | -0·169 | (-0·64) | -0·171 | (-0·65) | 1·18 |
| Asian | 0·94 (0·6 to 1·48) | 0·79 | -0·0602 | (-0·26) | -0·0606 | (-0·26) | 0·66 |
| Pacific Islands | 0·56 (0·26 to 1·22) | 0·15 | -0·566 | (-1·43) | -0·573 | (-1·45) | 1·24 |
| **Material deprivation** |  |  |  |  |  |  |  |
| No deprivation | Ref. |  | Ref. |  |  |  |  |
| Severe deprivation | 1·02 (0·91 to 1·13) | 0·77 | 0·0143 | -0·27 | 0·0157 | -0·29 | 9·79 |
| **Chronic conditions** |  |  |  |  |  |  |  |
| No chronic disease | Ref. |  | Ref. |  |  |  |  |
| One chronic disease | 0·95 (0·91 to 1) | 0·063 | -0·0472 | (-1·83) | -0·0479 | (-1·86) | 1·48 |
| More than one chronic disease | 0·86 (0·81 to 0·92) | <0·0001 | -0·150*** | (-4·66) | -0·148*** | (-4·59) | -1·33 |
| **Living with a person with disability** |  |  |  |  |  |  |  |
| No | Ref. |  |  |  |  |  |  |
| Yes | 1 (0·94 to 1·06) | 0·99 | 0·00312 | -0·11 | -0·000405 | (-0·01) | -112·98 |
| **Inadequate health literacy** |  |  |  |  |  |  |  |
| No | Ref. |  |  |  |  |  |  |
| Yes | 1·13 (1·08 to 1·19) | <0·0001 | 0·126*** | -5·57 | 0·126*** | -5·55 | 0·00 |
| **Knowing someone who had AEFI** |  |  |  |  |  |  |  |
| No | Ref. |  | Ref. |  |  |  |  |
| Yes | 3·37 (3·21 to 3·54) | <0·0001 | 1·214*** | -48·72 | 1·216*** | -48·76 | 0·16 |
| **Knowing someone who had VPD** |  |  |  |  |  |  |  |
| No | Ref. |  | Ref. |  |  |  |  |
| Yes | 0·52 (0·5 to 0·55) | <0·0001 | -0·648*** | (-22·88) | -0·647*** | (-22·83) | -0·15 |
| **Reported barriers to vaccination** |  |  |  |  |  |  |  |
| No | Ref. |  | Ref. |  |  |  |  |
| Yes | 1·33 (1·27 to 1·39) | <0·0001 | 0·285*** | -12·36 | 0·284*** | -12·32 | -0·35 |
| **Information source cluster** |  |  |  |  |  |  |  |
| Diversified sources | Ref. |  | Ref. |  |  |  |  |
| Professional-only sources | 0·73 (0·69 to 0·76) | <0·0001 | -0·320*** | (-13·72) | -0·321*** | (-13·73) | 0·31 |
| **Trust in sources** | 0·44 (0·43 to 0·46) | <0·0001 | -0·813*** | (-40·71) | -0·813*** | (-40·69) | 0·00 |
| **By religious leaders** |  |  |  |  |  |  |  |
| Yes | Ref. |  | Ref. |  |  |  |  |
| No | 1·27 (1·17 to 1·37) | <0·0001 | 0·241*** | -6·26 | 0·236*** | -6·13 | -2·07 |
| Don’t know | 0·98 (0·92 to 1·05) | 0·59 | -0·0177 | (-0·56) | -0·017 | (-0·54) | -3·95 |
| **By political leaders** |  |  |  |  |  |  |  |
| Yes | Ref. |  | Ref. |  |  |  |  |
| No | 1·05 (0·97 to 1·13) | 0·23 | 0·052 | -1·34 | 0·0469 | -1·21 | -9·81 |
| Don’t know | 0·69 (0·64 to 0·73) | <0·0001 | -0·377*** | (-11·57) | -0·377*** | (-11·58) | 0·00 |
| **By teachers** |  |  |  |  |  |  |  |
| Yes | Ref. |  | Ref. |  |  |  |  |
| No | 1·44 (1·32 to 1·56) | <0·0001 | 0·356*** | -8·61 | 0·362*** | -8·75 | 1·69 |
| Don’t know | 1·33 (1·25 to 1·42) | <0·0001 | 0·286*** | -8·96 | 0·288*** | -9·02 | 0·70 |
| **By health professionals** |  |  |  |  |  |  |  |
| Yes | Ref. |  | Ref. |  |  |  |  |
| No | 1·64 (1·51 to 1·78) | <0·0001 | 0·492*** | -11·66 | 0·493*** | -11·67 | 0·20 |
| Don’t know | 1·86 (1·75 to 1·98) | <0·0001 | 0·625*** | -19·42 | 0·622*** | -19·32 | -0·48 |
| **Use of non-conventional medicine** |  |  |  |  |  |  |  |
| No | Ref. |  | Ref. |  |  |  |  |
| Yes, integrated with conventional medicine | 1·21 (1·15 to 1·28) | <0·0001 | 0·192*** | -7·14 | 0·194*** | -7·2 | 1·04 |
| Yes, as alternative to conventional medicine | 2·28 (2·1 to 2·46) | <0·0001 | 0·820*** | -20·35 | 0·822*** | -20·41 | 0·24 |
| **Political orientation** |  |  |  |  |  |  |  |
| Right (7–9) | Ref. |  | Ref. |  |  |  |  |
| Centre (4–6) | 0·96 (0·91 to 1·02) | 0·19 | -0·0393 | (-1·31) | -0·0392 | (-1·31) | -0·25 |
| Extreme left (0) | 0·66 (0·59 to 0·75) | <0·0001 | -0·413*** | (-6·85) | -0·410*** | (-6·80) | -0·73 |
| Left (1–3) | 0·55 (0·51 to 0·6) | <0·0001 | -0·584*** | (-15·10) | -0·589*** | (-15·21) | 0·86 |
| Extreme right (10) | 1·11 (0·99 to 1·25) | 0·065 | 0·104 | -1·76 | 0·109 | -1·85 | 4·81 |
| Non-aligned with traditional parties | 1·03 (0·97 to 1·11) | 0·34 | 0·0318 | -0·91 | 0·0334 | -0·95 | 5·03 |
| Prefer not to answer | 0·94 (0·85 to 1·03) | 0·19 | -0·0553 | (-1·15) | -0·0634 | (-1·32) | 14·65 |
| **Religion** |  |  |  |  |  |  |  |
| Catholic | Ref. |  | Ref. |  |  |  |  |
| Orthodox | 0·93 (0·81 to 1·07) | 0·303 | -0·0717 | (-1·03) | -0·0718 | (-1·03) | 0·14 |
| Protestant | 1·43 (1·13 to 1·81) | 0·0031 | 0·358** | -2·96 | 0·358** | -2·96 | 0·00 |
| Jewish | 1·95 (1·28 to 2·98) | 0·0019 | 0·671** | -3·12 | 0·670** | -3·11 | -0·15 |
| Muslim | 1·19 (0·95 to 1·48) | 0·13 | 0·172 | -1·54 | 0·171 | -1·53 | -0·58 |
| Jehovah’s Witness | 1·4 (1·12 to 1·76) | 0·0036 | 0·337** | -2·9 | 0·339** | -2·91 | 0·59 |
| Atheist | 0·93 (0·85 to 1·02) | 0·14 | -0·0713 | (-1·57) | -0·0679 | (-1·49) | -4·77 |
| Agnostic | 0·91 (0·8 to 1·03) | 0·13 | -0·076 | (-1·18) | -0·0983 | (-1·52) | 29·34 |
| Buddhist | 1·28 (0·96 to 1·71) | 0·099 | 0·246 | -1·66 | 0·245 | -1·65 | -0·41 |
| Hindu | 2·42 (1·24 to 4·73) | 0·0094 | 0·778* | -2·33 | 0·886** | -2·6 | 13·88 |
| Other | 1·42 (1·21 to 1·67) | <0·0001 | 0·351*** | -4·22 | 0·352*** | -4·23 | 0·28 |
| Prefer not to answer | 1·26 (1·14 to 1·4) | <0·0001 | 0·231*** | -4·4 | 0·233*** | -4·43 | 0·87 |
| **Importance of religion** |  |  |  |  |  |  |  |
| Not at all (0) | Ref. |  | Ref. |  |  |  |  |
| Slightly (1–3) | 1·16 (1·06 to 1·27) | 0·0012 | 0·141** | -3·11 | 0·147** | -3·25 | 4·26 |
| Somewhat important (4–6) | 1·27 (1·16 to 1·39) | <0·0001 | 0·235*** | -5·24 | 0·240*** | -5·36 | 2·13 |
| Very (7–9) | 1·19 (1·08 to 1·3) | 0·00022 | 0·165*** | -3·56 | 0·171*** | -3·7 | 3·64 |
| Extremely (10) | 1·08 (0·97 to 1·21) | 0·17 | 0·0809 | -1·44 | 0·0771 | -1·37 | -4·70 |
| Prefer not to answer | 1·6 (1·4 to 1·81) | <0·0001 | 0·458*** | -7·06 | 0·467*** | -7·19 | 1·97 |
| **Perceived NHS quality** | 0·9 (0·89 to 0·92) | <0·0001 | -0·102*** | (-13·96) | -0·103*** | (-13·99) | 0·98 |
| **Perceived NHS access** | 0·91 (0·9 to 0·92) | <0·0001 | -0·0920*** | (-13·01) | -0·0922*** | (-13·03) | 0·22 |
| **Survey mode** |  |  |  |  |  |  |  |
| CAWI | Ref. |  | Ref. |  |  |  |  |
| CATI | 0·77 (0·73 to 0·83) | <0·0001 | -0·253*** | (-7·75) | -0·255*** | (-7·80) | 0·79 |

Abbreviations: adjOR adjusted Odds Ratio, AEFI Adverse Event Following Immunisation, CATI Computer-Assisted Telephone Interviewing, CAWI Computer-Assisted Web Interviewing, CI Confidence Interval, NHS National Health Service, VPD Vaccine Preventable Disease.

* p<0.05, ** p<0.01, *** p<0.001

**M5.4 Calibration plot**


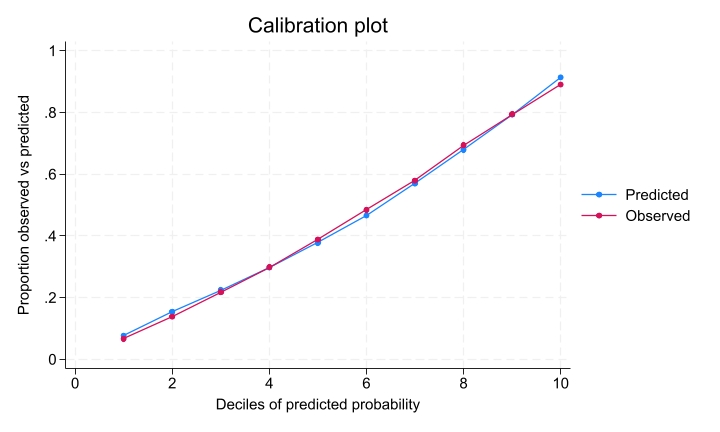


**M5.5 Bootstrap validation of the vaccine hesitancy final logistic regression model (blocks 1-6)**

| **Metric** | **Apparent performance** | **Optimism-adjusted performance (number of replications: 50)** |
| --- | --- | --- |
| **Discrimination** |  |  |
| C-Statistic | 0.812 (95% CI:0.808 to 0.816) | 0.811 (95% CI: 0.807 to 0.814) |
| **Calibration** |  |  |
| Calibration-in-the-large (CITL) | 0 (95% CI: –0.021 to 0.021) | 0.002 (–0.020 to 0.025) |
| Calibration slope | 1 (95% CI: 0.981 to 1.019) | 0.992 (95% CI: 0.970 to 1.011) |
| E:O ratio (expected:observed) | 1 | 1 (95% CI: 0.991 to 1.008 |
| **Overall accuracy** |  |  |
| Brier score scaled (%) | 29 | 28.7 |
| **Shrinkage factors** |  |  |
| Heuristic shrinkage | 0.994 |  |
| Bootstrap shrinkage | 0.992 |  |

**M5.6 aVHS Lack of Trust: Added-variable (partial regression) plot: Trust in sources**


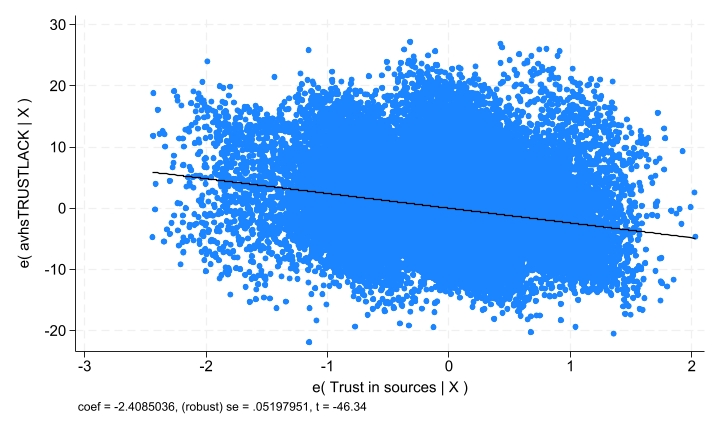


**M5.7 aVHS Lack of Trust: Added-variable (partial regression) plot: Perceived National Health Service quality**


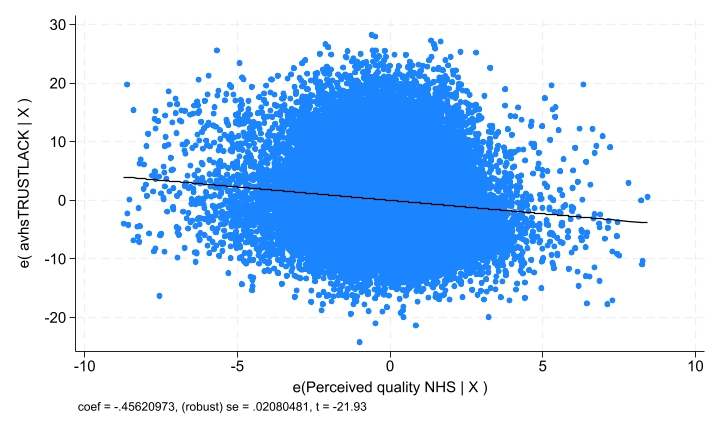


**M5.8 aVHS Lack of Trust: Added-variable (partial regression) plot: Perceived National Health Service access**


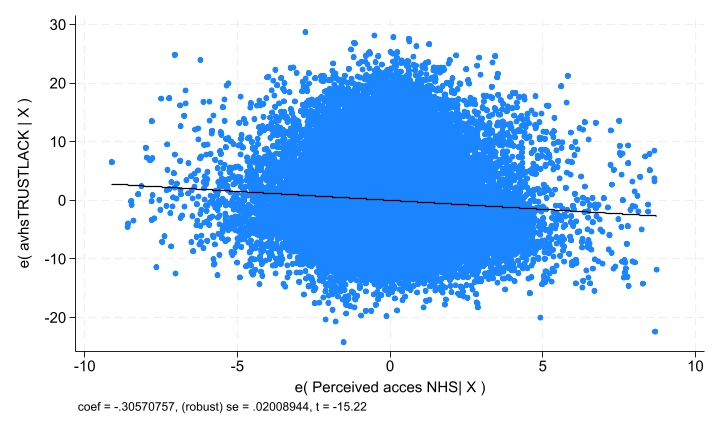


**M5.9 aVHS Lack of Trust: Added-variable (partial regression) plot: Vaccine Conspiracy Belief Scale**


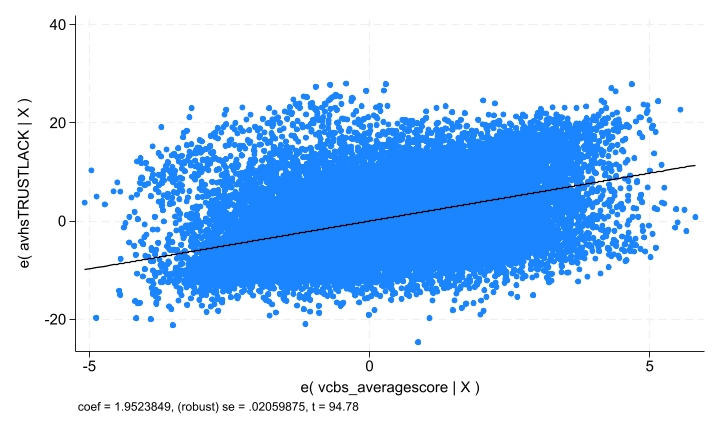


**M5.10 aVHS Risk perception: Added-variable (partial regression) plot: Trust in sources**


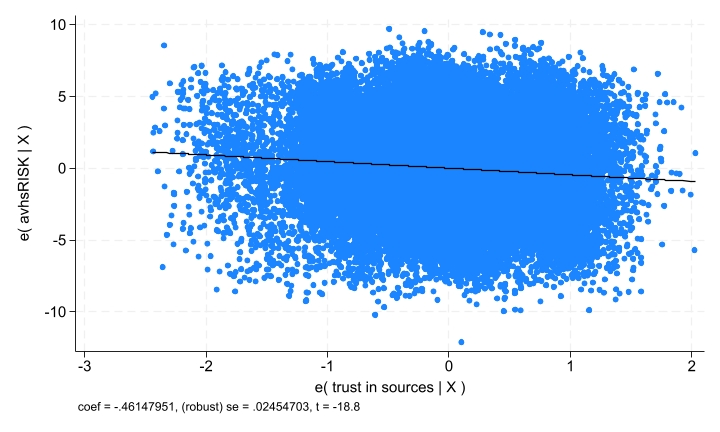


**M5.11 aVHS Risk perception: Added-variable (partial regression) plot: Perceived National Health Service quality**


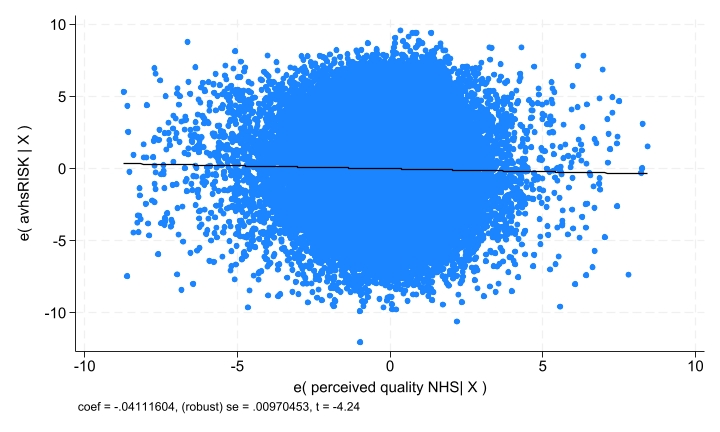


**M5.12 aVHS Risk perception: Added-variable (partial regression) plot: Perceived National Health Service access**


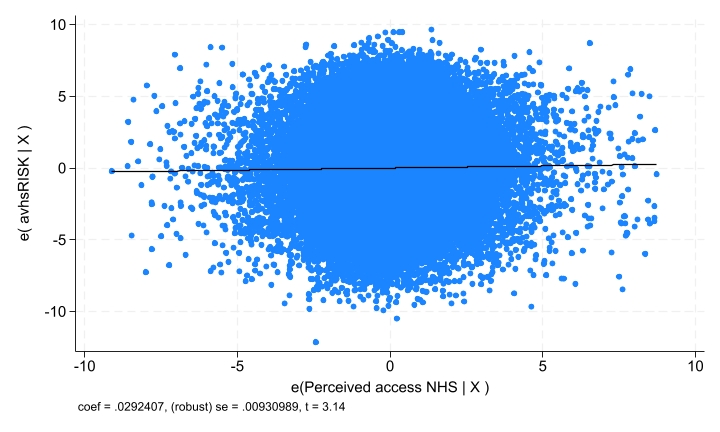


**M5.13 aVHS Risk perception: Added-variable (partial regression) plot: Vaccine Conspiracy Belief Scale**


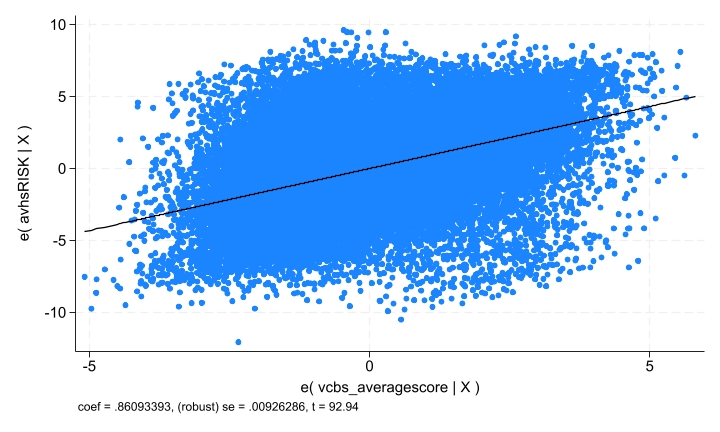


**M5.14 aVHS total score (sensitivity analysis): Added-variable (partial regression) plot: Trust in sources**


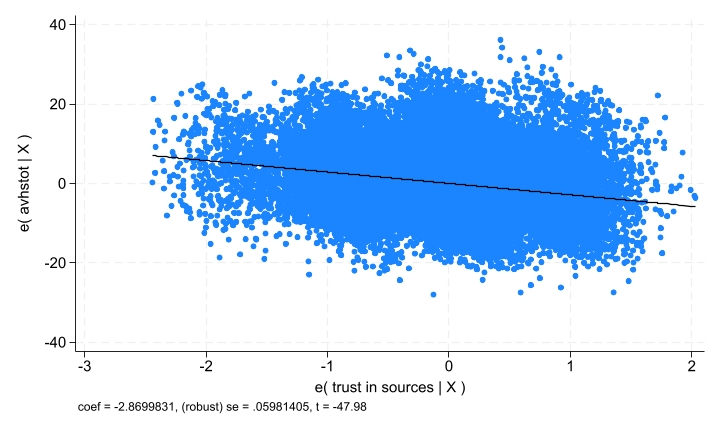


**M5.15 aVHS total score (sensitivity analysis): Added-variable (partial regression) plot: Perceived National Health Service quality**


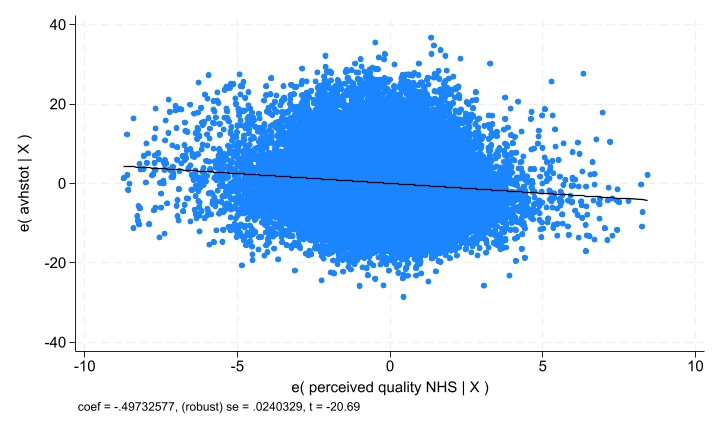


**M5.16 aVHS total score (sensitivity analysis): Added-variable (partial regression) plot: Perceived National Health Service access**


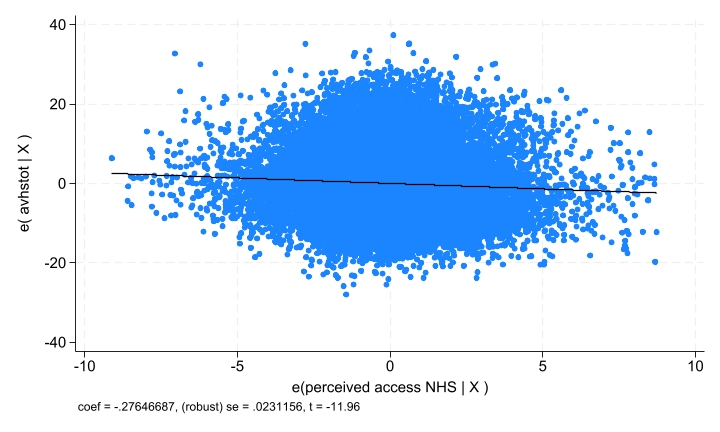


**M5.17 aVHS total score (sensitivity analysis): Added-variable (partial regression) plot: Vaccine Conspiracy Belief Scale**


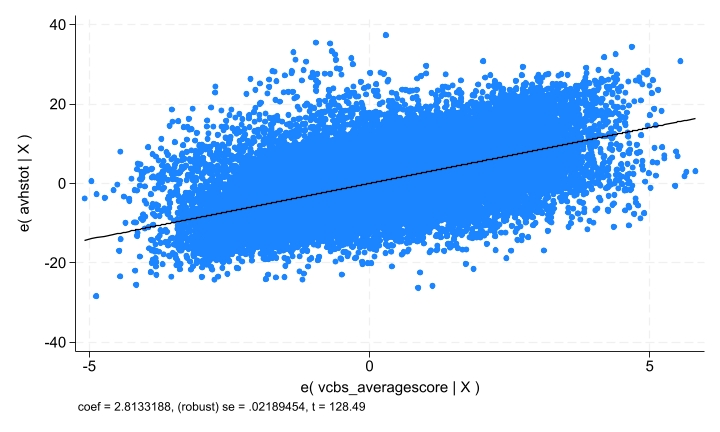


**M5.18 aVHS Lack of Trust: kernel density estimation**


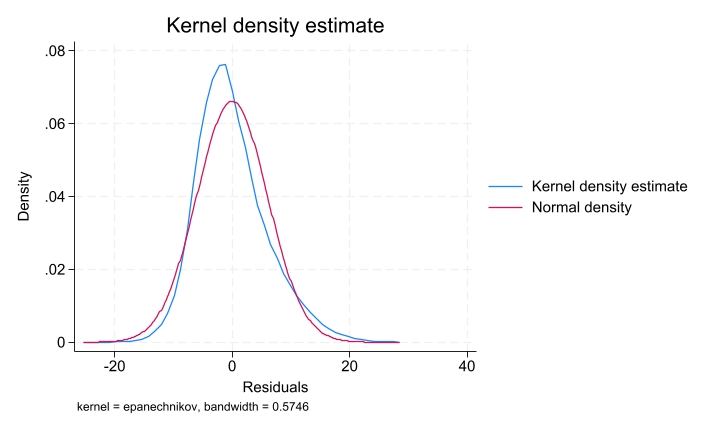


**M5.19 aVHS Lack of Trust: normal Q-Q plot**


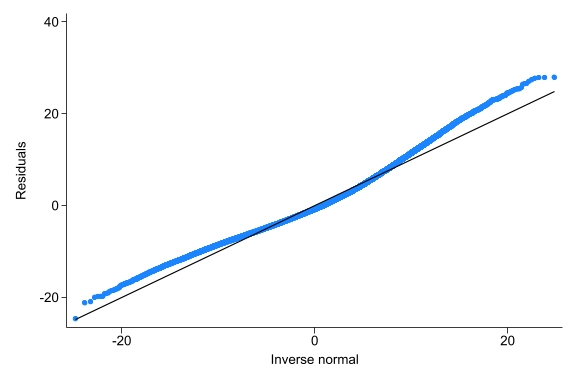


**M5.20 aVHS Risk perception: kernel density estimation**


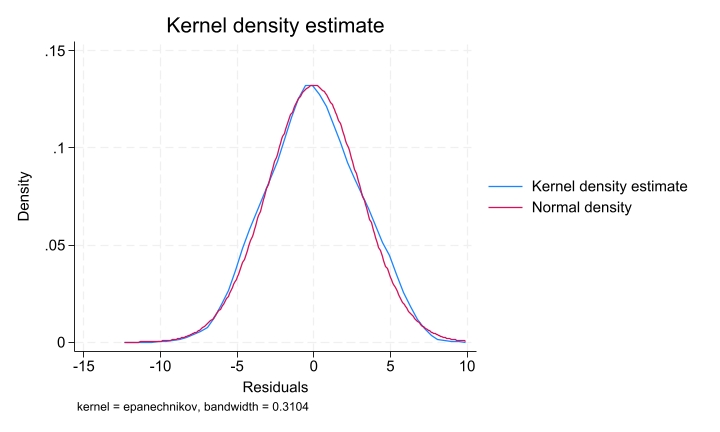


**M5.21 aVHS Risk perception: normal Q-Q plot**


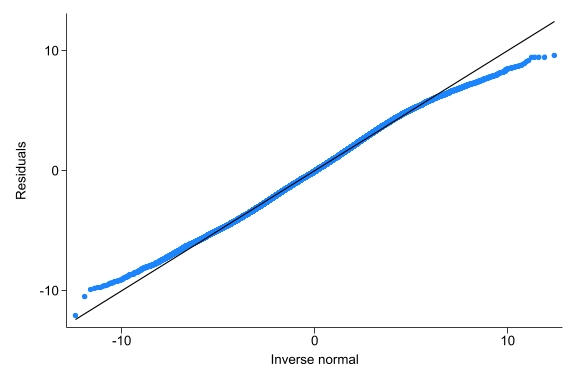


**M5.22 aVHS total score (sensitivity analysis): kernel density estimation**


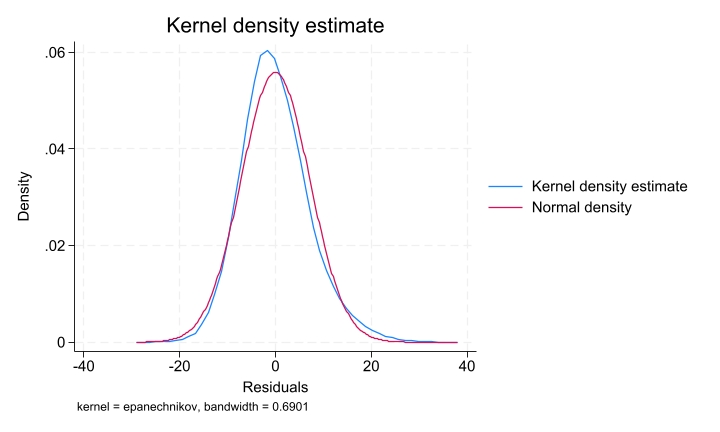


**M5.23 aVHS total score (sensitivity analysis): normal Q-Q plot**


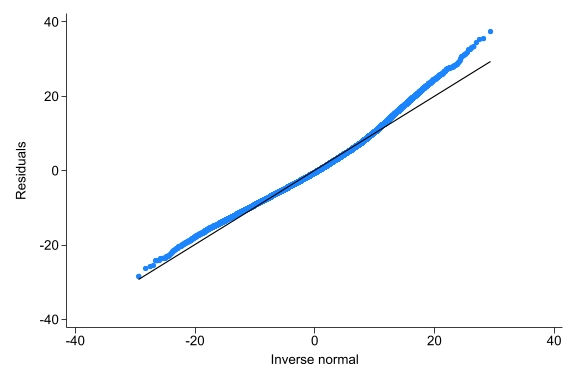


## M6. References Supplementary Methods

1 ISTAT - Istituto Nazionale di Statistica. Banche dati. https://www.istat.it/en/data/databases/ (accessed July 7, 2025).

2 Ledda C, Costantino C, Liberti G, Rapisarda V. The Italian Version of the Adult Vaccine Hesitancy Scale (aVHS) for the Working-Age Population: Cross-Cultural Adaptation, Reliability, and Validity. *Vaccines (Basel)* 2022; **10**: 224.

3 Akel KB, Masters NB, Shih S-F, Lu Y, Wagner AL. Modification of a vaccine hesitancy scale for use in adult vaccinations in the United States and China. *Hum Vaccin Immunother* 2021; **17**: 2639–46.

4 Vicario CM, Mucciardi M, Faraone G, *et al.* Individual predictors of vaccine hesitancy in the Italian post COVID-19 pandemic era. *Hum Vaccin Immunother* 2024; **20**. DOI:10.1080/21645515.2024.2306677.

5 Alghalyini B, Garatli T, Hamoor R, *et al.* Hesitance and Misconceptions about the Annual Influenza Vaccine among the Saudi Population Post-COVID-19. *Vaccines (Basel)* 2023; **11**: 1595.

6 Joachim G, Shih S-F, Singh A, *et al.* Parental vaccine hesitancy and acceptance of a COVID-19 vaccine: An internet-based survey in the US and five Asian countries. *PLOS Global Public Health* 2024; **4**: e0002961.

7 Li X, Zhang F, Li M, Lin C, Shi K, Yang F. Effect of vaccine hesitancy on female college students’ willingness to receive the HPV vaccine in China: a multicenter cross-sectional study. *BMC Public Health* 2024; **24**: 1930.

8 Xu Y, Cao Y, Ma Y, *et al.* COVID-19 Vaccination Attitudes With Neuromyelitis Optica Spectrum Disorders: Vaccine Hesitancy and Coping Style. *Front Neurol* 2021; **12**: 717111.

9 Kafadar AH, Tekeli GG, Jones KA, Stephan B, Dening T. Determinants for COVID-19 vaccine hesitancy in the general population: a systematic review of reviews. *J Public Health (Bangkok)* 2023; **31**: 1829–45.

10 Troiano G, Nardi A. Vaccine hesitancy in the era of COVID-19. *Public Health* 2021; **194**: 245–51.

11 Veronese N, Saccaro C, Demurtas J, *et al.* Prevalence of unwillingness and uncertainty to vaccinate against COVID-19 in older people: A systematic review and meta-analysis. *Ageing Res Rev* 2021; **72**: 101489.

12 Traister T. COVID-19 Vaccine Accessibility, Perceptions, and Attitudes in the LGBTQ+ Community. *West J Nurs Res* 2023; **45**: 1130–8.

13 INAPP - Istituto nazionale per l’analisi delle politiche pubbliche. European Social Survey (Round 10): Opinioni e atteggiamenti su nuove tecnologie, benessere, lavoro, apprendimento, salute, immigrazione. https://www.astrid-online.it/static/upload/inap/inapp_european-social-survey-round-10_ir-41_2023.pdf (accessed July 7, 2025).

14 Presidenza del Consiglio dei Ministri Conferenza permanente per i rapporti tra lo stato le regioni e le province autonome. Intesa, ai sensi dell’articolo 8, comma 6, della legge 5 giugno 2003, n. 131, tra il Governo, le regioni e le Province autonome di Trento e di Bolzano, sul documento recante «Piano nazionale di prevenzione vaccinale (PNPV) 2023-2025» e sul documento recante «Calendario nazionale vaccinale». (Rep. atti n. 193/CSR del 2 agosto 2023). (23A04685). CSR, 2023 https://www.trovanorme.salute.gov.it/norme/dettaglioAtto.spring?id=95963&page=newsett (accessed July 7, 2025).

15 Pastorelli E, Stocchiero A. Inequalities in Italy. 2019 https://www.sdgwatcheurope.org/wp-content/uploads/2019/06/8.3.a-report-IT.pdf (accessed July 7, 2025).

16 Babalola S. Maternal reasons for non‐immunisation and partial immunisation in northern Nigeria. *J Paediatr Child Health* 2011; **47**: 276–81.

17 Danis K, Georgakopoulou T, Stavrou T, Laggas D, Panagiotopoulos T. Socioeconomic factors play a more important role in childhood vaccination coverage than parental perceptions: a cross-sectional study in Greece. *Vaccine* 2010; **28**: 1861–9.

18 ISTAT - Istituto Nazionale di Statistica. Popolazione residente e dinamica della popolazione Anno 2023. 2024 https://www.istat.it/wp-content/uploads/2024/12/CENSIMENTO-E-DINAMICA-DELLA-POPOLAZIONE-2023.pdf (accessed July 7, 2025).

19 ISTAT - Istituto Nazionale di Statistica. La geografia delle aree interne nel 2020 - vasti territori tra potenzialità e debolezze. https://www.istat.it/comunicato-stampa/la-geografia-delle-aree-interne-nel-2020-vasti-territori-tra-potenzialita-e-debolezze/ (accessed July 7, 2025).

20 Dhanani LY, Franz B. A meta-analysis of COVID-19 vaccine attitudes and demographic characteristics in the United States. *Public Health* 2022; **207**: 31–8.

21 Wei F, Mullooly JP, Goodman M, *et al.* Identification and characteristics of vaccine refusers. *BMC Pediatr* 2009; **9**: 18.

22 Antai D. Gender inequities, relationship power, and childhood immunization uptake in Nigeria: a population-based cross-sectional study. *International Journal of Infectious Diseases* 2012; **16**: e136–45.

23 Eurostat. Glossary:At risk of poverty or social exclusion (AROPE). https://ec.europa.eu/eurostat/statistics-explained/index.php?title=Glossary:At_risk_of_poverty_or_social_exclusion_(AROPE) (accessed July 7, 2025).

24 Balaji JN, Prakash S, Joshi A, Surapaneni KM. A Scoping Review on COVID-19 Vaccine Hesitancy among the Lesbian, Gay, Bisexual, Transgender, Queer, Intersex and Asexual (LGBTQIA+) Community and Factors Fostering Its Refusal. *Healthcare* 2023; **11**: 245.

25 McNaghten AD, Brewer NT, Hung M-C, *et al.* COVID-19 Vaccination Coverage and Vaccine Confidence by Sexual Orientation and Gender Identity — United States, August 29–October 30, 2021. *MMWR Morb Mortal Wkly Rep* 2022; **71**: 171–6.

26 Khan MS, Ali SAM, Adelaine A, Karan A. Rethinking vaccine hesitancy among minority groups. *The Lancet* 2021; **397**: 1863–5.

27 Lazarus J V., Ratzan SC, Palayew A, *et al.* A global survey of potential acceptance of a COVID-19 vaccine. *Nat Med* 2021; **27**: 225–8.

28 Wilson RF, Kota KK, Sheats KJ, *et al.* Call out racism and inequity in reports on vaccine intentions. *Nat Hum Behav* 2023; **7**: 300–2.

29 Ufficio federale di statistica. Elenco degli Stati e dei territori (MS-Excel). 2024. https://www.bfs.admin.ch/bfs/it/home/statistiche/cataloghi-banche-dati.assetdetail.32028071.html (accessed July 7, 2025).

30 Nindrea RD, Usman E, Katar Y, Sari NP. Acceptance of COVID-19 vaccination and correlated variables among global populations: A systematic review and meta-analysis. *Clin Epidemiol Glob Health* 2021; **12**: 100899.

31 Nehal KR, Steendam LM, Campos Ponce M, van der Hoeven M, Smit GSA. Worldwide Vaccination Willingness for COVID-19: A Systematic Review and Meta-Analysis. *Vaccines (Basel)* 2021; **9**: 1071.

32 Lorini C, Santomauro F, Donzellini M, *et al.* Health literacy and vaccination: A systematic review. *Hum Vaccin Immunother* 2018; **14**: 478–88.

33 Morris NS, MacLean CD, Chew LD, Littenberg B. The Single Item Literacy Screener: Evaluation of a brief instrument to identify limited reading ability. *BMC Fam Pract* 2006; **7**: 21.

34 Bonaccorsi G, Grazzini M, Pieri L, Santomauro F, Ciancio M, Lorini C. Assessment of Health Literacy and validation of single-item literacy screener (SILS) in a sample of Italian people. *Ann Ist Super Sanita* 2017; **53**: 205–12.

35 Gauna F, Raude J, Khouri C, Cracowski J-L, Ward JK. Exploring the relationship between experience of vaccine adverse events and vaccine hesitancy: A scoping review. *Hum Vaccin Immunother* 2025; **21**. DOI:10.1080/21645515.2025.2471225.

36 Goje O, Kapoor A. Meeting the challenge of vaccine hesitancy. *Cleve Clin J Med* 2024; **91**: S50–6.

37 Dubé E, Laberge C, Guay M, Bramadat P, Roy R, Bettinger JA. Vaccine hesitancy. *Hum Vaccin Immunother* 2013; **9**: 1763–73.

38 Gautier A, Verger P, Jestin C, groupe Baromètre santé 2016*. Sources d’information, opinions et pratiques des parents en matière de vaccination en  France en  2016. *Bulletin Epidémiologique Hebdomadaire* 2017; : 28–35.

39 Charron J, Gautier A, Jestin C. Influence of information sources on vaccine hesitancy and practices. *Med Mal Infect* 2020; **50**: 727–33.

40 MacDonald NE. Vaccine hesitancy: Definition, scope and determinants. *Vaccine* 2015; **33**: 4161–4.

41 Hornsey MJ, Lobera J, Díaz-Catalán C. Vaccine hesitancy is strongly associated with distrust of conventional medicine, and only weakly associated with trust in alternative medicine. *Soc Sci Med* 2020; **255**: 113019.

42 Kennedy J. Populist politics and vaccine hesitancy in Western Europe: an analysis of national-level data. *Eur J Public Health* 2019; **29**: 512–6.

43 Ye X. Exploring the relationship between political partisanship and COVID-19 vaccination rate. *J Public Health (Bangkok)* 2023; **45**: 91–8.

44 Aybar C, Pérez V, Pavía JM. Scale matters: unravelling the impact of Likert scales on political self-placement. *Qual Quant* 2024; **58**: 3725–46.

45 Grabenstein JD. What the World’s religions teach, applied to vaccines and immune globulins. *Vaccine* 2013; **31**: 2011–23.

46 Kibongani Volet A, Scavone C, Catalán-Matamoros D, Capuano A. Vaccine Hesitancy Among Religious Groups: Reasons Underlying This Phenomenon and Communication Strategies to Rebuild Trust. *Front Public Health* 2022; **10**. DOI:10.3389/fpubh.2022.824560.

47 Pertwee E, Simas C, Larson HJ. An epidemic of uncertainty: rumors, conspiracy theories and vaccine hesitancy. *Nat Med* 2022; **28**: 456–9.

48 Al-Sanafi M, Sallam M. Psychological Determinants of COVID-19 Vaccine Acceptance among Healthcare Workers in Kuwait: A Cross-Sectional Study Using the 5C and Vaccine Conspiracy Beliefs Scales. *Vaccines (Basel)* 2021; **9**: 701.

49 Portoghese I, Siddi M, Chessa L, *et al.* COVID-19 Vaccine Hesitancy among Italian Healthcare Workers: Latent Profiles and Their Relationships to Predictors and Outcome. *Vaccines (Basel)* 2023; **11**: 273.

50 Shapiro GK, Holding A, Perez S, Amsel R, Rosberger Z. Validation of the vaccine conspiracy beliefs scale. *Papillomavirus Research* 2016; **2**: 167–72.

51 Lumley T, Diehr P, Emerson S, Chen L. The Importance of the Normality Assumption in Large Public Health Data Sets. *Annu Rev Public Health* 2002; **23**: 151–69.

52 Istituto Nazionale di Statistica (ISTAT). Demo: popolazione residente – dati al 1° gennaio 2025. 2025. https://demo.istat.it/app/?i=POS (accessed Nov 9, 2025).

53 Fernandez-Felix BM, García-Esquinas E, Muriel A, Royuela A, Zamora J. Bootstrap internal validation command for predictive logistic regression models. *The Stata Journal: Promoting communications on statistics and Stata* 2021; **21**: 498–509.

54 Richardson JTE. Eta squared and partial eta squared as measures of effect size in educational research. *Educ Res Rev* 2011; **6**: 135–47.

# Supplementary Tables

## Table S1. Unweighted analysis: Sociodemographic, socioeconomic, health-related characteristics and personal experience: analyses with Vaccine Hesitancy as outcome.

|  | **Overall sample (n=52094)** | **Prevalence of Vaccine Hesitancy*** | **Univariable regression** | | **Multivariable regression**** | |
| --- | --- | --- | --- | --- | --- | --- |
|  | **N (%)** | **% (95% CI)** | **OR (95%CI)** | **p** | **adjOR (95% CI)** | **p** |
| **Block 1 - Sociodemographic and socioeconomic characteristics** |  |  |  |  |  |  |
| **Age group** |  | <0·0001 |  |  |  |  |
| 18–29 | 7443 (14·29%) | 45·18% (44·06 to 46·32) | Ref. |  | Ref. |  |
| 30–44 | 10713 (20·56%) | 53·48% (52·53 to 54·42) | 1·39 (1·31 to 1·48) | <0·0001 | 1·47 (1·35 to 1·59) | <0·0001 |
| 45–59 | 14937 (28·67%) | 49·55% (48·75 to 50·35) | 1·19 (1·13 to 1·26) | <0·0001 | 1·58 (1·45 to 1·72) | <0·0001 |
| 60–74 | 11262 (21·62%) | 42·02% (41·11 to 42·93) | 0·88 (0·83 to 0·93) | <0·0001 | 1·79 (1·61 to 1·99) | <0·0001 |
| 75+ | 7739 (14·86%) | 33·84% (32·80 to 34·90) | 0·62 (0·58 to 0·66) | <0·0001 | 1·41 (1·24 to 1·6) | <0·0001 |
| **Gender** |  | <0·0001 |  |  |  |  |
| Male | 25145 (48·27%) | 44·82% (44·21 to 45·44) | Ref. |  | Ref. |  |
| Female | 26428 (50·73%) | 46·28% (45·68 to 46·88) | 1·06 (1·02 to 1·1) | 0·00090 | 1·01 (0·97 to 1·06) | 0·56 |
| Non-binary/Other | 481 (0·92%) | 67·36% (63·04 to 71·40) | 2·54 (2·1 to 3·08) | <0·0001 | 2·12 (1·67 to 2·69) | <0·0001 |
| Prefer not to answer | 40 (0·08%) | 50% (34·98 to 65·02) | 1·23 (0·66 to 2·29) | 0·51 | 1·03 (0·46 to 2·29) | 0·95 |
| **Marital status** |  | <0·0001 |  |  |  |  |
| Single | 11920 (22·88%) | 49·73% (48·83 to 50·63) | Ref. |  | Ref. |  |
| Married | 27741 (53·25%) | 42·94% (42·36 to 43·52) | 0·76 (0·73 to 0·79) | <0·0001 | 0·9 (0·84 to 0·97) | 0·0033 |
| Separated/Divorced | 3625 (6·96%) | 53·96% (52·33 to 55·58) | 1·18 (1·1 to 1·28) | <0·0001 | 1·12 (1·01 to 1·24) | 0·032 |
| Cohabiting | 6308 (12·11%) | 51·43% (50·19 to 52·66) | 1·07 (1·01 to 1·14) | 0·029 | 1·04 (0·96 to 1·12) | 0·39 |
| Widowed | 2500 (4·8%) | 32·16% (30·36 to 34·02) | 0·48 (0·44 to 0·52) | <0·0001 | 0·76 (0·67 to 0·86) | <0·0001 |
| **Children** |  | <0·0001 |  |  |  |  |
| No children | 18727 (35·95%) | 49·89% (49·17 to 50·61) | Ref. |  | Ref. |  |
| Only children ≤11 years | 6271 (12·04%) | 53·32% (52·09 to 54·56) | 1·15 (1·08 to 1·22) | <0·0001 | 0·93 (0·86 to 1·01) | 0·091 |
| Only children 12-18 years | 3518 (6·75%) | 53·41% (51·76 to 55·06) | 1·15 (1·07 to 1·24) | 0·0013 | 0·89 (0·81 to 0·98) | 0·017 |
| Only children >18 years | 20916 (40·15%) | 38·4% (37·74 to 39·06) | 0·63 (0·6 to 0·65) | <0·0001 | 0·78 (0·72 to 0·84) | <0·0001 |
| Children of various ages | 2662 (5·11%) | 46·84% (44·95 to 48·74) | 0·89 (0·82 to 0·96) | 0·0033 | 0·76 (0·68 to 0·84) | <0·0001 |
| **Sexual orientation** |  | <0·0001 |  |  |  |  |
| Heterosexual | 46178 (88·64%) | 45·45% (45·00 to 45·91) | Ref. |  | Ref. |  |
| Homosexual | 851 (1·63%) | 46·3% (42·97 to 49·66) | 1·03 (0·9 to 1·19) | 0·62 | 0·8 (0·67 to 0·94) | 0·0074 |
| Bisexual | 1103 (2·12%) | 46·24% (43·31 to 49·19) | 1·03 (0·92 to 1·16) | 0·605 | 0·77 (0·66 to 0·89) | 0·00067 |
| Pansexual | 367 (0·7%) | 65·4% (60·38 to 70·09) | 2·27 (1·83 to 2·81) | <0·0001 | 0·92 (0·7 to 1·21) | 0·55 |
| Ace spectrum | 499 (0·96%) | 69·14% (64·95 to 73·04) | 2·69 (2·22 to 3·25) | <0·0001 | 0·88 (0·69 to 1·12) | 0·303 |
| Prefer not to answer | 3096 (5·94%) | 44·12% (42·38 to 45·88) | 0·95 (0·88 to 1·02) | 0·15 | 0·65 (0·59 to 0·72) | <0·0001 |
| **Municipality size (inhabitants)** |  | <0·0001 |  |  |  |  |
| ≤10,000 | 15841 (30·41%) | 45·57% (44·80 to 46·35) | Ref. |  |  |  |
| 10,001–25,000 | 10888 (20·9%) | 43·39% (42·46 to 44·32) | 0·92 (0·87 to 0·96) | 0·00042 | 0·98 (0·92 to 1·04) | 0·51 |
| 25,001–50,000 | 7615 (14·62%) | 43·57% (42·46 to 44·69) | 0·92 (0·87 to 0·97) | 0·0039 | 0·98 (0·91 to 1·06) | 0·63 |
| 50,001–100,000 | 5675 (10·89%) | 48·21% (46·91 to 49·51) | 1·11 (1·05 to 1·18) | 0·00062 | 1·06 (0·96 to 1·17) | 0·28 |
| 100,001–250,000 | 3984 (7·65%) | 50·95% (49·40 to 52·50) | 1·24 (1·16 to 1·33) | <0·0001 | 1·09 (0·96 to 1·23) | 0·17 |
| >250,000 | 8091 (15·53%) | 47·18% (46·09 to 48·26) | 1·07 (1·01 to 1·13) | 0·019 | 0·99 (0·88 to 1·1) | 0·82 |
| **Geographic macro-area** |  | 0·00011 |  |  |  |  |
| North-West | 14073 (27·01%) | 46·27% (45·45 to 47·10) | Ref. |  | Ref. |  |
| North-East | 10249 (19·67%) | 45·02% (44·06 to 45·98) | 0·95 (0·9 to 1) | 0·053 | 1·02 (0·96 to 1·09) | 0·52 |
| Centre | 10404 (19·97%) | 44·4% (43·44 to 45·35) | 0·93 (0·88 to 0·98) | 0·0036 | 1 (0·94 to 1·07) | 0·96 |
| South | 11761 (22·58%) | 47·29% (46·39 to 48·20) | 1·04 (0·99 to 1·09) | 0·102 | 0·92 (0·86 to 0·98) | 0·0093 |
| Islands | 5607 (10·76%) | 45·25% (43·95 to 46·55) | 0·96 (0·9 to 1·02) | 0·19 | 0·86 (0·79 to 0·93) | 0·00013 |
| **Degree of urbanisation** |  | <0·0001 |  |  |  |  |
| Pole | 18750 (35·99%) | 47·28% (46·57 to 48·00) | Ref. |  | Ref. |  |
| Intermunicipal pole | 1316 (2·53%) | 44·07% (41·41 to 46·77) | 0·88 (0·79 to 0·98) | 0·024 | 0·96 (0·83 to 1·11) | 0·58 |
| Belt | 19439 (37·32%) | 45·94% (45·24 to 46·65) | 0·95 (0·91 to 0·99) | 0·0089 | 1·09 (0·99 to 1·19) | 0·068 |
| Intermediate | 7460 (14·32%) | 44·41% (43·29 to 45·54) | 0·89 (0·84 to 0·94) | <0·0001 | 1·08 (0·98 to 1·2) | 0·14 |
| Peripheral | 4366 (8·38%) | 41·64% (40·19 to 43·11) | 0·8 (0·74 to 0·85) | <0·0001 | 0·98 (0·87 to 1·1) | 0·76 |
| Ultra-peripheral | 763 (1·46%) | 44·17% (40·68 to 47·71) | 0·88 (0·76 to 1·02) | 0·092 | 0·97 (0·8 to 1·19) | 0·78 |
| **Education level** |  | <0·0001 |  |  |  |  |
| Upper secondary | 27061 (51·95%) | 48·21% (47·61 to 48·81) | Ref. |  | Ref. |  |
| Primary/None | 1458 (2·8%) | 32·44% (30·09 to 34·89) | 0·52 (0·46 to 0·58) | <0·001 | 0·57 (0·49 to 0·66) | <0·0001 |
| Lower secondary | 7850 (15·07%) | 47·01% (45·90 to 48·11) | 0·95 (0·91 to 1) | 0·0603 | 1·05 (0·99 to 1·12) | 0·12 |
| University | 12508 (24·01%) | 42·57% (41·71 to 43·44) | 0·8 (0·76 to 0·83) | <0·0001 | 0·84 (0·8 to 0·89) | <0·0001 |
| Postgraduate | 3217 (6·18%) | 40·72% (39·04 to 42·43) | 0·74 (0·69 to 0·79) | <0·0001 | 0·7 (0·64 to 0·77) | <0·0001 |
| **Occupational status** |  | <0·0001 |  |  |  |  |
| Non-healthcare worker | 23467 (45·87%) | 49·35% (48·71 to 49·99) | Ref. |  | Ref. |  |
| Healthcare worker | 2512 (4·91%) | 42·64% (40·71 to 44·58) | 0·76 (0·7 to 0·83) | <0·0001 | 0·71 (0·64 to 0·78) | <0·0001 |
| Homemaker | 4034 (7·89%) | 52·58% (51·04 to 54·12) | 1·14 (1·06 to 1·22) | 0·00015 | 1·1 (1·01 to 1·2) | 0·03005 |
| Retired | 13979 (27·33%) | 35·81% (35·02 to 36·61) | 0·57 (0·55 to 0·6) | <0·0001 | 0·83 (0·77 to 0·91) | <0·0001 |
| Student (non-health field) | 2079 (4·06%) | 38·72% (36·65 to 40·83) | 0·65 (0·59 to 0·71) | <0·0001 | 0·65 (0·58 to 0·74) | <0·0001 |
| Student (health field) | 809 (1·58%) | 34·98% (31·77 to 38·33) | 0·55 (0·48 to 0·64) | <0·0001 | 0·54 (0·45 to 0·65) | <0·0001 |
| Job seeker | 1863 (3·64%) | 55·45% (53·18 to 57·69) | 1·28 (1·16 to 1·4) | <0·0001 | 1·02 (0·91 to 1·14) | 0·75 |
| Unemployed | 2340 (4·57%) | 58·12% (56·11 to 60·10) | 1·42 (1·31 to 1·55) | <0·0001 | 1·02 (0·92 to 1·14) | 0·67 |
| Other | 74 (0·14%) | 44·59% (33·73 to 56·00) | 0·83 (0·52 to 1·31) | 0·42 | 0·73 (0·42 to 1·25) | 0·25 |
| **Continent of citizenship** |  | 0·26 |  |  |  |  |
| Italy | 51093 (98·1%) | 45·72% (45·29 to 46·15) | Ref. |  | Ref. |  |
| Europe (non-Italy) | 591 (1·13%) | 49·41% (45·39 to 53·43) | 1·16 (0·99 to 1·36) | 0·074 | 1·01 (0·83 to 1·24) | 0·899 |
| Africa | 141 (0·27%) | 51·06% (42·86 to 59·21) | 1·24 (0·89 to 1·72) | 0·204 | 1·16 (0·74 to 1·83) | 0·52 |
| America | 150 (0·29%) | 45·33% (37·55 to 53·35) | 0·98 (0·71 to 1·36) | 0·92 | 0·86 (0·54 to 1·35) | 0·506 |
| Asia | 106 (0·2%) | 42·45% (33·42 to 52·02) | 0·88 (0·6 to 1·29) | 0·4996 | 0·95 (0·54 to 1·66) | 0·85 |
| Oceania | 1 (0%) | 100% (-) | - |  | - |  |
| **Self-identified ethnicity** |  | <0·0001 |  |  |  |  |
| European | 50389 (96·73%) | 45·28% (44·84 to 45·71) | Ref. |  | Ref. |  |
| Multi-ethnic | 394 (0·76%) | 65·99% (61·17 to 70·50) | 2·35 (1·9 to 2·89) | <0·0001 | 1·35 (1·04 to 1·76) | 0·026 |
| North American / Australian | 160 (0·31%) | 71·25% (63·77 to 77·73) | 3 (2·13 to 4·22) | <0·0001 | 1·44 (0·95 to 2·2) | 0·087 |
| Arab-Middle Eastern | 222 (0·43%) | 66·22% (59·74 to 72·13) | 2·37 (1·79 to 3·13) | <0·0001 | 1·05 (0·74 to 1·51) | 0·78 |
| North African | 235 (0·45%) | 58·3% (51·89 to 64·44) | 1·69 (1·3 to 2·19) | <0·0001 | 1·31 (0·92 to 1·87) | 0·14 |
| Latino-American | 347 (0·67%) | 54·76% (49·48 to 59·92) | 1·46 (1·18 to 1·81) | 0·00044 | 0·89 (0·65 to 1·21) | 0·45 |
| African American | 38 (0·07%) | 73·68% (57·63 to 85·22) | 3·38 (1·64 to 6·97) | 0·00094 | 1·15 (0·48 to 2·77) | 0·75 |
| Black African | 95 (0·18%) | 48·42% (38·57 to 58·40) | 1·13 (0·76 to 1·7) | 0·54 | 0·84 (0·5 to 1·42) | 0·52 |
| Asian | 166 (0·32%) | 50·6% (43·04 to 58·14) | 1·24 (0·91 to 1·68) | 0·17 | 0·94 (0·6 to 1·48) | 0·79 |
| Pacific Islands | 48 (0·09%) | 50% (36·22 to 63·78) | 1·21 (0·69 to 2·13) | 0·51 | 0·57 (0·26 to 1·23) | 0·15 |
| **Material deprivation** |  | <0·0001 |  |  |  |  |
| No deprivation | 49884 (95·76%) | 45·47% (45·03 to 45·90) | Ref. |  | Ref. |  |
| Severe deprivation | 2210 (4·24%) | 52·62% (50·54 to 54·70) | 1·33 (1·22 to 1·45) | <0·0001 | 1·01 (0·91 to 1·13) | 0·79 |
| **Block 2 - Health-related characteristics and personal experience** |  |  |  |  |  |  |
| **Chronic conditions** |  | <0·0001 |  |  |  |  |
| No chronic disease | 28070 (53·88%) | 46·65% (46·07 to 47·24) | Ref. |  | Ref. |  |
| One chronic disease | 14717 (28·25%) | 46·8% (45·99 to 47·60) | 1·01 (0·97 to 1·05) | 0·78 | 0·95 (0·91 to 1) | 0·067 |
| More than one chronic disease | 9307 (17·87%) | 41·48 (40·49 to 42·49) | 0·81 (0·77 to 0·85) | <0·00001 | 0·86 (0·81 to 0·92) | <0·0001 |
| **Living with a person with disability** |  | <0·0001 |  |  |  |  |
| No | 43000 (82·54%) | 44·68% (44·21 to 45·15) | Ref. |  | Ref. |  |
| Yes | 9094 (17·46%) | 50·91% (49·88 to 51·94) | 1·28 (1·23 to 1·34) | <0·0001 | 1 (0·95 to 1·06) | 0·91 |
| **Inadequate health literacy** |  | <0·0001 |  |  |  |  |
| No | 30863 (59·24%) | 44·16% (43·61 to 44·71) | Ref. |  | Ref. |  |
| Yes | 21231 (40·76%) | 48·11% (47·44 to 48·79) | 1·17 (1·13 to 1·21) | <0·0001 | 1·13 (1·09 to 1·19) | <0·0001 |
| **Knowing someone who had AEFI** |  | <0·0001 |  |  |  |  |
| No | 35485 (68·12%) | 35·88% (35·39 to 36·38) | Ref. |  | Ref. |  |
| Yes | 16609 (31·88%) | 66·9% (66·18 to 67·61) | 3·61 (3·47 to 3·75) | <0·0001 | 3·37 (3·21 to 3·54) | <0·0001 |
| **Knowing someone who had VPD** |  | <0·0001 |  |  |  |  |
| No | 40222 (77·21%) | 46·44% (45·96 to 46·93) | Ref. |  | Ref. |  |
| Yes | 11872 (22·79%) | 43·49% (42·60 to 44·38) | 0·89 (0·85 to 0·92) | <0·0001 | 0·52 (0·49 to 0·55) | <0·0001 |
| **Reported barriers to vaccination** |  | <0·0001 |  |  |  |  |
| No | 27576 (52·94%) | 37·92% (37·35 to 38·49) | Ref. |  | Ref. |  |
| Yes | 24518 (47·06%) | 45·4% (44·77 to 46·02) | 1·97 (1·9 to 2·04) | <0·0001 | 1·33 (1·27 to 1·39) | <0·0001 |

*p-value in this column were obtained via Chi-squared tests.

**The multivariable regression model reported in Table 1 was adjusted for all the variables presented in Tables 1 and 2 (Block 1-6).

Abbreviations: adjOR adjusted Odds Ratio, AEFI Adverse Event Following Immunisation, CI Confidence Interval, OR Odds Ratio, VPD Vaccine Preventable Disease.

Note: Regression estimates should not be interpreted as causal effects.

## Table S2. Unweighted analysis: Information sources and trust, external influences, beliefs, attitudes and survey mode: analyses with Vaccine Hesitancy as outcome.

|  | **Overall sample (n=52094)** | **Prevalence of Vaccine Hesitancy*** | **Univariable regression** | | **Multivariable regression**** | |
| --- | --- | --- | --- | --- | --- | --- |
|  | **N (%)** | **% (95% CI)** | **OR (95%CI)** | **p** | **adjOR (95% CI)** | **p** |
| **Block 3 - Information sources and trust** |  |  |  |  |  |  |
| **Information source cluster** |  | <0·0001 |  |  |  |  |
| Diversified sources | 32741 (62·85%) | 53·1% (52·56 to 53·64) | Ref. |  | Ref. |  |
| Professional-only sources | 19353 (37·15%) | 33·37% (32·71 to 34·04) | 0·44 (0·43 to 0·46) | <0·0001 | 0·73 (0·69 to 0·76) | <0·0001 |
| **Trust in sources***** | 2·98 (0·65%) | - | 0·3 (0·29 to 0·31) | <0·0001 | 0·44 (0·43 to 0·46) | <0·0001 |
| **Block 4 – External influences (perceived vaccination endorsement in the respondent’s community by:)** |  |  |  |  |  |  |
| **By religious leaders** |  | <0·0001 |  |  |  |  |
| Yes | 14188 (27·24%) | 40·96% (40·16 to 41·78) | Ref. |  | Ref. |  |
| No | 10073 (19·34%) | 61·91% (60·96 to 62·85) | 2·34 (2·22 to 2·47) | <0·0001 | 1·27 (1·18 to 1·37) | <0·0001 |
| Don’t know | 27833 (53·43%) | 42·38% (41·80 to 42·96) | 1·06 (1·02 to 1·1) | 0·0054 | 0·98 (0·92 to 1·04) | 0·57 |
| **By political leaders** |  | <0·0001 |  |  |  |  |
| Yes | 20679 (39·7%) | 42·51% (41·83 to 43·18) | Ref. |  | Ref. |  |
| No | 9144 (17·55%) | 63·13% (62·14 to 64·12) | 2·32 (2·2 to 2·44) | <0·0001 | 1·05 (0·98 to 1·14) | 0·18 |
| Don’t know | 22271 (42·75%) | 41·67% (41·03 to 42·32) | 0·97 (0·93 to 1) | 0·0803 | 0·69 (0·64 to 0·73) | <0·0001 |
| **By teachers** |  | <0·0001 |  |  |  |  |
| Yes | 23393 (44·91%) | 38·05% (37·43 to 38·68) | Ref. |  | Ref. |  |
| No | 7735 (14·85%) | 67·67% (66·62 to 68·70) | 3·41 (3·23 to 3·6) | <0·0001 | 1·43 (1·32 to 1·55) | <0·0001 |
| Don’t know | 20966 (40·25%) | 46·3% (45·63 to 46·98) | 1·4 (1·35 to 1·46) | <0·0001 | 1·33 (1·25 to 1·42) | <0·0001 |
| **By health professionals** |  | <0·0001 |  |  |  |  |
| Yes | 32162 (61·74%) | 37·67% (37·14 to 38·20) | Ref. |  | Ref. |  |
| No | 6345 (12·18%) | 69·91% (68·77 to 71·03) | 3·85 (3·63 to 4·08) | <0·0001 | 1·64 (1·51 to 1·78) | <0·0001 |
| Don’t know | 13587 (26·08%) | 53·68% (52·84 to 54·51) | 1·92 (1·84 to 2) | <0·0001 | 1·87 (1·75 to 1·99) | <0·0001 |
| **Block 5 – Beliefs and attitudes** |  |  |  |  |  |  |
| **Use of non-conventional medicine** |  | <0·0001 |  |  |  |  |
| No | 35437 (68·03%) | 40·35% (39·84 to 40·86) | Ref. |  | Ref. |  |
| Yes, integrated with conventional medicine | 11799 (22·65%) | 51·75% (50·85 to 52·65) | 1·59 (1·52 to 1·65) | <0·0001 | 1·21 (1·15 to 1·28) | <0·0001 |
| Yes, as alternative to conventional medicine | 4858 (9·33%) | 70·79% (69·50 to 72·05) | 3·58 (3·36 to 3·82) | <0·0001 | 2·27 (2·1 to 2·46) | <0·0001 |
| **Political orientation** |  | <0·001 |  |  |  |  |
| Right (7–9) | 10957 (21·03%) | 47·05% (46·11 to 47·98) | Ref. |  | Ref. |  |
| Centre (4–6) | 16099 (30·9%) | 50·67% (49·90 to 51·44) | 1·16 (1·1 to 1·21) | <0·0001 | 0·96 (0·91 to 1·02) | 0·19 |
| Extreme left (0) | 2158 (4·14%) | 44·76% (42·68 to 46·87) | 0·91 (0·83 to 1) | 0·052 | 0·66 (0·59 to 0·74) | <0·0001 |
| Left (1–3) | 7184 (13·79%) | 33·89% (32·81 to 35·00) | 0·58 (0·54 to 0·61) | <0·0001 | 0·56 (0·52 to 0·6) | <0·0001 |
| Extreme right (10) | 2041 (3·92%) | 52·08% (49·91 to 54·24) | 1·22 (1·11 to 1·34) | <0·001 | 1·11 (0·99 to 1·24) | 0·078 |
| Non-aligned with traditional parties | 9304 (17·86%) | 49·31% (48·30 to 50·33) | 1·09 (1·04 to 1·16) | 0·0013 | 1·03 (0·96 to 1·11) | 0·37 |
| Prefer not to answer | 4351 (8·35%) | 34·02% (32·62 to 35·44) | 0·58 (0·54 to 0·62) | <0·0001 | 0·95 (0·86 to 1·04) | 0·25 |
| **Religion** |  | <0·001 |  |  |  |  |
| Catholic | 37400 (71·79%) | 44·76% (44·25 to 45·26) | Ref. |  | Ref. |  |
| Orthodox | 1557 (2·99%) | 40·72% (38·30 to 43·18) | 0·85 (0·76 to 0·94) | 0·0017 | 0·93 (0·81 to 1·07) | 0·304 |
| Protestant | 455 (0·87%) | 64·4% (59·89 to 68·66) | 2·23 (1·84 to 2·71) | <0·0001 | 1·43 (1·13 to 1·81) | 0·00307 |
| Jewish | 167 (0·32%) | 72·46% (65·20 to 78·70) | 3·25 (2·31 to 4·56) | <0·0001 | 1·96 (1·28 to 2·98) | 0·0018 |
| Muslim | 671 (1·29%) | 57·53% (53·75 to 61·22) | 1·67 (1·43 to 1·95) | <0·0001 | 1·19 (0·95 to 1·48) | 0·12 |
| Jehovah’s Witness | 485 (0·93%) | 64·74% (60·38 to 68·87) | 2·27 (1·88 to 2·73) | <0·0001 | 1·4 (1·11 to 1·76) | 0·0038 |
| Atheist | 5535 (10·63%) | 41·3% (40·01 to 42·60) | 0·87 (0·82 to 0·92) | <0·0001 | 0·93 (0·85 to 1·02) | 0·12 |
| Agnostic | 1759 (3·38%) | 39% (36·75 to 41·30) | 0·79 (0·72 to 0·87) | <0·0001 | 0·93 (0·82 to 1·05) | 0·24 |
| Buddhist | 289 (0·55%) | 61·59% (55·85 to 67·02) | 1·98 (1·56 to 2·51) | <0·0001 | 1·28 (0·96 to 1·71) | 0·097 |
| Hindu | 68 (0·13%) | 76·47% (64·98 to 85·06) | 4·01 (2·29 to 7·03) | <0·0001 | 2·18 (1·13 to 4·19) | 0·0197 |
| Other | 958 (1·84%) | 63·57% (60·47 to 66·56) | 2·15 (1·89 to 2·46) | <0·0001 | 1·42 (1·21 to 1·67) | <0·0001 |
| Prefer not to answer | 2750 (5·28%) | 56·22% (54·36 to 58·06) | 1·58 (1·47 to 1·71) | <0·0001 | 1·26 (1·14 to 1·4) | <0·0001 |
| **Importance of religion** |  | <0·0001 |  |  |  |  |
| Not at all (0) | 7461 (14·32%) | 42·7% (41·58 to 43·83) | Ref. |  | Ref. |  |
| Slightly (1–3) | 7176 (13·78%) | 46·39% (45·24 to 47·55) | 1·16 (1·09 to 1·24) | <0·0001 | 1·15 (1·05 to 1·26) | 0·0019 |
| Somewhat important (4–6) | 14352 (27·55%) | 52·78% (51·96 to 53·60) | 1·5 (1·42 to 1·59) | <0·0001 | 1·26 (1·16 to 1·38) | <0·0001 |
| Very (7–9) | 16192 (31·08%) | 40·63% (39·88 to 41·39) | 0·92 (0·87 to 0·97) | 0·0027 | 1·18 (1·08 to 1·29) | 0·00038 |
| Extremely (10) | 4331 (8·31%) | 43·18% (41·71 to 44·66) | 1·02 (0·95 to 1·1) | 0·62 | 1·08 (0·97 to 1·21) | 0·15 |
| Prefer not to answer | 2582 (4·96%) | 50·54% (48·61 to 52·47) | 1·37 (1·25 to 1·5) | <0·0001 | 1·58 (1·39 to 1·8) | <0·0001 |
| **Perceived NHS quality***** | 5·73 (2·10%) | - | 0·78 (0·77 to 0·79) | <0·0001 | 0·9 (0·89 to 0·92) | <0·0001 |
| **Perceived NHS access***** | 6·01 (2·14%) | - | 0·79 (0·79 to 0·8) | <0·0001 | 0·91 (0·9 to 0·92) | <0·0001 |
| **Block 6 – Survey mode** |  |  |  |  |  |  |
| **Survey mode** |  | <0·0001 |  |  |  |  |
| CAWI | 36644 (70·34%) | 50·74% (50·23 to 51·25) | Ref. |  | Ref. |  |
| CATI | 15450 (29·66%) | 33·99% (33·24 to 34·74) | 0·5 (0·48 to 0·52) | <0·0001 | 0·78 (0·73 to 0·83) | <0·0001 |

*p-value in this column were obtained via Chi-squared tests.

**The multivariable regression model reported in Table 1 was adjusted for all the variables presented in Tables 1 and 2 (Block 1-6).

***Variables expressed as mean and standard deviation in brackets. Group differences were assessed using independent samples t-tests (all comparisons: p<0.001).

Abbreviations: adjOR adjusted Odds Ratio, CATI Computer-Assisted Telephone Interviewing, CAWI Computer-Assisted Web Interviewing, CI Confidence Interval, NHS National Health Service, OR Odds Ratio.

Note: Regression estimates should not be interpreted as causal effects.

## Table S3. Comparison of unweighted and post-stratification weighted prevalence of vaccine hesitancy by study variables (percentage difference).

|  | **Prevalence of Vaccine Hesitancy** | |  |
| --- | --- | --- | --- |
|  | **Unweighted** | **Post-stratification weighting** | **Percentage difference** |
| **Block 1 - Sociodemographic and socioeconomic characteristics** |  |  |  |
| **Age group** |  |  |  |
| 18–29 | 45·18% | 45·59% | 0·90% |
| 30–44 | 53·48% | 53·60% | 0·22% |
| 45–59 | 49·55% | 49·60% | 0·10% |
| 60–74 | 42·02% | 42·07% | 0·12% |
| 75+ | 33·84% | 36·17% | 6·44% |
| **Gender** |  |  |  |
| Male | 44·82% | 44·96% | 0·31% |
| Female | 46·28% | 46·78% | 1·07% |
| Non-binary/Other | 67·36% | 66·76% | -0·90% |
| Prefer not to answer | 50·00% | 51·70% | 3·29% |
| **Marital status** |  |  |  |
| Single | 49·73% | 49·74% | 0·02% |
| Married | 42·94% | 43·31% | 0·85% |
| Separated/Divorced | 53·96% | 53·89% | -0·13% |
| Cohabiting | 51·43% | 51·71% | 0·54% |
| Widowed | 32·16% | 33·43% | 3·80% |
| **Children** |  |  |  |
| No children | 49·89% | 49·88% | -0·02% |
| Only children ≤11 years | 53·32% | 53·52% | 0·37% |
| Only children 12-18 years | 53·41% | 53·59% | 0·34% |
| Only children >18 years | 38·40% | 39·30% | 2·29% |
| Children of various ages | 46·84% | 46·88% | 0·09% |
| **Sexual orientation** |  |  |  |
| Heterosexual | 45·45% | 45·68% | 0·50% |
| Homosexual | 46·30% | 46·25% | -0·11% |
| Bisexual | 46·24% | 47·24% | 2·12% |
| Pansexual | 65·40% | 65·79% | 0·59% |
| Ace spectrum | 69·14% | 69·09% | -0·07% |
| Prefer not to answer | 44·12% | 45·67% | 3·39% |
| **Municipality size (inhabitants)** |  |  |  |
| ≤10,000 | 45·57% | 47·04% | 3·13% |
| 10,001–25,000 | 43·39% | 44·34% | 2·14% |
| 25,001–50,000 | 43·57% | 43·40% | -0·39% |
| 50,001–100,000 | 48·21% | 47·20% | -2·14% |
| 100,001–250,000 | 50·95% | 50·64% | -0·61% |
| >250,000 | 47·18% | 45·87% | -2·86% |
| **Geographic macro-area** |  |  |  |
| North-West | 46·27% | 46·28% | 0·02% |
| North-East | 45·02% | 45·65% | 1·38% |
| Centre | 44·40% | 44·78% | 0·85% |
| South | 47·29% | 47·51% | 0·46% |
| Islands | 45·25% | 45·87% | 1·35% |
| **Degree of urbanisation** |  |  |  |
| Pole | 47·28% | 46·53% | -1·61% |
| Intermunicipal pole | 44·07% | 44·41% | 0·77% |
| Belt | 45·94% | 46·51% | 1·23% |
| Intermediate | 44·41% | 45·76% | 2·95% |
| Peripheral | 41·64% | 43·33% | 3·90% |
| Ultra-peripheral | 44·17% | 45·91% | 3·79% |
| **Education level** |  |  |  |
| Upper secondary | 48·21% | 48·49% | 0·58% |
| Primary/None | 32·44% | 33·36% | 2·76% |
| Lower secondary | 47·01% | 48·21% | 2·49% |
| University | 42·57% | 42·24% | -0·78% |
| Postgraduate | 40·72% | 40·77% | 0·12% |
| **Occupational status** |  |  |  |
| Non-healthcare worker | 49·35% | 49·41% | 0·12% |
| Healthcare worker | 42·64% | 42·56% | -0·19% |
| Homemaker | 52·58% | 52·69% | 0·21% |
| Retired | 35·81% | 36·72% | 2·48% |
| Student (non-health field) | 38·72% | 38·83% | 0·28% |
| Student (health field) | 34·98% | 35·86% | 2·45% |
| Job seeker | 55·45% | 55·73% | 0·50% |
| Unemployed | 58·12% | 58·10% | -0·03% |
| Other | 44·59% | 45·88% | 2·81% |
| **Continent of citizenship** |  |  |  |
| Italy | 45·72% | 46·05% | 0·72% |
| Europe (non-Italy) | 49·41% | 49·01% | -0·82% |
| Africa | 51·06% | 50·52% | -1·07% |
| America | 45·33% | 45·53% | 0·44% |
| Asia | 42·45% | 44·41% | 4·41% |
| Oceania | 100·00% | 100·00% | 0·00% |
| **Self-identified ethnicity** |  |  |  |
| European | 45·28% | 45·60% | 0·70% |
| Multi-ethnic | 65·99% | 65·65% | -0·52% |
| North American / Australian | 71·25% | 70·47% | -1·11% |
| Arab-Middle Eastern | 66·22% | 64·85% | -2·11% |
| North African | 58·30% | 57·48% | -1·43% |
| Latino-American | 54·76% | 55·22% | 0·83% |
| African American | 73·68% | 78·63% | 6·30% |
| Black African | 48·42% | 47·95% | -0·98% |
| Asian | 50·60% | 51·66% | 2·05% |
| Pacific Islands | 50·00% | 51·75% | 3·38% |
| **Material deprivation** |  |  |  |
| No deprivation | 45·47% | 45·80% | 0·72% |
| Severe deprivation | 52·62% | 52·54% | -0·15% |
| **Block 2 - Health-related characteristics and personal experience** |  |  |  |
| **Chronic conditions** |  |  |  |
| No chronic disease | 46·65% | 46·95% | 0·64% |
| One chronic disease | 46·80% | 47·03% | 0·49% |
| More than one chronic disease | 41·48% | 41·94% | 1·10% |
| **Living with a person with disability** |  |  |  |
| No | 44·68% | 44·92% | 0·53% |
| Yes | 50·91% | 51·64% | 1·41% |
| **Inadequate health literacy** |  |  |  |
| No | 44·16% | 44·21% | 0·11% |
| Yes | 48·11% | 48·91% | 1·64% |
| **Knowing someone who had AEFI** |  |  |  |
| No | 35·88% | 36·06% | 0·50% |
| Yes | 66·90% | 67·53% | 0·93% |
| **Knowing someone who had VPD** |  |  |  |
| No | 46·44% | 46·71% | 0·58% |
| Yes | 43·49% | 43·96% | 1·07% |
| **Reported barriers to vaccination** |  |  |  |
| No | 37·92% | 37·97% | 0·13% |
| Yes | 45·40% | 55·47% | 18·15% |
| **Block 3 - Information sources and trust** |  |  |  |
| **Information source cluster** |  |  |  |
| Diversified sources | 53·10% | 53·45% | 0·65% |
| Professional-only sources | 33·37% | 33·67% | 0·89% |
| **Trust in sources** | - | - | - |
| **Block 4 – External influences (perceived vaccination endorsement in the respondent’s community by:)** |  |  |  |
| **By religious leaders** |  |  |  |
| Yes | 40·96% | 40·68% | -0·69% |
| No | 61·91% | 62·43% | 0·83% |
| Don’t know | 42·38% | 42·94% | 1·30% |
| **By political leaders** |  |  |  |
| Yes | 42·51% | 42·40% | -0·26% |
| No | 63·13% | 63·49% | 0·57% |
| Don’t know | 41·67% | 42·34% | 1·58% |
| **By teachers** |  |  |  |
| Yes | 38·05% | 38·13% | 0·21% |
| No | 67·67% | 67·68% | 0·01% |
| Don’t know | 46·30% | 46·95% | 1·38% |
| **By health professionals** |  |  |  |
| Yes | 37·67% | 37·72% | 0·13% |
| No | 69·91% | 69·96% | 0·07% |
| Don’t know | 53·68% | 54·68% | 1·83% |
| **Block 5 – Beliefs and attitudes** |  |  |  |
| **Use of non-conventional medicine** |  |  |  |
| No | 40·35% | 40·63% | 0·69% |
| Yes, integrated with conventional medicine | 51·75% | 52·03% | 0·54% |
| Yes, as alternative to conventional medicine | 70·79% | 71·09% | 0·42% |
| **Political orientation** |  |  |  |
| Right (7–9) | 47·05% | 47·72% | 1·40% |
| Centre (4–6) | 50·67% | 50·81% | 0·28% |
| Extreme left (0) | 44·76% | 44·35% | -0·92% |
| Left (1–3) | 33·89% | 34·00% | 0·32% |
| Extreme right (10) | 52·08% | 52·52% | 0·84% |
| Non-aligned with traditional parties | 49·31% | 50·22% | 1·81% |
| Prefer not to answer | 34·02% | 33·80% | -0·65% |
| **Religion** |  |  |  |
| Catholic | 44·76% | 45·03% | 0·60% |
| Orthodox | 40·72% | 42·47% | 4·12% |
| Protestant | 64·40% | 62·82% | -2·52% |
| Jewish | 72·46% | 69·86% | -3·72% |
| Muslim | 57·53% | 58·07% | 0·93% |
| Jehovah’s Witness | 64·74% | 66·30% | 2·35% |
| Atheist | 41·30% | 41·53% | 0·55% |
| Agnostic | 39·00% | 39·21% | 0·54% |
| Buddhist | 61·59% | 62·54% | 1·52% |
| Hindu | 76·47% | 76·57% | 0·13% |
| Other | 63·57% | 63·35% | -0·35% |
| Prefer not to answer | 56·22% | 56·52% | 0·53% |
| **Importance of religion** |  |  |  |
| Not at all (0) | 42·70% | 42·63% | -0·16% |
| Slightly (1–3) | 46·39% | 46·23% | -0·35% |
| Somewhat important (4–6) | 52·78% | 52·98% | 0·38% |
| Very (7–9) | 40·63% | 41·52% | 2·14% |
| Extremely (10) | 43·18% | 42·97% | -0·49% |
| Prefer not to answer | 50·54% | 50·42% | -0·24% |
| **Perceived NHS quality** | - | - | - |
| **Perceived NHS access** | - | - | - |
| **Block 6 – Survey mode** |  |  |  |
| **Survey mode** |  |  |  |
| CAWI | 50·74% | 50·61% | -0·26% |
| CATI | 33·99% | 34·79% | 2·30% |

Abbreviations: CATI Computer Assisted Telephone Interviewing, CAWI Computer Assisted Web Interviewing, NHS National Health Service, VPD Vaccine Preventable Disease.

## Table S4. Unweighted analysis: Hierarchical logistic regression model for vaccine hesitancy

| **Variable** | **BLOCK 1** | | **BLOCK 2** | | **BLOCK 3** | | **BLOCK 4** | | **BLOCK 5** | | **BLOCK 6** | | **BLOCK 7 - sensitivity analysis** | | |
| --- | --- | --- | --- | --- | --- | --- | --- | --- | --- | --- | --- | --- | --- | --- | --- |
|  | **adjOR**  **(95% CI)** | **p** | **adjOR**  **(95% CI)** | **p** | **adjOR**  **(95% CI)** | **p** | **adjOR**  **(95% CI)** | **p** | **adjOR**  **(95% CI)** | **p** | **adjOR**  **(95% CI)** | **p** | **adjOR**  **(95% CI)** | **p** |  |
| Age group |  |  |  |  |  |  |  |  |  |  |  |  |  |  |  |
| 18–29 | Ref. |  | Ref. |  | Ref. |  | Ref. |  | Ref. |  | Ref. |  | Ref. |  |  |
| 30–44 | 1·32 (1·23;1·41) | <0·0001 | 1·48 (1·37;1·59) | <0·0001 | 1·47 (1·36;1·58) | <0·0001 | 1·47 (1·36;1·59) | <0·0001 | 1·47 (1·36;1·6) | <0·0001 | 1·47 (1·35;1·59) | <0·0001 | 1·36 (1·25;1·49) | <0·0001 |  |
| 45–59 | 1·24 (1·16;1·34) | <0·0001 | 1·51 (1·4;1·64) | <0·0001 | 1·56 (1·44;1·69) | <0·0001 | 1·56 (1·44;1·7) | <0·0001 | 1·59 (1·46;1·74) | <0·0001 | 1·58 (1·45;1·72) | <0·0001 | 1·41 (1·29;1·55) | <0·0001 |  |
| 60–74 | 1·34 (1·22;1·46) | <0·0001 | 1·67 (1·51;1·84) | <0·0001 | 1·76 (1·59;1·94) | <0·0001 | 1·73 (1·56;1·92) | <0·0001 | 1·78 (1·6;1·98) | <0·0001 | 1·79 (1·61;1·99) | <0·0001 | 1·51 (1·34;1·7) | <0·0001 |  |
| 75+ | 1·09 (0·98;1·21) | 0·12 | 1·27 (1·13;1·42) | <0·0001 | 1·29 (1·15;1·45) | <0·0001 | 1·3 (1·15;1·46) | <0·0001 | 1·33 (1·18;1·51) | <0·0001 | 1·41 (1·24;1·6) | <0·0001 | 1·65 (1·44;1·9) | <0·0001 |  |
| Gender |  |  |  |  |  |  |  |  |  |  |  |  |  |  |  |
| Male | Ref. |  | Ref. |  | Ref. |  | Ref. |  | Ref. |  | Ref. |  | Ref. |  |  |
| Female | 1·05 (1·01;1·09) | 0·015 | 1·03 (0·99;1·07) | 0·15 | 1·04 (1;1·09) | 0·061 | 1·06 (1·02;1·11) | 0·0042 | 1·01 (0·97;1·06) | 0·52 | 1·01 (0·97;1·06) | 0·56 | 1·04 (0·99;1·1) | 0·081 |  |
| Non-binary/Other | 2·42 (1·96;2·98) | <0·0001 | 2·3 (1·85;2·87) | <0·0001 | 2·2 (1·75;2·77) | <0·0001 | 2·3 (1·82;2·9) | <0·0001 | 2·11 (1·66;2·68) | <0·0001 | 2·12 (1·67;2·69) | <0·0001 | 1·68 (1·29;2·18) | 0·00012 |  |
| Prefer not to answer | 1·16 (0·58;2·32) | 0·67 | 1·36 (0·64;2·87) | 0·43 | 1·23 (0·56;2·7) | 0·605 | 1·17 (0·53;2·6) | 0·7001 | 1·06 (0·47;2·38) | 0·88 | 1·03 (0·46;2·29) | 0·95 | 0·98 (0·42;2·28) | 0·96 |  |
| Marital status |  |  |  |  |  |  |  |  |  |  |  |  |  |  |  |
| Single | Ref. |  | Ref. |  | Ref. |  | Ref. |  | Ref. |  | Ref. |  | Ref. |  |  |
| Married | 0·89 (0·84;0·95) | 0·00022 | 0·89 (0·83;0·95) | 0·00030 | 0·93 (0·87;1) | 0·036 | 0·92 (0·86;0·99) | 0·019 | 0·89 (0·83;0·95) | 0·0010 | 0·9 (0·84;0·97) | 0·0033 | 0·9 (0·83;0·98) | 0·011 |  |
| Separated/Divorced | 1·28 (1·18;1·4) | <0·0001 | 1·24 (1·13;1·36) | <0·0001 | 1·22 (1·11;1·35) | <0·0001 | 1·22 (1·1;1·35) | <0·0001 | 1·12 (1·01;1·24) | 0·034 | 1·12 (1·01;1·24) | 0·032 | 1·08 (0·96;1·21) | 0·19 |  |
| Cohabiting | 1·03 (0·96;1·1) | 0·44 | 0·99 (0·92;1·07) | 0·81 | 1·03 (0·96;1·11) | 0·43 | 1·05 (0·97;1·13) | 0·25 | 1·03 (0·95;1·12) | 0·43 | 1·04 (0·96;1·12) | 0·39 | 1·02 (0·93;1·11) | 0·697 |  |
| Widowed | 0·77 (0·69;0·86) | <0·0001 | 0·75 (0·67;0·85) | <0·0001 | 0·81 (0·72;0·91) | 0·00051 | 0·78 (0·69;0·88) | <0·0001 | 0·75 (0·66;0·85) | <0·0001 | 0·76 (0·67;0·86) | <0·0001 | 0·77 (0·67;0·89) | 0·00024 |  |
| Children |  |  |  |  |  |  |  |  |  |  |  |  |  |  |  |
| No children | Ref. |  | Ref. |  | Ref. |  | Ref. |  | Ref. |  | Ref. |  | Ref. |  |  |
| Only children <11 years | 1·09 (1·02;1·17) | 0·0083 | 1·01 (0·94;1·09) | 0·6997 | 0·98 (0·91;1·06) | 0·64 | 0·96 (0·89;1·04) | 0·306 | 0·95 (0·87;1·02) | 0·16 | 0·93 (0·86;1·01) | 0·091 | 0·93 (0·85;1·01) | 0·088 |  |
| Only adolescents | 1·05 (0·97;1·14) | 0·24 | 0·93 (0·85;1·01) | 0·09001 | 0·93 (0·85;1·02) | 0·105 | 0·92 (0·84;1·01) | 0·0696 | 0·9 (0·82;0·99) | 0·028 | 0·89 (0·81;0·98) | 0·017 | 0·83 (0·75;0·92) | 0·00068 |  |
| Only adults | 0·68 (0·64;0·72) | <0·0001 | 0·67 (0·63;0·72) | <0·0001 | 0·73 (0·68;0·78) | <0·0001 | 0·75 (0·7;0·81) | <0·0001 | 0·76 (0·71;0·82) | <0·0001 | 0·78 (0·72;0·84) | <0·0001 | 0·75 (0·69;0·81) | <0·0001 |  |
| Children of various ages | 0·82 (0·74;0·89) | <0·0001 | 0·74 (0·67;0·82) | <0·0001 | 0·76 (0·69;0·85) | <0·0001 | 0·76 (0·68;0·84) | <0·0001 | 0·77 (0·69;0·86) | <0·0001 | 0·76 (0·68;0·84) | <0·0001 | 0·73 (0·64;0·82) | <0·0001 |  |
| Sexual orientation |  |  |  |  |  |  |  |  |  |  |  |  |  |  |  |
| Heterosexual | Ref. |  | Ref. |  | Ref. |  | Ref. |  | Ref. |  | Ref. |  | Ref. |  |  |
| Homosexual | 0·86 (0·75;0·99) | 0·042 | 0·81 (0·7;0·95) | 0·0072 | 0·77 (0·65;0·9) | 0·00083 | 0·75 (0·64;0·89) | 0·00061 | 0·79 (0·67;0·93) | 0·0053 | 0·8 (0·67;0·94) | 0·0074 | 0·83 (0·69;1) | 0·045 |  |
| Bisexual | 0·84 (0·74;0·95) | 0·0067 | 0·8 (0·7;0·91) | 0·00094 | 0·76 (0·66;0·87) | 0·00013 | 0·75 (0·65;0·87) | 0·00013 | 0·78 (0·67;0·9) | 0·0012 | 0·77 (0·66;0·89) | 0·00067 | 0·86 (0·73;1·02) | 0·081 |  |
| Pansexual | 1·58 (1·26;1·98) | <0·0001 | 1·41 (1·11;1·79) | 0·0056 | 1·16 (0·9;1·49) | 0·26 | 1·03 (0·8;1·34) | 0·797 | 0·93 (0·71;1·23) | 0·62 | 0·92 (0·7;1·21) | 0·55 | 1·06 (0·79;1·43) | 0·68 |  |
| Ace spectrum | 1·88 (1·53;2·31) | <0·0001 | 1·59 (1·28;1·97) | <0·0001 | 1·27 (1·02;1·59) | 0·036 | 1·1 (0·88;1·39) | 0·396 | 0·9 (0·71;1·15) | 0·41 | 0·88 (0·69;1·12) | 0·303 | 1·03 (0·8;1·33) | 0·81 |  |
| Prefer not to answer | 1·03 (0·95;1·12) | 0·44 | 0·91 (0·83;0·99) | 0·026 | 0·65 (0·59;0·71) | <0·0001 | 0·62 (0·56;0·68) | <0·001 | 0·63 (0·57;0·7) | <0·0001 | 0·65 (0·59;0·72) | <0·0001 | 0·67 (0·6;0·75) | <0·0001 |  |
| Municipality size (inhabitants) |  |  |  |  |  |  |  |  |  |  |  |  |  |  |  |
| ≤10,000 | Ref. |  | Ref. |  | Ref. |  | Ref. |  | Ref. |  | Ref. |  | Ref. |  |  |
| 10,001–25,000 | 0·9 (0·85;0·95) | <0·0001 | 0·92 (0·87;0·97) | 0·0021 | 0·95 (0·89;1) | 0·056 | 0·95 (0·89;1) | 0·061 | 0·98 (0·93;1·05) | 0·62 | 0·98 (0·92;1·04) | 0·51 | 0·99 (0·93;1·06) | 0·87 |  |
| 25,001–50,000 | 0·91 (0·85;0·97) | 0·0026 | 0·93 (0·87;1) | 0·048 | 0·96 (0·89;1·03) | 0·23 | 0·95 (0·89;1·03) | 0·198 | 0·99 (0·92;1·07) | 0·89 | 0·98 (0·91;1·06) | 0·63 | 0·97 (0·9;1·06) | 0·52 |  |
| 50,001–100,000 | 1·08 (0·99;1·18) | 0·079 | 1·11 (1·01;1·21) | 0·032 | 1·11 (1·01;1·22) | 0·038 | 1·07 (0·97;1·19) | 0·15 | 1·08 (0·98;1·19) | 0·14 | 1·06 (0·96;1·17) | 0·28 | 1·05 (0·94;1·18) | 0·36 |  |
| 100,001–250,000 | 1·27 (1·14;1·41) | <0·0001 | 1·23 (1·1;1·37) | 0·00031 | 1·16 (1·03;1·3) | 0·015 | 1·11 (0·98;1·25) | 0·088 | 1·12 (0·99;1·27) | 0·065 | 1·09 (0·96;1·23) | 0·17 | 1·16 (1·01;1·33) | 0·031 |  |
| >250,000 | 1·07 (0·97;1·17) | 0·18 | 1·05 (0·95;1·16) | 0·37 | 1·03 (0·92;1·14) | 0·64 | 1·01 (0·9;1·12) | 0·91 | 1·02 (0·92;1·14) | 0·68 | 0·99 (0·88;1·1) | 0·82 | 0·98 (0·86;1·11) | 0·72 |  |
| Geographic macro-area |  |  |  |  |  |  |  |  |  |  |  |  |  |  |  |
| North-West | Ref. |  | Ref. |  | Ref. |  | Ref. |  | Ref. |  | Ref. |  | Ref. |  |  |
| North-East | 0·94 (0·89;0·99) | 0·026 | 0·9 (0·85;0·95) | 0·00031 | 0·94 (0·88;0·99) | 0·033 | 0·93 (0·87;0·99) | 0·016 | 1 (0·94;1·07) | 0·93 | 1·02 (0·96;1·09) | 0·52 | 1 (0·93;1·07) | 0·996 |  |
| Centre | 0·94 (0·89;0·99) | 0·0302 | 0·93 (0·88;0·99) | 0·0202 | 0·96 (0·9;1·02) | 0·18 | 0·97 (0·92;1·03) | 0·36 | 0·98 (0·92;1·04) | 0·45 | 1 (0·94;1·07) | 0·96 | 1·03 (0·96;1·11) | 0·399 |  |
| South | 1·05 (0·99;1·11) | 0·077 | 0·95 (0·9;1) | 0·065 | 0·94 (0·89;1) | 0·039 | 0·96 (0·9;1·02) | 0·16 | 0·92 (0·86;0·98) | 0·0096 | 0·92 (0·86;0·98) | 0·0093 | 0·89 (0·83;0·96) | 0·0017 |  |
| Islands | 0·97 (0·9;1·03) | 0·31 | 0·92 (0·86;0·99) | 0·024 | 0·92 (0·86;0·99) | 0·034 | 0·92 (0·85;1) | 0·038 | 0·86 (0·79;0·93) | 0·00015 | 0·86 (0·79;0·93) | 0·00013 | 0·83 (0·76;0·91) | <0·0001 |  |
| Degree of urbanization |  |  |  |  |  |  |  |  |  |  |  |  |  |  |  |
| A-Pole | Ref. |  | Ref. |  | Ref. |  | Ref. |  | Ref. |  | Ref. |  | Ref. |  |  |
| B-Intermunicipal pole | 0·99 (0·87;1·12) | 0·88 | 0·98 (0·86;1·12) | 0·76 | 0·98 (0·86;1·13) | 0·801 | 1 (0·87;1·15) | 0·99 | 0·96 (0·83;1·11) | 0·61 | 0·96 (0·83;1·11) | 0·58 | 0·98 (0·83;1·15) | 0·78 |  |
| C-Belt | 1·14 (1·05;1·23) | 0·0012 | 1·12 (1·03;1·21) | 0·0065 | 1·1 (1·01;1·2) | 0·027 | 1·11 (1·02;1·21) | 0·021 | 1·09 (1;1·19) | 0·051 | 1·09 (0·99;1·19) | 0·068 | 1·13 (1·02;1·25) | 0·016 |  |
| D-Intermediate | 1·11 (1·01;1·21) | 0·024 | 1·09 (0·99;1·2) | 0·066 | 1·1 (1;1·21) | 0·051 | 1·08 (0·98;1·19) | 0·13 | 1·07 (0·97;1·19) | 0·17 | 1·08 (0·98;1·2) | 0·14 | 1·19 (1·06;1·33) | 0·0027 |  |
| E-Peripheral | 0·99 (0·9;1·1) | 0·86 | 0·99 (0·89;1·1) | 0·796 | 1 (0·89;1·11) | 0·95 | 1 (0·89;1·12) | 0·98 | 0·97 (0·86;1·09) | 0·63 | 0·98 (0·87;1·1) | 0·76 | 1·04 (0·92;1·19) | 0·52 |  |
| F-Ultra-peripheral | 1·04 (0·88;1·24) | 0·63 | 1·07 (0·89;1·28) | 0·49 | 1·07 (0·88;1·3) | 0·49 | 1·04 (0·85;1·26) | 0·72 | 0·96 (0·78;1·17) | 0·67 | 0·97 (0·8;1·19) | 0·78 | 0·98 (0·78;1·23) | 0·88 |  |
| Education level |  |  |  |  |  |  |  |  |  |  |  |  |  |  |  |
| Upper secondary | Ref. |  | Ref. |  | Ref. |  | Ref. |  | Ref. |  | Ref. |  | Ref. |  |  |
| Primary/None | 0·72 (0·64;0·81) | <0·0001 | 0·65 (0·57;0·74) | <0·0001 | 0·64 (0·55;0·73) | <0·0001 | 0·62 (0·54;0·71) | <0·0001 | 0·55 (0·48;0·64) | <0·0001 | 0·57 (0·49;0·66) | <0·0001 | 0·56 (0·48;0·66) | <0·0001 |  |
| Lower secondary | 1·12 (1·06;1·18) | <0·0001 | 1·12 (1·06;1·19) | 0·00014 | 1·09 (1·03;1·16) | 0·0041 | 1·06 (1;1·13) | 0·068 | 1·04 (0·97;1·1) | 0·26 | 1·05 (0·99;1·12) | 0·12 | 0·99 (0·92;1·06) | 0·75 |  |
| University | 0·73 (0·7;0·76) | <0·0001 | 0·75 (0·71;0·78) | <0·0001 | 0·79 (0·75;0·83) | <0·0001 | 0·81 (0·77;0·86) | <0·0001 | 0·84 (0·8;0·89) | <0·0001 | 0·84 (0·8;0·89) | <0·0001 | 0·89 (0·84;0·94) | 0·00011 |  |
| Postgraduate | 0·64 (0·59;0·69) | <0·0001 | 0·6 (0·55;0·65) | <0·0001 | 0·65 (0·6;0·71) | <0·0001 | 0·67 (0·62;0·74) | <0·0001 | 0·71 (0·65;0·78) | <0·0001 | 0·7 (0·64;0·77) | <0·0001 | 0·73 (0·66;0·81) | <0·0001 |  |
| Occupational status |  |  |  |  |  |  |  |  |  |  |  |  |  |  |  |
| Non-healthcare worker | Ref. |  | Ref. |  | Ref. |  | Ref. |  | Ref. |  | Ref. |  | Ref. |  |  |
| Healthcare worker | 0·78 (0·71;0·85) | <0·0001 | 0·72 (0·66;0·79) | <0·0001 | 0·72 (0·65;0·79) | <0·0001 | 0·72 (0·65;0·8) | <0·0001 | 0·71 (0·64;0·79) | <0·0001 | 0·71 (0·64;0·78) | <0·0001 | 0·75 (0·67;0·84) | <0·0001 |  |
| Homemaker | 1·09 (1·01;1·18) | 0·019 | 1·16 (1·08;1·26) | 0·00013 | 1·11 (1·03;1·21) | 0·011 | 1·11 (1·02;1·21) | 0·013 | 1·11 (1·02;1·21) | 0·018 | 1·1 (1·01;1·2) | 0·0305 | 0·99 (0·9;1·09) | 0·91 |  |
| Retired | 0·73 (0·68;0·78) | <0·0001 | 0·75 (0·69;0·8) | <0·0001 | 0·71 (0·66;0·77) | <0·0001 | 0·75 (0·69;0·81) | <0·0001 | 0·79 (0·73;0·86) | <0·0001 | 0·83 (0·77;0·91) | <0·0001 | 1 (0·91;1·1) | 0·98 |  |
| Student (non-health field) | 0·62 (0·56;0·69) | <0·0001 | 0·63 (0·56;0·71) | <0·0001 | 0·59 (0·53;0·67) | <0·0001 | 0·62 (0·55;0·7) | <0·0001 | 0·66 (0·58;0·74) | <0·0001 | 0·65 (0·58;0·74) | <0·0001 | 0·74 (0·64;0·84) | <0·0001 |  |
| Student (health field) | 0·52 (0·45;0·61) | <0·0001 | 0·52 (0·44;0·61) | <0·0001 | 0·5 (0·42;0·6) | <0·0001 | 0·52 (0·43;0·62) | <0·0001 | 0·54 (0·45;0·65) | <0·0001 | 0·54 (0·45;0·65) | <0·0001 | 0·64 (0·52;0·78) | <0·0001 |  |
| Job seeker | 1·14 (1·03;1·26) | 0·01001 | 1·12 (1·01;1·24) | 0·039 | 1·03 (0·92;1·15) | 0·58 | 1·05 (0·94;1·18) | 0·36 | 1·03 (0·92;1·16) | 0·603 | 1·02 (0·91;1·14) | 0·75 | 1·02 (0·9;1·16) | 0·74 |  |
| Unemployed | 1·22 (1·11;1·33) | <0·0001 | 1·24 (1·13;1·37) | <0·0001 | 1·09 (0·98;1·2) | 0·099 | 1·07 (0·96;1·18) | 0·22 | 1·04 (0·93;1·15) | 0·51 | 1·02 (0·92;1·14) | 0·67 | 0·99 (0·89;1·12) | 0·92 |  |
| Other | 0·73 (0·46;1·16) | 0·19 | 0·81 (0·49;1·33) | 0·402 | 0·83 (0·5;1·38) | 0·47 | 0·85 (0·51;1·43) | 0·55 | 0·72 (0·42;1·24) | 0·23 | 0·73 (0·42;1·25) | 0·25 | 0·76 (0·42;1·36) | 0·35 |  |
| Continent of citizenship |  |  |  |  |  |  |  |  |  |  |  |  |  |  |  |
| Italy | Ref. |  | Ref. |  | Ref. |  | Ref. |  | Ref. |  | Ref. |  | Ref. |  |  |
| Europe (non-Italy) | 0·98 (0·83;1·16) | 0·78 | 0·93 (0·78;1·12) | 0·45 | 0·93 (0·78;1·12) | 0·46 | 0·94 (0·78;1·13) | 0·49 | 1·01 (0·83;1·23) | 0·91 | 1·01 (0·83;1·24) | 0·899 | 1·09 (0·87;1·36) | 0·47 |  |
| Africa | 0·67 (0·45;1) | 0·047 | 0·74 (0·49;1·12) | 0·16 | 0·92 (0·59;1·41) | 0·69 | 1·05 (0·68;1·63) | 0·81 | 1·15 (0·73;1·81) | 0·55 | 1·16 (0·74;1·83) | 0·5210 | 1·55 (0·95;2·51) | 0·079 |  |
| America | 0·58 (0·39;0·86) | 0·0064 | 0·64 (0·42;0·97) | 0·037 | 0·72 (0·47;1·11) | 0·13 | 0·77 (0·5;1·2) | 0·25 | 0·86 (0·55;1·36) | 0·52 | 0·86 (0·54;1·35) | 0·51 | 1·15 (0·68;1·94) | 0·61 |  |
| Asia | 0·56 (0·35;0·91) | 0·019 | 0·61 (0·37;1·01) | 0·056 | 0·71 (0·42;1·21) | 0·21 | 0·84 (0·49;1·46) | 0·54 | 0·95 (0·54;1·65) | 0·84 | 0·95 (0·54;1·66) | 0·85 | 0·86 (0·48;1·55) | 0·62 |  |
| Oceania | 1 (0;0) |  | 1 (0;0) |  | 1 (0;0) |  | 1 (0;0) |  | 1 (0;0) |  | 1 (0;0) |  | 1 (0;0) |  |  |
| Self-identified ethnicity |  |  |  |  |  |  |  |  |  |  |  |  |  |  |  |
| European | Ref. |  | Ref. |  | Ref. |  | Ref. |  | Ref. |  | Ref. |  | Ref. |  |  |
| Multi-ethnic | 2·1 (1·68;2·62) | <0·0001 | 1·92 (1·52;2·42) | <0·0001 | 1·7 (1·33;2·17) | <0·0001 | 1·57 (1·22;2·02) | 0·00046 | 1·37 (1·05;1·78) | 0·021 | 1·35 (1·04;1·76) | 0·026 | 1·32 (1;1·75) | 0·052 |  |
| North American / Australian | 2·43 (1·7;3·48) | <0·0001 | 2·15 (1·48;3·14) | <0·0001 | 1·85 (1·24;2·76) | 0·0027 | 1·61 (1·07;2·43) | 0·022 | 1·44 (0·95;2·21) | 0·088 | 1·44 (0·95;2·2) | 0·087 | 1·31 (0·83;2·04) | 0·24 |  |
| Arab-Middle Eastern | 2·13 (1·58;2·88) | <0·001 | 1·79 (1·31;2·44) | 0·00026 | 1·62 (1·17;2·26) | 0·00402 | 1·36 (0·97;1·91) | 0·077 | 1·07 (0·75;1·52) | 0·72 | 1·05 (0·74;1·51) | 0·78 | 1·04 (0·71;1·51) | 0·84 |  |
| North African | 1·6 (1·2;2·13) | 0·0013 | 1·73 (1·28;2·34) | 0·00037 | 1·74 (1·26;2·4) | 0·00072 | 1·53 (1·1;2·12) | 0·011 | 1·29 (0·9;1·84) | 0·16 | 1·31 (0·92;1·87) | 0·14 | 1·2 (0·82;1·76) | 0·34 |  |
| Latino-American | 1·35 (1·04;1·74) | 0·023 | 1·23 (0·93;1·62) | 0·14 | 1·09 (0·82;1·46) | 0·54 | 1·01 (0·75;1·36) | 0·95 | 0·88 (0·65;1·19) | 0·41 | 0·89 (0·65;1·21) | 0·45 | 0·81 (0·57;1·14) | 0·22 |  |
| African American | 2·41 (1·15;5·08) | 0·0204 | 2·14 (0·98;4·67) | 0·056 | 2·1 (0·93;4·78) | 0·075 | 1·66 (0·71;3·86) | 0·24 | 1·15 (0·48;2·75) | 0·75 | 1·15 (0·48;2·77) | 0·75 | 1·01 (0·41;2·5) | 0·98 |  |
| Black African | 1·13 (0·72;1·78) | 0·597 | 1·12 (0·69;1·8) | 0·65 | 1·11 (0·68;1·8) | 0·69 | 1·05 (0·64;1·73) | 0·84 | 0·85 (0·5;1·42) | 0·53 | 0·84 (0·5;1·42) | 0·52 | 0·77 (0·44;1·34) | 0·35 |  |
| Asian | 1·38 (0·94;2·04) | 0·10 | 1·31 (0·87;1·97) | 0·19997 | 1·21 (0·79;1·85) | 0·39 | 1·19 (0·77;1·84) | 0·44 | 0·94 (0·6;1·47) | 0·79 | 0·94 (0·6;1·48) | 0·79 | 1·1 (0·67;1·78) | 0·71 |  |
| Pacific Islands | 0·94 (0·51;1·71) | 0·83 | 0·9 (0·48;1·69) | 0·74 | 0·84 (0·42;1·68) | 0·62 | 0·7 (0·34;1·44) | 0·34 | 0·58 (0·27;1·26) | 0·17 | 0·57 (0·26;1·23) | 0·15 | 0·46 (0·21;1·02) | 0·057 |  |
| Material deprivation |  |  |  |  |  |  |  |  |  |  |  |  |  |  |  |
| No deprivation | Ref. |  | Ref. |  | Ref. |  | Ref. |  | Ref. |  | Ref. |  | Ref. |  |  |
| Severe deprivation | 1·07 (0·98;1·17) | 0·14 | 1·01 (0·92;1·11) | 0·87 | 1·07 (0·97;1·18) | 0·18 | 1·11 (1;1·23) | 0·047 | 1·03 (0·93;1·14) | 0·59 | 1·01 (0·91;1·13) | 0·79 | 0·89 (0·79;1) | 0·0495 |  |
| Chronic conditions |  |  |  |  |  |  |  |  |  |  |  |  |  |  |  |
| No chronic disease |  |  | Ref. |  | Ref. |  | Ref. |  | Ref. |  | Ref. |  | Ref. |  |  |
| One chronic disease |  |  | 0·96 (0·92;1·01) | 0·095 | 0·97 (0·92;1·02) | 0·19 | 0·97 (0·93;1·02) | 0·28 | 0·96 (0·92;1·01) | 0·15 | 0·95 (0·91;1) | 0·067 | 0·96 (0·91;1·02) | 0·18 |  |
| More than one chronic disease |  |  | 0·86 (0·81;0·91) | <0·0001 | 0·83 (0·79;0·89) | <0·0001 | 0·87 (0·81;0·92) | <0·0001 | 0·86 (0·81;0·91) | <0·0001 | 0·86 (0·81;0·92) | <0·0001 | 0·82 (0·76;0·88) | <0·0001 |  |
| Living with a person with disability |  |  |  |  |  |  |  |  |  |  |  |  |  |  |  |
| No |  |  | Ref. |  | Ref. |  | Ref. |  | Ref. |  | Ref. |  | Ref. |  |  |
| Yes |  |  | 1·05 (0·99;1·1) | 0·093 | 1 (0·95;1·05) | 0·98 | 1·02 (0·96;1·08) | 0·496 | 1 (0·95;1·06) | 0·91 | 1 (0·95;1·06) | 0·91 | 1·03 (0·97;1·1) | 0·32 |  |
| Inadequate health literacy |  |  |  |  |  |  |  |  |  |  |  |  |  |  |  |
| No |  |  | Ref. |  | Ref. |  | Ref. |  | Ref. |  | Ref. |  | Ref. |  |  |
| Yes |  |  | 1·19 (1·14;1·24) | <0·0001 | 1·21 (1·16;1·26) | <0·0001 | 1·14 (1·09;1·19) | <0·0001 | 1·13 (1·08;1·18) | <0·0001 | 1·13 (1·09;1·19) | <0·0001 | 1·06 (1·01;1·12) | 0·012 |  |
| Knowing someone who had vaccine adverse reaction |  |  |  |  |  |  |  |  |  |  |  |  |  |  |  |
| No |  |  | Ref. |  | Ref. |  | Ref. |  | Ref. |  | Ref. |  | Ref. |  |  |
| Yes |  |  | 4·08 (3·9;4·26) | <0·0001 | 3·69 (3·52;3·87) | <0·0001 | 3·74 (3·56;3·92) | <0·0001 | 3·38 (3·22;3·55) | <0·0001 | 3·37 (3·21;3·54) | <0·0001 | 1·94 (1·84;2·05) | <0·0001 |  |
| Knowing someone who had VPD |  |  |  |  |  |  |  |  |  |  |  |  |  |  |  |
| No |  |  | Ref. |  | Ref. |  | Ref. |  | Ref. |  | Ref. |  | Ref. |  |  |
| Yes |  |  | 0·51 (0·49;0·54) | <0·0001 | 0·49 (0·46;0·52) | <0·0001 | 0·51 (0·49;0·54) | <0·0001 | 0·52 (0·49;0·55) | <0·0001 | 0·52 (0·49;0·55) | <0·0001 | 0·62 (0·59;0·66) | <0·0001 |  |
| Reported barriers to vaccination |  |  |  |  |  |  |  |  |  |  |  |  |  |  |  |
| No |  |  | Ref. |  | Ref. |  | Ref. |  | Ref. |  | Ref. |  | Ref. |  |  |
| Yes |  |  | 1·81 (1·74;1·88) | <0·0001 | 1·51 (1·45;1·58) | <0·0001 | 1·43 (1·37;1·49) | <0·0001 | 1·33 (1·27;1·39) | <0·0001 | 1·33 (1·27;1·39) | <0·0001 | 1·16 (1·11;1·22) | <0·0001 |  |
| Information source cluster |  |  |  |  |  |  |  |  |  |  |  |  |  |  |  |
| Diversified sources |  |  |  |  | Ref. |  | Ref. |  | Ref. |  | Ref. |  | Ref. |  |  |
| Professional-only sources |  |  |  |  | 0·68 (0·65;0·71) | <0·0001 | 0·71 (0·68;0·75) | <0·0001 | 0·72 (0·69;0·76) | <0·0001 | 0·73 (0·69;0·76) | <0·0001 | 0·76 (0·72;0·8) | <0·0001 |  |
| Trust in sources |  |  |  |  | 0·35 (0·34;0·36) | <0·0001 | 0·38 (0·37;0·4) | <0·0001 | 0·44 (0·42;0·46) | <0·0001 | 0·44 (0·43;0·46) | <0·0001 | 0·53 (0·51;0·56) | <0·0001 |  |
| Perceived vaccination endorsement by religious leaders |  |  |  |  |  |  |  |  |  |  |  |  |  |  |  |
| Yes |  |  |  |  |  |  | Ref. |  | Ref. |  | Ref. |  | Ref. |  |  |
| No |  |  |  |  |  |  | 1·28 (1·19;1·38) | <0·0001 | 1·27 (1·18;1·37) | <0·0001 | 1·27 (1·18;1·37) | <0·0001 | 1·18 (1·08;1·28) | 0·00012 |  |
| Don’t know |  |  |  |  |  |  | 0·98 (0·93;1·04) | 0·58 | 0·99 (0·93;1·05) | 0·74 | 0·98 (0·92;1·04) | 0·57 | 1·01 (0·94;1·08) | 0·76 |  |
| Perceived vaccination endorsement by political leaders |  |  |  |  |  |  |  |  |  |  |  |  |  |  |  |
| Yes |  |  |  |  |  |  | Ref. |  | Ref. |  | Ref. |  | Ref. |  |  |
| No |  |  |  |  |  |  | 1·07 (0·99;1·15) | 0·0698 | 1·05 (0·97;1·13) | 0·198 | 1·05 (0·98;1·14) | 0·18 | 1·09 (1·01;1·19) | 0·033 |  |
| Don’t know |  |  |  |  |  |  | 0·68 (0·64;0·73) | <0·0001 | 0·68 (0·64;0·72) | <0·0001 | 0·69 (0·64;0·73) | <0·0001 | 0·84 (0·78;0·9) | <0·0001 |  |
| Perceived vaccination endorsement by teachers |  |  |  |  |  |  |  |  |  |  |  |  |  |  |  |
| Yes |  |  |  |  |  |  | Ref. |  | Ref. |  | Ref. |  | Ref. |  |  |
| No |  |  |  |  |  |  | 1·49 (1·38;1·61) | <0·0001 | 1·42 (1·31;1·54) | <0·0001 | 1·43 (1·32;1·55) | <0·0001 | 1·26 (1·15;1·37) | <0·0001 |  |
| Don’t know |  |  |  |  |  |  | 1·34 (1·26;1·42) | <0·0001 | 1·32 (1·24;1·4) | <0·0001 | 1·33 (1·25;1·42) | <0·0001 | 1·26 (1·17;1·35) | <0·0001 |  |
| Perceived vaccination endorsement by health professionals |  |  |  |  |  |  |  |  |  |  |  |  |  |  |  |
| Yes |  |  |  |  |  |  | Ref. |  | Ref. |  | Ref. |  | Ref. |  |  |
| No |  |  |  |  |  |  | 1·73 (1·59;1·87) | <0·0001 | 1·67 (1·53;1·81) | <0·0001 | 1·64 (1·51;1·78) | <0·0001 | 1·32 (1·21;1·45) | <0·0001 |  |
| Don’t know |  |  |  |  |  |  | 1·97 (1·86;2·1) | <0·0001 | 1·89 (1·77;2·01) | <0·0001 | 1·87 (1·75;1·99) | <0·0001 | 1·48 (1·38;1·58) | <0·0001 |  |
| Use of non-conventional medicine |  |  |  |  |  |  |  |  |  |  |  |  |  |  |  |
| No |  |  |  |  |  |  |  |  | Ref. |  | Ref. |  | Ref. |  |  |
| Yes, integrated with conventional medicine |  |  |  |  |  |  |  |  | 1·22 (1·16;1·29) | <0·0001 | 1·21 (1·15;1·28) | <0·0001 | 1·05 (0·99;1·11) | 0·11 |  |
| Yes, as alternative to conventional medicine |  |  |  |  |  |  |  |  | 2·28 (2·1;2·46) | <0·0001 | 2·27 (2·1;2·46) | <0·0001 | 1·57 (1·44;1·72) | <0·0001 |  |
| Political orientation |  |  |  |  |  |  |  |  |  |  |  |  |  |  |  |
| Right (7–9) |  |  |  |  |  |  |  |  | Ref. |  | Ref. |  | Ref. |  |  |
| Centre (4–6) |  |  |  |  |  |  |  |  | 0·97 (0·91;1·03) | 0·28 | 0·96 (0·91;1·02) | 0·19 | 1·11 (1·04;1·19) | 0·0013 |  |
| Extreme left (0) |  |  |  |  |  |  |  |  | 0·67 (0·6;0·76) | <0·0001 | 0·66 (0·59;0·74) | <0·0001 | 0·9 (0·78;1·02) | 0·109 |  |
| Left (1–3) |  |  |  |  |  |  |  |  | 0·56 (0·52;0·61) | <0·0001 | 0·56 (0·52;0·6) | <0·0001 | 0·79 (0·73;0·86) | <0·0001 |  |
| Extreme right (10) |  |  |  |  |  |  |  |  | 1·14 (1·02;1·28) | 0·024 | 1·11 (0·99;1·24) | 0·078 | 0·82 (0·72;0·93) | 0·0024 |  |
| Non-aligned with traditional parties |  |  |  |  |  |  |  |  | 1·03 (0·96;1·1) | 0·48 | 1·03 (0·96;1·11) | 0·37 | 1·18 (1·09;1·27) | <0·0001 |  |
| Prefer not to answer |  |  |  |  |  |  |  |  | 0·9 (0·82;0·99) | 0·027 | 0·95 (0·86;1·04) | 0·25 | 1·26 (1·13;1·4) | <0·0001 |  |
| Religion |  |  |  |  |  |  |  |  |  |  |  |  |  |  |  |
| Catholic |  |  |  |  |  |  |  |  | Ref. |  | Ref. |  | Ref. |  |  |
| Orthodox |  |  |  |  |  |  |  |  | 0·91 (0·8;1·05) | 0·19 | 0·93 (0·81;1·07) | 0·304 | 0·92 (0·79;1·06) | 0·25 |  |
| Protestant |  |  |  |  |  |  |  |  | 1·44 (1·14;1·82) | 0·0026 | 1·43 (1·13;1·81) | 0·00307 | 1·38 (1·07;1·79) | 0·014 |  |
| Jewish |  |  |  |  |  |  |  |  | 1·94 (1·28;2·97) | 0·00199 | 1·96 (1·28;2·98) | 0·0018 | 2·35 (1·48;3·72) | 0·00027 |  |
| Muslim |  |  |  |  |  |  |  |  | 1·19 (0·96;1·48) | 0·12 | 1·19 (0·95;1·48) | 0·12 | 1·04 (0·82;1·31) | 0·77 |  |
| Jehovah’s Witness |  |  |  |  |  |  |  |  | 1·41 (1·12;1·77) | 0·0032 | 1·4 (1·11;1·76) | 0·0038 | 1·57 (1·23;2) | 0·00031 |  |
| Atheist |  |  |  |  |  |  |  |  | 0·92 (0·84;1·01) | 0·075 | 0·93 (0·85;1·02) | 0·12 | 0·96 (0·87;1·06) | 0·45 |  |
| Agnostic |  |  |  |  |  |  |  |  | 0·93 (0·82;1·05) | 0·23 | 0·93 (0·82;1·05) | 0·24 | 1·05 (0·91;1·21) | 0·52 |  |
| Buddhist |  |  |  |  |  |  |  |  | 1·27 (0·95;1·71) | 0·102 | 1·28 (0·96;1·71) | 0·097 | 1·24 (0·9;1·71) | 0·19 |  |
| Hindu |  |  |  |  |  |  |  |  | 2·16 (1·12;4·15) | 0·022 | 2·18 (1·13;4·19) | 0·0197 | 1·44 (0·69;3) | 0·33 |  |
| Other |  |  |  |  |  |  |  |  | 1·45 (1·23;1·71) | <0·0001 | 1·42 (1·21;1·67) | <0·0001 | 1·32 (1·1;1·58) | 0·0035 |  |
| Prefer not to answer |  |  |  |  |  |  |  |  | 1·27 (1·15;1·41) | <0·0001 | 1·26 (1·14;1·4) | <0·0001 | 1·2 (1·07;1·34) | 0·00203 |  |
| Importance of religion |  |  |  |  |  |  |  |  |  |  |  |  |  |  |  |
| Not at all (0) |  |  |  |  |  |  |  |  | Ref. |  | Ref. |  | Ref. |  |  |
| Slightly (1–3) |  |  |  |  |  |  |  |  | 1·14 (1·04;1·24) | 0·00399 | 1·15 (1·05;1·26) | 0·0019 | 1·05 (0·95;1·16) | 0·31 |  |
| Somewhat important (4–6) |  |  |  |  |  |  |  |  | 1·23 (1·13;1·35) | <0·0001 | 1·26 (1·16;1·38) | <0·0001 | 1·1 (1;1·22) | 0·0503 |  |
| Very (7–9) |  |  |  |  |  |  |  |  | 1·13 (1·03;1·23) | 0·0095 | 1·18 (1·08;1·29) | 0·00038 | 0·96 (0·87;1·07) | 0·49 |  |
| Extremely (10) |  |  |  |  |  |  |  |  | 1·07 (0·96;1·2) | 0·21 | 1·08 (0·97;1·21) | 0·15 | 0·78 (0·69;0·88) | 0·00010 |  |
| Prefer not to answer |  |  |  |  |  |  |  |  | 1·46 (1·29;1·65) | <0·0001 | 1·58 (1·39;1·8) | <0·0001 | 1·08 (0·93;1·24) | 0·31 |  |
| Perceived quality of the National Health Service |  |  |  |  |  |  |  |  | 0·9 (0·89;0·91) | <0·0001 | 0·9 (0·89;0·92) | <0·0001 | 0·93 (0·91;0·94) | <0·0001 |  |
| Perceived access to the National Health Service |  |  |  |  |  |  |  |  | 0·91 (0·9;0·93) | <0·0001 | 0·91 (0·9;0·92) | <0·0001 | 0·89 (0·88;0·91) | <0·0001 |  |
| Survey mode |  |  |  |  |  |  |  |  |  |  |  |  |  |  |  |
| CAWI |  |  |  |  |  |  |  |  |  |  | Ref. |  | Ref. |  |  |
| CATI |  |  |  |  |  |  |  |  |  |  | 0·78 (0·73;0·83) | <0·0001 | 1·27 (1·18;1·36) | <0·0001 |  |
| Vaccine Conspiracy Belief Scale |  |  |  |  |  |  |  |  |  |  |  |  | 2·08 (2·05;2·12) | <0·0001 |  |

Abbreviations: adjOR adjusted Odds Ratio, CATI Computer Assisted Telephone Interviewing, CAWI Computer Assisted Web Interviewing, CI Confidence Interval, NHS National Health Service, OR Odds Ratio, VPD Vaccine Preventable Disease.

Note: Regression estimates should not be interpreted as causal effects.

*Results note on the sensitivity analysis adjusting for the VCBS (Block 7):* The VCBS had a mean of 3·15 (SD=1·79), with a higher mean among hesitant participants (4·29, SD=1·52, p<0·0001). VCBS was correlated with the aVHS score (r=0·693, p<0·0001). The regression models confirmed a relationship between VH and VCBS (adjOR=2·08, 95% CI=2·05 to 2·12, p<0·0001). Most variables retained similar directions and significance levels compared with the main model. However, for some groups, e.g., individuals who were separated/divorced, identified as bisexual, homemakers, retired, or of multi-ethnic, the associations observed in the main model were no longer significant, despite maintaining similar effect sizes. More substantial changes emerged. Living in belt or intermediate areas became significantly associated with higher hesitancy compared with residing in poles. Knowing someone who experienced AEFI remained significant but adjOR showed a 42% reduction. While being unaware of local pro-vaccine politicians remained protective, not knowing of any such figures became significantly associated with higher VH. Regarding political orientation, both centrists and non-aligned individuals became significantly more hesitant than those on the right, whereas the association with the extreme left lost significance. The extreme right gained significance, showing lower hesitancy. The association between religious importance and hesitancy lost significance overall, but being extremely religious (vs. not at all religious) became significantly protective. Participants who completed the survey via CATI were more likely to be hesitant.

## Table S5. Unweighted analysis: Hierarchical logistic regression model for vaccine hesitancy: model fit statistics by block

| **Block** | **Variables included** | **LL** | **LR χ²** | **df** | **Pseudo R²** | **LR Test vs previous block (χ², df)** | **p-value** |
| --- | --- | --- | --- | --- | --- | --- | --- |
| 1 | Sociodemographic and socioeconomic characteristics | –34,035·863 | 2,420·87 | 60 | 0·0343 | – | – |
| 2 | + Health-related characteristics and personal experience | –31,176·903 | 8,138·79 | 67 | 0·1155 | χ²(7) = 5,717·92 | <0·0001 |
| 3 | + Information sources and trust | –28,927·742 | 12,637·11 | 69 | 0·1793 | χ²(2) = 4,498·32 | <0·0001 |
| 4 | + External influences | –28,080·513 | 14,331·57 | 77 | 0·2033 | χ²(8) = 1,694·46 | <0·0001 |
| 5 | + Beliefs and attitudes | –27,035·921 | 16,420·75 | 103 | 0·2329 | χ²(26) = 2,089·18 | <0·0001 |
| 6 | + Survey mode (CAWI/CATI) | –27,005·842 | 16,480·91 | 104 | 0·2338 | χ²(1) = 60·16 | <0·0001 |
| 7 | + Conspiracy beliefs (VCBS – sensitivity analysis) | –22,541·437 | 25,409·72 | 105 | 0·3605 | χ²(1) = 8,928·81 | <0·0001 |

Abbreviations: chi-square statistic (χ²), degrees of freedom (df), likelihood ratio (LR), log-likelihood (LL).

## Table S6. Comparison of unweighted and post-stratification weighted estimates in the final multivariable model for vaccine hesitancy (Blocks 1–6).

|  | **Final model (Blocks 1-6)**  **unweighted** | | **Final model (Blocks 1-6) weighted** | | **Percentage difference adjOR** |
| --- | --- | --- | --- | --- | --- |
|  | **adjOR** | **SE** | **adjOR** | **SE** |  |
| **Block 1 - Sociodemographic and socioeconomic characteristics** |  |  |  |  |  |
| Age group |  |  |  |  |  |
| 18–29 | 1 |  | 1 |  |  |
| 30–44 | 1·468*** | -0·0613 | 1·456*** | -0·0625 | -0·82 |
| 45–59 | 1·581*** | -0·0697 | 1·571*** | -0·0711 | -0·63 |
| 60–74 | 1·790*** | -0·0985 | 1·751*** | -0·0992 | -2·18 |
| 75+ | 1·408*** | -0·0895 | 1·471*** | -0·101 | 4·47 |
| Gender |  |  |  |  |  |
| Male | 1 |  | 1 |  |  |
| Female | 1·013 | -0·0229 | 1·018 | -0·024 | 0·49 |
| Non-binary/Other | 2·120*** | -0·258 | 2·028*** | -0·248 | -4·34 |
| Prefer not to answer | 1·026 | -0·421 | 1·016 | -0·411 | -0·97 |
| Marital status |  |  |  |  |  |
| Single | 1 |  | 1 |  |  |
| Married | 0·899** | -0·0326 | 0·907* | -0·0344 | 0·89 |
| Separated/Divorced | 1·118* | -0·058 | 1·130* | -0·0613 | 1·07 |
| Cohabiting | 1·035 | -0·0416 | 1·046 | -0·0438 | 1·06 |
| Widowed | 0·763*** | -0·0483 | 0·790*** | -0·0544 | 3·54 |
| Children |  |  |  |  |  |
| No children | 1 |  | 1 |  |  |
| Only children ≤11 years | 0·934 | -0·0376 | 0·933 | -0·0384 | -0·11 |
| Only children 12-18 years | 0·889* | -0·0437 | 0·884* | -0·0445 | -0·56 |
| Only children >18 years | 0·779*** | -0·0295 | 0·788*** | -0·0312 | 1·16 |
| Children of various ages | 0·759*** | -0·0413 | 0·757*** | -0·0417 | -0·26 |
| Sexual orientation |  |  |  |  |  |
| Heterosexual | 1 |  | 1 |  |  |
| Homosexual | 0·796** | -0·0678 | 0·815* | -0·0774 | 2·39 |
| Bisexual | 0·768*** | -0·0596 | 0·790** | -0·0626 | 2·86 |
| Pansexual | 0·92 | -0·13 | 0·916 | -0·119 | -0·43 |
| Ace spectrum | 0·88 | -0·109 | 0·857 | -0·113 | -2·61 |
| Prefer not to answer | 0·649*** | -0·0333 | 0·657*** | -0·0363 | 1·23 |
| Municipality size (inhabitants) |  |  |  |  |  |
| ≤10,000 | 1 |  | 1 |  |  |
| 10,001–25,000 | 0·98 | -0·0302 | 0·971 | -0·0306 | -0·92 |
| 25,001–50,000 | 0·982 | -0·0372 | 0·977 | -0·0377 | -0·51 |
| 50,001–100,000 | 1·057 | -0·0546 | 1·067 | -0·0575 | 0·95 |
| 100,001–250,000 | 1·09 | -0·0686 | 1·111 | -0·0717 | 1·93 |
| >250,000 | 0·987 | -0·056 | 0·997 | -0·0589 | 1·01 |
| Geographic macro-area |  |  |  |  |  |
| North-West | 1 |  | 1 |  |  |
| North-East | 1·021 | -0·033 | 1·04 | -0·0343 | 1·86 |
| Centre | 1·002 | -0·032 | 1·02 | -0·034 | 1·80 |
| South | 0·920** | -0·0295 | 0·938 | -0·0309 | 1·96 |
| Islands | 0·858*** | -0·0345 | 0·876** | -0·0385 | 2·10 |
| Degree of urbanisation |  |  |  |  |  |
| Pole | 1 |  | 1 |  |  |
| Intermunicipal pole | 0·96 | -0·0704 | 0·993 | -0·0738 | 3·44 |
| Belt | 1·086 | -0·0494 | 1·08 | -0·0504 | -0·55 |
| Intermediate | 1·08 | -0·0559 | 1·091 | -0·0587 | 1·02 |
| Peripheral | 0·982 | -0·0585 | 0·997 | -0·0615 | 1·53 |
| Ultra-peripheral | 0·972 | -0·0987 | 0·963 | -0·1 | -0·93 |
| Education level |  |  |  |  |  |
| Upper secondary | 1 |  | 1 |  |  |
| Primary/None | 0·571*** | -0·0415 | 0·530*** | -0·0397 | -7·18 |
| Lower secondary | 1·051 | -0·034 | 1·066 | -0·0362 | 1·43 |
| University | 0·841*** | -0·0229 | 0·825*** | -0·0235 | -1·90 |
| Postgraduate | 0·703*** | -0·033 | 0·698*** | -0·0342 | -0·71 |
| Occupational status |  |  |  |  |  |
| Non-healthcare worker | 1 |  | 1 |  |  |
| Healthcare worker | 0·707*** | -0·0367 | 0·700*** | -0·0365 | -0·99 |
| Homemaker | 1·100* | -0·0481 | 1·087 | -0·0493 | -1·18 |
| Retired | 0·834*** | -0·0352 | 0·832*** | -0·038 | -0·24 |
| Student (non-health field) | 0·655*** | -0·0414 | 0·651*** | -0·0427 | -0·61 |
| Student (health field) | 0·539*** | -0·0505 | 0·537*** | -0·0534 | -0·37 |
| Job seeker | 1·019 | -0·0603 | 1·025 | -0·0632 | 0·59 |
| Unemployed | 1·023 | -0·0549 | 1·019 | -0·0578 | -0·39 |
| Other | 0·729 | -0·201 | 0·768 | -0·226 | 5·35 |
| Continent of citizenship |  |  |  |  |  |
| Italy | 1 |  | 1 |  |  |
| Europe (non-Italy) | 1·013 | -0·104 | 0·973 | -0·104 | -3·95 |
| Africa | 1·16 | -0·269 | 1·194 | -0·286 | 2·93 |
| America | 0·857 | -0·199 | 0·867 | -0·208 | 1·17 |
| Asia | 0·948 | -0·27 | 1·022 | -0·289 | 7·81 |
| Oceania | 1 |  |  |  |  |
| Self-identified ethnicity |  |  |  |  |  |
| European | 1 |  | 1 |  |  |
| Multi-ethnic | 1·352* | -0·183 | 1·238 | -0·164 | -8·43 |
| North American / Australian | 1·445 | -0·311 | 1·44 | -0·328 | -0·35 |
| Arab-Middle Eastern | 1·053 | -0·192 | 0·947 | -0·191 | -10·07 |
| North African | 1·312 | -0·239 | 1·254 | -0·239 | -4·42 |
| Latino-American | 0·887 | -0·139 | 0·885 | -0·146 | -0·23 |
| African American | 1·154 | -0·515 | 1·729 | -0·748 | 49·83 |
| Black African | 0·844 | -0·223 | 0·838 | -0·229 | -0·71 |
| Asian | 0·942 | -0·217 | 0·987 | -0·23 | 4·78 |
| Pacific Islands | 0·568 | -0·224 | 0·583 | -0·244 | 2·64 |
| Material deprivation |  |  |  |  |  |
| No deprivation | 1 |  | 1 |  |  |
| Severe deprivation | 1·014 | -0·0546 | 1·008 | -0·0568 | -0·59 |
| **Block 2 - Health-related characteristics and personal experience** |  |  |  |  |  |
| Chronic conditions |  |  |  |  |  |
| No chronic disease | 1 |  | 1 |  |  |
| One chronic disease | 0·954 | -0·0245 | 0·934* | -0·0249 | -2·10 |
| More than one chronic disease | 0·860*** | -0·0278 | 0·852*** | -0·0292 | -0·93 |
| Living with a person with disability |  |  |  |  |  |
| No |  |  |  |  |  |
| Yes | 1·003 | -0·029 | 1·011 | -0·031 | 0·80 |
| Inadequate health literacy |  |  |  |  |  |
| No |  |  |  |  |  |
| Yes | 1·134*** | -0·0257 | 1·142*** | -0·0271 | 0·71 |
| Knowing someone who had AEFI |  |  |  |  |  |
| No | 1 |  | 1 |  |  |
| Yes | 3·369*** | -0·084 | 3·439*** | -0·0897 | 2·08 |
| Knowing someone who had VPD |  |  |  |  |  |
| No | 1 |  | 1 |  |  |
| Yes | 0·523*** | -0·0148 | 0·522*** | -0·0155 | -0·19 |
| Reported barriers to vaccination |  |  |  |  |  |
| No | 1 |  | 1 |  |  |
| Yes | 1·330*** | -0·0307 | 1·350*** | -0·0322 | 1·50 |
| **Block 3 - Information sources and trust** |  |  |  |  |  |
| Information source cluster |  |  |  |  |  |
| Diversified sources | 1 |  | 1 |  |  |
| Professional-only sources | 0·726*** | -0·017 | 0·734*** | -0·0179 | 1·10 |
| Trust in sources | 0·444*** | -0·00886 | 0·449*** | -0·00941 | 1·13 |
| **Block 4 – External influences (perceived vaccination endorsement in the respondent’s community by:)** |  |  |  |  |  |
| By religious leaders |  |  |  |  |  |
| Yes | 1 |  | 1 |  |  |
| No | 1·273*** | -0·0491 | 1·313*** | -0·0531 | 3·14 |
| Don’t know | 0·982 | -0·0309 | 1·006 | -0·0324 | 2·44 |
| By political leaders |  |  |  |  |  |
| Yes | 1 |  | 1 |  |  |
| No | 1·053 | -0·0409 | 1·056 | -0·0429 | 0·28 |
| Don’t know | 0·686*** | -0·0223 | 0·691*** | -0·0235 | 0·73 |
| By teachers |  |  |  |  |  |
| Yes | 1 |  | 1 |  |  |
| No | 1·427*** | -0·059 | 1·398*** | -0·0611 | -2·03 |
| Don’t know | 1·331*** | -0·0425 | 1·328*** | -0·044 | -0·23 |
| By health professionals |  |  |  |  |  |
| Yes | 1 |  | 1 |  |  |
| No | 1·636*** | -0·0691 | 1·660*** | -0·0733 | 1·47 |
| Don’t know | 1·869*** | -0·0602 | 1·916*** | -0·0639 | 2·51 |
| Block 5 – Beliefs and attitudes |  |  |  |  |  |
| Use of non-conventional medicine |  |  |  |  |  |
| No | 1 |  | 1 |  |  |
| Yes, integrated with conventional medicine | 1·212*** | -0·0327 | 1·213*** | -0·0341 | 0·08 |
| Yes, as alternative to conventional medicine | 2·271*** | -0·0915 | 2·245*** | -0·0987 | -1·14 |
| Political orientation |  |  |  |  |  |
| Right (7–9) | 1 |  | 1 |  |  |
| Centre (4–6) | 0·961 | -0·0288 | 0·958 | -0·0303 | -0·31 |
| Extreme left (0) | 0·662*** | -0·0399 | 0·647*** | -0·0417 | -2·27 |
| Left (1–3) | 0·557*** | -0·0216 | 0·559*** | -0·023 | 0·36 |
| Extreme right (10) | 1·109 | -0·0653 | 1·11 | -0·0703 | 0·09 |
| Non-aligned with traditional parties | 1·032 | -0·0363 | 1·031 | -0·0379 | -0·10 |
| Prefer not to answer | 0·946 | -0·0455 | 0·896* | -0·0444 | -5·29 |
| Religion |  |  |  |  |  |
| Catholic | 1 |  | 1 |  |  |
| Orthodox | 0·931 | -0·0649 | 0·934 | -0·0675 | 0·32 |
| Protestant | 1·431** | -0·173 | 1·353* | -0·168 | -5·45 |
| Jewish | 1·956** | -0·421 | 1·873** | -0·423 | -4·24 |
| Muslim | 1·188 | -0·133 | 1·241 | -0·146 | 4·46 |
| Jehovah’s Witness | 1·400** | -0·163 | 1·506*** | -0·186 | 7·57 |
| Atheist | 0·931 | -0·0424 | 0·964 | -0·0457 | 3·54 |
| Agnostic | 0·927 | -0·0598 | 0·96 | -0·0644 | 3·56 |
| Buddhist | 1·279 | -0·19 | 1·267 | -0·197 | -0·94 |
| Hindu | 2·176* | -0·726 | 2·235* | -0·707 | 2·71 |
| Other | 1·420*** | -0·118 | 1·429*** | -0·119 | 0·63 |
| Prefer not to answer | 1·260*** | -0·0662 | 1·284*** | -0·0707 | 1·90 |
| Importance of religion |  |  |  |  |  |
| Not at all (0) | 1 |  | 1 |  |  |
| Slightly (1–3) | 1·151** | -0·0521 | 1·171*** | -0·0546 | 1·74 |
| Somewhat important (4–6) | 1·264*** | -0·0566 | 1·300*** | -0·0603 | 2·85 |
| Very (7–9) | 1·179*** | -0·0545 | 1·224*** | -0·059 | 3·82 |
| Extremely (10) | 1·084 | -0·0611 | 1·059 | -0·0627 | -2·31 |
| Prefer not to answer | 1·581*** | -0·103 | 1·601*** | -0·112 | 1·27 |
| Perceived NHS quality | 0·903*** | -0·00662 | 0·903*** | -0·00704 | 0·00 |
| Perceived NHS access | 0·912*** | -0·00645 | 0·911*** | -0·0069 | -0·11 |
| **Block 6 – Survey mode** |  |  |  |  |  |
| Survey mode |  |  |  |  |  |
| CAWI | 1 |  | 1 |  |  |
| CATI | 0·776*** | -0·0254 | 0·775*** | -0·0265 | -0·13 |

Abbreviations: adjOR adjusted Odds Ratio, AEFI Adverse Event Following Immunisation, CATI Computer-Assisted Telephone Interviewing, CAWI Computer-Assisted Web Interviewing, CI Confidence Interval, NHS National Health Service, VPD Vaccine Preventable Disease.

* p<0.05, ** p<0.01, *** p<0.001

## Table S7. Post-stratification weighted analysis: Predicted probabilities of vaccine hesitancy, calculated after the multiple logistic regression model with Blocks from 1 to 6

| **Variable** | **Predicted probability of Vaccine Hesitancy** |
| --- | --- |
|  | **% (95% CI)** |
| **Block 1 - Sociodemographic and socioeconomic characteristics** |  |
| **Age group** |  |
| 18–29 | 39·25% (37·98; 40·52) |
| 30–44 | 45·72% (44·7; 46·73) |
| 45–59 | 47·06% (46·24; 47·88) |
| 60–74 | 48·98% (47·94; 50·01) |
| 75+ | 45·9% (44·33; 47·48) |
| **Gender** |  |
| Male | 45·63% (45·06; 46·2) |
| Female | 45·95% (45·4; 46·5) |
| Non-binary/Other | 58·2% (54·02; 62·39) |
| Prefer not to answer | 45·9% (31·9; 59·89) |
| **Marital status** |  |
| Single | 46·76% (45·73; 47·8) |
| Married | 45·04% (44·46; 45·62) |
| Separated/Divorced | 48·93% (47·43; 50·43) |
| Cohabiting | 47·56% (46·41; 48·72) |
| Widowed | 42·62% (40·62; 44·62) |
| **Children** |  |
| No children | 48·17% (47·34; 49) |
| Only children <11 years | 46·94% (45·69; 48·18) |
| Only adolescents | 45·98% (44·45; 47·51) |
| Only adults | 43·94% (43·12; 44·77) |
| Children of various ages | 43·24% (41·57; 44·92) |
| **Sexual orientation** |  |
| Heterosexual | 46·5% (46·1; 46·91) |
| Homosexual | 42·93% (39·73; 46·13) |
| Bisexual | 42·39% (39·74; 45·03) |
| Pansexual | 44·96% (40·52; 49·4) |
| Ace spectrum | 43·79% (39·33; 48·25) |
| Prefer not to answer | 39·23% (37·49; 40·98) |
| **Municipality size (inhabitants)** |  |
| ≤10,000 | 45·8% (44·88; 46·72) |
| 10,001–25,000 | 45·28% (44·32; 46·25) |
| 25,001–50,000 | 45·39% (44·41; 46·38) |
| 50,001–100,000 | 46·95% (45·59; 48·3) |
| 100,001–250,000 | 47·66% (45·93; 49·38) |
| >250,000 | 45·75% (44·3; 47·21) |
| **Geographic macro-area** |  |
| North-West | 46·2% (45·46; 46·94) |
| North-East | 46·9% (46·03; 47·77) |
| Centre | 46·56% (45·68; 47·44) |
| South | 45·08% (44·25; 45·9) |
| Islands | 43·88% (42·61; 45·15) |
| **Degree of urbanization** |  |
| Pole | 45·18% (44·1; 46·27) |
| Intermunicipal pole | 45·06% (42·73; 47·38) |
| Belt | 46·54% (45·76; 47·32) |
| Intermediate | 46·72% (45·58; 47·85) |
| Peripheral | 45·12% (43·63; 46·62) |
| Ultra-peripheral | 44·52% (41·31; 47·73) |
| **Education level** |  |
| Upper secondary | 47·19% (46·65; 47·72) |
| Primary/None | 36·23% (33·88; 38·59) |
| Lower secondary | 48·32% (47·27; 49·38) |
| University | 43·78% (42·97; 44·6) |
| Postgraduate | 40·88% (39·33; 42·44) |
| **Occupational status** |  |
| Non-healthcare worker | 47·42% (46·72; 48·11) |
| Healthcare worker | 41·12% (39·4; 42·85) |
| Homemaker | 48·91% (47·46; 50·35) |
| Retired | 44·14% (42·98; 45·31) |
| Student (non-health field) | 39·87% (37·74; 42·01) |
| Student (health field) | 36·63% (33·44; 39·82) |
| Job seeker | 47·85% (45·75; 49·96) |
| Unemployed | 47·76% (45·84; 49·68) |
| Other | 42·74% (32·67; 52·82) |
| **Continent of citizenship** |  |
| Italy | 45·91% (45·52; 46·29) |
| Europe (non-Italy) | 45·43% (41·77; 49·09) |
| Africa | 49·05% (40·71; 57·39) |
| America | 43·4% (35·23; 51·56) |
| Asia | 46·28% (36·52; 56·05) |
| Oceania | - |
| **Self-identified ethnicity** |  |
| European | 45·87% (45·48; 46·26) |
| Multi-ethnic | 49·66% (45·06; 54·26) |
| North American / Australian | 52·35% (44·38; 60·32) |
| Arab-Middle Eastern | 44·9% (37·99; 51·81) |
| North African | 49·89% (43·25; 56·53) |
| Latino-American | 43·72% (38·1; 49·34) |
| African American | 55·61% (40·55; 70·68) |
| Black African | 42·77% (33·47; 52·06) |
| Asian | 45·64% (37·61; 53·67) |
| Pacific Islands | 36·63% (23·14; 50·12) |
| **Material deprivation** |  |
| No deprivation | 45·9% (45·51; 46·28) |
| Severe deprivation | 46·04% (44·14; 47·95) |
| **Block 2 - Health-related characteristics and personal experience** |  |
| **Chronic conditions** |  |
| No chronic disease | 46·75% (46·2; 47·3) |
| One chronic disease | 45·55% (44·83; 46·27) |
| More than one chronic disease | 43·93% (42·94; 44·91) |
| **Living with a person with disability** |  |
| No | 45·87% (45·45; 46·29) |
| Yes | 46·07% (45·11; 47·03) |
| **Inadequate health literacy** |  |
| No | 44·94% (44·43; 45·45) |
| Yes | 47·28% (46·67; 47·9) |
| **Knowing someone who had a vaccine adverse reaction** |  |
| No | 38·6% (38·11; 39·09) |
| Yes | 61·9% (61·14; 62·66) |
| **Knowing someone who had a VPD** |  |
| No | 48·37% (47·93; 48·8) |
| Yes | 37·19% (36·37; 38·01) |
| **Reported barriers to vaccination** |  |
| No | 48·77% (48·17; 49·36) |
| Yes | 43·4% (42·86; 43·95) |
| **Block 3 - Information sources and trust** |  |
| **Information source cluster** |  |
| Diversified sources | 47·89% (47·4; 48·39) |
| Professional-only sources | 42·37% (41·7; 43·04) |
| **Trust in sources** | - |
| **Block 4 – External influences (perceived vaccination endorsement in the respondent’s community by:)** |  |
| **By religious leaders** |  |
| Yes | 44·94% (44·1; 45·79) |
| No | 49·81% (48·66; 50·96) |
| Don’t know | 45·05% (44·44; 45·65) |
| **By political leaders** |  |
| Yes | 48·6% (47·87; 49·33) |
| No | 49·57% (48·35; 50·8) |
| Don’t know | 42·07% (41·34; 42·81) |
| **By teachers** |  |
| Yes | 42·97% (42·28; 43·65) |
| No | 48·95% (47·59; 50·31) |
| Don’t know | 48·02% (47·26; 48·78) |
| **By health professionals** |  |
| Yes | 41·64% (41·1; 42·19) |
| No | 50·82% (49·37; 52·27) |
| Don’t know | 53·45% (52·51; 54·39) |
| **Block 5 – Beliefs and attitudes** |  |
| **Use of non-conventional medicine** |  |
| No | 43·84% (43·36; 44·31) |
| Yes, integrated with conventional medicine | 47·32% (46·47; 48·16) |
| Yes, as alternative to conventional medicine | 58·47% (57·01; 59·93) |
| **Political orientation** |  |
| Right (7–9) | 47·04% (46·34; 47·73) |
| Centre (4–6) | 40·15% (38·19; 42·12) |
| Extreme left (0) | 37·67% (36·62; 38·72) |
| Left (1–3) | 47·82% (46·94; 48·69) |
| Extreme right (10) | 49·7% (47·62; 51·77) |
| Non-aligned with traditional parties | 48·36% (47·44; 49·28) |
| Prefer not to answer | 45·85% (44·37; 47·33) |
| **Religion** |  |
| Catholic | 45·45% (44·97; 45·94) |
| Orthodox | 44·24% (41·8; 46·69) |
| Protestant | 50·85% (46·5; 55·2) |
| Jewish | 56·66% (48·8; 64·53) |
| Muslim | 49·3% (45·22; 53·38) |
| Jehovah’s Witness | 52·77% (48·47; 57·08) |
| Atheist | 44·81% (43·33; 46·28) |
| Agnostic | 44·73% (42·52; 46·95) |
| Buddhist | 49·68% (44·24; 55·11) |
| Hindu | 59·79% (48·92; 70·66) |
| Other | 51·83% (48·96; 54·7) |
| Prefer not to answer | 49·9% (48·07; 51·74) |
| **Importance of religion** |  |
| Not at all (0) | 47·24% (46·49; 48) |
| Slightly (1–3) | 42·62% (41·32; 43·93) |
| Somewhat important (4–6) | 45·39% (44·34; 46·43) |
| Very (7–9) | 46·18% (45·43; 46·93) |
| Extremely (10) | 43·63% (42·22; 45·04) |
| Prefer not to answer | 50·95% (48·91; 52·99) |
| **Perceived quality of the NHS** | - |
| **Perceived access to the NHS** | - |
| **Block 6 – Survey mode** |  |
| **Survey mode** |  |
| CAWI | 42·67% (41·76; 43·58) |
| CATI | 47·19% (46·67; 47·72) |

Abbreviations: CATI Computer Assisted Telephone Interviewing, CAWI Computer Assisted Web Interviewing, CI Confidence Interval, NHS National Health Service, VPD Vaccine Preventable Disease.

## Table S8. Unweighted analysis: Predicted probabilities of vaccine hesitancy, calculated after the multiple logistic regression model with Blocks from 1 to 6

| **Variable** | **Cases in the final model**  **n=51145**  **N (%)** | **Predicted probability of Vaccine Hesitancy** |
| --- | --- | --- |
|  |  | **% (95% CI)** |
| **Block 1 - Sociodemographic and socioeconomic characteristics** |  |  |
| **Age group** |  |  |
| 18–29 | 7343 (14·36%) | 38·85% (37·62; 40·08) |
| 30–44 | 10463 (20·46%) | 45·48% (44·48; 46·48) |
| 45–59 | 14464 (28·28%) | 46·78% (45·98; 47·59) |
| 60–74 | 11155 (21·81%) | 48·99% (47·97; 50·01) |
| 75+ | 7720 (15·09%) | 44·75% (43·36; 46·14) |
| **Gender** |  |  |
| Male | 24604 (48·11%) | 45·29% (44·75; 45·84) |
| Female | 26037 (50·91%) | 45·52% (45; 46·05) |
| Non-binary/Other | 469 (0·92%) | 58·7% (54·53; 62·87) |
| Prefer not to answer | 35 (0·07%) | 45·75% (31·53; 59·97) |
| **Marital status** |  |  |
| Single | 11664 (22·81%) | 46·56% (45·56; 47·55) |
| Married | 27312 (53·4%) | 44·67% (44·11; 45·22) |
| Separated/Divorced | 3532 (6·91%) | 48·54% (47·1; 49·98) |
| Cohabiting | 6148 (12·02%) | 47·17% (46·07; 48·27) |
| Widowed | 2489 (4·87%) | 41·78% (39·97; 43·6) |
| **Children** |  |  |
| No children | 18349 (35·88%) | 47·86% (47·06; 48·66) |
| Only children <11 years | 6131 (11·99%) | 46·64% (45·42; 47·87) |
| Only adolescents | 3404 (6·66%) | 45·77% (44·27; 47·26) |
| Only adults | 20680 (40·43%) | 43·43% (42·63; 44·22) |
| Children of various ages | 2581 (5·05%) | 42·96% (41·3; 44·63) |
| **Sexual orientation** |  |  |
| Heterosexual | 45335 (88·64%) | 46·18% (45·79; 46·58) |
| Homosexual | 841 (1·64%) | 42·18% (39·32; 45·05) |
| Bisexual | 1083 (2·12%) | 41·57% (38·99; 44·16) |
| Pansexual | 364 (0·71%) | 44·72% (39·89; 49·54) |
| Ace spectrum | 488 (0·95%) | 43·94% (39·73; 48·15) |
| Prefer not to answer | 3034 (5·93%) | 38·7% (37·08; 40·32) |
| **Municipality size (inhabitants)** |  |  |
| ≤10,000 | 15595 (30·49%) | 45·47% (44·57; 46·36) |
| 10,001–25,000 | 10707 (20·93%) | 45·11% (44·16; 46·06) |
| 25,001–50,000 | 7456 (14·58%) | 45·14% (44·16; 46·12) |
| 50,001–100,000 | 5572 (10·89%) | 46·44% (45·16; 47·73) |
| 100,001–250,000 | 3904 (7·63%) | 46·99% (45·31; 48·67) |
| >250,000 | 7911 (15·47%) | 45·23% (43·87; 46·6) |
| **Geographic macro-area** |  |  |
| North-West | 13795 (26·97%) | 46·07% (45·35; 46·8) |
| North-East | 10096 (19·74%) | 46·44% (45·58; 47·31) |
| Centre | 10232 (20·01%) | 46·1% (45·27; 46·93) |
| South | 11536 (22·56%) | 44·61% (43·81; 45·4) |
| Islands | 5486 (10·73%) | 43·37% (42·24; 44·51) |
| **Degree of urbanization** |  |  |
| Pole | 18369 (35·92%) | 44·84% (43·79; 45·88) |
| Intermunicipal pole | 1291 (2·52%) | 44·12% (41·81; 46·43) |
| Belt | 19086 (37·32%) | 46·3% (45·54; 47·06) |
| Intermediate | 7359 (14·39%) | 46·19% (45·09; 47·29) |
| Peripheral | 4290 (8·39%) | 44·52% (43·08; 45·96) |
| Ultra-peripheral | 750 (1·47%) | 44·34% (41·2; 47·49) |
| **Education level** |  |  |
| Upper secondary | 26503 (51·82%) | 46·75% (46·24; 47·26) |
| Primary/None | 1437 (2·81%) | 37·05% (34·73; 39·37) |
| Lower secondary | 7712 (15·08%) | 47·64% (46·64; 48·65) |
| University | 12309 (24·07%) | 43·68% (42·9; 44·46) |
| Postgraduate | 3184 (6·23%) | 40·57% (39·08; 42·06) |
| **Occupational status** |  |  |
| Non-healthcare worker | 23459 (45·87%) | 47·01% (46·36; 47·67) |
| Healthcare worker | 2512 (4·91%) | 40·9% (39·19; 42·62) |
| Homemaker | 4033 (7·89%) | 48·72% (47·32; 50·12) |
| Retired | 13977 (27·33%) | 43·79% (42·7; 44·89) |
| Student (non-health field) | 2079 (4·06%) | 39·57% (37·51; 41·62) |
| Student (health field) | 809 (1·58%) | 36·28% (33·28; 39·29) |
| Job seeker | 1863 (3·64%) | 47·35% (45·33; 49·38) |
| Unemployed | 2339 (4·57%) | 47·43% (45·62; 49·24) |
| Other | 74 (0·14%) | 41·42% (32·04; 50·8) |
| **Continent of citizenship** |  |  |
| Italy | 50178 (98·11%) | 45·53% (45·16; 45·9) |
| Europe (non-Italy) | 582 (1·14%) | 45·76% (42·24; 49·28) |
| Africa | 137 (0·27%) | 48·17% (40·09; 56·25) |
| America | 142 (0·28%) | 42·82% (34·91; 50·72) |
| Asia | 106 (0·21%) | 44·59% (34·79; 54·39) |
| Oceania |  |  |
| **Self-identified ethnicity** |  |  |
| European | 49482 (96·75%) | 45·48% (45·11; 45·85) |
| Multi-ethnic | 381 (0·74%) | 50·86% (46·13; 55·59) |
| North American / Australian | 157 (0·31%) | 52·05% (44·5; 59·6) |
| Arab-Middle Eastern | 218 (0·43%) | 46·39% (40·07; 52·71) |
| North African | 233 (0·46%) | 50·31% (43·95; 56·68) |
| Latino-American | 336 (0·66%) | 43·38% (38·04; 48·72) |
| African American | 37 (0·07%) | 48·03% (32·46; 63·6) |
| Black African | 93 (0·18%) | 42·51% (33·53; 51·5) |
| Asian | 163 (0·32%) | 44·42% (36·49; 52·35) |
| Pacific Islands | 45 (0·09%) | 35·79% (23·13; 48·46) |
| **Material deprivation** |  |  |
| No deprivation | 48976 (95·76%) | 45·52% (45·15; 45·89) |
| Severe deprivation | 2169 (4·24%) | 45·77% (43·95; 47·6) |
| **Block 2 - Health-related characteristics and personal experience** |  |  |
| **Chronic conditions** |  |  |
| No chronic disease | 27490 (53·75%) | 46·25% (45·73; 46·78) |
| One chronic disease | 14454 (28·26%) | 45·42% (44·72; 46·12) |
| More than one chronic disease | 9201 (17·99%) | 43·6% (42·68; 44·52) |
| **Living with a person with disability** |  |  |
| No | 42195 (82·5%) | 45·52% (45·11; 45·92) |
| Yes | 8950 (17·5%) | 45·57% (44·57; 46·48) |
| **Inadequate health literacy** |  |  |
| No | 30289 (59·22%) | 44·60% (44·11; 45·09) |
| Yes | 20856 (40·78%) | 46·83% (46·24; 47·41) |
| **Knowing someone who had a vaccine adverse reaction** |  |  |
| No | 34935 (68·31%) | 38·35% (37·88; 38·82) |
| Yes | 16210 (31·69%) | 61·28% (60·55; 62·02) |
| **Knowing someone who had a VPD** |  |  |
| No | 39481 (77·19%) | 48·03% (47·61; 48·45) |
| Yes | 11664 (22·81%) | 36·88% (36·1; 37·66) |
| **Reported barriers to vaccination** |  |  |
| No | 27118 (53·02%) | 43·12% (42·59; 43·65) |
| Yes | 24027 (46·98%) | 48·22% (47·65; 48·79) |
| **Block 3 - Information sources and trust** |  |  |
| **Information source cluster** |  |  |
| Diversified sources | 32027 (62·62%) | 47·59% (47·11; 48·06) |
| Professional-only sources | 19118 (37·38%) | 41·86% (41·22; 42·5) |
| **Trust in sources** | - | - |
| **Block 4 – External influences (perceived vaccination endorsement in the respondent’s community by:)** |  |  |
| **By religious leaders** |  |  |
| Yes | 13947 (27·27%) | 44·9% (44·07; 45·73) |
| No | 9828 (19·22%) | 49·22% (48·13; 50·31) |
| Don’t know | 27370 (53·51%) | 44·59% (44; 45·17) |
| **By political leaders** |  |  |
| Yes | 20329 (39·75%) | 48·32% (47·62; 49·03) |
| No | 8926 (17·45%) | 49·26% (48·08; 50·43) |
| Don’t know | 21890 (42·8%) | 41·65% (40·95; 42·36) |
| **By teachers** |  |  |
| Yes | 22985 (44·94%) | 42·53% (41·87; 43·18) |
| No | 7544 (14·75%) | 48·89% (47·6; 50·18) |
| Don’t know | 20616 (40·31%) | 47·63% (46·89; 48·36) |
| **By health professionals** |  |  |
| Yes | 31651 (61·88%) | 41·43% (40·9; 41·96) |
| No | 6165 (12·05%) | 50·34% (48·95; 51·73) |
| Don’t know | 13329 (26·06%) | 52·78% (51·87; 53·69) |
| **Block 5 – Beliefs and attitudes** |  |  |
| **Use of non-conventional medicine** | 34817 (68·08%) |  |
| No | 11572 (22·63%) | 43·46% (43; 43·92) |
| Yes, integrated with conventional medicine | 4756 (9·3%) | 46·93% (46·11; 47·74) |
| Yes, as alternative to conventional medicine |  | 58·35% (57·01; 59·68) |
| **Political orientation** |  |  |
| Right (7–9) | 10808 (21·13%) | 47·31% (46·49; 48·12) |
| Centre (4–6) | 15733 (30·76%) | 46·61% (45·93; 47·28) |
| Extreme left (0) | 2095 (4·1%) | 40·03% (38·18; 41·88) |
| Left (1–3) | 7056 (13·8%) | 37·12% (36·12; 38·12) |
| Extreme right (10) | 1958 (3·83%) | 49·18% (47·25; 51·1) |
| Non-aligned with traditional parties | 9191 (17·97%) | 47·88% (46·98; 48·78) |
| Prefer not to answer | 4304 (8·42%) | 46·32% (44·87; 47·77) |
| **Religion** |  |  |
| Catholic | 36755 (71·86%) | 45·2% (44·74; 45·67) |
| Orthodox | 1542 (3·01%) | 43·94% (41·58; 46·29) |
| Protestant | 443 (0·87%) | 51·62% (47·38; 55·86) |
| Jewish | 164 (0·32%) | 57·23% (49·73; 64·72) |
| Muslim | 652 (1·27%) | 48·27% (44·4; 52·14) |
| Jehovah’s Witness | 479 (0·94%) | 51·23% (47·16; 55·31) |
| Atheist | 5428 (10·61%) | 43·94% (42·53; 45·36) |
| Agnostic | 1733 (3·39%) | 43·86% (41·73; 45·99) |
| Buddhist | 280 (0·55%) | 49·61% (44·4; 54·81) |
| Hindu | 67 (0·13%) | 59·12% (47·58; 70·67) |
| Other | 918 (1·79%) | 51·48% (48·6; 54·37) |
| Prefer not to answer | 2684 (5·25%) | 49·34% (47·58; 51·09) |
| **Importance of religion** |  |  |
| Not at all (0) | 7286 (14·25%) | 42·61% (41·34; 43·87) |
| Slightly (1–3) | 7045 (13·77%) | 45·08% (44·07; 46·08) |
| Somewhat important (4–6) | 14027 (27·43%) | 46·74% (46·02; 47·46) |
| Very (7–9) | 16003 (31·29%) | 45·5% (44·78; 46·21) |
| Extremely (10) | 4233 (8·28%) | 44·02% (42·68; 45·36) |
| Prefer not to answer | 2551 (4·99%) | 50·74% (48·88; 52·59) |
| **Perceived quality of the NHS** | - | - |
| **Perceived access to the NHS** | - | - |
| **Block 6 – Survey mode** |  |  |
| **Survey mode** |  |  |
| CAWI | 35727 (69·85%) | 46·87% (46·37; 47·38) |
| CATI | 15418 (30·15%) | 42·37% (41·5; 43·25) |

Abbreviations: CATI Computer Assisted Telephone Interviewing, CAWI Computer Assisted Web Interviewing, CI Confidence Interval, NHS National Health Service, VPD Vaccine Preventable Disease.

Cell counts reported next to the predicted probabilities refer to the analytic sample for the final multivariable model (complete-case analysis), and may therefore slightly differ from the descriptive counts reported in Tables S1 and S2.

## Table S9. Post-stratification weighted analysis: Pairwise comparisons of predicted probabilities of vaccine hesitancy, calculated after the multiple logistic regression model with Blocks from 1 to 6 (Bonferroni correction)

| **Comparison** | **Δ Pr(hesitancy)** | **95% CI (Bonferroni)** | **p (Bonferroni)*** |
| --- | --- | --- | --- |
| **Block 1 - Sociodemographic and socioeconomic characteristics** | | | |
| **Age group** |  |  |  |
| 30–44 vs 18–29 | 0·0647 | 0·0441, 0·0852 | <0·001 |
| 45–59 vs 18–29 | 0·0781 | 0·0566, 0·0997 | <0·001 |
| 60–74 vs 18–29 | 0·0973 | 0·0704, 0·1242 | <0·001 |
| 75+ vs 18–29 | 0·0665 | 0·0337, 0·0993 | <0·001 |
| 45–59 vs 30–44 | 0·0135 | -0·0036, 0·0306 | 0·272 |
| 60–74 vs 30–44 | 0·0326 | 0·0083, 0·0568 | 0·002 |
| 75+ vs 30–44 | 0·0018 | -0·0291, 0·0328 | 1 |
| 60–74 vs 45–59 | 0·0191 | -0·0016, 0·0399 | 0·095 |
| 75+ vs 45–59 | -0·0116 | -0·0398, 0·0166 | 1 |
| 75+ vs 60–74 | -0·0307 | -0·0524, -0·0091 | 0·001 |
| **Gender** |  |  |  |
| Female vs Male | 0·0032 | -0·0078, 0·0142 | 1 |
| Non-binary/Other vs Male | 0·1258 | 0·0688, 0·1827 | <0·001 |
| Prefer not to answer vs Male | 0·0027 | -0·1859, 0·1913 | 1 |
| Non-binary/Other vs Female | 0·1226 | 0·0657, 0·1795 | <0·001 |
| Prefer not to answer vs Female | -0·0005 | -0·189, 0·188 | 1 |
| Prefer not to answer vs Non-binary/Other | -0·1231 | -0·3191, 0·073 | 0·586 |
| **Marital status** |  |  |  |
| Married vs Single | -0·0172 | -0·0361, 0·0017 | 0·105 |
| Separated/Divorced vs Single | 0·0217 | -0·0055, 0·0489 | 0·25 |
| Cohabiting vs Single | 0·0080 | -0·0129, 0·0289 | 1 |
| Widowed vs Single | -0·0414 | -0·0753, -0·0075 | 0·006 |
| Separated/Divorced vs Married | 0·0389 | 0·0162, 0·0616 | <0·001 |
| Cohabiting vs Married | 0·0252 | 0·0061, 0·0444 | 0·002 |
| Widowed vs Married | -0·0242 | -0·0536, 0·0051 | 0·206 |
| Cohabiting vs Separated/Divorced | -0·0137 | -0·041, 0·0137 | 1 |
| Widowed vs Separated/Divorced | -0·0631 | -0·099, -0·0272 | <0·001 |
| Widowed vs Cohabiting | -0·0494 | -0·0836, -0·0153 | <0·001 |
| **Children** |  |  |  |
| Only children ≤11 years vs No children | -0·0123 | -0·0329, 0·0082 | 0·923 |
| Only children 12-18 years vs No children | -0·0219 | -0·047, 0·0032 | 0·143 |
| Only children >18 years vs No children | -0·0423 | -0·062, -0·0225 | <0·001 |
| Children of various ages vs No children | -0·0493 | -0·0764, -0·0221 | <0·001 |
| Only children 12-18 years vs Only children ≤11 years | -0·0096 | -0·0365, 0·0174 | 1 |
| Only children >18 years vs Only children ≤11 years | -0·0299 | -0·0536, -0·0063 | 0·004 |
| Children of various ages vs Only children ≤11 years | -0·0369 | -0·0654, -0·0084 | 0·003 |
| Only children >18 years vs Only children 12-18 years | -0·0204 | -0·0459, 0·0052 | 0·252 |
| Children of various ages vs Only children 12-18 years | -0·0274 | -0·0583, 0·0035 | 0·129 |
| Children of various ages vs Only children >18 years | -0·0070 | -0·0342, 0·0202 | 1 |
| **Sexual orientation** |  |  |  |
| Homosexual vs Heterosexual | -0·0358 | -0·0841, 0·0126 | 0·449 |
| Bisexual vs Heterosexual | -0·0412 | -0·0814, -0·001 | 0·04 |
| Pansexual vs Heterosexual | -0·0154 | -0·0824, 0·0515 | 1 |
| Ace spectrum vs Heterosexual | -0·0271 | -0·0945, 0·0402 | 1 |
| Prefer not to answer vs Heterosexual | -0·0727 | -0·1, -0·0454 | <0·001 |
| Bisexual vs Homosexual | -0·0054 | -0·0667, 0·0559 | 1 |
| Pansexual vs Homosexual | 0·0203 | -0·0607, 0·1013 | 1 |
| Ace spectrum vs Homosexual | 0·0086 | -0·0728, 0·0901 | 1 |
| Prefer not to answer vs Homosexual | -0·0369 | -0·0914, 0·0175 | 0·698 |
| Pansexual vs Bisexual | 0·0257 | -0·0503, 0·1018 | 1 |
| Ace spectrum vs Bisexual | 0·0140 | -0·0624, 0·0905 | 1 |
| Prefer not to answer vs Bisexual | -0·0315 | -0·0786, 0·0156 | 0·742 |
| Ace spectrum vs Pansexual | -0·0117 | -0·1037, 0·0803 | 1 |
| Prefer not to answer vs Pansexual | -0·0573 | -0·1281, 0·0136 | 0·264 |
| Prefer not to answer vs Ace spectrum | -0·0456 | -0·1167, 0·0256 | 0·903 |
| **Municipality size (inhabitants)** |  |  |  |
| 10,001–25,000 vs ≤10,000 | -0·0052 | -0·0215, 0·0112 | 1 |
| 25,001–50,000 vs ≤10,000 | -0·0040 | -0·024, 0·0159 | 1 |
| 50,001–100,000 vs ≤10,000 | 0·0115 | -0·0165, 0·0395 | 1 |
| 100,001–250,000 vs ≤10,000 | 0·0186 | -0·015, 0·0522 | 1 |
| >250,000 vs ≤10,000 | -0·0005 | -0·0311, 0·0301 | 1 |
| 25,001–50,000 vs 10,001–25,000 | 0·0011 | -0·0192, 0·0214 | 1 |
| 50,001–100,000 vs 10,001–25,000 | 0·0167 | -0·011, 0·0443 | 1 |
| 100,001–250,000 vs 10,001–25,000 | 0·0238 | -0·0094, 0·0569 | 0·531 |
| >250,000 vs 10,001–25,000 | 0·0047 | -0·0256, 0·035 | 1 |
| 50,001–100,000 vs 25,001–50,000 | 0·0155 | -0·0097, 0·0408 | 1 |
| 100,001–250,000 vs 25,001–50,000 | 0·0226 | -0·0076, 0·0529 | 0·418 |
| >250,000 vs 25,001–50,000 | 0·0036 | -0·0233, 0·0304 | 1 |
| 100,001–250,000 vs 50,001–100,000 | 0·0071 | -0·0212, 0·0354 | 1 |
| >250,000 vs 50,001–100,000 | -0·0120 | -0·0366, 0·0127 | 1 |
| >250,000 vs 100,001–250,000 | -0·0191 | -0·0457, 0·0075 | 0·533 |
| **Geographic macro-area** |  |  |  |
| North-East vs North-West | 0·0070 | -0·0094, 0·0234 | 1 |
| Centre vs North-West | 0·0035 | -0·013, 0·0201 | 1 |
| South vs North-West | -0·0113 | -0·0275, 0·005 | 0·521 |
| Islands vs North-West | -0·0232 | -0·0448, -0·0017 | 0·025 |
| Centre vs North-East | -0·0034 | -0·0213, 0·0144 | 1 |
| South vs North-East | -0·0183 | -0·0358, -0·0007 | 0·035 |
| Islands vs North-East | -0·0302 | -0·0525, -0·008 | 0·001 |
| South vs Centre | -0·0148 | -0·0321, 0·0025 | 0·162 |
| Islands vs Centre | -0·0268 | -0·049, -0·0045 | 0·007 |
| Islands vs South | -0·0120 | -0·0331, 0·0091 | 1 |
| **Degree of urbanisation** |  |  |  |
| Intermunicipal pole vs Pole | -0·0013 | -0·0396, 0·0371 | 1 |
| Belt vs Pole | 0·0136 | -0·0105, 0·0377 | 1 |
| Intermediate vs Pole | 0·0153 | -0·0125, 0·0432 | 1 |
| Peripheral vs Pole | -0·0006 | -0·0325, 0·0312 | 1 |
| Ultra-peripheral vs Pole | -0·0066 | -0·0601, 0·0469 | 1 |
| Belt vs Intermunicipal pole | 0·0149 | -0·0219, 0·0516 | 1 |
| Intermediate vs Intermunicipal pole | 0·0166 | -0·0225, 0·0557 | 1 |
| Peripheral vs Intermunicipal pole | 0·0006 | -0·0412, 0·0424 | 1 |
| Ultra-peripheral vs Intermunicipal pole | -0·0054 | -0·0652, 0·0544 | 1 |
| Intermediate vs Belt | 0·0017 | -0·016, 0·0194 | 1 |
| Peripheral vs Belt | -0·0142 | -0·037, 0·0086 | 1 |
| Ultra-peripheral vs Belt | -0·0202 | -0·0684, 0·0279 | 1 |
| Peripheral vs Intermediate | -0·0160 | -0·0409, 0·009 | 0·905 |
| Ultra-peripheral vs Intermediate | -0·0220 | -0·071, 0·027 | 1 |
| Ultra-peripheral vs Peripheral | -0·0060 | -0·0564, 0·0443 | 1 |
| **Education level** |  |  |  |
| Primary/None vs Upper secondary | -0·1095 | -0·1443, -0·0747 | <0·001 |
| Lower secondary vs Upper secondary | 0·0114 | -0·0056, 0·0284 | 0·599 |
| University vs Upper secondary | -0·0340 | -0·0481, -0·0199 | <0·001 |
| Postgraduate vs Upper secondary | -0·0630 | -0·0868, -0·0393 | <0·001 |
| Lower secondary vs Primary/None | 0·1209 | 0·085, 0·1568 | <0·001 |
| University vs Primary/None | 0·0755 | 0·0391, 0·1119 | <0·001 |
| Postgraduate vs Primary/None | 0·0465 | 0·0054, 0·0876 | 0·015 |
| University vs Lower secondary | -0·0454 | -0·0654, -0·0254 | <0·001 |
| Postgraduate vs Lower secondary | -0·0744 | -0·1022, -0·0467 | <0·001 |
| Postgraduate vs University | -0·0290 | -0·0535, -0·0046 | 0·009 |
| **Occupational status** |  |  |  |
| Healthcare worker vs Non-healthcare worker | -0·0629 | -0·0919, -0·034 | <0·001 |
| Homemaker vs Non-healthcare worker | 0·0149 | -0·0111, 0·0409 | 1 |
| Retired vs Non-healthcare worker | -0·0327 | -0·0587, -0·0067 | 0·002 |
| Student (non-health field) vs Non-healthcare worker | -0·0754 | -0·1115, -0·0393 | <0·001 |
| Student (health field) vs Non-healthcare worker | -0·1078 | -0·1608, -0·0549 | <0·001 |
| Job seeker vs Non-healthcare worker | 0·0044 | -0·0309, 0·0397 | 1 |
| Unemployed vs Non-healthcare worker | 0·0034 | -0·029, 0·0358 | 1 |
| Other vs Non-healthcare worker | -0·0467 | -0·2114, 0·1179 | 1 |
| Homemaker vs Healthcare worker | 0·0778 | 0·0411, 0·1145 | <0·001 |
| Retired vs Healthcare worker | 0·0302 | -0·0063, 0·0667 | 0·293 |
| Student (non-health field) vs Healthcare worker | -0·0125 | -0·0565, 0·0315 | 1 |
| Student (health field) vs Healthcare worker | -0·0449 | -0·1033, 0·0135 | 0·503 |
| Job seeker vs Healthcare worker | 0·0673 | 0·0236, 0·111 | <0·001 |
| Unemployed vs Healthcare worker | 0·0664 | 0·0248, 0·108 | <0·001 |
| Other vs Healthcare worker | 0·0162 | -0·1504, 0·1828 | 1 |
| Retired vs Homemaker | -0·0476 | -0·0788, -0·0164 | <0·001 |
| Student (non-health field) vs Homemaker | -0·0903 | -0·1328, -0·0478 | <0·001 |
| Student (health field) vs Homemaker | -0·1227 | -0·18, -0·0654 | <0·001 |
| Job seeker vs Homemaker | -0·0105 | -0·052, 0·0309 | 1 |
| Unemployed vs Homemaker | -0·0114 | -0·05, 0·0271 | 1 |
| Other vs Homemaker | -0·0616 | -0·2276, 0·1043 | 1 |
| Student (non-health field) vs Retired | -0·0427 | -0·0855, 0·0001 | 0·052 |
| Student (health field) vs Retired | -0·0751 | -0·1327, -0·0175 | 0·001 |
| Job seeker vs Retired | 0·0371 | -0·005, 0·0792 | 0·175 |
| Unemployed vs Retired | 0·0362 | -0·0029, 0·0752 | 0·111 |
| Other vs Retired | -0·0140 | -0·1798, 0·1518 | 1 |
| Student (health field) vs Student (non-health field) | -0·0324 | -0·0908, 0·026 | 1 |
| Job seeker vs Student (non-health field) | 0·0798 | 0·0335, 0·126 | <0·001 |
| Unemployed vs Student (non-health field) | 0·0789 | 0·0337, 0·124 | <0·001 |
| Other vs Student (non-health field) | 0·0287 | -0·139, 0·1964 | 1 |
| Job seeker vs Student (health field) | 0·1122 | 0·0517, 0·1728 | <0·001 |
| Unemployed vs Student (health field) | 0·1113 | 0·0517, 0·1709 | <0·001 |
| Other vs Student (health field) | 0·0611 | -0·111, 0·2332 | 1 |
| Unemployed vs Job seeker | -0·0009 | -0·0457, 0·0439 | 1 |
| Other vs Job seeker | -0·0511 | -0·2187, 0·1164 | 1 |
| Other vs Unemployed | -0·0502 | -0·2172, 0·1169 | 1 |
| **Continent of citizenship** |  |  |  |
| Europe (non-Italy) vs Italy | -0·0047 | -0·0574, 0·048 | 1 |
| Africa vs Italy | 0·0314 | -0·0882, 0·1511 | 1 |
| America vs Italy | -0·0251 | -0·1423, 0·0921 | 1 |
| Asia vs Italy | 0·0038 | -0·1363, 0·1438 | 1 |
| Africa vs Europe (non-Italy) | 0·0362 | -0·094, 0·1663 | 1 |
| America vs Europe (non-Italy) | -0·0204 | -0·1483, 0·1076 | 1 |
| Asia vs Europe (non-Italy) | 0·0085 | -0·1402, 0·1573 | 1 |
| America vs Africa | -0·0565 | -0·2236, 0·1105 | 1 |
| Asia vs Africa | -0·0277 | -0·2094, 0·1541 | 1 |
| Asia vs America | 0·0289 | -0·1532, 0·2109 | 1 |
| **Self-identified ethnicity** |  |  |  |
| Multi-ethnic vs European | 0·0379 | -0·0391, 0·115 | 1 |
| North American / Australian vs European | 0·0648 | -0·068, 0·1977 | 1 |
| Arab-Middle Eastern vs European | -0·0097 | -0·1251, 0·1058 | 1 |
| North African vs European | 0·0402 | -0·0708, 0·1512 | 1 |
| Latino-American vs European | -0·0215 | -0·1154, 0·0724 | 1 |
| African American vs European | 0·0974 | -0·1534, 0·3482 | 1 |
| Black African vs European | -0·0310 | -0·186, 0·124 | 1 |
| Asian vs European | -0·0023 | -0·1362, 0·1317 | 1 |
| Pacific Islands vs European | -0·0924 | -0·317, 0·1322 | 1 |
| North American / Australian vs Multi-ethnic | 0·0269 | -0·1246, 0·1784 | 1 |
| Arab-Middle Eastern vs Multi-ethnic | -0·0476 | -0·1809, 0·0857 | 1 |
| North African vs Multi-ethnic | 0·0023 | -0·1256, 0·1302 | 1 |
| Latino-American vs Multi-ethnic | -0·0594 | -0·1776, 0·0588 | 1 |
| African American vs Multi-ethnic | 0·0595 | -0·2013, 0·3203 | 1 |
| Black African vs Multi-ethnic | -0·0689 | -0·2387, 0·1008 | 1 |
| Asian vs Multi-ethnic | -0·0402 | -0·1907, 0·1103 | 1 |
| Pacific Islands vs Multi-ethnic | -0·1303 | -0·3662, 0·1057 | 1 |
| Arab-Middle Eastern vs North American / Australian | -0·0745 | -0·2479, 0·0989 | 1 |
| North African vs North American / Australian | -0·0246 | -0·1952, 0·1459 | 1 |
| Latino-American vs North American / Australian | -0·0863 | -0·2448, 0·0722 | 1 |
| African American vs North American / Australian | 0·0326 | -0·2502, 0·3154 | 1 |
| Black African vs North American / Australian | -0·0958 | -0·2985, 0·1069 | 1 |
| Asian vs North American / Australian | -0·0671 | -0·2548, 0·1206 | 1 |
| Pacific Islands vs North American / Australian | -0·1572 | -0·4166, 0·1022 | 1 |
| North African vs Arab-Middle Eastern | 0·0499 | -0·0982, 0·1979 | 1 |
| Latino-American vs Arab-Middle Eastern | -0·0118 | -0·1585, 0·1349 | 1 |
| African American vs Arab-Middle Eastern | 0·1071 | -0·167, 0·3812 | 1 |
| Black African vs Arab-Middle Eastern | -0·0213 | -0·2062, 0·1636 | 1 |
| Asian vs Arab-Middle Eastern | 0·0074 | -0·1611, 0·1759 | 1 |
| Pacific Islands vs Arab-Middle Eastern | -0·0827 | -0·3325, 0·1671 | 1 |
| Latino-American vs North African | -0·0617 | -0·2049, 0·0816 | 1 |
| African American vs North African | 0·0572 | -0·2141, 0·3286 | 1 |
| Black African vs North African | -0·0712 | -0·2484, 0·106 | 1 |
| Asian vs North African | -0·0425 | -0·2118, 0·1268 | 1 |
| Pacific Islands vs North African | -0·1326 | -0·3805, 0·1153 | 1 |
| African American vs Latino-American | 0·1189 | -0·1483, 0·3862 | 1 |
| Black African vs Latino-American | -0·0095 | -0·1896, 0·1705 | 1 |
| Asian vs Latino-American | 0·0192 | -0·1433, 0·1818 | 1 |
| Pacific Islands vs Latino-American | -0·0709 | -0·3121, 0·1703 | 1 |
| Black African vs African American | -0·1284 | -0·4219, 0·165 | 1 |
| Asian vs African American | -0·0997 | -0·3832, 0·1838 | 1 |
| Pacific Islands vs African American | -0·1898 | -0·5252, 0·1456 | 1 |
| Asian vs Black African | 0·0287 | -0·1745, 0·2319 | 1 |
| Pacific Islands vs Black African | -0·0614 | -0·3325, 0·2097 | 1 |
| Pacific Islands vs Asian | -0·0901 | -0·3483, 0·1681 | 1 |
| **Block 2 - Health-related characteristics and personal experience** | | | |
| **Chronic conditions** |  |  |  |
| One chronic disease vs No chronic disease | -0·0120 | -0·0233, -0·0007 | 0·032 |
| More than one chronic disease vs No chronic disease | -0·0283 | -0·0427, -0·0139 | <0·001 |
| More than one chronic disease vs One chronic disease | -0·0162 | -0·0309, -0·0016 | 0·024 |
| **Block 4 – External influences (perceived vaccination endorsement in the respondent’s community by:)** | | | |
| **By religious leaders** |  |  |  |
| No vs Yes | 0·0487 | 0·0312, 0·0661 | <0·001 |
| Don’t know vs Yes | 0·0010 | -0·0127, 0·0147 | 1 |
| Don’t know vs No | -0·0477 | -0·065, -0·0303 | <0·001 |
| **By political leaders** |  |  |  |
| No vs Yes | 0·0097 | -0·0077, 0·0272 | 0·546 |
| Don’t know vs Yes | -0·0653 | -0·0795, -0·051 | <0·001 |
| Don’t know vs No | -0·0750 | -0·0943, -0·0557 | <0·001 |
| **By teachers** |  |  |  |
| No vs Yes | 0·0598 | 0·0409, 0·0788 | <0·001 |
| Don’t know vs Yes | 0·0505 | 0·0364, 0·0647 | <0·001 |
| Don’t know vs No | -0·0093 | -0·0298, 0·0113 | 0·843 |
| **By health professionals** |  |  |  |
| No vs Yes | 0·0917 | 0·0722, 0·1113 | <0·001 |
| Don’t know vs Yes | 0·1180 | 0·1035, 0·1325 | <0·001 |
| Don’t know vs No | 0·0263 | 0·0043, 0·0482 | 0·013 |
| **Block 4 - External influences** | | | |
| **Use of non-conventional medicine** |  |  |  |
| Yes, integrated with conventional medicine vs No | 0·0348 | 0·0226, 0·047 | <0·001 |
| Yes, as alternative to conventional medicine vs No | 0·1463 | 0·1273, 0·1653 | <0·001 |
| Yes, as alternative to conventional medicine vs Yes, integrated with conventional medicine | 0·1115 | 0·0913, 0·1318 | <0·001 |
| **Political orientation** |  |  |  |
| Extreme left (0) vs Centre (4–6) | -0·0689 | -0·1012, -0·0365 | <0·001 |
| Left (1–3) vs Centre (4–6) | -0·0937 | -0·1132, -0·0742 | <0·001 |
| Right (7–9) vs Centre (4–6) | 0·0078 | -0·0094, 0·025 | 1 |
| Extreme right (10) vs Centre (4–6) | 0·0266 | -0·0073, 0·0604 | 0·357 |
| Non-aligned with traditional parties vs Centre (4–6) | 0·0132 | -0·005, 0·0314 | 0·583 |
| Prefer not to answer vs Centre (4–6) | -0·0119 | -0·0379, 0·0141 | 1 |
| Left (1–3) vs Extreme left (0) | -0·0248 | -0·0588, 0·0091 | 0·55 |
| Right (7–9) vs Extreme left (0) | 0·0766 | 0·0428, 0·1105 | <0·001 |
| Extreme right (10) vs Extreme left (0) | 0·0954 | 0·0511, 0·1398 | <0·001 |
| Non-aligned with traditional parties vs Extreme left (0) | 0·0820 | 0·0484, 0·1156 | <0·001 |
| Prefer not to answer vs Extreme left (0) | 0·0570 | 0·0181, 0·0958 | <0·001 |
| Right (7–9) vs Left (1–3) | 0·1015 | 0·0799, 0·123 | <0·001 |
| Extreme right (10) vs Left (1–3) | 0·1203 | 0·0841, 0·1565 | <0·001 |
| Non-aligned with traditional parties vs Left (1–3) | 0·1069 | 0·085, 0·1287 | <0·001 |
| Prefer not to answer vs Left (1–3) | 0·0818 | 0·053, 0·1106 | <0·001 |
| Extreme right (10) vs Right (7–9) | 0·0188 | -0·0158, 0·0534 | 1 |
| Non-aligned with traditional parties vs Right (7–9) | 0·0054 | -0·0147, 0·0255 | 1 |
| Prefer not to answer vs Right (7–9) | -0·0197 | -0·0466, 0·0072 | 0·554 |
| Non-aligned with traditional parties vs Extreme right (10) | -0·0134 | -0·0488, 0·022 | 1 |
| Prefer not to answer vs Extreme right (10) | -0·0385 | -0·0785, 0·0015 | 0·073 |
| Prefer not to answer vs Non-aligned with traditional parties | -0·0251 | -0·0514, 0·0012 | 0·08 |
| **Religion** |  |  |  |
| Orthodox vs Catholic | -0·0121 | -0·0549, 0·0307 | 1 |
| Protestant vs Catholic | 0·0539 | -0·0213, 0·1291 | 1 |
| Jewish vs Catholic | 0·1121 | -0·0234, 0·2476 | 0·351 |
| Muslim vs Catholic | 0·0385 | -0·0324, 0·1095 | 1 |
| Jehovah’s Witness vs Catholic | 0·0732 | -0·0012, 0·1476 | 0·061 |
| Atheist vs Catholic | -0·0064 | -0·0346, 0·0218 | 1 |
| Agnostic vs Catholic | -0·0072 | -0·0471, 0·0327 | 1 |
| Buddhist vs Catholic | 0·0422 | -0·0516, 0·1361 | 1 |
| Hindu vs Catholic | 0·1434 | -0·0435, 0·3303 | 0·646 |
| Other vs Catholic | 0·0638 | 0·0135, 0·1141 | 0·001 |
| Prefer not to answer vs Catholic | 0·0445 | 0·0113, 0·0777 | <0·001 |
| Protestant vs Orthodox | 0·0660 | -0·0195, 0·1516 | 0·616 |
| Jewish vs Orthodox | 0·1242 | -0·017, 0·2654 | 0·201 |
| Muslim vs Orthodox | 0·0506 | -0·0302, 0·1314 | 1 |
| Jehovah’s Witness vs Orthodox | 0·0853 | 0·0006, 0·17 | 0·046 |
| Atheist vs Orthodox | 0·0057 | -0·0448, 0·0561 | 1 |
| Agnostic vs Orthodox | 0·0049 | -0·0528, 0·0627 | 1 |
| Buddhist vs Orthodox | 0·0543 | -0·0483, 0·157 | 1 |
| Hindu vs Orthodox | 0·1555 | -0·0358, 0·3467 | 0·408 |
| Other vs Orthodox | 0·0759 | 0·0106, 0·1412 | 0·006 |
| Prefer not to answer vs Orthodox | 0·0566 | 0·0038, 0·1094 | 0·02 |
| Jewish vs Protestant | 0·0582 | -0·0952, 0·2116 | 1 |
| Muslim vs Protestant | -0·0154 | -0·1158, 0·085 | 1 |
| Jehovah’s Witness vs Protestant | 0·0193 | -0·0851, 0·1236 | 1 |
| Atheist vs Protestant | -0·0604 | -0·1399, 0·0191 | 0·694 |
| Agnostic vs Protestant | -0·0611 | -0·1454, 0·0231 | 0·959 |
| Buddhist vs Protestant | -0·0117 | -0·1308, 0·1074 | 1 |
| Hindu vs Protestant | 0·0894 | -0·1111, 0·29 | 1 |
| Other vs Protestant | 0·0099 | -0·0794, 0·0991 | 1 |
| Prefer not to answer vs Protestant | -0·0094 | -0·0907, 0·0719 | 1 |
| Muslim vs Jewish | -0·0736 | -0·2234, 0·0761 | 1 |
| Jehovah’s Witness vs Jewish | -0·0389 | -0·1926, 0·1147 | 1 |
| Atheist vs Jewish | -0·1186 | -0·2562, 0·0191 | 0·246 |
| Agnostic vs Jewish | -0·1193 | -0·2595, 0·0209 | 0·275 |
| Buddhist vs Jewish | -0·0699 | -0·2335, 0·0937 | 1 |
| Hindu vs Jewish | 0·0313 | -0·1981, 0·2607 | 1 |
| Other vs Jewish | -0·0483 | -0·1917, 0·0951 | 1 |
| Prefer not to answer vs Jewish | -0·0676 | -0·2065, 0·0713 | 1 |
| Jehovah’s Witness vs Muslim | 0·0347 | -0·0649, 0·1343 | 1 |
| Atheist vs Muslim | -0·0449 | -0·1203, 0·0304 | 1 |
| Agnostic vs Muslim | -0·0457 | -0·126, 0·0346 | 1 |
| Buddhist vs Muslim | 0·0037 | -0·1119, 0·1194 | 1 |
| Hindu vs Muslim | 0·1049 | -0·0928, 0·3026 | 1 |
| Other vs Muslim | 0·0253 | -0·0599, 0·1104 | 1 |
| Prefer not to answer vs Muslim | 0·0060 | -0·071, 0·083 | 1 |
| Atheist vs Jehovah’s Witness | -0·0796 | -0·1587, -0·0006 | 0·045 |
| Agnostic vs Jehovah’s Witness | -0·0804 | -0·1643, 0·0036 | 0·083 |
| Buddhist vs Jehovah’s Witness | -0·0310 | -0·1497, 0·0877 | 1 |
| Hindu vs Jehovah’s Witness | 0·0702 | -0·1301, 0·2705 | 1 |
| Other vs Jehovah’s Witness | -0·0094 | -0·098, 0·0792 | 1 |
| Prefer not to answer vs Jehovah’s Witness | -0·0287 | -0·1093, 0·0519 | 1 |
| Agnostic vs Atheist | -0·0007 | -0·0422, 0·0407 | 1 |
| Buddhist vs Atheist | 0·0487 | -0·0481, 0·1454 | 1 |
| Hindu vs Atheist | 0·1498 | -0·0388, 0·3385 | 0·494 |
| Other vs Atheist | 0·0702 | 0·0157, 0·1247 | 0·001 |
| Prefer not to answer vs Atheist | 0·0510 | 0·0119, 0·0901 | 0·001 |
| Buddhist vs Agnostic | 0·0494 | -0·0513, 0·1501 | 1 |
| Hindu vs Agnostic | 0·1506 | -0·0401, 0·3412 | 0·516 |
| Other vs Agnostic | 0·0710 | 0·0095, 0·1325 | 0·007 |
| Prefer not to answer vs Agnostic | 0·0517 | 0·0032, 0·1002 | 0·022 |
| Hindu vs Buddhist | 0·1012 | -0·1068, 0·3091 | 1 |
| Other vs Buddhist | 0·0216 | -0·0835, 0·1266 | 1 |
| Prefer not to answer vs Buddhist | 0·0023 | -0·0963, 0·1009 | 1 |
| Other vs Hindu | -0·0796 | -0·2722, 0·113 | 1 |
| Prefer not to answer vs Hindu | -0·0989 | -0·2886, 0·0908 | 1 |
| Prefer not to answer vs Other | -0·0193 | -0·0775, 0·0389 | 1 |
| **Importance of religion** |  |  |  |
| Not at all (0) vs Somewhat important (4–6) | -0·0462 | -0·07, -0·0224 | <0·001 |
| Slightly (1–3) vs Somewhat important (4–6) | -0·0186 | -0·0379, 0·0008 | 0·072 |
| Very (7–9) vs Somewhat important (4–6) | -0·0107 | -0·0263, 0·005 | 0·686 |
| Extremely (10) vs Somewhat important (4–6) | -0·0362 | -0·06, -0·0123 | <0·001 |
| Prefer not to answer vs Somewhat important (4–6) | 0·0370 | 0·0039, 0·0701 | 0·015 |
| Slightly (1–3) vs Not at all (0) | 0·0276 | 0·0037, 0·0515 | 0·011 |
| Very (7–9) vs Not at all (0) | 0·0355 | 0·0109, 0·0602 | <0·001 |
| Extremely (10) vs Not at all (0) | 0·0100 | -0·0203, 0·0403 | 1 |
| Prefer not to answer vs Not at all (0) | 0·0832 | 0·0467, 0·1197 | <0·001 |
| Very (7–9) vs Slightly (1–3) | 0·0079 | -0·012, 0·0279 | 1 |
| Extremely (10) vs Slightly (1–3) | -0·0176 | -0·0444, 0·0092 | 0·816 |
| Prefer not to answer vs Slightly (1–3) | 0·0556 | 0·0208, 0·0904 | <0·001 |
| Extremely (10) vs Very (7–9) | -0·0255 | -0·0489, -0·0021 | 0·02 |
| Prefer not to answer vs Very (7–9) | 0·0477 | 0·0151, 0·0802 | <0·001 |
| Prefer not to answer vs Extremely (10) | 0·0732 | 0·0359, 0·1105 | <0·001 |

The table includes refers exclusively to categorical variables with more than two levels. Results are expressed as absolute differences in predicted probability (Δ Pr), accompanied by 95% confidence intervals and adjusted p-values.

*Note: Pairwise comparison p-values are reported as provided by Stata, which displays a maximum of three decimal places. It is not possible to obtain more precise p-values directly.

## Table S10. Unweighted analysis: Pairwise comparisons of predicted probabilities of vaccine hesitancy, calculated after the multiple logistic regression model with Blocks from 1 to 6 (Bonferroni correction)

| **Comparison** | **Δ Pr(hesitancy)** | **95% CI (Bonferroni)** | **p (Bonferroni)*** |
| --- | --- | --- | --- |
| **Block 1 - Sociodemographic and socioeconomic characteristics** |  |  |  |
| **Age group** |  |  |  |
| 30–44 vs 18–29 | 0·0663 | 0·0463, 0·0863 | <0·001 |
| 45–59 vs 18–29 | 0·0793 | 0·0583, 0·1003 | <0·001 |
| 60–74 vs 18–29 | 0·1014 | 0·0752, 0·1276 | <0·001 |
| 75+ vs 18–29 | 0·0590 | 0·0285, 0·0894 | <0·001 |
| 45–59 vs 30–44 | 0·0131 | -0·0038, 0·0299 | 0·298 |
| 60–74 vs 30–44 | 0·0351 | 0·0114, 0·0588 | <0·001 |
| 75+ vs 30–44 | -0·0073 | -0·0359, 0·0213 | 1 |
| 60–74 vs 45–59 | 0·0221 | 0·0019, 0·0423 | 0·022 |
| 75+ vs 45–59 | -0·0204 | -0·046, 0·0053 | 0·259 |
| 75+ vs 60–74 | -0·0424 | -0·0618, -0·023 | <0·001 |
| **Gender** |  |  |  |
| Female vs Male | 0·0023 | -0·0082, 0·0128 | 1 |
| Non-binary/Other vs Male | 0·1341 | 0·0774, 0·1907 | <0·001 |
| Prefer not to answer vs Male | 0·0046 | -0·187, 0·1962 | 1 |
| Non-binary/Other vs Female | 0·1318 | 0·0751, 0·1884 | <0·001 |
| Prefer not to answer vs Female | 0·0023 | -0·1893, 0·1938 | 1 |
| Prefer not to answer vs Non-binary/Other | -0·1295 | -0·3283, 0·0693 | 0·514 |
| **Marital status** |  |  |  |
| Married vs Single | -0·0189 | -0·037, -0·0008 | 0·034 |
| Separated/Divorced vs Single | 0·0199 | -0·0062, 0·0459 | 0·324 |
| Cohabiting vs Single | 0·0062 | -0·0139, 0·0263 | 1 |
| Widowed vs Single | -0·0477 | -0·0789, -0·0166 | <0·001 |
| Separated/Divorced vs Married | 0·0388 | 0·0171, 0·0605 | <0·001 |
| Cohabiting vs Married | 0·0251 | 0·0068, 0·0433 | 0·001 |
| Widowed vs Married | -0·0288 | -0·0555, -0·0022 | 0·024 |
| Cohabiting vs Separated/Divorced | -0·0137 | -0·0399, 0·0125 | 1 |
| Widowed vs Separated/Divorced | -0·0676 | -0·1008, -0·0344 | <0·001 |
| Widowed vs Cohabiting | -0·0539 | -0·0852, -0·0226 | <0·001 |
| **Children** |  |  |  |
| Only children ≤11 years vs No children | -0·0122 | -0·0323, 0·008 | 0·902 |
| Only children 12-18 years vs No children | -0·0209 | -0·0455, 0·0036 | 0·167 |
| Only children >18 years vs No children | -0·0443 | -0·0633, -0·0254 | <0·001 |
| Children of various ages vs No children | -0·0490 | -0·0759, -0·0221 | <0·001 |
| Only children 12-18 years vs Only children ≤11 years | -0·0088 | -0·0352, 0·0177 | 1 |
| Only children >18 years vs Only children ≤11 years | -0·0322 | -0·0552, -0·0091 | 0·001 |
| Children of various ages vs Only children ≤11 years | -0·0368 | -0·0651, -0·0085 | 0·003 |
| Only children >18 years vs Only children 12-18 years | -0·0234 | -0·0485, 0·0017 | 0·088 |
| Children of various ages vs Only children 12-18 years | -0·0280 | -0·0586, 0·0026 | 0·101 |
| Children of various ages vs Only children >18 years | -0·0046 | -0·0316, 0·0224 | 1 |
| **Sexual orientation** |  |  |  |
| Homosexual vs Heterosexual | -0·0400 | -0·0833, 0·0033 | 0·101 |
| Bisexual vs Heterosexual | -0·0461 | -0·0853, -0·0068 | 0·009 |
| Pansexual vs Heterosexual | -0·0147 | -0·0873, 0·0579 | 1 |
| Ace spectrum vs Heterosexual | -0·0224 | -0·0858, 0·041 | 1 |
| Prefer not to answer vs Heterosexual | -0·0748 | -0·1002, -0·0494 | <0·001 |
| Bisexual vs Homosexual | -0·0061 | -0·0631, 0·051 | 1 |
| Pansexual vs Homosexual | 0·0253 | -0·058, 0·1087 | 1 |
| Ace spectrum vs Homosexual | 0·0176 | -0·058, 0·0932 | 1 |
| Prefer not to answer vs Homosexual | -0·0348 | -0·0841, 0·0146 | 0·578 |
| Pansexual vs Bisexual | 0·0314 | -0·0493, 0·1121 | 1 |
| Ace spectrum vs Bisexual | 0·0237 | -0·0491, 0·0964 | 1 |
| Prefer not to answer vs Bisexual | -0·0287 | -0·074, 0·0166 | 0·944 |
| Ace spectrum vs Pansexual | -0·0077 | -0·1012, 0·0857 | 1 |
| Prefer not to answer vs Pansexual | -0·0601 | -0·1357, 0·0155 | 0·293 |
| Prefer not to answer vs Ace spectrum | -0·0524 | -0·1194, 0·0146 | 0·325 |
| **Municipality size (inhabitants)** |  |  |  |
| 10,001–25,000 vs ≤10,000 | -0·0036 | -0·0196, 0·0124 | 1 |
| 25,001–50,000 vs ≤10,000 | -0·0032 | -0·0229, 0·0164 | 1 |
| 50,001–100,000 vs ≤10,000 | 0·0098 | -0·0171, 0·0367 | 1 |
| 100,001–250,000 vs ≤10,000 | 0·0152 | -0·0175, 0·048 | 1 |
| >250,000 vs ≤10,000 | -0·0023 | -0·0317, 0·0271 | 1 |
| 25,001–50,000 vs 10,001–25,000 | 0·0003 | -0·0197, 0·0204 | 1 |
| 50,001–100,000 vs 10,001–25,000 | 0·0134 | -0·0133, 0·04 | 1 |
| 100,001–250,000 vs 10,001–25,000 | 0·0188 | -0·0136, 0·0513 | 1 |
| >250,000 vs 10,001–25,000 | 0·0013 | -0·0278, 0·0303 | 1 |
| 50,001–100,000 vs 25,001–50,000 | 0·0130 | -0·0113, 0·0374 | 1 |
| 100,001–250,000 vs 25,001–50,000 | 0·0185 | -0·0111, 0·0481 | 0·997 |
| >250,000 vs 25,001–50,000 | 0·0009 | -0·0248, 0·0266 | 1 |
| 100,001–250,000 vs 50,001–100,000 | 0·0055 | -0·0217, 0·0326 | 1 |
| >250,000 vs 50,001–100,000 | -0·0121 | -0·0348, 0·0106 | 1 |
| >250,000 vs 100,001–250,000 | -0·0176 | -0·0425, 0·0074 | 0·583 |
| **Geographic macro-area** |  |  |  |
| North-East vs North-West | 0·0037 | -0·0124, 0·0197 | 1 |
| Centre vs North-West | 0·0003 | -0·0156, 0·0161 | 1 |
| South vs North-West | -0·0147 | -0·0305, 0·0012 | 0·093 |
| Islands vs North-West | -0·0270 | -0·0467, -0·0072 | 0·001 |
| Centre vs North-East | -0·0034 | -0·0207, 0·0139 | 1 |
| South vs North-East | -0·0184 | -0·0356, -0·0011 | 0·028 |
| Islands vs North-East | -0·0307 | -0·0514, -0·0099 | <0·001 |
| South vs Centre | -0·0149 | -0·0315, 0·0016 | 0·114 |
| Islands vs Centre | -0·0273 | -0·0475, -0·007 | 0·002 |
| Islands vs South | -0·0123 | -0·0318, 0·0071 | 0·752 |
| **Degree of urbanisation** |  |  |  |
| Intermunicipal pole vs Pole | -0·0072 | -0·045, 0·0306 | 1 |
| Belt vs Pole | 0·0146 | -0·0089, 0·0382 | 1 |
| Intermediate vs Pole | 0·0136 | -0·0133, 0·0404 | 1 |
| Peripheral vs Pole | -0·0032 | -0·034, 0·0276 | 1 |
| Ultra-peripheral vs Pole | -0·0049 | -0·0573, 0·0474 | 1 |
| Belt vs Intermunicipal pole | 0·0218 | -0·0146, 0·0582 | 1 |
| Intermediate vs Intermunicipal pole | 0·0207 | -0·0179, 0·0593 | 1 |
| Peripheral vs Intermunicipal pole | 0·0040 | -0·0372, 0·0452 | 1 |
| Ultra-peripheral vs Intermunicipal pole | 0·0022 | -0·0567, 0·0612 | 1 |
| Intermediate vs Belt | -0·0011 | -0·0184, 0·0162 | 1 |
| Peripheral vs Belt | -0·0178 | -0·0399, 0·0042 | 0·263 |
| Ultra-peripheral vs Belt | -0·0196 | -0·0668, 0·0276 | 1 |
| Peripheral vs Intermediate | -0·0167 | -0·0409, 0·0074 | 0·629 |
| Ultra-peripheral vs Intermediate | -0·0185 | -0·0666, 0·0296 | 1 |
| Ultra-peripheral vs Peripheral | -0·0018 | -0·051, 0·0475 | 1 |
| **Education level** |  |  |  |
| Primary/None vs Upper secondary | -0·0970 | -0·1311, -0·0629 | <0·001 |
| Lower secondary vs Upper secondary | 0·0089 | -0·0073, 0·0251 | 1 |
| University vs Upper secondary | -0·0307 | -0·0441, -0·0172 | <0·001 |
| Postgraduate vs Upper secondary | -0·0618 | -0·0845, -0·0391 | <0·001 |
| Lower secondary vs Primary/None | 0·1060 | 0·0708, 0·1411 | <0·001 |
| University vs Primary/None | 0·0663 | 0·0308, 0·1018 | <0·001 |
| Postgraduate vs Primary/None | 0·0352 | -0·0047, 0·0752 | 0·133 |
| University vs Lower secondary | -0·0396 | -0·0586, -0·0206 | <0·001 |
| Postgraduate vs Lower secondary | -0·0707 | -0·0972, -0·0442 | <0·001 |
| Postgraduate vs University | -0·0311 | -0·0546, -0·0076 | 0·002 |
| **Occupational status** |  |  |  |
| Healthcare worker vs Non-healthcare worker | -0·0611 | -0·09, -0·0323 | <0·001 |
| Homemaker vs Non-healthcare worker | 0·0171 | -0·0081, 0·0422 | 1 |
| Retired vs Non-healthcare worker | -0·0322 | -0·0563, -0·0082 | 0·001 |
| Student (non-health field) vs Non-healthcare worker | -0·0745 | -0·1093, -0·0397 | <0·001 |
| Student (health field) vs Non-healthcare worker | -0·1073 | -0·1571, -0·0575 | <0·001 |
| Job seeker vs Non-healthcare worker | 0·0034 | -0·0306, 0·0373 | 1 |
| Unemployed vs Non-healthcare worker | 0·0041 | -0·0266, 0·0349 | 1 |
| Other vs Non-healthcare worker | -0·0559 | -0·2092, 0·0973 | 1 |
| Homemaker vs Healthcare worker | 0·0782 | 0·0421, 0·1143 | <0·001 |
| Retired vs Healthcare worker | 0·0289 | -0·0065, 0·0644 | 0·329 |
| Student (non-health field) vs Healthcare worker | -0·0133 | -0·0563, 0·0296 | 1 |
| Student (health field) vs Healthcare worker | -0·0462 | -0·1018, 0·0095 | 0·287 |
| Job seeker vs Healthcare worker | 0·0645 | 0·0219, 0·1071 | <0·001 |
| Unemployed vs Healthcare worker | 0·0653 | 0·025, 0·1056 | <0·001 |
| Other vs Healthcare worker | 0·0052 | -0·1502, 0·1606 | 1 |
| Retired vs Homemaker | -0·0493 | -0·0791, -0·0195 | <0·001 |
| Student (non-health field) vs Homemaker | -0·0915 | -0·1325, -0·0506 | <0·001 |
| Student (health field) vs Homemaker | -0·1244 | -0·1785, -0·0702 | <0·001 |
| Job seeker vs Homemaker | -0·0137 | -0·0537, 0·0263 | 1 |
| Unemployed vs Homemaker | -0·0129 | -0·0498, 0·024 | 1 |
| Other vs Homemaker | -0·0730 | -0·2275, 0·0816 | 1 |
| Student (non-health field) vs Retired | -0·0423 | -0·0831, -0·0014 | 0·034 |
| Student (health field) vs Retired | -0·0751 | -0·1292, -0·021 | <0·001 |
| Job seeker vs Retired | 0·0356 | -0·0045, 0·0757 | 0·165 |
| Unemployed vs Retired | 0·0364 | -0·0001, 0·0728 | 0·052 |
| Other vs Retired | -0·0237 | -0·1781, 0·1307 | 1 |
| Student (health field) vs Student (non-health field) | -0·0328 | -0·0881, 0·0224 | 1 |
| Job seeker vs Student (non-health field) | 0·0779 | 0·0336, 0·1221 | <0·001 |
| Unemployed vs Student (non-health field) | 0·0786 | 0·0354, 0·1218 | <0·001 |
| Other vs Student (non-health field) | 0·0186 | -0·1378, 0·1749 | 1 |
| Job seeker vs Student (health field) | 0·1107 | 0·0535, 0·1678 | <0·001 |
| Unemployed vs Student (health field) | 0·1114 | 0·0552, 0·1677 | <0·001 |
| Other vs Student (health field) | 0·0514 | -0·109, 0·2117 | 1 |
| Unemployed vs Job seeker | 0·0008 | -0·042, 0·0435 | 1 |
| Other vs Job seeker | -0·0593 | -0·2154, 0·0968 | 1 |
| Other vs Unemployed | -0·0601 | -0·2156, 0·0955 | 1 |
| **Continent of citizenship** |  |  |  |
| Europe (non-Italy) vs Italy | 0·0023 | -0·0484, 0·053 | 1 |
| Africa vs Italy | 0·0264 | -0·0896, 0·1424 | 1 |
| America vs Italy | -0·0271 | -0·1406, 0·0863 | 1 |
| Asia vs Italy | -0·0094 | -0·15, 0·1311 | 1 |
| Africa vs Europe (non-Italy) | 0·0241 | -0·1017, 0·15 | 1 |
| America vs Europe (non-Italy) | -0·0294 | -0·1533, 0·0945 | 1 |
| Asia vs Europe (non-Italy) | -0·0117 | -0·1605, 0·137 | 1 |
| America vs Africa | -0·0535 | -0·2153, 0·1082 | 1 |
| Asia vs Africa | -0·0358 | -0·2161, 0·1444 | 1 |
| Asia vs America | 0·0177 | -0·1624, 0·1978 | 1 |
| **Self-identified ethnicity** |  |  |  |
| Multi-ethnic vs European | 0·0538 | -0·0254, 0·1329 | 1 |
| North American / Australian vs European | 0·0657 | -0·0602, 0·1915 | 1 |
| Arab-Middle Eastern vs European | 0·0091 | -0·0965, 0·1147 | 1 |
| North African vs European | 0·0483 | -0·058, 0·1546 | 1 |
| Latino-American vs European | -0·0210 | -0·1102, 0·0682 | 1 |
| African American vs European | 0·0255 | -0·2336, 0·2846 | 1 |
| Black African vs European | -0·0297 | -0·1795, 0·1201 | 1 |
| Asian vs European | -0·0106 | -0·1429, 0·1217 | 1 |
| Pacific Islands vs European | -0·0969 | -0·3077, 0·1139 | 1 |
| North American / Australian vs Multi-ethnic | 0·0119 | -0·1349, 0·1587 | 1 |
| Arab-Middle Eastern vs Multi-ethnic | -0·0447 | -0·1715, 0·0821 | 1 |
| North African vs Multi-ethnic | -0·0055 | -0·1321, 0·1212 | 1 |
| Latino-American vs Multi-ethnic | -0·0748 | -0·1914, 0·0418 | 1 |
| African American vs Multi-ethnic | -0·0283 | -0·2975, 0·241 | 1 |
| Black African vs Multi-ethnic | -0·0835 | -0·2497, 0·0828 | 1 |
| Asian vs Multi-ethnic | -0·0644 | -0·2148, 0·086 | 1 |
| Pacific Islands vs Multi-ethnic | -0·1507 | -0·3747, 0·0734 | 1 |
| Arab-Middle Eastern vs North American / Australian | -0·0566 | -0·2184, 0·1053 | 1 |
| North African vs North American / Australian | -0·0173 | -0·1799, 0·1453 | 1 |
| Latino-American vs North American / Australian | -0·0867 | -0·2376, 0·0643 | 1 |
| African American vs North American / Australian | -0·0401 | -0·3264, 0·2461 | 1 |
| Black African vs North American / Australian | -0·0953 | -0·2896, 0·099 | 1 |
| Asian vs North American / Australian | -0·0763 | -0·2578, 0·1053 | 1 |
| Pacific Islands vs North American / Australian | -0·1625 | -0·4071, 0·082 | 1 |
| North African vs Arab-Middle Eastern | 0·0393 | -0·099, 0·1775 | 1 |
| Latino-American vs Arab-Middle Eastern | -0·0301 | -0·1659, 0·1057 | 1 |
| African American vs Arab-Middle Eastern | 0·0164 | -0·2608, 0·2937 | 1 |
| Black African vs Arab-Middle Eastern | -0·0388 | -0·2143, 0·1368 | 1 |
| Asian vs Arab-Middle Eastern | -0·0197 | -0·1817, 0·1423 | 1 |
| Pacific Islands vs Arab-Middle Eastern | -0·1059 | -0·3398, 0·128 | 1 |
| Latino-American vs North African | -0·0694 | -0·2061, 0·0674 | 1 |
| African American vs North African | -0·0228 | -0·3001, 0·2544 | 1 |
| Black African vs North African | -0·0780 | -0·2491, 0·093 | 1 |
| Asian vs North African | -0·0589 | -0·2241, 0·1062 | 1 |
| Pacific Islands vs North African | -0·1452 | -0·3793, 0·0889 | 1 |
| African American vs Latino-American | 0·0465 | -0·2252, 0·3183 | 1 |
| Black African vs Latino-American | -0·0087 | -0·1818, 0·1645 | 1 |
| Asian vs Latino-American | 0·0104 | -0·1482, 0·169 | 1 |
| Pacific Islands vs Latino-American | -0·0759 | -0·3032, 0·1515 | 1 |
| Black African vs African American | -0·0552 | -0·3533, 0·2429 | 1 |
| Asian vs African American | -0·0361 | -0·3259, 0·2537 | 1 |
| Pacific Islands vs African American | -0·1224 | -0·4552, 0·2104 | 1 |
| Asian vs Black African | 0·0191 | -0·179, 0·2171 | 1 |
| Pacific Islands vs Black African | -0·0672 | -0·324, 0·1896 | 1 |
| Pacific Islands vs Asian | -0·0863 | -0·3327, 0·1602 | 1 |
| **Block 2 - Health-related characteristics and personal experience** | | | |
| **Chronic conditions** |  |  |  |
| One chronic disease vs No chronic disease | -0·0084 | -0·0193, 0·0025 | 0·199 |
| More than one chronic disease vs No chronic disease | -0·0265 | -0·0401, -0·0129 | <0·001 |
| More than one chronic disease vs One chronic disease | -0·0182 | -0·0321, -0·0043 | 0·005 |
| **Block 4 – External influences (perceived vaccination endorsement in the respondent’s community by:)** | | | |
| **By religious leaders** |  |  |  |
| No vs Yes | 0·0432 | 0·0266, 0·0598 | <0·001 |
| Don’t know vs Yes | -0·0032 | -0·0165, 0·0102 | 1 |
| Don’t know vs No | -0·0463 | -0·063, -0·0297 | <0·001 |
| **By political leaders** |  |  |  |
| No vs Yes | 0·0093 | -0·0074, 0·0261 | 0·545 |
| Don’t know vs Yes | -0·0667 | -0·0804, -0·053 | <0·001 |
| Don’t know vs No | -0·0760 | -0·0946, -0·0575 | <0·001 |
| **By teachers** |  |  |  |
| No vs Yes | 0·0636 | 0·0456, 0·0816 | <0·001 |
| Don’t know vs Yes | 0·0510 | 0·0374, 0·0647 | <0·001 |
| Don’t know vs No | -0·0126 | -0·0322, 0·007 | 0·374 |
| **By health professionals** |  |  |  |
| No vs Yes | 0·0891 | 0·0704, 0·1078 | <0·001 |
| Don’t know vs Yes | 0·1135 | 0·0995, 0·1275 | <0·001 |
| Don’t know vs No | 0·0244 | 0·0033, 0·0455 | 0·017 |
| **Block 4 - External influences** | | | |
| **Use of non-conventional medicine** |  |  |  |
| Yes, integrated with conventional medicine vs No | 0·0347 | 0·023, 0·0464 | <0·001 |
| Yes, as alternative to conventional medicine vs No | 0·1489 | 0·1314, 0·1664 | <0·001 |
| Yes, as alternative to conventional medicine vs Yes, integrated with conventional medicine | 0·1142 | 0·0955, 0·133 | <0·001 |
| **Political orientation** |  |  |  |
| Extreme left (0) vs Centre (4–6) | -0·0657 | -0·0963, -0·0352 | <0·001 |
| Left (1–3) vs Centre (4–6) | -0·0948 | -0·1135, -0·0762 | <0·001 |
| Right (7–9) vs Centre (4–6) | 0·0070 | -0·0093, 0·0233 | 1 |
| Extreme right (10) vs Centre (4–6) | 0·0257 | -0·0058, 0·0572 | 0·278 |
| Non-aligned with traditional parties vs Centre (4–6) | 0·0128 | -0·0049, 0·0304 | 0·593 |
| Prefer not to answer vs Centre (4–6) | -0·0029 | -0·0283, 0·0226 | 1 |
| Left (1–3) vs Extreme left (0) | -0·0291 | -0·0612, 0·0031 | 0·126 |
| Right (7–9) vs Extreme left (0) | 0·0728 | 0·0411, 0·1045 | <0·001 |
| Extreme right (10) vs Extreme left (0) | 0·0914 | 0·0501, 0·1327 | <0·001 |
| Non-aligned with traditional parties vs Extreme left (0) | 0·0785 | 0·0467, 0·1103 | <0·001 |
| Prefer not to answer vs Extreme left (0) | 0·0629 | 0·0259, 0·0999 | <0·001 |
| Right (7–9) vs Left (1–3) | 0·1019 | 0·0816, 0·1221 | <0·001 |
| Extreme right (10) vs Left (1–3) | 0·1205 | 0·0868, 0·1542 | <0·001 |
| Non-aligned with traditional parties vs Left (1–3) | 0·1076 | 0·0866, 0·1286 | <0·001 |
| Prefer not to answer vs Left (1–3) | 0·0920 | 0·0641, 0·1198 | <0·001 |
| Extreme right (10) vs Right (7–9) | 0·0187 | -0·0135, 0·0509 | 1 |
| Non-aligned with traditional parties vs Right (7–9) | 0·0057 | -0·0135, 0·0249 | 1 |
| Prefer not to answer vs Right (7–9) | -0·0099 | -0·0361, 0·0163 | 1 |
| Non-aligned with traditional parties vs Extreme right (10) | -0·0129 | -0·046, 0·0201 | 1 |
| Prefer not to answer vs Extreme right (10) | -0·0286 | -0·0664, 0·0092 | 0·455 |
| Prefer not to answer vs Non-aligned with traditional parties | -0·0156 | -0·0414, 0·0101 | 1 |
| **Religion** |  |  |  |
| Orthodox vs Catholic | -0·0127 | -0·0541, 0·0287 | 1 |
| Protestant vs Catholic | 0·0641 | -0·0092, 0·1375 | 0·213 |
| Jewish vs Catholic | 0·1202 | -0·0089, 0·2493 | 0·113 |
| Muslim vs Catholic | 0·0307 | -0·0367, 0·0981 | 1 |
| Jehovah’s Witness vs Catholic | 0·0603 | -0·0102, 0·1307 | 0·262 |
| Atheist vs Catholic | -0·0126 | -0·0397, 0·0144 | 1 |
| Agnostic vs Catholic | -0·0134 | -0·0517, 0·0249 | 1 |
| Buddhist vs Catholic | 0·0440 | -0·0458, 0·1339 | 1 |
| Hindu vs Catholic | 0·1392 | -0·0594, 0·3378 | 1 |
| Other vs Catholic | 0·0628 | 0·0123, 0·1132 | 0·002 |
| Prefer not to answer vs Catholic | 0·0413 | 0·0095, 0·0731 | 0·001 |
| Protestant vs Orthodox | 0·0768 | -0·006, 0·1596 | 0·118 |
| Jewish vs Orthodox | 0·1329 | -0·0016, 0·2674 | 0·058 |
| Muslim vs Orthodox | 0·0434 | -0·0334, 0·1201 | 1 |
| Jehovah’s Witness vs Orthodox | 0·0729 | -0·0076, 0·1535 | 0·152 |
| Atheist vs Orthodox | 0·0001 | -0·0483, 0·0485 | 1 |
| Agnostic vs Orthodox | -0·0008 | -0·0563, 0·0548 | 1 |
| Buddhist vs Orthodox | 0·0567 | -0·0416, 0·155 | 1 |
| Hindu vs Orthodox | 0·1519 | -0·0504, 0·3541 | 0·755 |
| Other vs Orthodox | 0·0755 | 0·0112, 0·1398 | 0·005 |
| Prefer not to answer vs Orthodox | 0·0540 | 0·0031, 0·1049 | 0·023 |
| Jewish vs Protestant | 0·0561 | -0·0907, 0·2029 | 1 |
| Muslim vs Protestant | -0·0335 | -0·1301, 0·0632 | 1 |
| Jehovah’s Witness vs Protestant | -0·0039 | -0·104, 0·0963 | 1 |
| Atheist vs Protestant | -0·0767 | -0·1541, 0·0006 | 0·055 |
| Agnostic vs Protestant | -0·0776 | -0·1594, 0·0043 | 0·094 |
| Buddhist vs Protestant | -0·0201 | -0·135, 0·0948 | 1 |
| Hindu vs Protestant | 0·0751 | -0·1358, 0·2859 | 1 |
| Other vs Protestant | -0·0013 | -0·0891, 0·0865 | 1 |
| Prefer not to answer vs Protestant | -0·0228 | -0·1019, 0·0563 | 1 |
| Muslim vs Jewish | -0·0896 | -0·2315, 0·0524 | 1 |
| Jehovah’s Witness vs Jewish | -0·0600 | -0·206, 0·0861 | 1 |
| Atheist vs Jewish | -0·1328 | -0·264, -0·0017 | 0·043 |
| Agnostic vs Jewish | -0·1337 | -0·2675, 0·0001 | 0·051 |
| Buddhist vs Jewish | -0·0762 | -0·2323, 0·0799 | 1 |
| Hindu vs Jewish | 0·0190 | -0·2167, 0·2547 | 1 |
| Other vs Jewish | -0·0574 | -0·1951, 0·0802 | 1 |
| Prefer not to answer vs Jewish | -0·0789 | -0·2112, 0·0534 | 1 |
| Jehovah’s Witness vs Muslim | 0·0296 | -0·0653, 0·1245 | 1 |
| Atheist vs Muslim | -0·0433 | -0·1146, 0·028 | 1 |
| Agnostic vs Muslim | -0·0441 | -0·1204, 0·0322 | 1 |
| Buddhist vs Muslim | 0·0133 | -0·0969, 0·1236 | 1 |
| Hindu vs Muslim | 0·1085 | -0·0995, 0·3165 | 1 |
| Other vs Muslim | 0·0321 | -0·0501, 0·1144 | 1 |
| Prefer not to answer vs Muslim | 0·0106 | -0·0625, 0·0838 | 1 |
| Atheist vs Jehovah’s Witness | -0·0729 | -0·1477, 0·002 | 0·069 |
| Agnostic vs Jehovah’s Witness | -0·0737 | -0·1533, 0·0059 | 0·12 |
| Buddhist vs Jehovah’s Witness | -0·0162 | -0·1295, 0·097 | 1 |
| Hindu vs Jehovah’s Witness | 0·0789 | -0·131, 0·2889 | 1 |
| Other vs Jehovah’s Witness | 0·0025 | -0·0829, 0·088 | 1 |
| Prefer not to answer vs Jehovah’s Witness | -0·0190 | -0·0954, 0·0575 | 1 |
| Agnostic vs Atheist | -0·0008 | -0·041, 0·0393 | 1 |
| Buddhist vs Atheist | 0·0566 | -0·0361, 0·1494 | 1 |
| Hindu vs Atheist | 0·1518 | -0·0483, 0·3519 | 0·7 |
| Other vs Atheist | 0·0754 | 0·0212, 0·1296 | <0·001 |
| Prefer not to answer vs Atheist | 0·0539 | 0·0167, 0·0911 | <0·001 |
| Buddhist vs Agnostic | 0·0574 | -0·0391, 0·154 | 1 |
| Hindu vs Agnostic | 0·1526 | -0·0492, 0·3545 | 0·718 |
| Other vs Agnostic | 0·0762 | 0·0155, 0·137 | 0·002 |
| Prefer not to answer vs Agnostic | 0·0547 | 0·0083, 0·1012 | 0·005 |
| Hindu vs Buddhist | 0·0952 | -0·1216, 0·312 | 1 |
| Other vs Buddhist | 0·0188 | -0·083, 0·1205 | 1 |
| Prefer not to answer vs Buddhist | -0·0027 | -0·0971, 0·0917 | 1 |
| Other vs Hindu | -0·0764 | -0·2805, 0·1277 | 1 |
| Prefer not to answer vs Hindu | -0·0979 | -0·2987, 0·1029 | 1 |
| Prefer not to answer vs Other | -0·0215 | -0·079, 0·0361 | 1 |
| **Importance of religion** |  |  |  |
| Not at all (0) vs Somewhat important (4–6) | -0·0413 | -0·0644, -0·0183 | <0·001 |
| Slightly (1–3) vs Somewhat important (4–6) | -0·0166 | -0·0352, 0·0019 | 0·129 |
| Very (7–9) vs Somewhat important (4–6) | -0·0124 | -0·0274, 0·0025 | 0·215 |
| Extremely (10) vs Somewhat important (4–6) | -0·0272 | -0·0499, -0·0045 | 0·007 |
| Prefer not to answer vs Somewhat important (4–6) | 0·0400 | 0·0096, 0·0703 | 0·002 |
| Slightly (1–3) vs Not at all (0) | 0·0247 | 0·0015, 0·048 | 0·027 |
| Very (7–9) vs Not at all (0) | 0·0289 | 0·0052, 0·0526 | 0·005 |
| Extremely (10) vs Not at all (0) | 0·0142 | -0·0148, 0·0431 | 1 |
| Prefer not to answer vs Not at all (0) | 0·0813 | 0·0475, 0·1151 | <0·001 |
| Very (7–9) vs Slightly (1–3) | 0·0042 | -0·0149, 0·0233 | 1 |
| Extremely (10) vs Slightly (1–3) | -0·0106 | -0·0361, 0·015 | 1 |
| Prefer not to answer vs Slightly (1–3) | 0·0566 | 0·0245, 0·0887 | <0·001 |
| Extremely (10) vs Very (7–9) | -0·0147 | -0·037, 0·0076 | 0·785 |
| Prefer not to answer vs Very (7–9) | 0·0524 | 0·0226, 0·0822 | <0·001 |
| Prefer not to answer vs Extremely (10) | 0·0671 | 0·0327, 0·1016 | <0·001 |

The table includes refers exclusively to categorical variables with more than two levels. Results are expressed as absolute differences in predicted probability (Δ Pr), accompanied by 95% confidence intervals and adjusted p-values.

*Note: Pairwise comparison p-values are reported as provided by Stata, which displays a maximum of three decimal places. It is not possible to obtain more precise p-values directly.

## Table S11. Post-stratification weighted analysis: Hierarchical linear regression model for “Lack of trust” subscale

| **LACK of TRUST** | **Univariable regression** | | **Multiple regression** | | **Multiple regression** | |
| --- | --- | --- | --- | --- | --- | --- |
|  |  |  | **BLOCK 1 [F(105, 51040)=374·23, p<0·0001; adjR2=0·334]** | | **BLOCK 2 [F(106, 51040)=675·97, p<0·0001); adjR2=0·480)** | |
| **Variable** | **Coef (95%CI)** | **p** | **adjCoef (95% CI)** | **p** | **adjCoef (95% CI)** | **p** |
|  |  |  |  |  |  |  |
| Age group |  |  |  |  |  |  |
| 18–29 | Ref. |  | Ref. |  | Ref. |  |
| 30–44 | 1·5 (1·29; 1·71) | <0·0001 | 1·23 (1·02; 1·44) | <0·0001 | 0·81 (0·62; 1·01) | <0·0001 |
| 45–59 | 1·07 (0·87; 1·26) | <0·0001 | 1·5 (1·28; 1·72) | <0·0001 | 0·92 (0·71; 1·13) | <0·0001 |
| 60–74 | -0·16 (-0·36; 0·05) | 0·14 | 1·74 (1·45; 2·03) | <0·0001 | 0·95 (0·69; 1·21) | <0·0001 |
| 75+ | -1·52 (-1·75; -1·29) | <0·0001 | 1·06 (0·72; 1·4) | <0·0001 | 0·94 (0·64; 1·25) | <0·0001 |
| Gender |  |  |  |  |  |  |
| Male | Ref. |  | Ref. |  | Ref. |  |
| Female | 0·28 (0·15; 0·42) | <0·0001 | -0·11 (-0·22; 0·01) | 0·071 | -0·08 (-0·18; 0·02) | 0·13 |
| Non-binary/Other | 3·05 (2·39; 3·71) | <0·0001 | 1·73 (1·13; 2·32) | <0·0001 | 1·16 (0·6; 1·72) | <0·0001 |
| Prefer not to answer | 3·09 (0·53; 5·64) | 0·018 | 1·29 (-0·45; 3·03) | 0·15 | 1·16 (-0·54; 2·85) | 0·18 |
| Marital status |  |  |  |  |  |  |
| Single | Ref. |  | Ref. |  | Ref. |  |
| Married | -1·23 (-1·39; -1·07) | <0·0001 | -0·32 (-0·51; -0·12) | 0·0014 | -0·29 (-0·47; -0·12) | 0·0011 |
| Separated/Divorced | 0·94 (0·63; 1·25) | <0·0001 | 0·55 (0·26; 0·85) | 0·00022 | 0·35 (0·09; 0·61) | 0·0074 |
| Cohabiting | 0·28 (0·04; 0·51) | 0·022 | 0·1 (-0·11; 0·32) | 0·35 | -0·02 (-0·21; 0·17) | 0·81 |
| Widowed | -2·58 (-2·91; -2·25) | <0·0001 | -0·42 (-0·76; -0·08) | 0·015 | -0·37 (-0·66; -0·08) | 0·013 |
| Children |  |  |  |  |  |  |
| No children | Ref. |  | Ref. |  | Ref. |  |
| Only children ≤11 years | 0·03 (-0·18; 0·24) | 0·81 | -0·34 (-0·55; -0·14) | 0·0012 | -0·28 (-0·47; -0·09) | 0·0036 |
| Only children 12-18 years | 0·26 (-0·01; 0·54) | 0·061 | -0·55 (-0·81; -0·29) | <0·0001 | -0·59 (-0·83; -0·35) | <0·0001 |
| Only children >18 years | -1·82 (-1·97; -1·67) | <0·0001 | -0·63 (-0·84; -0·42) | <0·0001 | -0·58 (-0·77; -0·4) | <0·0001 |
| Children of various ages | -0·49 (-0·8; -0·18) | 0·0021 | -0·75 (-1·03; -0·47) | <0·0001 | -0·69 (-0·94; -0·44) | <0·0001 |
| Sexual orientation |  |  |  |  |  |  |
| Heterosexual | Ref. |  | Ref. |  | Ref. |  |
| Homosexual | 0·33 (-0·19; 0·85) | 0·21 | -0·4 (-0·86; 0·06) | 0·088 | -0·3 (-0·69; 0·09) | 0·14 |
| Bisexual | 0·22 (-0·23; 0·67) | 0·34 | -0·72 (-1·11; -0·34) | 0·00024 | -0·32 (-0·66; 0·03) | 0·069 |
| Pansexual | 2·37 (1·66; 3·07) | <0·0001 | -0·66 (-1·26; -0·06) | 0·031 | 0·03 (-0·56; 0·62) | 0·93 |
| Ace spectrum | 2·6 (1·99; 3·21) | <0·0001 | -0·81 (-1·41; -0·21) | 0·0082 | -0·16 (-0·77; 0·45) | 0·61 |
| Prefer not to answer | -0·48 (-0·76; -0·21) | 0·00064 | -1·54 (-1·83; -1·26) | <0·0001 | -1·28 (-1·55; -1·01) | <0·0001 |
| Municipality size (inhabitants) |  |  |  |  |  |  |
| ≤10,000 | Ref. |  | Ref. |  | Ref. |  |
| 10,001–25,000 | -0·47 (-0·65; -0·29) | <0·0001 | -0·08 (-0·23; 0·08) | 0·35 | -0·02 (-0·16; 0·12) | 0·78 |
| 25,001–50,000 | -0·71 (-0·91; -0·51) | <0·0001 | -0·12 (-0·31; 0·07) | 0·21 | -0·1 (-0·26; 0·07) | 0·26 |
| 50,001–100,000 | -0·12 (-0·35; 0·11) | 0·32 | 0·11 (-0·15; 0·38) | 0·41 | 0·03 (-0·21; 0·26) | 0·81 |
| 100,001–250,000 | 0·34 (0·08; 0·61) | 0·012 | 0·1 (-0·22; 0·42) | 0·55 | 0·15 (-0·14; 0·43) | 0·31 |
| >250,000 | -0·21 (-0·43; 0) | 0·053 | -0·11 (-0·4; 0·18) | 0·47 | -0·18 (-0·44; 0·08) | 0·18 |
| Geographic macro-area |  |  |  |  |  |  |
| North-West | Ref. |  | Ref. |  | Ref. |  |
| North-East | -0·12 (-0·31; 0·07) | 0·23 | 0·28 (0·11; 0·44) | 0·001 | 0·22 (0·08; 0·36) | 0·0027 |
| Centre | -0·31 (-0·51; -0·12) | 0·0017 | -0·05 (-0·22; 0·11) | 0·52 | -0·01 (-0·16; 0·13) | 0·86 |
| South | -0·08 (-0·26; 0·11) | 0·41 | -0·43 (-0·6; -0·27) | <0·0001 | -0·44 (-0·59; -0·3) | <0·0001 |
| Islands | -0·28 (-0·52; -0·05) | 0·019 | -0·57 (-0·79; -0·35) | <0·0001 | -0·56 (-0·76; -0·37) | <0·0001 |
| Degree of urbanisation |  |  |  |  |  |  |
| Pole | Ref. |  | Ref. |  | Ref. |  |
| Intermunicipal pole | -0·45 (-0·87; -0·04) | 0·033 | -0·19 (-0·57; 0·18) | 0·31 | -0·15 (-0·49; 0·19) | 0·39 |
| Belt | 0·08 (-0·07; 0·23) | 0·31 | 0·22 (-0·01; 0·44) | 0·061 | 0·24 (0·03; 0·44) | 0·022 |
| Intermediate | -0·23 (-0·44; -0·03) | 0·026 | 0·16 (-0·1; 0·43) | 0·22 | 0·29 (0·06; 0·53) | 0·014 |
| Peripheral | -0·38 (-0·64; -0·13) | 0·0031 | 0·16 (-0·14; 0·46) | 0·302 | 0·26 (-0·01; 0·52) | 0·056 |
| Ultra-peripheral | 0·09 (-0·47; 0·65) | 0·76 | 0·07 (-0·44; 0·58) | 0·79 | 0·14 (-0·29; 0·57) | 0·53 |
| Education level |  |  |  |  |  |  |
| Upper secondary | Ref. |  | Ref. |  | Ref. |  |
| Primary/None | -2·09 (-2·54; -1·65) | <0·0001 | -0·94 (-1·32; -0·56) | <0·0001 | -0·69 (-1·06; -0·33) | 0·00021 |
| Lower secondary | -0·06 (-0·26; 0·14) | 0·57 | 0·27 (0·09; 0·45) | 0·0033 | 0·09 (-0·07; 0·25) | 0·29 |
| University | -0·97 (-1·13; -0·82) | <0·0001 | -0·41 (-0·55; -0·27) | <0·0001 | -0·17 (-0·3; -0·05) | 0·0048 |
| Postgraduate | -0·88 (-1·17; -0·6) | <0·0001 | -0·57 (-0·81; -0·33) | <0·0001 | -0·29 (-0·49; -0·08) | 0·0063 |
| Occupational status |  |  |  |  |  |  |
| Non-healthcare worker | Ref. |  | Ref. |  | Ref. |  |
| Healthcare worker | -1·55 (-1·84; -1·26) | <0·0001 | -1·22 (-1·46; -0·98) | <0·0001 | -0·86 (-1·08; -0·64) | <0·0001 |
| Homemaker | 0·42 (0·16; 0·69) | 0·00196 | 0·22 (-0·01; 0·46) | 0·065 | 0·04 (-0·18; 0·25) | 0·74 |
| Retired | -2·14 (-2·3; -1·99) | <0·0001 | -0·48 (-0·71; -0·24) | <0·0001 | 0·03 (-0·17; 0·24) | 77 |
| Student (non-health field) | -1·55 (-1·84; -1·25) | <0·0001 | -0·81 (-1·11; -0·51) | <0·0001 | -0·42 (-0·69; -0·15) | 0·0023 |
| Student (health field) | -2·45 (-2·88; -2·03) | <0·0001 | -1·42 (-1·82; -1·02) | <0·0001 | -0·73 (-1·08; -0·37) | <0·0001 |
| Job seeker | 1 (0·63; 1·36) | <0·0001 | 0·1 (-0·21; 0·42) | 0·52 | 0·1 (-0·18; 0·39) | 0·48 |
| Unemployed | 1·64 (1·29; 1·99) | <0·0001 | 0·21 (-0·09; 0·51) | 0·17 | 0·11 (-0·17; 0·39) | 0·43 |
| Other | 0·78 (-1·06; 2·62) | 0·41 | 0·44 (-1·14; 2·01) | 0·59 | 0·58 (-0·86; 2·02) | 0·43 |
| Continent of citizenship |  |  |  |  |  |  |
| Italy | Ref. |  | Ref. |  | Ref. |  |
| Europe (non-Italy) | 0·37 (-0·24; 0·97) | 0·24 | 0·11 (-0·42; 0·64) | 0·69 | 0·25 (-0·24; 0·74) | 0·31 |
| Africa | 1·61 (0·29; 2·92) | 0·016 | 2·11 (0·82; 3·39) | 0·0013 | 2·56 (1·19; 3·93) | 0·00024 |
| America | 0·12 (-1·04; 1·28) | 0·84 | -0·12 (-1·28; 1·04) | 0·84 | 0·43 (-0·65; 1·51) | 0·44 |
| Asia | 0·11 (-1·27; 1·49) | 0·88 | 1·08 (-0·35; 2·51) | 0·14 | 0·95 (-0·38; 2·28) | 0·16 |
| Oceania | 1·71 (1·65; 1·78) | <0·0001 | -9·14 (-10·62; -7·66) | <0·0001 | -8·06 (-9·44; -6·69) | <0·0001 |
| Self-identified ethnicity |  |  |  |  |  |  |
| European | Ref. |  | Ref. |  | Ref. |  |
| Multi-ethnic | 2·39 (1·69; 3·09) | <0·0001 | 0·12 (-0·53; 0·77) | 0·71 | 0·24 (-0·42; 0·9) | 0·47 |
| North American / Australian | 3·53 (2·26; 4·8) | <0·0001 | 1·04 (-0·09; 2·18) | 0·072 | 0·77 (-0·28; 1·83) | 0·15 |
| Arab-Middle Eastern | 2·33 (1·46; 3·2) | <0·0001 | -0·37 (-1·22; 0·47) | 0·39 | -0·16 (-1·04; 0·71) | 0·71 |
| North African | 0·28 (-0·57; 1·14) | 0·51 | -0·7 (-1·59; 0·18) | 0·12 | -0·75 (-1·6; 0·1) | 0·082 |
| Latino-American | 1·34 (0·57; 2·11) | 0·0007 | -0·1 (-0·89; 0·69) | 0·804 | -0·07 (-0·83; 0·69) | 0·85 |
| African American | 3·34 (1·42; 5·26) | 0·00066 | -0·11 (-2·03; 1·81) | 0·91 | 0·22 (-1·69; 2·13) | 0·82 |
| Black African | 0·55 (-0·9; 2) | 0·46 | -0·88 (-2·24; 0·48) | 0·21 | -1·13 (-2·53; 0·26) | 0·11 |
| Asian | 0·41 (-0·67; 1·49) | 0·46 | -0·78 (-1·91; 0·34) | 0·17 | -0·56 (-1·7; 0·58) | 0·34 |
| Pacific Islands | 1·42 (-1·17; 4·01) | 0·28 | -0·76 (-2·93; 1·41) | 0·49 | -0·5 (-2·89; 1·89) | 0·68 |
| Material deprivation |  |  |  |  |  |  |
| No deprivation | Ref. |  | Ref. |  | Ref. |  |
| Severe deprivation | 1·8 (1·44; 2·17) | <0·0001 | 0·2 (-0·1; 0·51) | 0·19 | -0·14 (-0·4; 0·12) | 0·29 |
| Chronic conditions |  |  |  |  |  |  |
| No chronic disease | Ref. |  | Ref. |  | Ref. |  |
| One chronic disease | -0·13 (-0·28; 0·02) | 0·081 | -0·27 (-0·4; -0·14) | <0·0001 | -0·22 (-0·34; -0·1) | 0·00029 |
| More than one chronic disease | -0·97 (-1·15; -0·79) | <0·0001 | -0·52 (-0·7; -0·35) | <0·0001 | -0·57 (-0·72; -0·42) | <0·0001 |
| Living with a person with disability |  |  |  |  |  |  |
| No | Ref. |  | Ref. |  | Ref. |  |
| Yes | 0·65 (0·47; 0·82) | <0·0001 | -0·19 (-0·34; -0·03) | 0·017 | -0·13 (-0·27; 0) | 0·055 |
| Inadequate health literacy |  |  |  |  |  |  |
| No | Ref. |  | Ref. |  | Ref. |  |
| Yes | -0·39 (-0·52; -0·26) | <0·0001 | -0·38 (-0·5; -0·26) | <0·0001 | -0·57 (-0·67; -0·46) | <0·0001 |
| Knowing someone who had AEFI |  |  |  |  |  |  |
| No | Ref. |  | Ref. |  | Ref. |  |
| Yes | 5·3 (5·15; 5·44) | <0·0001 | 4·2 (4·05; 4·34) | <0·0001 | 2·08 (1·95; 2·21) | <0·0001 |
| Knowing someone who had VPD |  |  |  |  |  |  |
| No | Ref. |  | Ref. |  | Ref. |  |
| Yes | -0·65 (-0·81; -0·5) | <0·0001 | -1·95 (-2·09; -1·8) | <0·0001 | -1·15 (-1·27; -1·02) | <0·0001 |
| Reported barriers to vaccination |  |  |  |  |  |  |
| No | Ref. |  | Ref. |  | Ref. |  |
| Yes | 1·8 (1·67; 1·93) | <0·0001 | 0·33 (0·21; 0·45) | <0·0001 | -0·12 (-0·23; -0·01) | 0·027 |
| Information source cluster |  |  |  |  |  |  |
| Diversified sources | Ref. |  | Ref. |  | Ref. |  |
| Professional-only sources | -2·49 (-2·62; -2·36) | <0·0001 | -0·47 (-0·59; -0·34) | <0·0001 | -0·19 (-0·29; -0·08) | 0·00045 |
| Trust in sources | -4·14 (-4·24; -4·04) | <0·0001 | -2·38 (-2·49; -2·28) | <0·0001 | -1·57 (-1·66; -1·47) | <0·0001 |
| By religious leaders |  |  |  |  |  |  |
| Yes | Ref. |  | Ref. |  | Ref. |  |
| No | 2·25 (2·06; 2·45) | <0·0001 | 0·24 (0·05; 0·44) | 0·015 | -0·11 (-0·29; 0·07) | 0·24 |
| Don’t know | 0·29 (0·13; 0·45) | 0·00034 | -0·18 (-0·34; -0·02) | 0·026 | -0·23 (-0·37; -0·1) | 0·00079 |
| By political leaders |  |  |  |  |  |  |
| Yes | Ref. |  | Ref. |  | Ref. |  |
| No | 2·02 (1·83; 2·2) | <0·0001 | -0·38 (-0·58; -0·19) | 0·00014 | -0·19 (-0·37; 0) | 0·047 |
| Don’t know | -0·28 (-0·42; -0·13) | 0·00019 | -1·18 (-1·34; -1·02) | <0·0001 | -0·51 (-0·65; -0·38) | <0·0001 |
| By teachers |  |  |  |  |  |  |
| Yes | Ref. |  | Ref. |  | Ref. |  |
| No | 3·7 (3·51; 3·89) | <0·0001 | 0·89 (0·68; 1·11) | <0·0001 | 0·47 (0·27; 0·67) | <0·0001 |
| Don’t know | 1·44 (1·3; 1·58) | <0·0001 | 1·03 (0·87; 1·19) | <0·0001 | 0·79 (0·66; 0·92) | <0·0001 |
| By health professionals |  |  |  |  |  |  |
| Yes | Ref. |  | Ref. |  | Ref. |  |
| No | 4 (3·8; 4·2) | <0·0001 | 1·21 (0·99; 1·43) | <0·0001 | 0·59 (0·38; 0·79) | <0·0001 |
| Don’t know | 2·37 (2·21; 2·52) | <0·0001 | 1·48 (1·31; 1·64) | <0·0001 | 0·64 (0·5; 0·78) | <0·0001 |
| Use of non-conventional medicine |  |  |  |  |  |  |
| No | Ref. |  | Ref. |  | Ref. |  |
| Yes, integrated with conventional medicine | 1·21 (1·06; 1·36) | <0·0001 | 0·26 (0·12; 0·4) | 0·00028 | -0·09 (-0·21; 0·04) | 0·17 |
| Yes, as alternative to conventional medicine | 4·55 (4·31; 4·8) | <0·0001 | 2·4 (2·17; 2·62) | <0·0001 | 1·18 (0·97; 1·38) | <0·0001 |
| Political orientation |  |  |  |  |  |  |
| Right (7–9) | Ref. |  | Ref. |  | Ref. |  |
| Centre (4–6) | 0·62 (0·45; 0·8) | <0·0001 | -0·1 (-0·25; 0·06) | 0·21 | 0·28 (0·14; 0·41) | <0·0001 |
| Extreme left (0) | 0·64 (0·24; 1·04) | 0·0018 | -0·76 (-1·1; -0·42) | <0·0001 | 0·15 (-0·15; 0·46) | 0·33 |
| Left (1–3) | -1·2 (-1·41; -0·99) | <0·0001 | -1·12 (-1·31; -0·93) | <0·0001 | -0·04 (-0·21; 0·13) | 0·64 |
| Extreme right (10) | 1·59 (1·18; 2) | <0·0001 | 0·89 (0·53; 1·26) | <0·0001 | 0·07 (-0·26; 0·4) | 0·66 |
| Non-aligned with traditional parties | 1·38 (1·16; 1·6) | <0·0001 | 0·67 (0·48; 0·86) | <0·0001 | 0·77 (0·61; 0·93) | <0·0001 |
| Prefer not to answer | -1·99 (-2·24; -1·74) | <0·0001 | -0·15 (-0·38; 0·08) | 0·21 | 0·54 (0·34; 0·74) | <0·0001 |
| Religion |  |  |  |  |  |  |
| Catholic | Ref. |  | Ref. |  | Ref. |  |
| Orthodox | -1 (-1·35; -0·65) | <0·0001 | -0·61 (-0·95; -0·28) | 0·00032 | -0·63 (-0·94; -0·32) | <0·0001 |
| Protestant | 2·76 (2·02; 3·49) | <0·0001 | 0·86 (0·23; 1·49) | 0·0071 | 0·82 (0·23; 1·41) | 0·0063 |
| Jewish | 3·51 (2·35; 4·67) | <0·0001 | 1·58 (0·64; 2·51) | 0·00096 | 1·89 (1·01; 2·77) | <0·0001 |
| Muslim | 1·59 (1·04; 2·13) | <0·0001 | 0·84 (0·27; 1·42) | 0·00395 | 0·44 (-0·14; 1·03) | 0·13 |
| Jehovah’s Witness | 2·71 (2·05; 3·38) | <0·0001 | 0·56 (-0·05; 1·18) | 0·073 | 0·71 (0·15; 1·26) | 0·012 |
| Atheist | 0·28 (0·05; 0·5) | 0·015 | -0·01 (-0·26; 0·23) | 0·92 | 0·07 (-0·15; 0·28) | 0·54 |
| Agnostic | -0·17 (-0·54; 0·19) | 0·35 | -0·1 (-0·43; 0·22) | 0·53 | 0·15 (-0·12; 0·43) | 0·27 |
| Buddhist | 3·71 (2·7; 4·72) | <0·0001 | 1·43 (0·64; 2·23) | 0·0004 | 1·08 (0·39; 1·76) | 0·00203 |
| Hindu | 5·22 (3·34; 7·1) | <0·0001 | 2·39 (0·85; 3·93) | 0·0023 | 1·23 (-0·32; 2·78) | 0·12 |
| Other | 4·05 (3·49; 4·62) | <0·0001 | 1·75 (1·28; 2·21) | <0·0001 | 1·3 (0·89; 1·7) | <0·0001 |
| Prefer not to answer | 2·15 (1·83; 2·47) | <0·0001 | 0·82 (0·54; 1·11) | <0·0001 | 0·55 (0·29; 0·81) | <0·0001 |
| Importance of religion |  |  |  |  |  |  |
| Not at all (0) | Ref. |  | Ref. |  | Ref. |  |
| Slightly (1–3) | 0·14 (-0·13; 0·4) | 0·31 | 0·25 (0·01; 0·49) | 0·041 | 0·02 (-0·19; 0·23) | 0·85 |
| Somewhat important (4–6) | 0·17 (-0·06; 0·4) | 0·15 | -0·08 (-0·33; 0·16) | 0·501 | -0·42 (-0·64; -0·21) | 0·00011 |
| Very (7–9) | -1·54 (-1·77; -1·32) | <0·0001 | -0·14 (-0·4; 0·11) | 0·27 | -0·68 (-0·9; -0·46) | <0·0001 |
| Extremely (10) | -0·85 (-1·17; -0·53) | <0·0001 | -0·16 (-0·48; 0·16) | 0·34 | -0·94 (-1·23; -0·66) | <0·0001 |
| Prefer not to answer | -0·18 (-0·56; 0·19) | 0·34 | 0·17 (-0·21; 0·54) | 0·38 | -0·82 (-1·16; -0·48) | <0·0001 |
| Perceived NHS quality | -1·14 (-1·18; -1·11) | <0·0001 | -0·45 (-0·5; -0·41) | <0·0001 | -0·3 (-0·34; -0·26) | <0·0001 |
| Perceived NHS access | -1·01 (-1·04; -0·97) | <0·0001 | -0·3 (-0·34; -0·26) | <0·0001 | -0·31 (-0·35; -0·27) | <0·0001 |
| Survey mode |  |  |  |  |  |  |
| CAWI | Ref. |  | Ref. |  | Ref. |  |
| CATI | -2·81 (-2·95; -2·67) | <0·0001 | -0·87 (-1·05; -0·7) | <0·0001 | 0·37 (0·22; 0·52) | <0·0001 |
| VCBS | 2·54 (2·51; 2·58) | <0·0001 |  |  | 1·95 (1·91; 1·99) | <0·0001 |

Abbreviations: adjCoef adjusted coefficient, CATI Computer Assisted Telephone Interviewing, CAWI Computer Assisted Web Interviewing, CI Confidence Interval, NHS National Health Service, VPD Vaccine Preventable Disease.

Note: Regression estimates should not be interpreted as causal effects.

## Table S12. Post-stratification weighted analysis: Hierarchical linear regression model for “Risk perception” subscale

| **RISK PERCEPTION** | **Univariable regression** | | **Multiple regression** | | **Multiple regression** | |
| --- | --- | --- | --- | --- | --- | --- |
|  |  |  | **BLOCK 1 [F(105, 51040)=120·12, p<0·0001; adjR2=0·197]** | | **BLOCK 2 [F(106, 51040)=220·34, p<0·0001; adjR2=0·335)** | |
| **Variable** | **Coef (95%CI)** | **p** | **adjCoef (95% CI)** | **p** | **adjCoef (95% CI)** | **p** |
| Age group |  |  |  |  |  |  |
| 18–29 | Ref. |  | Ref. |  | Ref. |  |
| 30–44 | 0·44 (0·35; 0·54) | <0·0001 | 0·29 (0·19; 0·4) | <0·0001 | 0·11 (0·02; 0·2) | 0·016 |
| 45–59 | 0·35 (0·26; 0·43) | <0·0001 | 0·44 (0·34; 0·55) | <0·0001 | 0·19 (0·09; 0·28) | 0·00014 |
| 60–74 | 0·36 (0·26; 0·45) | <0·0001 | 0·61 (0·47; 0·75) | <0·0001 | 0·26 (0·14; 0·38) | <0·0001 |
| 75+ | 0·96 (0·84; 1·08) | <0·0001 | 0·82 (0·65; 0·99) | <0·0001 | 0·77 (0·62; 0·92) | <0·0001 |
| Gender |  |  |  |  |  |  |
| Male | Ref. |  | Ref. |  | Ref. |  |
| Female | 0·34 (0·28; 0·4) | <0·0001 | 0·34 (0·28; 0·4) | <0·0001 | 0·35 (0·3; 0·4) | <0·0001 |
| Non-binary/Other | 0·28 (-0·01; 0·56) | 0·055 | -0·53 (-0·82; -0·24) | 0·00035 | -0·78 (-1·05; -0·5) | <0·0001 |
| Prefer not to answer | -0·97 (-1·89; -0·04) | 0·0404 | -1·34 (-2·28; -0·4) | 0·0052 | -1·4 (-2·27; -0·53) | 0·0016 |
| Marital status |  |  |  |  |  |  |
| Single | Ref. |  | Ref. |  | Ref. |  |
| Married | 0·29 (0·22; 0·36) | <0·0001 | -0·03 (-0·12; 0·07) | 0·595 | -0·01 (-0·09; 0·07) | 0·75 |
| Separated/Divorced | 0·42 (0·3; 0·55) | <0·0001 | 0·01 (-0·13; 0·14) | 0·93 | -0·08 (-0·2; 0·04) | 0·18 |
| Cohabiting | 0·37 (0·27; 0·47) | <0·0001 | 0·15 (0·05; 0·25) | 0·0044 | 0·09 (0; 0·18) | 0·042 |
| Widowed | 0·56 (0·39; 0·73) | <0·0001 | -0·09 (-0·27; 0·09) | 0·31 | -0·07 (-0·25; 0·1) | 0·43 |
| Children |  |  |  |  |  |  |
| No children | Ref. |  | Ref. |  | Ref. |  |
| Only children ≤11 years | 0·34 (0·25; 0·44) | <0·0001 | 0·07 (-0·03; 0·17) | 0·16 | 0·1 (0·01; 0·19) | 0·026 |
| Only children 12-18 years | 0·49 (0·37; 0·61) | <0·0001 | 0·11 (-0·01; 0·24) | 0·077 | 0·09 (-0·02; 0·2) | 0·091 |
| Only children >18 years | 0·24 (0·17; 0·31) | <0·0001 | -0·25 (-0·35; -0·15) | <0·0001 | -0·23 (-0·32; -0·14) | <0·0001 |
| Children of various ages | 0·26 (0·13; 0·4) | 0·00013 | 0·03 (-0·11; 0·17) | 0·68 | 0·05 (-0·06; 0·17) | 0·37 |
| Sexual orientation |  |  |  |  |  |  |
| Heterosexual | Ref. |  | Ref. |  | Ref. |  |
| Homosexual | -0·48 (-0·71; -0·24) | <0·0001 | -0·27 (-0·48; -0·05) | 0·016 | -0·22 (-0·4; -0·04) | 0·019 |
| Bisexual | -0·38 (-0·57; -0·18) | 0·00021 | -0·21 (-0·4; -0·02) | 0·032 | -0·03 (-0·19; 0·13) | 0·74 |
| Pansexual | -0·23 (-0·54; 0·08) | 0·15 | -0·47 (-0·76; -0·17) | 0·00199 | -0·16 (-0·44; 0·12) | 0·25 |
| Ace spectrum | 0·36 (0·09; 0·62) | 0·0077 | -0·11 (-0·39; 0·17) | 0·45 | 0·18 (-0·07; 0·43) | 0·16 |
| Prefer not to answer | 1·88 (1·76; 2) | <0·0001 | 1·16 (1·02; 1·29) | <0·0001 | 1·27 (1·14; 1·41) | <0·0001 |
| Municipality size (inhabitants) |  |  |  |  |  |  |
| ≤10,000 | Ref. |  | Ref. |  | Ref. |  |
| 10,001–25,000 | -0·25 (-0·33; -0·17) | <0·0001 | -0·01 (-0·08; 0·07) | 0·88 | 0·02 (-0·05; 0·09) | 0·58 |
| 25,001–50,000 | -0·25 (-0·34; -0·16) | <0·0001 | 0·01 (-0·08; 0·1) | 0·84 | 0·02 (-0·07; 0·11) | 0·63 |
| 50,001–100,000 | -0·09 (-0·2; 0·01) | 0·084 | 0·11 (-0·02; 0·25) | 0·094 | 0·08 (-0·05; 0·2) | 0·22 |
| 100,001–250,000 | 0·03 (-0·09; 0·14) | 0·68 | 0·14 (-0·02; 0·3) | 0·094 | 0·16 (0·01; 0·31) | 0·035 |
| >250,000 | -0·19 (-0·29; -0·09) | 0·00013 | 0·09 (-0·06; 0·23) | 0·25 | 0·05 (-0·08; 0·19) | 0·43 |
| Geographic macro-area |  |  |  |  |  |  |
| North-West | Ref. |  | Ref. |  | Ref. |  |
| North-East | -0·04 (-0·13; 0·04) | 0·31 | -0·07 (-0·15; 0·01) | 0·09 | -0·1 (-0·17; -0·02) | 0·012 |
| Centre | 0·07 (-0·02; 0·16) | 0·13 | 0·21 (0·13; 0·29) | <0·0001 | 0·23 (0·15; 0·3) | <0·0001 |
| South | 0·15 (0·07; 0·24) | 0·00046 | 0·09 (0·01; 0·17) | 0·036 | 0·08 (0·01; 0·16) | 0·0298 |
| Islands | -0·28 (-0·39; -0·17) | <0·0001 | -0·29 (-0·4; -0·18) | <0·0001 | -0·29 (-0·39; -0·19) | <0·0001 |
| Degree of urbanisation |  |  |  |  |  |  |
| Pole | Ref. |  | Ref. |  | Ref. |  |
| Intermunicipal pole | 0·08 (-0·12; 0·27) | 0·43 | 0·12 (-0·07; 0·31) | 0·22 | 0·14 (-0·03; 0·31) | 0·12 |
| Belt | 0·01 (-0·06; 0·08) | 0·81 | 0 (-0·11; 0·12) | 0·98 | 0·01 (-0·1; 0·12) | 0·85 |
| Intermediate | -0·01 (-0·11; 0·08) | 0·76 | -0·08 (-0·22; 0·05) | 0·22 | -0·03 (-0·15; 0·1) | 0·67 |
| Peripheral | 0·09 (-0·03; 0·2) | 0·15 | -0·01 (-0·16; 0·15) | 0·94 | 0·04 (-0·1; 0·18) | 0·59 |
| Ultra-peripheral | 0·32 (0·06; 0·58) | 0·017 | -0·02 (-0·28; 0·25) | 0·89 | 0·01 (-0·23; 0·25) | 0·92 |
| Education level |  |  |  |  |  |  |
| Upper secondary | Ref. |  | Ref. |  | Ref. |  |
| Primary/None | 0·17 (-0·07; 0·4) | 0·16 | -0·51 (-0·73; -0·28) | <0·0001 | -0·39 (-0·61; -0·18) | 0·00032 |
| Lower secondary | 0·75 (0·65; 0·84) | <0·0001 | 0·33 (0·24; 0·42) | <0·0001 | 0·25 (0·16; 0·33) | <0·0001 |
| University | -0·59 (-0·66; -0·51) | <0·0001 | -0·36 (-0·42; -0·29) | <0·0001 | -0·25 (-0·31; -0·19) | <0·0001 |
| Postgraduate | -0·46 (-0·59; -0·33) | <0·0001 | -0·37 (-0·49; -0·25) | <0·0001 | -0·25 (-0·35; -0·15) | <0·0001 |
| Occupational status |  |  |  |  |  |  |
| Non-healthcare worker | Ref. |  | Ref. |  | Ref. |  |
| Healthcare worker | -0·24 (-0·38; -0·1) | 0·00096 | -0·28 (-0·41; -0·15) | <0·0001 | -0·12 (-0·23; 0) | 0·047 |
| Homemaker | 0·29 (0·18; 0·41) | <0·0001 | -0·08 (-0·19; 0·03) | 0·18 | -0·16 (-0·26; -0·06) | 0·0015 |
| Retired | 0·39 (0·32; 0·47) | <0·0001 | 0·03 (-0·08; 0·14) | 0·56 | 0·26 (0·16; 0·36) | <0·0001 |
| Student (non-health field) | -0·81 (-0·95; -0·67) | <0·0001 | -0·62 (-0·77; -0·47) | <0·0001 | -0·44 (-0·58; -0·31) | <0·0001 |
| Student (health field) | -0·94 (-1·17; -0·72) | <0·0001 | -0·83 (-1·04; -0·62) | <0·0001 | -0·52 (-0·71; -0·34) | <0·0001 |
| Job seeker | 0·28 (0·13; 0·43) | 0·00023 | -0·02 (-0·17; 0·13) | 0·79 | -0·02 (-0·15; 0·11) | 0·76 |
| Unemployed | 0·4 (0·26; 0·55) | <0·0001 | 0 (-0·14; 0·14) | 0·99 | -0·04 (-0·16; 0·08) | 0·48 |
| Other | 0·23 (-0·4; 0·85) | 0·48 | -0·12 (-0·72; 0·49) | 0·71 | -0·05 (-0·55; 0·45) | 0·84 |
| Continent of citizenship |  |  |  |  |  |  |
| Italy | Ref. |  | Ref. |  | Ref. |  |
| Europe (non-Italy) | 0·1 (-0·17; 0·37) | 0·46 | -0·41 (-0·67; -0·14) | 0·0032 | -0·34 (-0·59; -0·09) | 0·0067 |
| Africa | -0·57 (-1·07; -0·06) | 0·028 | -0·38 (-1·01; 0·25) | 0·24 | -0·18 (-0·74; 0·38) | 0·53 |
| America | -0·25 (-0·79; 0·29) | 0·36 | -0·33 (-1; 0·34) | 0·34 | -0·08 (-0·7; 0·53) | 0·79 |
| Asia | 0·08 (-0·47; 0·62) | 0·78 | 0·53 (-0·11; 1·17) | 0·11 | 0·47 (-0·12; 1·06) | 0·12 |
| Oceania | 0·03 (0; 0·06) | 0·027 | -2·04 (-2·74; -1·35) | <0·0001 | -1·56 (-2·22; -0·91) | <0·0001 |
| Self-identified ethnicity |  |  |  |  |  |  |
| European | Ref. |  | Ref. |  | Ref. |  |
| Multi-ethnic | 0·01 (-0·3; 0·32) | 0·93 | -0·45 (-0·75; -0·14) | 0·0045 | -0·39 (-0·66; -0·13) | 0·00396 |
| North American / Australian | 0·34 (-0·21; 0·9) | 0·22 | -0·4 (-0·95; 0·15) | 0·15 | -0·52 (-1·05; 0) | 0·0504 |
| Arab-Middle Eastern | 0·01 (-0·4; 0·42) | 0·97 | -0·57 (-1·01; -0·14) | 0·01001 | -0·48 (-0·86; -0·1) | 0·013 |
| North African | -0·09 (-0·48; 0·3) | 0·65 | -0·29 (-0·73; 0·14) | 0·18 | -0·32 (-0·69; 0·05) | 0·094 |
| Latino-American | 0·06 (-0·28; 0·4) | 73 | -0·32 (-0·72; 0·09) | 0·12 | -0·31 (-0·68; 0·06) | 0·11 |
| African American | 0·51 (-0·36; 1·38) | 0·25 | -0·35 (-1·2; 0·49) | 0·41 | -0·21 (-0·99; 0·57) | 0·5998 |
| Black African | -0·33 (-0·97; 0·31) | 0·31 | -0·24 (-0·96; 0·47) | 0·51 | -0·36 (-1·02; 0·31) | 0·29 |
| Asian | -0·09 (-0·59; 0·41) | 0·72 | -0·7 (-1·29; -0·11) | 0·0195 | -0·6 (-1·12; -0·09) | 0·022 |
| Pacific Islands | -0·17 (-1·23; 0·9) | 0·76 | -1·33 (-2·49; -0·18) | 0·024 | -1·22 (-2·22; -0·22) | 0·017 |
| Material deprivation |  |  |  |  |  |  |
| No deprivation | Ref. |  | Ref. |  | Ref. |  |
| Severe deprivation | 0·16 (0·02; 0·31) | 0·031 | 0·03 (-0·11; 0·18) | 0·65 | -0·12 (-0·24; 0·01) | 0·061 |
| Chronic conditions |  |  |  |  |  |  |
| No chronic disease | Ref. |  | Ref. |  | Ref. |  |
| One chronic disease | 0·18 (0·11; 0·25) | <0·0001 | -0·06 (-0·12; 0·01) | 0·095 | -0·03 (-0·09; 0·03) | 0·27 |
| More than one chronic disease | 1·12 (1·04; 1·21) | <0·0001 | 0·58 (0·5; 0·67) | <0·0001 | 0·57 (0·48; 0·65) | <0·0001 |
| Living with a person with disability |  |  |  |  |  |  |
| No | Ref. |  | Ref. |  | Ref. |  |
| Yes | 0·49 (0·41; 0·56) | <0·0001 | 0·05 (-0·02; 0·12) | 0·17 | 0·08 (0·01; 0·14) | 0·026 |
| Inadequate health literacy |  |  |  |  |  |  |
| No | Ref. |  | Ref. |  | Ref. |  |
| Yes | 0·86 (0·8; 0·92) | <0·0001 | 0·58 (0·52; 0·63) | <0·0001 | 0·49 (0·44; 0·55) | <0·0001 |
| Knowing someone who had AEFI |  |  |  |  |  |  |
| No | Ref. |  | Ref. |  | Ref. |  |
| Yes | 1·81 (1·75; 1·88) | <0·0001 | 1·44 (1·38; 1·51) | <0·0001 | 0·5 (0·44; 0·57) | <0·0001 |
| Knowing someone who had VPD |  |  |  |  |  |  |
| No | Ref. |  | Ref. |  | Ref. |  |
| Yes | 0·22 (0·15; 0·3) | <0·0001 | -0·42 (-0·49; -0·35) | <0·0001 | -0·07 (-0·13; 0) | 0·047 |
| Reported barriers to vaccination |  |  |  |  |  |  |
| No | Ref. |  | Ref. |  | Ref. |  |
| Yes | 1·06 (1; 1·12) | <0·0001 | 0·45 (0·39; 0·51) | <0·0001 | 0·25 (0·2; 0·31) | <0·0001 |
| Information source cluster |  |  |  |  |  |  |
| Diversified sources | Ref. |  | Ref. |  | Ref. |  |
| Professional-only sources | -1 (-1·06; -0·93) | <0·0001 | -0·51 (-0·57; -0·45) | <0·0001 | -0·38 (-0·44; -0·33) | <0·0001 |
| Trust in sources | -1·02 (-1·06; -0·97) | <0·0001 | -0·47 (-0·52; -0·42) | <0·0001 | -0·11 (-0·16; -0·06) | <0·0001 |
| By religious leaders |  |  |  |  |  |  |
| Yes | Ref. |  | Ref. |  | Ref. |  |
| No | 0·62 (0·53; 0·7) | <0·0001 | 0·34 (0·25; 0·44) | <0·0001 | 0·19 (0·1; 0·27) | <0·0001 |
| Don’t know | -0·01 (-0·09; 0·06) | 0·69 | 0·22 (0·14; 0·3) | <0·0001 | 0·2 (0·13; 0·27) | <0·0001 |
| By political leaders |  |  |  |  |  |  |
| Yes | Ref. |  | Ref. |  | Ref. |  |
| No | 0·37 (0·28; 0·45) | <0·0001 | -0·26 (-0·35; -0·16) | <0·0001 | -0·17 (-0·26; -0·08) | 0·00014 |
| Don’t know | -0·25 (-0·31; -0·18) | <0·0001 | -0·48 (-0·56; -0·4) | <0·0001 | -0·19 (-0·25; -0·12) | <0·0001 |
| By teachers |  |  |  |  |  |  |
| Yes | Ref. |  | Ref. |  | Ref. |  |
| No | 0·78 (0·69; 0·86) | <0·0001 | -0·02 (-0·12; 0·08) | 0·74 | -0·2 (-0·3; -0·11) | <0·0001 |
| Don’t know | 0·08 (0·02; 0·15) | 0·014 | -0·26 (-0·34; -0·18) | <0·0001 | -0·37 (-0·44; -0·3) | <0·0001 |
| By health professionals |  |  |  |  |  |  |
| Yes | Ref. |  | Ref. |  | Ref. |  |
| No | 1·02 (0·93; 1·1) | <0·0001 | 0·32 (0·21; 0·42) | <0·0001 | 0·04 (-0·05; 0·13) | 0·402 |
| Don’t know | 0·81 (0·74; 0·88) | <0·0001 | 0·74 (0·66; 0·82) | <0·0001 | 0·37 (0·3; 0·44) | <0·0001 |
| Use of non-conventional medicine |  |  |  |  |  |  |
| No | Ref. |  | Ref. |  | Ref. |  |
| Yes, integrated with conventional medicine | 0·31 (0·24; 0·38) | <0·0001 | -0·02 (-0·09; 0·05) | 0·56 | -0·17 (-0·23; -0·11) | <0·0001 |
| Yes, as alternative to conventional medicine | 1·43 (1·33; 1·52) | <0·0001 | 0·67 (0·57; 0·77) | <0·0001 | 0·13 (0·04; 0·22) | 0·0037 |
| Political orientation |  |  |  |  |  |  |
| Right (7–9) | Ref. |  | Ref. |  | Ref. |  |
| Centre (4–6) | -0·47 (-0·55; -0·39) | <0·0001 | -0·35 (-0·42; -0·27) | <0·0001 | -0·18 (-0·25; -0·11) | <0·0001 |
| Extreme left (0) | -1·16 (-1·33; -1) | <0·0001 | -0·79 (-0·95; -0·62) | <0·0001 | -0·38 (-0·53; -0·24) | <0·0001 |
| Left (1–3) | -1·46 (-1·56; -1·36) | <0·0001 | -0·89 (-0·99; -0·79) | <0·0001 | -0·41 (-0·5; -0·32) | <0·0001 |
| Extreme right (10) | 0·53 (0·36; 0·7) | <0·0001 | 0·56 (0·39; 0·72) | <0·0001 | 0·19 (0·05; 0·34) | 0·0101 |
| Non-aligned with traditional parties | 0·19 (0·09; 0·28) | 0·00015 | 0·33 (0·24; 0·43) | <0·0001 | 0·38 (0·29; 0·46) | <0·0001 |
| Prefer not to answer | -0·9 (-1·03; -0·77) | <0·0001 | -0·32 (-0·44; -0·19) | <0·0001 | -0·01 (-0·13; 0·11) | 0·85 |
| Religion |  |  |  |  |  |  |
| Catholic | Ref. |  | Ref. |  | Ref. |  |
| Orthodox | 1·51 (1·35; 1·68) | <0·0001 | 0·64 (0·47; 0·8) | <0·0001 | 0·63 (0·46; 0·79) | <0·0001 |
| Protestant | 0·06 (-0·23; 0·35) | 0·69 | -0·22 (-0·51; 0·06) | 0·13 | -0·24 (-0·5; 0·02) | 0·067 |
| Jewish | -0·36 (-0·88; 0·15) | 0·17 | -0·62 (-1·15; -0·09) | 0·022 | -0·48 (-0·97; 0·01) | 0·056 |
| Muslim | 0·21 (-0·03; 0·45) | 0·082 | 0·29 (0·01; 0·56) | 0·042 | 0·11 (-0·13; 0·35) | 0·37 |
| Jehovah’s Witness | 0·7 (0·45; 0·95) | <0·0001 | 0·08 (-0·18; 0·34) | 0·55 | 0·14 (-0·11; 0·39) | 0·26 |
| Atheist | -0·81 (-0·91; -0·72) | <0·0001 | -0·16 (-0·28; -0·04) | 0·0066 | -0·12 (-0·23; -0·02) | 0·015 |
| Agnostic | -0·92 (-1·07; -0·76) | <0·0001 | -0·14 (-0·3; 0·01) | 0·067 | -0·03 (-0·16; 0·1) | 0·67 |
| Buddhist | 0·21 (-0·17; 0·6) | 0·28 | -0·06 (-0·42; 0·3) | 0·74 | -0·22 (-0·54; 0·1) | 0·18 |
| Hindu | 1·11 (0·36; 1·87) | 0·0039 | 0·41 (-0·29; 1·11) | 0·25 | -0·1 (-0·72; 0·52) | 0·75 |
| Other | 0·52 (0·3; 0·74) | <0·0001 | 0·29 (0·08; 0·5) | 0·0069 | 0·09 (-0·08; 0·26) | 0·301 |
| Prefer not to answer | 0·23 (0·1; 0·36) | 0·00079 | -0·09 (-0·24; 0·05) | 0·198 | -0·21 (-0·35; -0·08) | 0·0015 |
| Importance of religion |  |  |  |  |  |  |
| Not at all (0) | Ref. |  | Ref. |  | Ref. |  |
| Slightly (1–3) | 0·34 (0·23; 0·45) | <0·0001 | 0·2 (0·09; 0·32) | 0·00056 | 0·1 (0; 0·2) | 0·0499 |
| Somewhat important (4–6) | 1·03 (0·93; 1·12) | <0·0001 | 0·47 (0·35; 0·59) | <0·0001 | 0·32 (0·22; 0·42) | <0·0001 |
| Very (7–9) | 0·84 (0·74; 0·94) | <0·0001 | 0·43 (0·3; 0·55) | <0·0001 | 0·19 (0·08; 0·29) | 0·00068 |
| Extremely (10) | 1·03 (0·89; 1·17) | <0·0001 | 0·45 (0·3; 0·6) | <0·0001 | 0·1 (-0·03; 0·24) | 0·13 |
| Prefer not to answer | 1·79 (1·63; 1·96) | <0·0001 | 1·41 (1·22; 1·6) | <0·0001 | 0·98 (0·8; 1·16) | <0·0001 |
| Perceived NHS quality | -0·14 (-0·15; -0·12) | <0·0001 | -0·04 (-0·06; -0·02) | <0·0001 | 0·03 (0·01; 0·04) | 0·0036 |
| Perceived NHS access | -0·1 (-0·12; -0·09) | <0·0001 | 0·03 (0·01; 0·05) | 0·0071 | 0·02 (0·01; 0·04) | 0·0104 |
| Survey mode |  |  |  |  |  |  |
| CAWI | Ref. |  | Ref. |  | Ref. |  |
| CATI | 0·1 (0·03; 0·17) | 0·0068 | -0·19 (-0·27; -0·1) | <0·0001 | 0·36 (0·29; 0·44) | <0·0001 |
| VCBS | 0·92 (0·91; 0·94) | <0·0001 |  |  | 0·86 (0·84; 0·88) | <0·0001 |

Abbreviations: adjCoef adjusted coefficient, AEFI Adverse Event Following Immunization, CATI Computer Assisted Telephone Interviewing, CAWI Computer Assisted Web Interviewing, CI Confidence Interval, NHS National Health Service, VPD Vaccine Preventable Disease.

Note: Regression estimates should not be interpreted as causal effects.

## Table S13. Unweighted analysis: Hierarchical linear regression model for “Lack of trust” subscale

| **LACK of TRUST** | **Univariable regression** |  | **Multiple regression** | | | **Multiple regression** | | |
| --- | --- | --- | --- | --- | --- | --- | --- | --- |
|  |  |  | **BLOCK 1 (R-squared=0.3344)** | | | **BLOCK 2 (R-squared=0.4805)** | | |
| **Variable** | **Coef (95%CI)** | **p** | **adjCoef (95% CI)** | **p** | **Effect**  **Size*** | **adjCoef (95% CI)** | **p** | **Effect**  **Size*** |
| Age group |  |  |  |  | 0·0050 |  |  | 0·0020 |
| 18–29 | Ref. |  |  |  |  |  |  |  |
| 30–44 | 1·53 (1·33; 1·74) | <0·0001 | 1·24 (1·03; 1·44) | <0·0001 |  | 0·83 (0·64; 1·03) | <0·0001 |  |
| 45–59 | 1·13 (0·93; 1·32) | <0·0001 | 1·5 (1·28; 1·72) | <0·0001 |  | 0·93 (0·73; 1·14) | <0·0001 |  |
| 60–74 | -0·11 (-0·32; 0·09) | 0·28 | 1·76 (1·48; 2·05) | <0·0001 |  | 0·98 (0·72; 1·23) | <0·0001 |  |
| 75+ | -1·71 (-1·92; -1·51) | <0·0001 | 1·03 (0·71; 1·35) | <0·0001 |  | 1·07 (0·78; 1·36) | <0·0001 |  |
| Gender |  |  |  |  | 0·0009 |  |  | 0·0005 |
| Male | Ref. |  | Ref. |  |  | Ref. |  |  |
| Female | 0·2 (0·07; 0·33) | 0·0025 | -0·15 (-0·26; -0·04) | 0·0097 |  | -0·12 (-0·22; -0·02) | 0·018 |  |
| Non-binary/Other | 3·07 (2·42; 3·71) | <0·0001 | 1·77 (1·18; 2·35) | <0·0001 |  | 1·11 (0·56; 1·65) | <0·0001 |  |
| Prefer not to answer | 2·74 (0·15; 5·34) | 0·038 | 1·39 (-0·55; 3·34) | 0·16 |  | 1·13 (-0·62; 2·89) | 0·21 |  |
| Marital status |  |  |  |  | 0·0017 |  |  | 0·0012 |
| Single | Ref. |  | Ref. |  |  | Ref. |  |  |
| Married | -1·31 (-1·47; -1·15) | <0·0001 | -0·36 (-0·55; -0·17) | 0·00021 |  | -0·3 (-0·47; -0·12) | 0·00072 |  |
| Separated/Divorced | 0·95 (0·66; 1·25) | <0·0001 | 0·55 (0·27; 0·84) | 0·00015 |  | 0·37 (0·12; 0·62) | 0·0034 |  |
| Cohabiting | 0·23 (0; 0·45) | 0·052 | 0·07 (-0·14; 0·28) | 0·495 |  | -0·01 (-0·2; 0·17) | 0·89 |  |
| Widowed | -2·76 (-3·06; -2·46) | <0·0001 | -0·58 (-0·89; -0·26) | 0·00038 |  | -0·45 (-0·73; -0·18) | 0·0011 |  |
| Children |  |  |  |  | 0·0012 |  |  | 0·0015 |
| No children | Ref. |  | Ref. |  |  | Ref. |  |  |
| Only children ≤11 years | -0·06 (-0·27; 0·14) | 0·54 | -0·38 (-0·58; -0·18) | 0·00026 |  | -0·35 (-0·54; -0·16) | 0·00023 |  |
| Only children 12-18 years | 0·19 (-0·08; 0·46) | 0·17 | -0·58 (-0·83; -0·32) | <0·0001 |  | -0·65 (-0·88; -0·42) | <0·0001 |  |
| Only children >18 years | -1·96 (-2·1; -1·81) | <0·0001 | -0·68 (-0·88; -0·47) | <0·0001 |  | -0·62 (-0·8; -0·45) | <0·0001 |  |
| Children of various ages | -0·52 (-0·83; -0·21) | 0·0085 | -0·76 (-1·04; -0·49) | <0·0001 |  | -0·73 (-0·98; -0·49) | <0·0001 |  |
| Sexual orientation |  |  |  |  | 0·0037 |  |  | 0·0033 |
| Heterosexual | Ref. |  | Ref. |  |  | Ref. |  |  |
| Homosexual | 0·34 (-0·16; 0·85) | 0·19 | -0·52 (-0·94; -0·09) | 0·018 |  | -0·32 (-0·71; 0·06) | 0·098 |  |
| Bisexual | 0·09 (-0·35; 0·53) | 0·69 | -0·76 (-1·14; -0·38) | <0·0001 |  | -0·33 (-0·66; 0·01) | 0·061 |  |
| Pansexual | 2·31 (1·62; 3) | <0·0001 | -0·72 (-1·32; -0·12) | 0·019 |  | -0·05 (-0·64; 0·53) | 0·85 |  |
| Ace spectrum | 2·55 (1·94; 3·16) | <0·0001 | -0·9 (-1·48; -0·33) | 0·0022 |  | -0·21 (-0·8; 0·38) | 0·49 |  |
| Prefer not to answer | -0·75 (-1·01; -0·49) | <0·0001 | -1·72 (-1·99; -1·45) | <0·0001 |  | -1·48 (-1·73; -1·23) | <0·0001 |  |
| Municipality size (inhabitants) |  |  |  |  | 0·0002 |  |  | 0·0002 |
| ≤10,000 | Ref. |  | Ref. |  |  | Ref. |  |  |
| 10,001–25,000 | -0·38 (-0·56; -0·2) | <0·0001 | -0·06 (-0·21; 0·1) | 0·49 |  | 0·02 (-0·12; 0·15) | 0·82 |  |
| 25,001–50,000 | -0·45 (-0·65; -0·25) | <0·0001 | -0·13 (-0·31; 0·06) | 0·18 |  | -0·11 (-0·27; 0·06) | 0·201 |  |
| 50,001–100,000 | 0·35 (0·12; 0·57) | 0·0027 | 0·13 (-0·13; 0·39) | 0·34 |  | 0·07 (-0·17; 0·3) | 0·58 |  |
| 100,001–250,000 | 0·67 (0·41; 0·93) | <0·0001 | 0·05 (-0·26; 0·36) | 0·75 |  | 0·14 (-0·14; 0·42) | 0·33 |  |
| >250,000 | 0·24 (0·04; 0·44) | 0·018 | -0·1 (-0·38; 0·18) | 0·48 |  | -0·11 (-0·37; 0·14) | 0·38 |  |
| Geographic macro-area |  |  |  |  | 0·0018 |  |  | 0·0019 |
| North-West | Ref. |  | Ref. |  |  | Ref. |  |  |
| North-East | -0·2 (-0·39; -0·01) | 0·038 | 0·23 (0·07; 0·39) | 0·0402 |  | 0·17 (0·03; 0·31) | 0·017 |  |
| Centre | -0·38 (-0·57; -0·19) | <0·0001 | -0·1 (-0·26; 0·06) | 0·202 |  | -0·04 (-0·18; 0·1) | 0·56 |  |
| South | -0·1 (-0·28; 0·08) | 0·28 | -0·48 (-0·64; -0·32) | <0·0001 |  | -0·45 (-0·59; -0·31) | <0·0001 |  |
| Islands | -0·28 (-0·51; -0·05) | 0·017 | -0·56 (-0·77; -0·36) | <0·0001 |  | -0·52 (-0·7; -0·34) | <0·0001 |  |
| Degree of urbanisation |  |  |  |  | 0·0002 |  |  | 0·0002 |
| Pole | Ref. |  | Ref. |  |  | Ref. |  |  |
| Intermunicipal pole | -0·6 (-1·01; -0·19) | 0·0044 | -0·22 (-0·59; 0·14) | 0·23 |  | -0·14 (-0·48; 0·19) | 0·397 |  |
| Belt | -0·18 (-0·33; -0·03) | 0·018 | 0·22 (0; 0·44) | 0·053 |  | 0·23 (0·03; 0·43) | 0·022 |  |
| Intermediate | -0·58 (-0·78; -0·38) | <0·0001 | 0·14 (-0·12; 0·4) | 0·28 |  | 0·28 (0·05; 0·51) | 0·018 |  |
| Peripheral | -0·77 (-1·01; -0·52) | <0·0001 | 0·14 (-0·15; 0·43) | 0·36 |  | 0·24 (-0·01; 0·5) | 0·064 |  |
| Ultra-peripheral | -0·29 (-0·83; 0·24) | 0·29 | 0·14 (-0·36; 0·63) | 0·59 |  | 0·19 (-0·22; 0·61) | 0·37 |  |
| Education level |  |  |  |  | 0·0017 |  |  | 0·0005 |
| Upper secondary | Ref. |  | Ref. |  |  | Ref. |  |  |
| Primary/None | -2·17 (-2·58; -1·76) | <0·0001 | -0·74 (-1·09; -0·39) | <0·0001 |  | -0·53 (-0·86; -0·21) | 0·0013 |  |
| Lower secondary | -0·12 (-0·31; 0·07) | 0·23 | 0·29 (0·12; 0·47) | 0·00081 |  | 0·11 (-0·04; 0·26) | 0·16 |  |
| University | -0·89 (-1·04; -0·73) | <0·0001 | -0·36 (-0·49; -0·23) | <0·0001 |  | -0·14 (-0·26; -0·02) | 0·021 |  |
| Postgraduate | -0·85 (-1·12; -0·58) | <0·0001 | -0·55 (-0·78; -0·32) | <0·0001 |  | -0·28 (-0·48; -0·08) | 0·00703 |  |
| Occupational status |  |  |  |  | 0·0034 |  |  | 0·0015 |
| Non-healthcare worker | Ref. |  | Ref. |  |  | Ref. |  |  |
| Healthcare worker | -1·54 (-1·82; -1·25) | <0·0001 | -1·18 (-1·42; -0·94) | <0·0001 |  | -0·83 (-1·05; -0·61) | <0·0001 |  |
| Homemaker | 0·41 (0·15; 0·68) | 0·0019 | 0·25 (0·01; 0·48) | 0·037 |  | 0·05 (-0·16; 0·25) | 0·67 |  |
| Retired | -2·25 (-2·4; -2·1) | <0·0001 | -0·5 (-0·72; -0·28) | <0·0001 |  | 0·02 (-0·17; 0·22) | 0·81 |  |
| Student (non-health field) | -1·57 (-1·86; -1·29) | <0·0001 | -0·81 (-1·1; -0·52) | <0·0001 |  | -0·43 (-0·69; -0·16) | 0·0018 |  |
| Student (health field) | -2·59 (-3; -2·18) | <0·0001 | -1·42 (-1·81; -1·03) | <0·0001 |  | -0·73 (-1·08; -0·38) | <0·0001 |  |
| Job seeker | 0·98 (0·63; 1·33) | <0·0001 | 0·1 (-0·21; 0·41) | 0·53 |  | 0·1 (-0·18; 0·39) | 0·48 |  |
| Unemployed | 1·67 (1·32; 2·01) | <0·0001 | 0·22 (-0·08; 0·51) | 0·15 |  | 0·13 (-0·14; 0·4) | 0·35 |  |
| Other | 0·58 (-1·23; 2·38) | 0·53 | 0·17 (-1·38; 1·71) | 0·83 |  | 0·34 (-1; 1·67) | 0·62 |  |
| Continent of citizenship |  |  |  |  | 0·0003 |  |  | 0·0006 |
| Italy | Ref. |  | Ref. |  |  | Ref. |  |  |
| Europe (non-Italy) | 0·46 (-0·15; 1·06) | 0·14 | 0·22 (-0·3; 0·73) | 0·41 |  | 0·42 (-0·04; 0·89) | 0·075 |  |
| Africa | 1·81 (0·49; 3·14) | 0·0073 | 2·09 (0·82; 3·37) | 0·0013 |  | 2·66 (1·3; 4·02) | 0·00012 |  |
| America | 0·11 (-1·06; 1·27) | 0·85 | -0·16 (-1·34; 1·01) | 0·78 |  | 0·33 (-0·78; 1·44) | 0·56 |  |
| Asia | -0·06 (-1·43; 1·31) | 0·93 | 0·96 (-0·46; 2·38) | 0·19 |  | 0·76 (-0·55; 2·06) | 0·26 |  |
| Oceania | 1·75 (1·68; 1·81) | <0·0001 | -9·79 (-11·21; -8·36) | <0·0001 |  | -8·67 (-10·04; -7·3) | <0·0001 |  |
| Self-identified ethnicity |  |  |  |  | 0·0003 |  |  | 0·0003 |
| European | Ref. |  | Ref. |  |  | Ref. |  |  |
| Multi-ethnic | 2·51 (1·81; 3·22) | <0·0001 | 0·4 (-0·25; 1·05) | 0·23 |  | 0·46 (-0·2; 1·12) | 0·17 |  |
| North American / Australian | 3·75 (2·5; 5) | <0·0001 | 1·18 (0·09; 2·26) | 0·033 |  | 1·01 (-0·07; 2·1) | 0·066 |  |
| Arab-Middle Eastern | 2·49 (1·62; 3·37) | <0·0001 | -0·21 (-1·05; 0·63) | 0·62 |  | 0 (-0·86; 0·85) | 0·99 |  |
| North African | 0·38 (-0·44; 1·2) | 0·37 | -0·67 (-1·52; 0·18) | 0·12 |  | -0·78 (-1·6; 0·04) | 0·062 |  |
| Latino-American | 1·27 (0·51; 2·03) | 0·0011 | -0·12 (-0·92; 0·67) | 0·76 |  | -0·1 (-0·88; 0·67) | 0·799 |  |
| African American | 2·98 (0·88; 5·08) | 0·0055 | -0·65 (-2·78; 1·48) | 0·55 |  | -0·37 (-2·5; 1·75) | 0·73 |  |
| Black African | 0·82 (-0·69; 2·32) | 0·29 | -0·84 (-2·26; 0·58) | 0·25 |  | -1·08 (-2·5; 0·33) | 0·13 |  |
| Asian | 0·35 (-0·68; 1·38) | 0·51 | -0·84 (-1·87; 0·18) | 0·11 |  | -0·55 (-1·58; 0·47) | 0·29 |  |
| Pacific Islands | 1·13 (-1·48; 3·74) | 0·396 | -0·89 (-3; 1·22) | 0·41 |  | -0·64 (-3·02; 1·74) | 0·599 |  |
| Material deprivation |  |  |  |  | 0·0000 |  |  | 0·0000 |
| No deprivation | Ref. |  | Ref. |  |  | Ref. |  |  |
| Severe deprivation | 1·84 (1·48; 2·19) | <0·0001 | 0·18 (-0·12; 0·48) | 0·23 |  | -0·18 (-0·43; 0·08) | 0·18 |  |
| Chronic conditions |  |  |  |  | 0·0006 |  |  | 0·0010 |
| No chronic disease | Ref. |  | Ref. |  |  | Ref. |  |  |
| One chronic disease | -0·09 (-0·24; 0·05) | 0·21 | -0·17 (-0·3; -0·04) | 0·00897 |  | -0·16 (-0·27; -0·04) | 0·0074 |  |
| More than one chronic disease | -0·95 (-1·13; -0·78) | <0·0001 | -0·45 (-0·61; -0·28) | <0·0001 |  | -0·52 (-0·67; -0·38) | <0·0001 |  |
| Living with a person with disability |  |  |  |  |  |  |  |  |
| No |  |  |  |  |  |  |  |  |
| Yes | 0·53 (0·37; 0·7) | <0·0001 | -0·25 (-0·4; -0·11) | 0·00074 | 0·0002 | -0·16 (-0·29; -0·03) | 0·015 | 0·0001 |
| Inadequate health literacy |  |  |  |  |  |  |  |  |
| No |  |  |  |  |  |  |  |  |
| Yes | -0·47 (-0·6; -0·35) | <0·0001 | -0·39 (-0·51; -0·28) | <0·0001 | 0·0009 | -0·58 (-0·68; -0·48) | <0·0001 | 0·0025 |
| Knowing someone who had AEFI |  |  |  |  | 0·0766 |  |  | 0·0225 |
| No | Ref. |  | Ref. |  |  | Ref. |  |  |
| Yes | 5·2 (5·06; 5·35) | <0·0001 | 4·11 (3·97; 4·24) | <0·0001 |  | 2 (1·88; 2·13) | <0·0001 |  |
| Knowing someone who had VPD |  |  |  |  | 0·0154 |  |  | 0·0070 |
| No | Ref. |  | Ref. |  |  | Ref. |  |  |
| Yes | -0·71 (-0·86; -0·56) | <0·0001 | -1·95 (-2·09; -1·81) | <0·0001 |  | -1·16 (-1·28; -1·04) | <0·0001 |  |
| Reported barriers to vaccination |  |  |  |  | 0·0005 |  |  | 0·0001 |
| No | Ref. |  | Ref. |  |  | Ref. |  |  |
| Yes | 1·7 (1·57; 1·83) | <0·0001 | 0·31 (0·19; 0·43) | <0·0001 |  | -0·11 (-0·22; -0·01) | 0·032 |  |
| Information source cluster |  |  |  |  | 0·0012 |  |  | 0·0003 |
| Diversified sources | Ref. |  | Ref. |  |  | Ref. |  |  |
| Professional-only sources | -2·47 (-2·6; -2·34) | <0·0001 | -0·48 (-0·59; -0·36) | <0·0001 |  | -0·21 (-0·31; -0·11) | <0·0001 |  |
| Trust in sources | -4·15 (-4·25; -4·05) | <0·0001 | -2·41 (-2·51; -2·31) | <0·0001 | 0·0492 | -1·58 (-1·67; -1·49) | <0·0001 | 0·0270 |
| By religious leaders |  |  |  |  | 0·0004 |  |  | 0·0002 |
| Yes | Ref. |  | Ref. |  |  | Ref. |  |  |
| No | 2·16 (1·97; 2·35) | <0·0001 | 0·17 (-0·02; 0·36) | 0·077 |  | -0·11 (-0·28; 0·07) | 0·24 |  |
| Don’t know | 0·18 (0·03; 0·34) | 0·021 | -0·25 (-0·4; -0·09) | 0·0016 |  | -0·24 (-0·37; -0·11) | 0·00034 |  |
| By political leaders |  |  |  |  | 0·0044 |  |  | 0·0010 |
| Yes | Ref. |  | Ref. |  |  | Ref. |  |  |
| No | 1·98 (1·8; 2·16) | <0·0001 | -0·37 (-0·56; -0·18) | 0·00015 |  | -0·19 (-0·37; -0·01) | 0·039 |  |
| Don’t know | -0·37 (-0·51; -0·23) | <0·0001 | -1·22 (-1·37; -1·06) | <0·0001 |  | -0·52 (-0·65; -0·38) | <0·0001 |  |
| By teachers |  |  |  |  | 0·0040 |  |  | 0·0025 |
| Yes | Ref. |  | Ref. |  |  | Ref. |  |  |
| No | 3·74 (3·55; 3·92) | <0·0001 | 0·91 (0·71; 1·12) | <0·0001 |  | 0·48 (0·29; 0·68) | <0·0001 |  |
| Don’t know | 1·41 (1·27; 1·54) | <0·0001 | 1·07 (0·92; 1·22) | <0·0001 |  | 0·79 (0·66; 0·91) | <0·0001 |  |
| By health professionals |  |  |  |  | 0·0077 |  |  | 0·0020 |
| Yes | Ref. |  | Ref. |  |  | Ref. |  |  |
| No | 4·06 (3·87; 4·26) | <0·0001 | 1·24 (1·03; 1·45) | <0·0001 |  | 0·59 (0·39; 0·79) | <0·0001 |  |
| Don’t know | 2·3 (2·15; 2·45) | <0·0001 | 1·48 (1·32; 1·64) | <0·0001 |  | 0·67 (0·53; 0·81) | <0·0001 |  |
| Use of non-conventional medicine |  |  |  |  | 0·0119 |  |  | 0·0042 |
| No | Ref. |  | Ref. |  |  | Ref. |  |  |
| Yes, integrated with conventional medicine | 1·19 (1·04; 1·33) | <0·0001 | 0·25 (0·11; 0·39) | 0·00036 |  | -0·12 (-0·24; 0) | 0·052 |  |
| Yes, as alternative to conventional medicine | 4·52 (4·28; 4·76) | <0·0001 | 2·41 (2·2; 2·63) | <0·0001 |  | 1·18 (0·99; 1·37) | <0·0001 |  |
| Political orientation |  |  |  |  | 0·0076 |  |  | 0·0027 |
| Right (7–9) | Ref. |  | Ref. |  |  | Ref. |  |  |
| Centre (4–6) | 0·71 (0·54; 0·88) | <0·0001 | -0·07 (-0·22; 0·08) | 0·37 |  | 0·31 (0·18; 0·44) | <0·0001 |  |
| Extreme left (0) | 0·83 (0·44; 1·21) | <0·0001 | -0·69 (-1·02; -0·36) | <0·0001 |  | 0·2 (-0·09; 0·5) | 0·17 |  |
| Left (1–3) | -1·12 (-1·32; -0·92) | <0·0001 | -1·14 (-1·33; -0·96) | <0·0001 |  | -0·06 (-0·22; 0·1) | 0·48 |  |
| Extreme right (10) | 1·59 (1·19; 1·99) | <0·0001 | 0·87 (0·53; 1·21) | <0·0001 |  | 0·06 (-0·25; 0·38) | 0·69 |  |
| Non-aligned with traditional parties | 1·33 (1·11; 1·54) | <0·0001 | 0·65 (0·47; 0·83) | <0·0001 |  | 0·79 (0·63; 0·95) | <0·0001 |  |
| Prefer not to answer | -1·87 (-2·11; -1·63) | <0·0001 | -0·06 (-0·28; 0·16) | 0·62 |  | 0·6 (0·4; 0·79) | <0·0001 |  |
| Religion |  |  |  |  | 0·0034 |  |  | 0·0025 |
| Catholic | Ref. |  | Ref. |  |  | Ref. |  |  |
| Orthodox | -1·24 (-1·57; -0·91) | <0·0001 | -0·62 (-0·93; -0·3) | 0·00013 |  | -0·64 (-0·93; -0·35) | <0·0001 |  |
| Protestant | 3·06 (2·36; 3·77) | <0·0001 | 1·05 (0·43; 1·67) | 0·00089 |  | 0·96 (0·39; 1·53) | 0·001 |  |
| Jewish | 3·72 (2·65; 4·78) | <0·0001 | 1·49 (0·56; 2·42) | 0·0017 |  | 1·78 (0·89; 2·67) | <0·0001 |  |
| Muslim | 1·59 (1·05; 2·13) | <0·0001 | 0·8 (0·24; 1·36) | 0·0051 |  | 0·4 (-0·15; 0·96) | 0·16 |  |
| Jehovah’s Witness | 2·53 (1·88; 3·17) | <0·0001 | 0·38 (-0·19; 0·95) | 0·19 |  | 0·58 (0·05; 1·11) | 0·033 |  |
| Atheist | 0·28 (0·07; 0·5) | 0·011 | -0·12 (-0·36; 0·12) | 0·33 |  | -0·01 (-0·22; 0·2) | 0·96 |  |
| Agnostic | -0·14 (-0·49; 0·21) | 0·43 | -0·18 (-0·5; 0·14) | 0·27 |  | 0·09 (-0·18; 0·36) | 0·501 |  |
| Buddhist | 3·45 (2·49; 4·4) | <0·0001 | 1·34 (0·56; 2·11) | 0·00076 |  | 0·96 (0·28; 1·65) | 0·0057 |  |
| Hindu | 5·48 (3·59; 7·38) | <0·0001 | 2·55 (1·01; 4·09) | 0·0012 |  | 1·43 (-0·07; 2·94) | 0·062 |  |
| Other | 4·19 (3·64; 4·75) | <0·0001 | 1·79 (1·32; 2·25) | <0·0001 |  | 1·31 (0·91; 1·71) | <0·0001 |  |
| Prefer not to answer | 2·15 (1·85; 2·46) | <0·0001 | 0·75 (0·47; 1·03) | <0·0001 |  | 0·49 (0·23; 0·74) | 0·00016 |  |
| Importance of religion |  |  |  |  | 0·0006 |  |  | 0·0022 |
| Not at all (0) | Ref. |  | Ref. |  |  | Ref. |  |  |
| Slightly (1–3) | 0·16 (-0·09; 0·42) | 0·22 | 0·21 (-0·02; 0·45) | 0·076 |  | -0·01 (-0·21; 0·2) | 0·96 |  |
| Somewhat important (4–6) | 0·1 (-0·12; 0·32) | 0·39 | -0·19 (-0·43; 0·04) | 0·11 |  | -0·52 (-0·73; -0·31) | <0·0001 |  |
| Very (7–9) | -1·67 (-1·89; -1·45) | <0·0001 | -0·22 (-0·47; 0·02) | 0·074 |  | -0·75 (-0·96; -0·53) | <0·0001 |  |
| Extremely (10) | -0·85 (-1·16; -0·54) | <0·0001 | -0·1 (-0·41; 0·21) | 0·51 |  | -0·88 (-1·16; -0·61) | <0·0001 |  |
| Prefer not to answer | -0·08 (-0·44; 0·28) | 0·67 | 0·24 (-0·12; 0·6) | 0·19 |  | -0·74 (-1·07; -0·42) | <0·0001 |  |
| Perceived NHS quality | -1·14 (-1·18; -1·11) | <0·0001 | -0·46 (-0·5; -0·42) | <0·0001 | 0·0117 | -0·3 (-0·34; -0·27) | <0·0001 | 0·0067 |
| Perceived NHS access | -1·01 (-1·04; -0·98) | <0·0001 | -0·31 (-0·35; -0·27) | <0·0001 | 0·0057 | -0·31 (-0·35; -0·27) | <0·0001 | 0·0074 |
| Survey mode |  |  |  |  | 0·0021 |  |  | 0·0005 |
| CAWI | Ref. |  | Ref. |  |  | Ref. |  |  |
| CATI | -2·91 (-3·04; -2·77) | <0·0001 | -0·86 (-1·03; -0·69) | <0·0001 |  | 0·36 (0·22; 0·51) | <0·0001 |  |
| Vaccine conspiracy belief scale | 2·53 (2·50; 2·57) | <0·0001 |  |  |  | 1·95 (1·91; 1·99) |  | 0·2195 |

*partial eta-squared

Abbreviations: adjCoef adjusted coefficient, CATI Computer Assisted Telephone Interviewing, CAWI Computer Assisted Web Interviewing, CI Confidence Interval, NHS National Health Service, VPD Vaccine Preventable Disease.

Note: Regression estimates should not be interpreted as causal effects.

All models were estimated using robust variance–covariance estimators to account for potential heteroskedasticity. Effect sizes (partial η²) were derived from the corresponding non-robust models.

## Table S14. Unweighted analysis: Hierarchical linear regression model for “Risk perception” subscale

| **RISK PERCEPTION** | **Univariable regression** | | **Multiple regression** | | | **Multiple regression** | | |
| --- | --- | --- | --- | --- | --- | --- | --- | --- |
|  |  |  | **BLOCK 1 (R-squared=0.1997)** | | | **BLOCK 2 (R-squared=0.3365)** | | |
| **Variable** | **Coef (95%CI)** | **p** | **adjCoef (95% CI)** | **p** | **Effect Size*** | **adjCoef (95% CI)** | **p** | **Effect Size*** |
|  |  |  |  |  |  |  |  |  |
| Age group |  |  |  |  | 0·0024 |  |  | 0·0036 |
| 18–29 | Ref. |  |  |  |  |  |  |  |
| 30–44 | 0·45 (0·35; 0·54) | <0·0001 | 0·31 (0·21; 0·41) | <0·0001 |  | 0·13 (0·05; 0·22) | 0·0029 |  |
| 45–59 | 0·35 (0·26; 0·43) | <0·0001 | 0·48 (0·37; 0·58) | <0·0001 |  | 0·23 (0·13; 0·32) | <0·0001 |  |
| 60–74 | 0·38 (0·28; 0·47) | <0·0001 | 0·69 (0·55; 0·82) | <0·0001 |  | 0·34 (0·22; 0·46) | <0·0001 |  |
| 75+ | 0·99 (0·88; 1·1) | <0·0001 | 0·85 (0·7; 1·01) | <0·0001 |  | 0·87 (0·73; 1·01) | <0·0001 |  |
| Gender |  |  |  |  | 0·0038 |  |  | 0·0055 |
| Male | Ref. |  | Ref. |  |  | Ref. |  |  |
| Female | 0·35 (0·29; 0·41) | <0·0001 | 0·36 (0·3; 0·41) | <0·0001 |  | 0·37 (0·32; 0·42) | <0·0001 |  |
| Non-binary/Other | 0·25 (-0·01; 0·51) | 0·064 | -0·64 (-0·92; -0·36) | <0·0001 |  | -0·93 (-1·19; -0·67) | <0·0001 |  |
| Prefer not to answer | -0·77 (-1·71; 0·17) | 0·11 | -1·19 (-2·1; -0·27) | 0·011 |  | -1·3 (-2·12; -0·48) | 0·0018 |  |
| Marital status |  |  |  |  | 0·0003 |  |  | 0·0003 |
| Single | Ref. |  | Ref. |  |  | Ref. |  |  |
| Married | 0·29 (0·22; 0·36) | <0·0001 | -0·04 (-0·13; 0·05) | 0·41 |  | -0·01 (-0·09; 0·07) | 0·797 |  |
| Separated/Divorced | 0·43 (0·3; 0·55) | <0·0001 | -0·03 (-0·16; 0·1) | 0·66 |  | -0·11 (-0·23; 0·01) | 0·0697 |  |
| Cohabiting | 0·35 (0·25; 0·45) | <0·0001 | 0·13 (0·03; 0·22) | 0·012 |  | 0·09 (0; 0·17) | 0·044 |  |
| Widowed | 0·49 (0·33; 0·65) | <0·0001 | -0·15 (-0·32; 0·02) | 0·085 |  | -0·1 (-0·26; 0·07) | 0·25 |  |
| Children |  |  |  |  | 0·0010 |  |  | 0·0010 |
| No children | Ref. |  | Ref. |  |  | Ref. |  |  |
| Only children ≤11 years | 0·35 (0·26; 0·44) | <0·0001 | 0·08 (-0·02; 0·18) | 0·11 |  | 0·09 (0·01; 0·18) | 0·035 |  |
| Only children 12-18 years | 0·51 (0·39; 0·62) | <0·0001 | 0·14 (0·02; 0·26) | 0·025 |  | 0·11 (0; 0·21) | 0·051 |  |
| Only children >18 years | 0·25 (0·18; 0·31) | <0·0001 | -0·25 (-0·34; -0·15) | <0·0001 |  | -0·22 (-0·31; -0·14) | <0·0001 |  |
| Children of various ages | 0·28 (0·14; 0·41) | <0·0001 | 0·04 (-0·1; 0·17) | 0·58 |  | 0·05 (-0·07; 0·17) | 0·39 |  |
| Sexual orientation |  |  |  |  | 0·0082 |  |  | 0·0109 |
| Heterosexual | Ref. |  | Ref. |  |  | Ref. |  |  |
| Homosexual | -0·47 (-0·7; -0·25) | <0·0001 | -0·28 (-0·48; -0·07) | 0·0097 |  | -0·19 (-0·37; -0·01) | 0·039 |  |
| Bisexual | -0·39 (-0·59; -0·2) | <0·0001 | -0·22 (-0·41; -0·04) | 0·019 |  | -0·03 (-0·19; 0·13) | 0·71 |  |
| Pansexual | -0·2 (-0·5; 0·1) | 0·18 | -0·46 (-0·75; -0·18) | 0·0014 |  | -0·17 (-0·43; 0·1) | 0·21 |  |
| Ace spectrum | 0·44 (0·18; 0·7) | 0·00082 | -0·05 (-0·32; 0·23) | 0·74 |  | 0·26 (0·03; 0·5) | 0·029 |  |
| Prefer not to answer | 1·99 (1·87; 2·11) | <0·0001 | 1·29 (1·15; 1·42) | <0·0001 |  | 1·39 (1·26; 1·52) | <0·0001 |  |
| Municipality size (inhabitants) |  |  |  |  | 0·0001 |  |  | 0·0001 |
| ≤10,000 | Ref. |  | Ref. |  |  | Ref. |  |  |
| 10,001–25,000 | -0·3 (-0·38; -0·22) | <0·0001 | -0·02 (-0·1; 0·06) | 0·63 |  | 0·01 (-0·06; 0·08) | 0·72 |  |
| 25,001–50,000 | -0·33 (-0·43; -0·24) | <0·0001 | 0·01 (-0·09; 0·1) | 0·902 |  | 0·01 (-0·07; 0·1) | 0·74 |  |
| 50,001–100,000 | -0·2 (-0·31; -0·1) | <0·0001 | 0·09 (-0·03; 0·22) | 0·15 |  | 0·07 (-0·05; 0·19) | 0·25 |  |
| 100,001–250,000 | -0·09 (-0·21; 0·02) | 0·12 | 0·14 (-0·01; 0·3) | 0·0703 |  | 0·18 (0·04; 0·33) | 0·011 |  |
| >250,000 | -0·27 (-0·36; -0·18) | <0·0001 | 0·09 (-0·05; 0·23) | 0·201 |  | 0·09 (-0·04; 0·22) | 0·18 |  |
| Geographic macro-area |  |  |  |  | 0·0025 |  |  | 0·0034 |
| North-West | Ref. |  | Ref. |  |  | Ref. |  |  |
| North-East | -0·09 (-0·17; 0) | 0·046 | -0·07 (-0·15; 0·01) | 0·075 |  | -0·1 (-0·17; -0·03) | 0·0067 |  |
| Centre | 0·11 (0·03; 0·2) | 0·01 | 0·24 (0·16; 0·32) | <0·0001 |  | 0·26 (0·19; 0·34) | <0·0001 |  |
| South | 0·13 (0·04; 0·21) | 0·003 | 0·06 (-0·02; 0·14) | 0·16 |  | 0·07 (0; 0·15) | 0·053 |  |
| Islands | -0·31 (-0·42; -0·21) | <0·0001 | -0·32 (-0·42; -0·22) | <0·0001 |  | -0·3 (-0·39; -0·21) | <0·0001 |  |
| Degree of urbanisation |  |  |  |  | 0·0002 |  |  | 0·0002 |
| Pole | Ref. |  | Ref. |  |  | Ref. |  |  |
| Intermunicipal pole | 0·18 (-0·01; 0·37) | 0·065 | 0·19 (0·01; 0·38) | 0·0399 |  | 0·23 (0·06; 0·4) | 0·0078 |  |
| Belt | 0·09 (0·02; 0·15) | 0·011 | 0·03 (-0·08; 0·15) | 0·55 |  | 0·04 (-0·06; 0·14) | 0·43 |  |
| Intermediate | 0·04 (-0·05; 0·13) | 0·42 | -0·07 (-0·2; 0·06) | 0·299 |  | -0·01 (-0·13; 0·11) | 0·895 |  |
| Peripheral | 0·16 (0·04; 0·27) | 0·0088 | 0·01 (-0·14; 0·16) | 0·88 |  | 0·06 (-0·08; 0·19) | 0·401 |  |
| Ultra-peripheral | 0·34 (0·09; 0·6) | 0·0083 | -0·03 (-0·29; 0·22) | 0·79 |  | -0·01 (-0·24; 0·22) | 0·93 |  |
| Education level |  |  |  |  | 0·0046 |  |  | 0·0029 |
| Upper secondary | Ref. |  | Ref. |  |  | Ref. |  |  |
| Primary/None | 0·13 (-0·08; 0·35) | 0·23 | -0·55 (-0·76; -0·35) | <0·0001 |  | -0·46 (-0·66; -0·26) | <0·0001 |  |
| Lower secondary | 0·66 (0·58; 0·75) | <0·0001 | 0·22 (0·14; 0·31) | <0·0001 |  | 0·14 (0·06; 0·22) | 0·00058 |  |
| University | -0·58 (-0·65; -0·51) | <0·0001 | -0·36 (-0·42; -0·29) | <0·0001 |  | -0·26 (-0·32; -0·2) | <0·0001 |  |
| Postgraduate | -0·46 (-0·59; -0·34) | <0·0001 | -0·4 (-0·51; -0·28) | <0·0001 |  | -0·28 (-0·38; -0·18) | <0·0001 |  |
| Occupational status |  |  |  |  | 0·0024 |  |  | 0·0021 |
| Non-healthcare worker | Ref. |  | Ref. |  |  | Ref. |  |  |
| Healthcare worker | -0·23 (-0·37; -0·1) | 0·00091 | -0·3 (-0·43; -0·18) | <0·0001 |  | -0·15 (-0·26; -0·03) | 0·012 |  |
| Homemaker | 0·29 (0·18; 0·4) | <0·0001 | -0·08 (-0·19; 0·03) | 0·14 |  | -0·17 (-0·27; -0·07) | 0·0005 |  |
| Retired | 0·41 (0·34; 0·48) | <0·0001 | 0·01 (-0·09; 0·12) | 0·83 |  | 0·24 (0·15; 0·34) | <0·0001 |  |
| Student (non-health field) | -0·8 (-0·94; -0·67) | <0·0001 | -0·62 (-0·77; -0·47) | <0·0001 |  | -0·45 (-0·58; -0·32) | <0·0001 |  |
| Student (health field) | -0·97 (-1·2; -0·75) | <0·0001 | -0·88 (-1·09; -0·67) | <0·0001 |  | -0·57 (-0·76; -0·38) | <0·0001 |  |
| Job seeker | 0·26 (0·11; 0·41) | 0·00057 | -0·04 (-0·18; 0·11) | 0·61 |  | -0·04 (-0·17; 0·09) | 0·57 |  |
| Unemployed | 0·39 (0·26; 0·53) | <0·0001 | 0 (-0·13; 0·14) | 0·96 |  | -0·03 (-0·15; 0·08) | 0·57 |  |
| Other | 0·23 (-0·41; 0·88) | 0·48 | -0·04 (-0·64; 0·57) | 0·91 |  | 0·04 (-0·45; 0·53) | 0·87 |  |
| Continent of citizenship |  |  |  |  | 0·0003 |  |  | 0·0002 |
| Italy | Ref. |  | Ref. |  |  | Ref. |  |  |
| Europe (non-Italy) | 0·09 (-0·18; 0·36) | 0·53 | -0·42 (-0·69; -0·16) | 0·0018 |  | -0·33 (-0·57; -0·09) | 0·0065 |  |
| Africa | -0·65 (-1·13; -0·17) | 0·0084 | -0·48 (-1·11; 0·14) | 0·13 |  | -0·23 (-0·79; 0·32) | 0·41 |  |
| America | -0·19 (-0·72; 0·33) | 0·47 | -0·24 (-0·9; 0·43) | 0·48 |  | -0·02 (-0·63; 0·59) | 0·95 |  |
| Asia | 0·08 (-0·46; 0·63) | 0·76 | 0·55 (-0·1; 1·19) | 0·099 |  | 0·46 (-0·15; 1·06) | 0·14 |  |
| Oceania | 0·03 (0; 0·06) | 0·063 | -1·81 (-2·48; -1·15) | <0·0001 |  | -1·32 (-1·94; -0·71) | <0·0001 |  |
| Self-identified ethnicity |  |  |  |  | 0·0006 |  |  | 0·0006 |
| European | Ref. |  | Ref. |  |  | Ref. |  |  |
| Multi-ethnic | 0·04 (-0·27; 0·34) | 0·81 | -0·41 (-0·71; -0·11) | 0·0071 |  | -0·39 (-0·64; -0·13) | 0·0033 |  |
| North American / Australian | 0·35 (-0·16; 0·87) | 0·18 | -0·47 (-0·99; 0·06) | 0·081 |  | -0·54 (-1·02; -0·06) | 0·029 |  |
| Arab-Middle Eastern | 0·08 (-0·32; 0·48) | 0·695 | -0·5 (-0·93; -0·08) | 0·0202 |  | -0·41 (-0·78; -0·04) | 0·0297 |  |
| North African | -0·01 (-0·39; 0·37) | 0·95 | -0·22 (-0·64; 0·2) | 0·31 |  | -0·27 (-0·63; 0·1) | 0·15 |  |
| Latino-American | 0·05 (-0·28; 0·38) | 0·78 | -0·38 (-0·78; 0·03) | 0·067 |  | -0·37 (-0·74; 0·01) | 0·054 |  |
| African American | 0·48 (-0·42; 1·37) | 0·295 | -0·54 (-1·43; 0·35) | 0·23 |  | -0·42 (-1·23; 0·39) | 0·31 |  |
| Black African | -0·43 (-1·05; 0·19) | 0·17 | -0·33 (-1·03; 0·37) | 0·36 |  | -0·44 (-1·09; 0·21) | 0·19 |  |
| Asian | -0·08 (-0·55; 0·38) | 0·72 | -0·72 (-1·28; -0·17) | 0·011 |  | -0·59 (-1·08; -0·1) | 0·018 |  |
| Pacific Islands | -0·12 (-1·2; 0·97) | 0·84 | -1·37 (-2·54; -0·21) | 0·021 |  | -1·26 (-2·27; -0·26) | 0·013 |  |
| Material deprivation |  |  |  |  | 0·0000 |  |  | 0·0001 |
| No deprivation | Ref. |  | Ref. |  |  | Ref. |  |  |
| Severe deprivation | 0·18 (0·03; 0·32) | 0·016 | 0·06 (-0·08; 0·2) | 0·42 |  | -0·1 (-0·22; 0·02) | 0·095 |  |
| Chronic conditions |  |  |  |  | 0·0043 |  |  | 0·0046 |
| No chronic disease | Ref. |  | Ref. |  |  | Ref. |  |  |
| One chronic disease | 0·18 (0·12; 0·25) | <0·0001 | -0·07 (-0·14; -0·01) | 0·022 |  | -0·07 (-0·12; -0·01) | 0·02002 |  |
| More than one chronic disease | 1·13 (1·05; 1·22) | <0·0001 | 0·53 (0·45; 0·61) | <0·0001 |  | 0·49 (0·42; 0·57) | <0·0001 |  |
| Living with a person with disability |  |  |  |  |  |  |  |  |
| No |  |  |  |  |  |  |  |  |
| Yes | 0·56 (0·49; 0·63) | <0·0001 | 0·11 (0·04; 0·18) | 0·0033 | 0·0002 | 0·15 (0·08; 0·21) | <0·0001 | 0·0004 |
| Inadequate health literacy |  |  |  |  |  |  |  |  |
| No |  |  |  |  |  |  |  |  |
| Yes | 0·84 (0·78; 0·9) | <0·0001 | 0·56 (0·51; 0·62) | <0·0001 | 0·0074 | 0·48 (0·43; 0·53) | <0·0001 | 0·0065 |
| Knowing someone who had AEFI |  |  |  |  | 0·0410 |  |  | 0·0064 |
| No | Ref. |  | Ref. |  |  | Ref. |  |  |
| Yes | 1·88 (1·82; 1·94) | <0·0001 | 1·47 (1·41; 1·54) | <0·0001 |  | 0·55 (0·49; 0·6) | <0·0001 |  |
| Knowing someone who had VPD |  |  |  |  | 0·0018 |  |  | 0·0000 |
| No | Ref. |  | Ref. |  |  | Ref. |  |  |
| Yes | 0·36 (0·29; 0·44) | <0·0001 | -0·33 (-0·4; -0·26) | <0·0001 |  | 0·02 (-0·05; 0·08) | 0·604 |  |
| Reported barriers to vaccination |  |  |  |  | 0·0049 |  |  | 0·0022 |
| No | Ref. |  | Ref. |  |  | Ref. |  |  |
| Yes | 1·12 (1·06; 1·18) | <0·0001 | 0·47 (0·42; 0·53) | <0·0001 |  | 0·29 (0·23; 0·34) | <0·0001 |  |
| Information source cluster |  |  |  |  | 0·0073 |  |  | 0·0056 |
| Diversified sources | Ref. |  | Ref. |  |  | Ref. |  |  |
| Professional-only sources | -1·08 (-1·14; -1·02) | <0·0001 | -0·58 (-0·64; -0·52) | <0·0001 |  | -0·46 (-0·52; -0·41) | <0·0001 |  |
| Trust in sources | -1·03 (-1·07; -0·98) | <0·0001 | -0·46 (-0·51; -0·41) | <0·0001 | 0·0076 | -0·1 (-0·14; -0·05) | <0·0001 | 0·0004 |
| By religious leaders |  |  |  |  | 0·0013 |  |  | 0·0011 |
| Yes | Ref. |  | Ref. |  |  | Ref. |  |  |
| No | 0·62 (0·53; 0·7) | <0·0001 | 0·35 (0·25; 0·44) | <0·0001 |  | 0·22 (0·14; 0·31) | <0·0001 |  |
| Don’t know | -0·01 (-0·09; 0·06) | 0·69 | 0·25 (0·17; 0·33) | <0·0001 |  | 0·25 (0·18; 0·32) | <0·0001 |  |
| By political leaders |  |  |  |  | 0·0022 |  |  | 0·0003 |
| Yes | Ref. |  | Ref. |  |  | Ref. |  |  |
| No | 0·38 (0·3; 0·46) | <0·0001 | -0·21 (-0·31; -0·12) | <0·0001 |  | -0·13 (-0·22; -0·05) | 0·00201 |  |
| Don’t know | -0·25 (-0·31; -0·18) | <0·0001 | -0·43 (-0·51; -0·36) | <0·0001 |  | -0·12 (-0·19; -0·05) | 0·0005 |  |
| By teachers |  |  |  |  | 0·0011 |  |  | 0·0026 |
| Yes | Ref. |  | Ref. |  |  | Ref. |  |  |
| No | 0·76 (0·68; 0·84) | <0·0001 | -0·01 (-0·11; 0·09) | 0·83 |  | -0·2 (-0·29; -0·11) | <0·0001 |  |
| Don’t know | 0·02 (-0·04; 0·08) | 0·51 | -0·29 (-0·37; -0·22) | <0·0001 |  | -0·42 (-0·49; -0·35) | <0·0001 |  |
| By health professionals |  |  |  |  | 0·0050 |  |  | 0·0012 |
| Yes | Ref. |  | Ref. |  |  | Ref. |  |  |
| No | 0·99 (0·91; 1·08) | <0·0001 | 0·28 (0·18; 0·38) | <0·0001 |  | -0·01 (-0·1; 0·08) | 0·88 |  |
| Don’t know | 0·72 (0·65; 0·78) | <0·0001 | 0·65 (0·57; 0·73) | <0·0001 |  | 0·29 (0·22; 0·36) | <0·0001 |  |
| Use of non-conventional medicine |  |  |  |  | 0·0037 |  |  | 0·0010 |
| No | Ref. |  | Ref. |  |  | Ref. |  |  |
| Yes, integrated with conventional medicine | 0·32 (0·25; 0·39) | <0·0001 | -0·03 (-0·1; 0·04) | 0·35 |  | -0·2 (-0·25; -0·14) | <0·0001 |  |
| Yes, as alternative to conventional medicine | 1·43 (1·34; 1·52) | <0·0001 | 0·65 (0·55; 0·74) | <0·0001 |  | 0·1 (0·02; 0·19) | 0·021 |  |
| Political orientation |  |  |  |  | 0·0168 |  |  | 0·0080 |
| Right (7–9) | Ref. |  | Ref. |  |  | Ref. |  |  |
| Centre (4–6) | -0·49 (-0·57; -0·41) | <0·0001 | -0·37 (-0·44; -0·29) | <0·0001 |  | -0·2 (-0·27; -0·14) | <0·0001 |  |
| Extreme left (0) | -1·16 (-1·33; -1) | <0·0001 | -0·76 (-0·92; -0·6) | <0·0001 |  | -0·36 (-0·5; -0·22) | <0·0001 |  |
| Left (1–3) | -1·46 (-1·56; -1·36) | <0·0001 | -0·88 (-0·97; -0·79) | <0·0001 |  | -0·4 (-0·48; -0·32) | <0·0001 |  |
| Extreme right (10) | 0·5 (0·34; 0·67) | <0·0001 | 0·52 (0·36; 0·69) | <0·0001 |  | 0·17 (0·02; 0·31) | 0·022 |  |
| Non-aligned with traditional parties | 0·18 (0·08; 0·27) | 0·00025 | 0·34 (0·25; 0·43) | <0·0001 |  | 0·4 (0·32; 0·49) | <0·0001 |  |
| Prefer not to answer | -1·02 (-1·14; -0·89) | <0·0001 | -0·34 (-0·46; -0·22) | <0·0001 |  | -0·05 (-0·16; 0·06) | 0·35 |  |
| Religion |  |  |  |  | 0·0018 |  |  | 0·0018 |
| Catholic | Ref. |  | Ref. |  |  | Ref. |  |  |
| Orthodox | 1·59 (1·43; 1·76) | <0·0001 | 0·64 (0·48; 0·81) | <0·0001 |  | 0·63 (0·48; 0·79) | <0·0001 |  |
| Protestant | 0·12 (-0·17; 0·4) | 0·42 | -0·18 (-0·46; 0·11) | 0·22 |  | -0·22 (-0·47; 0·03) | 0·091 |  |
| Jewish | -0·28 (-0·72; 0·17) | 0·23 | -0·63 (-1·09; -0·17) | 0·0077 |  | -0·5 (-0·91; -0·09) | 0·016 |  |
| Muslim | 0·21 (-0·03; 0·44) | 0·082 | 0·24 (-0·02; 0·51) | 0·073 |  | 0·07 (-0·16; 0·3) | 0·56 |  |
| Jehovah’s Witness | 0·68 (0·43; 0·93) | <0·0001 | 0·07 (-0·19; 0·32) | 0·61 |  | 0·15 (-0·09; 0·39) | 0·21 |  |
| Atheist | -0·83 (-0·93; -0·74) | <0·0001 | -0·16 (-0·28; -0·05) | 0·0041 |  | -0·11 (-0·21; -0·02) | 0·023 |  |
| Agnostic | -0·91 (-1·06; -0·76) | <0·0001 | -0·12 (-0·27; 0·03) | 0·11 |  | 0 (-0·13; 0·13) | 0·99 |  |
| Buddhist | 0·22 (-0·14; 0·59) | 0·23 | -0·02 (-0·37; 0·33) | 0·91 |  | -0·18 (-0·5; 0·13) | 0·25 |  |
| Hindu | 1·09 (0·36; 1·82) | 0·0035 | 0·34 (-0·36; 1·03) | 0·34 |  | -0·15 (-0·77; 0·47) | 0·63 |  |
| Other | 0·56 (0·34; 0·77) | <0·0001 | 0·32 (0·12; 0·53) | 0·00198 |  | 0·11 (-0·06; 0·28) | 0·19 |  |
| Prefer not to answer | 0·17 (0·04; 0·29) | 0·0087 | -0·07 (-0·2; 0·07) | 0·33 |  | -0·18 (-0·31; -0·06) | 0·00396 |  |
| Importance of religion |  |  |  |  | 0·0054 |  |  | 0·0036 |
| Not at all (0) | Ref. |  | Ref. |  |  | Ref. |  |  |
| Slightly (1–3) | 0·36 (0·26; 0·47) | <0·0001 | 0·23 (0·12; 0·34) | <0·0001 |  | 0·13 (0·04; 0·23) | 0·0069 |  |
| Somewhat important (4–6) | 1·09 (1; 1·19) | <0·0001 | 0·54 (0·42; 0·65) | <0·0001 |  | 0·39 (0·29; 0·49) | <0·0001 |  |
| Very (7–9) | 0·88 (0·78; 0·97) | <0·0001 | 0·47 (0·35; 0·58) | <0·0001 |  | 0·24 (0·13; 0·34) | <0·0001 |  |
| Extremely (10) | 1·07 (0·94; 1·2) | <0·0001 | 0·5 (0·35; 0·64) | <0·0001 |  | 0·15 (0·02; 0·28) | 0·021 |  |
| Prefer not to answer | 1·62 (1·47; 1·78) | <0·0001 | 1·29 (1·12; 1·47) | <0·0001 |  | 0·86 (0·69; 1·03) | <0·0001 |  |
| Perceived NHS quality | -0·14 (-0·15; -0·12) | <0·0001 | -0·04 (-0·06; -0·02) | <0·0001 | 0·0004 | 0·03 (0·01; 0·04) | 0·0033 | 0·0002 |
| Perceived NHS access | -0·1 (-0·11; -0·08) | <0·0001 | 0·03 (0·01; 0·05) | 0·0017 | 0·0002 | 0·03 (0·01; 0·04) | 0·0011 | 0·0002 |
| Survey mode |  |  |  |  | 0·0004 |  |  | 0·0017 |
| CAWI | Ref. |  | Ref. |  |  | Ref. |  |  |
| CATI | 0·09 (0·02; 0·16) | 0·0104 | -0·18 (-0·27; -0·1) | <0·0001 |  | 0·36 (0·28; 0·43) | <0·0001 |  |
| Vaccine Conspiracy Belief Scale | 0·91 (0·90; 0·93) | <0·0001 | 0 (0; 9·16) |  |  | 0·86 (0·84; 0·88) | <0·0001 | 0·1709 |

*partial eta-squared

Abbreviations: adjCoef adjusted coefficient, CATI Computer Assisted Telephone Interviewing, CAWI Computer Assisted Web Interviewing, CI Confidence Interval, NHS National Health Service, VPD Vaccine Preventable Disease.

Note: Regression estimates should not be interpreted as causal effects.

All models were estimated using robust variance–covariance estimators to account for potential heteroskedasticity. Effect sizes (partial η²) were derived from the corresponding non-robust models.

## Table S15. Comparison of unweighted and post-stratification weighted estimates in the multivariable models for the secondary outcomes (“Lack of trust” and “Risk perception” subscales)

|  | **LACK OF TRUST Multiple regression BLOCK 1** | | | | | **LACK OF TRUST Multiple regression BLOCK 2** | | | | | **RISK PERCEPTION Multiple regression BLOCK 1** | | | | | **RISK PERCEPTION Multiple regression BLOCK 2** | | | | |
| --- | --- | --- | --- | --- | --- | --- | --- | --- | --- | --- | --- | --- | --- | --- | --- | --- | --- | --- | --- | --- |
|  | **Unweighted** |  | **Weighted** |  |  | **Unweighted** |  | **Weighted** |  |  | **Unweighted** |  | **Weighted** |  |  | **Unweighted** |  | **Weighted** |  |  |
|  | **adjCoef** | **SE** | **adjCoef** | **SE** | **Percentage difference adjCoef** | **adjCoef** | **SE** | **adjCoef** | **SE** | **Percentage difference adjCoef** | **adjCoef** | **SE** | **adjCoef** | **SE** | **Percentage difference adjCoef** | **adjCoef** | **SE** | **adjCoef** | **SE** | **Percentage difference adjCoef** |
| Age group |  |  |  |  |  |  |  |  |  |  |  |  |  |  |  |  |  |  |  |  |
| 18–29 | Ref. |  | Ref. |  |  | Ref. |  | Ref. |  |  | Ref. |  | Ref. |  |  | Ref. |  | Ref. |  |  |
| 30–44 | 1·239*** | -0·105 | 1·228*** | -0·107 | -0·89 | 0·834*** | -0·0982 | 0·815*** | -0·0997 | -2·28 | 0·314*** | -0·0514 | 0·295*** | -0·0524 | -6·05 | 0·135** | -0·0453 | 0·112* | -0·0462 | -17·04 |
| 45–59 | 1·502*** | -0·113 | 1·501*** | -0·114 | -0·07 | 0·934*** | -0·105 | 0·920*** | -0·106 | -1·50 | 0·476*** | -0·0546 | 0·444*** | -0·0555 | -6·72 | 0·225*** | -0·0482 | 0·187*** | -0·0491 | -16·89 |
| 60–74 | 1·765*** | -0·144 | 1·743*** | -0·147 | -1·25 | 0·980*** | -0·13 | 0·951*** | -0·132 | -2·96 | 0·687*** | -0·0698 | 0·610*** | -0·0714 | -11·21 | 0·340*** | -0·0617 | 0·260*** | -0·0633 | -23·53 |
| 75+ | 1·032*** | -0·164 | 1·057*** | -0·174 | 2·42 | 1·070*** | -0·148 | 0·941*** | -0·155 | -12·06 | 0·853*** | -0·0806 | 0·821*** | -0·0851 | -3·75 | 0·870*** | -0·0736 | 0·770*** | -0·0776 | -11·49 |
| Gender |  |  |  |  |  |  |  |  |  |  |  |  |  |  |  |  |  |  |  |  |
| Male | Ref. |  | Ref. |  |  | Ref. |  | Ref. |  |  | Ref. |  | Ref. |  |  | Ref. |  | Ref. |  |  |
| Female | -0·148** | -0·0573 | -0·108 | -0·0596 | -27·03 | -0·120* | -0·0506 | -0·0805 | -0·0526 | -32·92 | 0·358*** | -0·0288 | 0·337*** | -0·03 | -5·87 | 0·371*** | -0·0264 | 0·349*** | -0·0274 | -5·93 |
| Non-binary/Other | 1·766*** | -0·3 | 1·727*** | -0·305 | -2·21 | 1·107*** | -0·277 | 1·159*** | -0·285 | 4·70 | -0·638*** | -0·142 | -0·527*** | -0·147 | -17·40 | -0·929*** | -0·134 | -0·778*** | -0·14 | -16·25 |
| Prefer not to answer | 1·395 | -0·991 | 1·294 | -0·888 | -7·24 | 1·131 | -0·896 | 1·159 | -0·865 | 2·48 | -1·185* | -0·467 | -1·341** | -0·48 | 13·16 | -1·301** | -0·418 | -1·401** | -0·444 | 7·69 |
| Marital status |  |  |  |  |  |  |  |  |  |  |  |  |  |  |  |  |  |  |  |  |
| Single | Ref. |  | Ref. |  |  | Ref. |  | Ref. |  |  | Ref. |  | Ref. |  |  | Ref. |  | Ref. |  |  |
| Married | -0·359*** | -0·097 | -0·319** | -0·0997 | -11·14 | -0·296*** | -0·0876 | -0·292** | -0·0896 | -1·35 | -0·0381 | -0·0459 | -0·0251 | -0·0472 | -34·12 | -0·0104 | -0·0402 | -0·0134 | -0·0415 | 28·85 |
| Separated/Divorced | 0·551*** | -0·146 | 0·553*** | -0·149 | 0·36 | 0·373** | -0·127 | 0·352** | -0·131 | -5·63 | -0·0301 | -0·0675 | 0·00595 | -0·0693 | -119·77 | -0·109 | -0·06 | -0·083 | -0·0622 | -23·85 |
| Cohabiting | 0·0723 | -0·106 | 0·104 | -0·11 | 43·85 | -0·0135 | -0·0933 | -0·0233 | -0·0964 | 72·59 | 0·125* | -0·0501 | 0·147** | -0·0516 | 17·60 | 0·0873* | -0·0434 | 0·0908* | -0·0446 | 4·01 |
| Widowed | -0·575*** | -0·162 | -0·421* | -0·174 | -26·78 | -0·455** | -0·139 | -0·370* | -0·149 | -18·68 | -0·149 | -0·0865 | -0·0936 | -0·0918 | -37·18 | -0·0958 | -0·084 | -0·071 | -0·0896 | -25·89 |
| Children |  |  |  |  |  |  |  |  |  |  |  |  |  |  |  |  |  |  |  |  |
| No children | Ref. |  | Ref. |  |  | Ref. |  | Ref. |  |  | Ref. |  | Ref. |  |  | Ref. |  | Ref. |  |  |
| Only children ≤11 years | -0·380*** | -0·104 | -0·344** | -0·106 | -9·47 | -0·350*** | -0·0952 | -0·282** | -0·0968 | -19·43 | 0·0796 | -0·0501 | 0·0722 | -0·0511 | -9·30 | 0·0925* | -0·0439 | 0·0998* | -0·0448 | 7·89 |
| Only children 12-18 years | -0·577*** | -0·13 | -0·549*** | -0·133 | -4·85 | -0·651*** | -0·118 | -0·589*** | -0·121 | -9·52 | 0·139* | -0·0623 | 0·112 | -0·0636 | -19·42 | 0·107 | -0·0548 | 0·0949 | -0·0561 | -11·31 |
| Only children >18 years | -0·676*** | -0·103 | -0·633*** | -0·107 | -6·36 | -0·625*** | -0·0903 | -0·584*** | -0·0937 | -6·56 | -0·247*** | -0·0497 | -0·252*** | -0·0516 | 2·02 | -0·225*** | -0·0445 | -0·231*** | -0·0465 | 2·67 |
| Children of various ages | -0·761*** | -0·14 | -0·748*** | -0·142 | -1·71 | -0·732*** | -0·124 | -0·690*** | -0·125 | -5·74 | 0·0383 | -0·0693 | 0·0289 | -0·0701 | -24·54 | 0·0509 | -0·0592 | 0·0544 | -0·0601 | 6·88 |
| Sexual orientation |  |  |  |  |  |  |  |  |  |  |  |  |  |  |  |  |  |  |  |  |
| Heterosexual | Ref. |  | Ref. |  |  | Ref. |  | Ref. |  |  | Ref. |  | Ref. |  |  | Ref. |  | Ref. |  |  |
| Homosexual | -0·516* | -0·218 | -0·398 | -0·233 | -22·87 | -0·323 | -0·195 | -0·296 | -0·199 | -8·36 | -0·275** | -0·106 | -0·266* | -0·11 | -3·27 | -0·190* | -0·0923 | -0·221* | -0·0938 | 16·32 |
| Bisexual | -0·758*** | -0·193 | -0·722*** | -0·197 | -4·75 | -0·325 | -0·173 | -0·317 | -0·175 | -2·46 | -0·222* | -0·0946 | -0·207* | -0·0963 | -6·76 | -0·0307 | -0·0824 | -0·0277 | -0·0827 | -9·77 |
| Pansexual | -0·719* | -0·306 | -0·660* | -0·306 | -8·21 | -0·0549 | -0·299 | 0·0262 | -0·301 | -147·72 | -0·463** | -0·145 | -0·465** | -0·15 | 0·43 | -0·169 | -0·135 | -0·162 | -0·142 | -4·14 |
| Ace spectrum | -0·905** | -0·296 | -0·808** | -0·306 | -10·72 | -0·209 | -0·303 | -0·159 | -0·311 | -23·92 | -0·0456 | -0·139 | -0·109 | -0·142 | 139·04 | 0·261* | -0·119 | 0·179 | -0·126 | -31·42 |
| Prefer not to answer | -1·717*** | -0·138 | -1·544*** | -0·145 | -10·08 | -1·482*** | -0·129 | -1·279*** | -0·137 | -13·70 | 1·285*** | -0·0685 | 1·155*** | -0·0706 | -10·12 | 1·389*** | -0·0661 | 1·272*** | -0·0682 | -8·42 |
| Municipality size (inhabitants) |  |  |  |  |  |  |  |  |  |  |  |  |  |  |  |  |  |  |  |  |
| ≤10,000 | Ref. |  | Ref. |  |  | Ref. |  | Ref. |  |  | Ref. |  | Ref. |  |  | Ref. |  | Ref. |  |  |
| 10,001–25,000 | -0·0554 | -0·0793 | -0·0762 | -0·0809 | 37·55 | 0·0159 | -0·0691 | -0·0197 | -0·071 | -223·90 | -0·0187 | -0·0393 | -0·00569 | -0·0391 | -69·57 | 0·0127 | -0·0361 | 0·0194 | -0·0352 | 52·76 |
| 25,001–50,000 | -0·127 | -0·0953 | -0·122 | -0·0968 | -3·94 | -0·108 | -0·0841 | -0·0955 | -0·0855 | -11·57 | 0·00589 | -0·0478 | 0·00997 | -0·0483 | 69·27 | 0·0144 | -0·044 | 0·0216 | -0·0443 | 50·00 |
| 50,001–100,000 | 0·126 | -0·132 | 0·112 | -0·135 | -11·11 | 0·0661 | -0·119 | 0·0288 | -0·12 | -56·43 | 0·0947 | -0·0661 | 0·114 | -0·068 | 20·38 | 0·0683 | -0·0599 | 0·0772 | -0·0624 | 13·03 |
| 100,001–250,000 | 0·0515 | -0·158 | 0·0976 | -0·162 | 89·51 | 0·139 | -0·142 | 0·146 | -0·145 | 5·04 | 0·145 | -0·08 | 0·139 | -0·0828 | -4·14 | 0·184* | -0·0725 | 0·160* | -0·0762 | -13·04 |
| >250,000 | -0·102 | -0·144 | -0·107 | -0·148 | 4·90 | -0·112 | -0·129 | -0·177 | -0·132 | 58·04 | 0·0922 | -0·0721 | 0·0859 | -0·0753 | -6·83 | 0·0875 | -0·0652 | 0·0545 | -0·0693 | -37·71 |
| Geographic macro-area |  |  |  |  |  |  |  |  |  |  |  |  |  |  |  |  |  |  |  |  |
| North-West | Ref. |  | Ref. |  |  | Ref. |  | Ref. |  |  | Ref. |  | Ref. |  |  | Ref. |  | Ref. |  |  |
| North-East | 0·235** | -0·0816 | 0·275*** | -0·0837 | 17·02 | 0·171* | -0·0718 | 0·220** | -0·0735 | 28·65 | -0·0729 | -0·0409 | -0·071 | -0·0419 | -2·61 | -0·101** | -0·0372 | -0·0954* | -0·038 | -5·54 |
| Centre | -0·104 | -0·0817 | -0·0548 | -0·0857 | -47·31 | -0·0414 | -0·0715 | -0·0135 | -0·0749 | -67·39 | 0·237*** | -0·0409 | 0·208*** | -0·0423 | -12·24 | 0·264*** | -0·0376 | 0·227*** | -0·0386 | -14·02 |
| South | -0·482*** | -0·0818 | -0·434*** | -0·0842 | -9·96 | -0·448*** | -0·0726 | -0·443*** | -0·0747 | -1·12 | 0·058 | -0·0409 | 0·0880* | -0·042 | 51·72 | 0·0728 | -0·0376 | 0·0843* | -0·0388 | 15·80 |
| Islands | -0·562*** | -0·104 | -0·569*** | -0·11 | 1·25 | -0·519*** | -0·0915 | -0·565*** | -0·0976 | 8·86 | -0·321*** | -0·0519 | -0·290*** | -0·0554 | -9·66 | -0·302*** | -0·0463 | -0·288*** | -0·0501 | -4·64 |
| Degree of urbanisation |  |  |  |  |  |  |  |  |  |  |  |  |  |  |  |  |  |  |  |  |
| Pole | Ref. |  | Ref. |  |  | Ref. |  | Ref. |  |  | Ref. |  | Ref. |  |  | Ref. |  | Ref. |  |  |
| Intermunicipal pole | -0·224 | -0·188 | -0·194 | -0·191 | -13·39 | -0·144 | -0·17 | -0·15 | -0·173 | 4·17 | 0·193* | -0·094 | 0·118 | -0·0958 | -38·86 | 0·229** | -0·0859 | 0·137 | -0·0877 | -40·17 |
| Belt | 0·219 | -0·113 | 0·216 | -0·115 | -1·37 | 0·234* | -0·102 | 0·236* | -0·103 | 0·85 | 0·0345 | -0·0576 | 0·00143 | -0·0591 | -95·86 | 0·0411 | -0·0523 | 0·0103 | -0·0544 | -74·94 |
| Intermediate | 0·14 | -0·131 | 0·164 | -0·134 | 17·14 | 0·277* | -0·117 | 0·294* | -0·12 | 6·14 | -0·0685 | -0·066 | -0·084 | -0·0677 | 22·63 | -0·00796 | -0·0601 | -0·0264 | -0·0622 | 231·66 |
| Peripheral | 0·137 | -0·148 | 0·158 | -0·153 | 15·33 | 0·243 | -0·131 | 0·259 | -0·135 | 6·58 | 0·0115 | -0·076 | -0·00604 | -0·0777 | -152·52 | 0·0584 | -0·0695 | 0·0388 | -0·0713 | -33·56 |
| Ultra-peripheral | 0·136 | -0·251 | 0·0689 | -0·261 | -49·34 | 0·191 | -0·211 | 0·14 | -0·221 | -26·70 | -0·035 | -0·13 | -0·0189 | -0·135 | -46·00 | -0·0109 | -0·119 | 0·0125 | -0·123 | -214·68 |
| Education level |  |  |  |  |  |  |  |  |  |  |  |  |  |  |  |  |  |  |  |  |
| Upper secondary | Ref. |  | Ref. |  |  | Ref. |  | Ref. |  |  | Ref. |  | Ref. |  |  | Ref. |  | Ref. |  |  |
| Primary/None | -0·740*** | -0·179 | -0·944*** | -0·194 | 27·57 | -0·535** | -0·166 | -0·690*** | -0·186 | 28·97 | -0·554*** | -0·105 | -0·506*** | -0·114 | -8·66 | -0·463*** | -0·102 | -0·393*** | -0·109 | -15·12 |
| Lower secondary | 0·294*** | -0·0877 | 0·267** | -0·0908 | -9·18 | 0·111 | -0·0784 | 0·0866 | -0·0814 | -21·98 | 0·223*** | -0·0437 | 0·326*** | -0·0456 | 46·19 | 0·143*** | -0·0415 | 0·246*** | -0·0434 | 72·03 |
| University | -0·360*** | -0·0682 | -0·410*** | -0·0705 | 13·89 | -0·138* | -0·0598 | -0·174** | -0·0618 | 26·09 | -0·359*** | -0·0327 | -0·356*** | -0·0338 | -0·84 | -0·261*** | -0·029 | -0·252*** | -0·0302 | -3·45 |
| Postgraduate | -0·550*** | -0·116 | -0·568*** | -0·122 | 3·27 | -0·276** | -0·102 | -0·288** | -0·105 | 4·35 | -0·399*** | -0·0585 | -0·373*** | -0·0616 | -6·52 | -0·278*** | -0·0505 | -0·249*** | -0·0525 | -10·43 |
| Occupational status |  |  |  |  |  |  |  |  |  |  |  |  |  |  |  |  |  |  |  |  |
| Non-healthcare worker | Ref. |  | Ref. |  |  | Ref. |  | Ref. |  |  | Ref. |  | Ref. |  |  | Ref. |  | Ref. |  |  |
| Healthcare worker | -1·183*** | -0·122 | -1·221*** | -0·124 | 3·21 | -0·826*** | -0·112 | -0·860*** | -0·114 | 4·12 | -0·304*** | -0·0644 | -0·277*** | -0·0657 | -8·88 | -0·146* | -0·058 | -0·118* | -0·0593 | -19·18 |
| Homemaker | 0·247* | -0·118 | 0·223 | -0·121 | -9·72 | 0·0461 | -0·106 | 0·0367 | -0·109 | -20·39 | -0·082 | -0·0563 | -0·0781 | -0·0575 | -4·76 | -0·170*** | -0·049 | -0·161** | -0·0506 | -5·29 |
| Retired | -0·499*** | -0·114 | -0·476*** | -0·12 | -4·61 | 0·0241 | -0·1 | 0·0305 | -0·105 | 26·56 | 0·0118 | -0·0537 | 0·0329 | -0·0567 | 178·81 | 0·243*** | -0·0479 | 0·257*** | -0·0507 | 5·76 |
| Student (non-health field) | -0·810*** | -0·15 | -0·810*** | -0·153 | 0·00 | -0·426** | -0·136 | -0·423** | -0·139 | -0·70 | -0·619*** | -0·0753 | -0·616*** | -0·0768 | -0·48 | -0·450*** | -0·0671 | -0·444*** | -0·0681 | -1·33 |
| Student (health field) | -1·421*** | -0·197 | -1·419*** | -0·204 | -0·14 | -0·727*** | -0·178 | -0·726*** | -0·182 | -0·14 | -0·877*** | -0·107 | -0·831*** | -0·108 | -5·25 | -0·571*** | -0·095 | -0·524*** | -0·0956 | -8·23 |
| Job seeker | 0·101 | -0·159 | 0·105 | -0·163 | 3·96 | 0·102 | -0·145 | 0·104 | -0·147 | 1·96 | -0·0375 | -0·074 | -0·0198 | -0·0748 | -47·20 | -0·037 | -0·0655 | -0·0201 | -0·0657 | -45·68 |
| Unemployed | 0·216 | -0·15 | 0·209 | -0·153 | -3·24 | 0·131 | -0·139 | 0·111 | -0·142 | -15·27 | 0·00337 | -0·0681 | -0·000568 | -0·07 | -116·85 | -0·0338 | -0·06 | -0·0437 | -0·0612 | 29·29 |
| Other | 0·165 | -0·787 | 0·436 | -0·802 | 164·24 | 0·336 | -0·679 | 0·58 | -0·734 | 72·62 | -0·0353 | -0·308 | -0·115 | -0·309 | 225·78 | 0·0401 | -0·251 | -0·0516 | -0·254 | -228·68 |
| Continent of citizenship |  |  |  |  |  |  |  |  |  |  |  |  |  |  |  |  |  |  |  |  |
| Italy | Ref. |  | Ref. |  |  | Ref. |  | Ref. |  |  | Ref. |  | Ref. |  |  | Ref. |  | Ref. |  |  |
| Europe (non-Italy) | 0·219 | -0·262 | 0·107 | -0·27 | -51·14 | 0·423 | -0·237 | 0·251 | -0·249 | -40·66 | -0·424** | -0·136 | -0·405** | -0·137 | -4·48 | -0·334** | -0·123 | -0·341** | -0·126 | 2·10 |
| Africa | 2·094** | -0·651 | 2·106** | -0·655 | 0·57 | 2·663*** | -0·693 | 2·559*** | -0·697 | -3·91 | -0·485 | -0·317 | -0·381 | -0·323 | -21·44 | -0·234 | -0·283 | -0·18 | -0·285 | -23·08 |
| America | -0·164 | -0·599 | -0·119 | -0·591 | -27·44 | 0·331 | -0·565 | 0·427 | -0·552 | 29·00 | -0·239 | -0·339 | -0·326 | -0·342 | 36·40 | -0·0208 | -0·312 | -0·0847 | -0·314 | 307·21 |
| Asia | 0·956 | -0·725 | 1·081 | -0·729 | 13·08 | 0·756 | -0·665 | 0·95 | -0·677 | 25·66 | 0·545 | -0·331 | 0·528 | -0·326 | -3·12 | 0·457 | -0·308 | 0·47 | -0·302 | 2·84 |
| Oceania | -9·787*** | -0·726 | -9·139*** | -0·754 | -6·62 | -8·670*** | -0·701 | -8·064*** | -0·703 | -6·99 | -1·815*** | -0·341 | -2·041*** | -0·354 | 12·45 | -1·322*** | -0·313 | -1·565*** | -0·332 | 18·38 |
| Self-identified ethnicity |  |  |  |  |  |  |  |  |  |  |  |  |  |  |  |  |  |  |  |  |
| European | Ref. |  | Ref. |  |  | Ref. |  | Ref. |  |  | Ref. |  | Ref. |  |  | Ref. |  | Ref. |  |  |
| Multi-ethnic | 0·399 | -0·331 | 0·122 | -0·331 | -69·42 | 0·461 | -0·337 | 0·242 | -0·337 | -47·51 | -0·414** | -0·154 | -0·446** | -0·157 | 7·73 | -0·386** | -0·132 | -0·393** | -0·136 | 1·81 |
| North American / Australian | 1·177* | -0·553 | 1·042 | -0·58 | -11·47 | 1·014 | -0·552 | 0·775 | -0·54 | -23·57 | -0·466 | -0·267 | -0·404 | -0·281 | -13·30 | -0·537* | -0·246 | -0·522 | -0·267 | -2·79 |
| Arab-Middle Eastern | -0·213 | -0·428 | -0·373 | -0·432 | 75·12 | -0·00433 | -0·435 | -0·164 | -0·445 | 3687·53 | -0·504* | -0·217 | -0·571* | -0·222 | 13·29 | -0·412* | -0·19 | -0·479* | -0·193 | 16·26 |
| North African | -0·67 | -0·436 | -0·704 | -0·452 | 5·07 | -0·781 | -0·418 | -0·754 | -0·434 | -3·46 | -0·218 | -0·214 | -0·294 | -0·22 | 34·86 | -0·267 | -0·185 | -0·317 | -0·189 | 18·73 |
| Latino-American | -0·125 | -0·406 | -0·1 | -0·404 | -20·00 | -0·101 | -0·395 | -0·0736 | -0·388 | -27·13 | -0·379 | -0·207 | -0·319 | -0·207 | -15·83 | -0·368 | -0·191 | -0·307 | -0·189 | -16·58 |
| African American | -0·649 | -1·086 | -0·107 | -0·98 | -83·51 | -0·373 | -1·085 | 0·221 | -0·974 | -159·25 | -0·542 | -0·453 | -0·355 | -0·432 | -34·50 | -0·42 | -0·414 | -0·21 | -0·4 | -50·00 |
| Black African | -0·841 | -0·725 | -0·88 | -0·696 | 4·64 | -1·083 | -0·722 | -1·132 | -0·711 | 4·52 | -0·331 | -0·358 | -0·244 | -0·366 | -26·28 | -0·437 | -0·332 | -0·356 | -0·337 | -18·54 |
| Asian | -0·843 | -0·522 | -0·783 | -0·573 | -7·12 | -0·553 | -0·522 | -0·56 | -0·582 | 1·27 | -0·721* | -0·283 | -0·702* | -0·3 | -2·64 | -0·593* | -0·25 | -0·603* | -0·262 | 1·69 |
| Pacific Islands | -0·889 | -1·075 | -0·759 | -1·107 | -14·62 | -0·638 | -1·213 | -0·499 | -1·221 | -21·79 | -1·374* | -0·596 | -1·333* | -0·59 | -2·98 | -1·264* | -0·511 | -1·218* | -0·509 | -3·64 |
| Material deprivation |  |  |  |  |  |  |  |  |  |  |  |  |  |  |  |  |  |  |  |  |
| No deprivation | Ref. |  | Ref. |  |  | Ref. |  | Ref. |  |  | Ref. |  | Ref. |  |  | Ref. |  | Ref. |  |  |
| Severe deprivation | 0·184 | -0·153 | 0·203 | -0·156 | 10·33 | -0·175 | -0·13 | -0·14 | -0·133 | -20·00 | 0·0567 | -0·0711 | 0·0335 | -0·0729 | -40·92 | -0·102 | -0·061 | -0·118 | -0·0631 | 15·69 |
| Chronic conditions |  |  |  |  |  |  |  |  |  |  |  |  |  |  |  |  |  |  |  |  |
| No chronic disease | Ref. |  | Ref. |  |  | Ref. |  | Ref. |  |  | Ref. |  | Ref. |  |  | Ref. |  | Ref. |  |  |
| One chronic disease | -0·171** | -0·0654 | -0·268*** | -0·0676 | 56·73 | -0·156** | -0·0581 | -0·218*** | -0·06 | 39·74 | -0·0745* | -0·0324 | -0·0558 | -0·0335 | -25·10 | -0·0678* | -0·0291 | -0·0335 | -0·0301 | -50·59 |
| More than one chronic disease | -0·448*** | -0·0847 | -0·524*** | -0·0887 | 16·96 | -0·524*** | -0·0731 | -0·567*** | -0·0767 | 8·21 | 0·528*** | -0·0425 | 0·584*** | -0·0442 | 10·61 | 0·495*** | -0·0395 | 0·565*** | -0·0413 | 14·14 |
| Living with a person with disability |  |  |  |  |  |  |  |  |  |  |  |  |  |  |  |  |  |  |  |  |
| No | Ref. |  | Ref. |  |  | Ref. |  | Ref. |  |  | Ref. |  | Ref. |  |  | Ref. |  | Ref. |  |  |
| Yes | -0·253*** | -0·0749 | -0·187* | -0·0783 | -26·09 | -0·162* | -0·0668 | -0·133 | -0·0694 | -17·90 | 0·107** | -0·0364 | 0·0515 | -0·0373 | -51·87 | 0·147*** | -0·0334 | 0·0755* | -0·034 | -48·64 |
| Inadequate health literacy |  |  |  |  |  |  |  |  |  |  |  |  |  |  |  |  |  |  |  |  |
| No | Ref. |  | Ref. |  |  | Ref. |  | Ref. |  |  | Ref. |  | Ref. |  |  | Ref. |  | Ref. |  |  |
| Yes | -0·395*** | -0·0572 | -0·380*** | -0·0595 | -3·80 | -0·581*** | -0·0513 | -0·567*** | -0·0533 | -2·41 | 0·562*** | -0·0285 | 0·576*** | -0·0296 | 2·49 | 0·481*** | -0·0261 | 0·493*** | -0·0272 | 2·49 |
| Knowing someone who had AEFI |  |  |  |  |  |  |  |  |  |  |  |  |  |  |  |  |  |  |  |  |
| No | Ref. |  | Ref. |  |  | Ref. |  | Ref. |  |  | Ref. |  | Ref. |  |  | Ref. |  | Ref. |  |  |
| Yes | 4·109*** | -0·0691 | 4·195*** | -0·0716 | 2·09 | 2·003*** | -0·0635 | 2·079*** | -0·0655 | 3·79 | 1·474*** | -0·0315 | 1·442*** | -0·0326 | -2·17 | 0·545*** | -0·03 | 0·505*** | -0·0311 | -7·34 |
| Knowing someone who had VPD |  |  |  |  |  |  |  |  |  |  |  |  |  |  |  |  |  |  |  |  |
| No | Ref. |  | Ref. |  |  | Ref. |  | Ref. |  |  | Ref. |  | Ref. |  |  | Ref. |  | Ref. |  |  |
| Yes | -1·949*** | -0·0695 | -1·945*** | -0·0717 | -0·21 | -1·158*** | -0·0617 | -1·147*** | -0·0633 | -0·95 | -0·332*** | -0·0348 | -0·419*** | -0·0358 | 26·20 | 0·0166 | -0·032 | -0·0651* | -0·0328 | -492·17 |
| Reported barriers to vaccination |  |  |  |  |  |  |  |  |  |  |  |  |  |  |  |  |  |  |  |  |
| No | Ref. |  | Ref. |  |  | Ref. |  | Ref. |  |  | Ref. |  | Ref. |  |  | Ref. |  | Ref. |  |  |
| Yes | 0·309*** | -0·0606 | 0·330*** | -0·0627 | 6·80 | -0·113* | -0·0527 | -0·121* | -0·0547 | 7·08 | 0·474*** | -0·0299 | 0·452*** | -0·0307 | -4·64 | 0·288*** | -0·0271 | 0·253*** | -0·028 | -12·15 |
| Information source cluster |  |  |  |  |  |  |  |  |  |  |  |  |  |  |  |  |  |  |  |  |
| Diversified sources | Ref. |  | Ref. |  |  | Ref. |  | Ref. |  |  | Ref. |  | Ref. |  |  | Ref. |  | Ref. |  |  |
| Professional-only sources | -0·476*** | -0·0607 | -0·467*** | -0·0631 | -1·89 | -0·209*** | -0·0518 | -0·189*** | -0·0539 | -9·57 | -0·582*** | -0·0303 | -0·507*** | -0·0313 | -12·89 | -0·465*** | -0·0277 | -0·384*** | -0·0285 | -17·42 |
| Trust in sources | -2·409*** | -0·052 | -2·384*** | -0·0536 | -1·04 | -1·580*** | -0·048 | -1·565*** | -0·0496 | -0·95 | -0·461*** | -0·0245 | -0·472*** | -0·0254 | 2·39 | -0·0963*** | -0·0224 | -0·110*** | -0·0232 | 14·23 |
| By religious leaders |  |  |  |  |  |  |  |  |  |  |  |  |  |  |  |  |  |  |  |  |
| Yes | Ref. |  | Ref. |  |  | Ref. |  | Ref. |  |  | Ref. |  | Ref. |  |  | Ref. |  | Ref. |  |  |
| No | 0·173 | -0·0975 | 0·244* | -0·1 | 41·04 | -0·106 | -0·0905 | -0·109 | -0·0934 | 2·83 | 0·347*** | -0·0474 | 0·344*** | -0·0487 | -0·86 | 0·224*** | -0·0429 | 0·188*** | -0·0441 | -16·07 |
| Don’t know | -0·248** | -0·0787 | -0·181* | -0·0811 | -27·02 | -0·240*** | -0·067 | -0·233*** | -0·0693 | -2·92 | 0·249*** | -0·0391 | 0·223*** | -0·0405 | -10·44 | 0·253*** | -0·0349 | 0·200*** | -0·0362 | -20·95 |
| By political leaders |  |  |  |  |  |  |  |  |  |  |  |  |  |  |  |  |  |  |  |  |
| Yes | Ref. |  | Ref. |  |  | Ref. |  | Ref. |  |  | Ref. |  | Ref. |  |  | Ref. |  | Ref. |  |  |
| No | -0·372*** | -0·0981 | -0·383*** | -0·101 | 2·96 | -0·188* | -0·091 | -0·187* | -0·094 | -0·53 | -0·215*** | -0·0476 | -0·256*** | -0·0491 | 19·07 | -0·133** | -0·0431 | -0·169*** | -0·0443 | 27·07 |
| Don’t know | -1·217*** | -0·0799 | -1·182*** | -0·0823 | -2·88 | -0·515*** | -0·0679 | -0·514*** | -0·0698 | -0·19 | -0·431*** | -0·0387 | -0·481*** | -0·04 | 11·60 | -0·122*** | -0·035 | -0·185*** | -0·0356 | 51·64 |
| By teachers |  |  |  |  |  |  |  |  |  |  |  |  |  |  |  |  |  |  |  |  |
| Yes | Ref. |  | Ref. |  |  | Ref. |  | Ref. |  |  | Ref. |  | Ref. |  |  | Ref. |  | Ref. |  |  |
| No | 0·915*** | -0·106 | 0·894*** | -0·11 | -2·30 | 0·483*** | -0·0998 | 0·473*** | -0·103 | -2·07 | -0·0107 | -0·0504 | -0·0171 | -0·052 | 59·81 | -0·201*** | -0·0456 | -0·203*** | -0·0469 | 1·00 |
| Don’t know | 1·069*** | -0·0782 | 1·029*** | -0·0815 | -3·74 | 0·786*** | -0·0655 | 0·790*** | -0·0678 | 0·51 | -0·295*** | -0·0388 | -0·262*** | -0·0402 | -11·19 | -0·420*** | -0·035 | -0·368*** | -0·0355 | -12·38 |
| By health professionals |  |  |  |  |  |  |  |  |  |  |  |  |  |  |  |  |  |  |  |  |
| Yes | Ref. |  | Ref. |  |  | Ref. |  | Ref. |  |  | Ref. |  | Ref. |  |  | Ref. |  | Ref. |  |  |
| No | 1·241*** | -0·108 | 1·212*** | -0·111 | -2·34 | 0·587*** | -0·103 | 0·587*** | -0·105 | 0·00 | 0·282*** | -0·0509 | 0·316*** | -0·0522 | 12·06 | -0·00673 | -0·0461 | 0·0395 | -0·0472 | -686·92 |
| Don’t know | 1·485*** | -0·0816 | 1·478*** | -0·0847 | -0·47 | 0·667*** | -0·0708 | 0·639*** | -0·0734 | -4·20 | 0·650*** | -0·04 | 0·740*** | -0·0412 | 13·85 | 0·289*** | -0·0363 | 0·369*** | -0·0371 | 27·68 |
| Use of non-conventional medicine |  |  |  |  |  |  |  |  |  |  |  |  |  |  |  |  |  |  |  |  |
| No | Ref. |  | Ref. |  |  | Ref. |  | Ref. |  |  | Ref. |  | Ref. |  |  | Ref. |  | Ref. |  |  |
| Yes, integrated with conventional medicine | 0·249*** | -0·0697 | 0·260*** | -0·0715 | 4·42 | -0·122 | -0·0626 | -0·0873 | -0·0642 | -28·44 | -0·0317 | -0·0342 | -0·0205 | -0·0349 | -35·33 | -0·195*** | -0·0304 | -0·174*** | -0·0311 | -10·77 |
| Yes, as alternative to conventional medicine | 2·415*** | -0·111 | 2·396*** | -0·115 | -0·79 | 1·180*** | -0·0983 | 1·176*** | -0·103 | -0·34 | 0·645*** | -0·0475 | 0·672*** | -0·0497 | 4·19 | 0·101* | -0·0438 | 0·132** | -0·0455 | 30·69 |
| Political orientation |  |  |  |  |  |  |  |  |  |  |  |  |  |  |  |  |  |  |  |  |
| Right (7–9) | Ref. |  | Ref. |  |  | Ref. |  | Ref. |  |  | Ref. |  | Ref. |  |  | Ref. |  | Ref. |  |  |
| Centre (4–6) | -0·0673 | -0·0754 | -0·0973 | -0·0781 | 44·58 | 0·305*** | -0·0663 | 0·276*** | -0·0685 | -9·51 | -0·369*** | -0·0377 | -0·346*** | -0·0393 | -6·23 | -0·204*** | -0·0337 | -0·181*** | -0·0353 | -11·27 |
| Extreme left (0) | -0·692*** | -0·168 | -0·763*** | -0·174 | 10·26 | 0·205 | -0·15 | 0·15 | -0·156 | -26·83 | -0·756*** | -0·0814 | -0·789*** | -0·0848 | 4·37 | -0·361*** | -0·0718 | -0·384*** | -0·0749 | 6·37 |
| Left (1–3) | -1·143*** | -0·0939 | -1·123*** | -0·097 | -1·75 | -0·0589 | -0·083 | -0·0404 | -0·0853 | -31·41 | -0·880*** | -0·0475 | -0·889*** | -0·0494 | 1·02 | -0·402*** | -0·042 | -0·409*** | -0·0439 | 1·74 |
| Extreme right (10) | 0·870*** | -0·175 | 0·895*** | -0·187 | 2·87 | 0·0633 | -0·16 | 0·0734 | -0·168 | 15·96 | 0·525*** | -0·0826 | 0·557*** | -0·0846 | 6·10 | 0·169* | -0·0735 | 0·193* | -0·0752 | 14·20 |
| Non-aligned with traditional parties | 0·650*** | -0·0938 | 0·668*** | -0·0972 | 2·77 | 0·788*** | -0·0806 | 0·770*** | -0·0833 | -2·28 | 0·340*** | -0·0466 | 0·331*** | -0·0481 | -2·65 | 0·401*** | -0·043 | 0·376*** | -0·044 | -6·23 |
| Prefer not to answer | -0·0565 | -0·113 | -0·148 | -0·117 | 161·95 | 0·596*** | -0·0999 | 0·539*** | -0·104 | -9·56 | -0·341*** | -0·0599 | -0·315*** | -0·0631 | -7·62 | -0·053 | -0·0564 | -0·0113 | -0·0599 | -78·68 |
| Religion |  |  |  |  |  |  |  |  |  |  |  |  |  |  |  |  |  |  |  |  |
| Catholic | Ref. |  | Ref. |  |  | Ref. |  | Ref. |  |  | Ref. |  | Ref. |  |  | Ref. |  | Ref. |  |  |
| Orthodox | -0·619*** | -0·161 | -0·614*** | -0·17 | -0·81 | -0·642*** | -0·149 | -0·630*** | -0·157 | -1·87 | 0·644*** | -0·0835 | 0·635*** | -0·0866 | -1·40 | 0·634*** | -0·0802 | 0·628*** | -0·0832 | -0·95 |
| Protestant | 1·048*** | -0·315 | 0·864** | -0·321 | -17·56 | 0·956** | -0·291 | 0·820** | -0·3 | -14·23 | -0·175 | -0·144 | -0·223 | -0·146 | 27·43 | -0·216 | -0·128 | -0·242 | -0·132 | 12·04 |
| Jewish | 1·487** | -0·475 | 1·575*** | -0·477 | 5·92 | 1·779*** | -0·454 | 1·891*** | -0·448 | 6·30 | -0·628** | -0·236 | -0·620* | -0·271 | -1·27 | -0·499* | -0·207 | -0·481 | -0·251 | -3·61 |
| Muslim | 0·801** | -0·286 | 0·845** | -0·293 | 5·49 | 0·405 | -0·285 | 0·445 | -0·296 | 9·88 | 0·244 | -0·136 | 0·287* | -0·141 | 17·62 | 0·0695 | -0·119 | 0·11 | -0·122 | 58·27 |
| Jehovah’s Witness | 0·384 | -0·291 | 0·564 | -0·314 | 46·88 | 0·576* | -0·271 | 0·709* | -0·283 | 23·09 | 0·0662 | -0·131 | 0·079 | -0·133 | 19·34 | 0·151 | -0·12 | 0·143 | -0·127 | -5·30 |
| Atheist | -0·12 | -0·123 | -0·0132 | -0·126 | -89·00 | -0·0056 | -0·107 | 0·0668 | -0·11 | -1292·86 | -0·164** | -0·0572 | -0·160** | -0·059 | -2·44 | -0·114* | -0·0499 | -0·125* | -0·0516 | 9·65 |
| Agnostic | -0·18 | -0·164 | -0·104 | -0·167 | -42·22 | 0·0918 | -0·136 | 0·155 | -0·139 | 68·85 | -0·121 | -0·0763 | -0·144 | -0·0784 | 19·01 | -0·000498 | -0·0662 | -0·029 | -0·0677 | 5723·29 |
| Buddhist | 1·336*** | -0·397 | 1·435*** | -0·405 | 7·41 | 0·963** | -0·348 | 1·076** | -0·349 | 11·73 | -0·0193 | -0·178 | -0·0615 | -0·183 | 218·65 | -0·184 | -0·159 | -0·22 | -0·163 | 19·57 |
| Hindu | 2·545** | -0·786 | 2·392** | -0·785 | -6·01 | 1·435 | -0·769 | 1·23 | -0·79 | -14·29 | 0·338 | -0·354 | 0·412 | -0·358 | 21·89 | -0·152 | -0·316 | -0·102 | -0·317 | -32·89 |
| Other | 1·786*** | -0·236 | 1·746*** | -0·237 | -2·24 | 1·308*** | -0·203 | 1·297*** | -0·208 | -0·84 | 0·322** | -0·104 | 0·289** | -0·107 | -10·25 | 0·111 | -0·0853 | 0·09 | -0·087 | -18·92 |
| Prefer not to answer | 0·751*** | -0·142 | 0·823*** | -0·147 | 9·59 | 0·485*** | -0·129 | 0·552*** | -0·134 | 13·81 | -0·0673 | -0·0695 | -0·0947 | -0·0736 | 40·71 | -0·185** | -0·064 | -0·215** | -0·0677 | 16·22 |
| Importance of religion |  |  |  |  |  |  |  |  |  |  |  |  |  |  |  |  |  |  |  |  |
| Not at all (0) | Ref. |  | Ref. |  |  | Ref. |  | Ref. |  |  | Ref. |  | Ref. |  |  | Ref. |  | Ref. |  |  |
| Slightly (1–3) | 0·213 | -0·12 | 0·252* | -0·123 | 18·31 | -0·00515 | -0·103 | 0·0201 | -0·106 | -490·29 | 0·231*** | -0·057 | 0·204*** | -0.059 | -11.69 | 0.135** | -0.0498 | 0.101* | -0.0515 | -25.19 |
| Somewhat important (4–6) | -0·195 | -0·122 | -0·0843 | -0·125 | -56·77 | -0·519*** | -0·107 | -0·423*** | -0·109 | -18·50 | 0·537*** | -0·0575 | 0·469*** | -0.0597 | -12.66 | 0.394*** | -0.0511 | 0.319*** | -0.0527 | -19.04 |
| Very (7–9) | -0·225 | -0·126 | -0·143 | -0·129 | -36·44 | -0·747*** | -0·11 | -0·684*** | -0·112 | -8·43 | 0·466*** | -0·0596 | 0·425*** | -0·0619 | -8·80 | 0·235*** | -0·0531 | 0·186*** | -0·0548 | -20·85 |
| Extremely (10) | -0·105 | -0·158 | -0·159 | -0·164 | 51·43 | -0·883*** | -0·14 | -0·943*** | -0·144 | 6·80 | 0·497*** | -0·0744 | 0·452*** | -0·0777 | -9·05 | 0·154* | -0·0666 | 0·104 | -0·0688 | -32·47 |
| Prefer not to answer | 0·242 | -0·184 | 0·168 | -0·191 | -30·58 | -0·742*** | -0·165 | -0·821*** | -0·174 | 10·65 | 1·295*** | -0·0916 | 1·414*** | -0·097 | 9·19 | 0·861*** | -0·0858 | 0·977*** | -0·0916 | 13·47 |
| Perceived NHS quality | -0·456*** | -0·0208 | -0·454*** | -0·0215 | -0·44 | -0·305*** | -0·0188 | -0·301*** | -0·0196 | -1·31 | -0·0411*** | -0·0097 | -0·0410*** | -0·0101 | -0·24 | 0·0257** | -0·00873 | 0·0267** | -0·00917 | 3·89 |
| Perceived NHS access | -0·306*** | -0·0201 | -0·301*** | -0·0209 | -1·63 | -0·310*** | -0·0181 | -0·309*** | -0·0192 | -0·32 | 0·0292** | -0·00931 | 0·0262** | -0·00972 | -10·27 | 0·0274** | -0·00839 | 0·0229* | -0·00893 | -16·42 |
| Survey mode |  |  |  |  |  |  |  |  |  |  |  |  |  |  |  |  |  |  |  |  |
| CAWI | Ref. |  | Ref. |  |  | Ref. |  | Ref. |  |  | Ref. |  | Ref. |  |  | Ref. |  | Ref. |  |  |
| CATI | -0·858*** | -0·0854 | -0·873*** | -0·0883 | 1·75 | 0·362*** | -0·0747 | 0·373*** | -0·0768 | 3·04 | -0·183*** | -0·0426 | -0·187*** | -0·0437 | 2·19 | 0·355*** | -0·0381 | 0·365*** | -0·0394 | 2·82 |
| VCBS |  |  |  |  |  | 1·952*** | -0·0206 | 1·950*** |  | -0·10 |  |  |  |  |  | 0·861*** |  | 0·863*** | -0·00969 | 0·23 |

Abbreviations: adjOR adjusted Odds Ratio, AEFI Adverse Event Following Immunisation, CATI Computer-Assisted Telephone Interviewing, CAWI Computer-Assisted Web Interviewing, CI Confidence Interval, NHS National Health Service, VPD Vaccine Preventable Disease.

* p<0.05, ** p<0.01, *** p<0.001

## Table S16. Post-stratification weighted analysis: Hierarchical linear regression model for vaccine hesitancy (Block 7, adjusted for Vaccine Conspiracy Belief Scale)

| **Variable** | **adjOR (95%CI)** | **p** |
| --- | --- | --- |
| Age group |  |  |
| 18–29 | Ref. |  |
| 30–44 | 1·34 (1·22 to 1·47) | <0·0001 |
| 45–59 | 1·39 (1·26 to 1·54) | <0·0001 |
| 60–74 | 1·47 (1·29 to 1·66) | <0·0001 |
| 75+ | 1·61 (1·39 to 1·87) | <0·0001 |
| Gender |  |  |
| Male | Ref. |  |
| Female | 1·05 (1 to 1·11) | 0·058 |
| Non-binary/Other | 1·66 (1·28 to 2·14) | 0·00011 |
| Prefer not to answer | 1·02 (0·47 to 2·21) | 0·96 |
| Marital status |  |  |
| Single | Ref. |  |
| Married | 0·9 (0·83 to 0·98) | 0·012 |
| Separated/Divorced | 1·08 (0·96 to 1·22) | 0·18 |
| Cohabiting | 1·01 (0·92 to 1·1) | 0·86 |
| Widowed | 0·79 (0·67 to 0·91) | 0·0018 |
| Children |  |  |
| No children | Ref. |  |
| Only children ≤11 years | 0·94 (0·86 to 1·03) | 0·18 |
| Only children 12-18 years | 0·84 (0·75 to 0·94) | 0·0023 |
| Only children >18 years | 0·76 (0·69 to 0·83) | <0·0001 |
| Children of various ages | 0·74 (0·65 to 0·83) | <0·0001 |
| Sexual orientation |  |  |
| Heterosexual | Ref. |  |
| Homosexual | 0·82 (0·68 to 0·99) | 0·044 |
| Bisexual | 0·88 (0·75 to 1·03) | 0·12 |
| Pansexual | 1·07 (0·81 to 1·41) | 0·63 |
| Ace spectrum | 0·98 (0·75 to 1·3) | 0·91 |
| Prefer not to answer | 0·69 (0·62 to 0·78) | <0·0001 |
| Municipality size (inhabitants) |  |  |
| ≤10,000 | Ref. |  |
| 10,001–25,000 | 0·98 (0·91 to 1·05) | 0·498 |
| 25,001–50,000 | 0·97 (0·89 to 1·06) | 0·48 |
| 50,001–100,000 | 1·05 (0·93 to 1·18) | 0·42 |
| 100,001–250,000 | 1·16 (1·01 to 1·34) | 0·038 |
| >250,000 | 0·96 (0·84 to 1·09) | 0·508 |
| Geographic macro-area |  |  |
| North-West | Ref. |  |
| North-East | 1·03 (0·95 to 1·1) | 0·49 |
| Centre | 1·04 (0·97 to 1·12) | 0·249 |
| South | 0·9 (0·84 to 0·97) | 0·0042 |
| Islands | 0·84 (0·76 to 0·92) | 0·00034 |
| Degree of urbanisation |  |  |
| Pole | Ref. |  |
| Intermunicipal pole | 1 (0·84 to 1·18) | 0·97 |
| Belt | 1·12 (1·01 to 1·24) | 0·026 |
| Intermediate | 1·2 (1·07 to 1·35) | 0·0024 |
| Peripheral | 1·06 (0·92 to 1·21) | 0·41 |
| Ultra-peripheral | 0·97 (0·77 to 1·23) | 0·82 |
| Education level |  |  |
| Upper secondary | Ref. |  |
| Primary/None | 0·52 (0·43 to 0·62) | <0·0001 |
| Lower secondary | 1·01 (0·93 to 1·09) | 0·86 |
| University | 0·88 (0·82 to 0·93) | <0·0001 |
| Postgraduate | 0·73 (0·66 to 0·81) | <0·0001 |
| Occupational status |  |  |
| Non-healthcare worker | Ref. |  |
| Healthcare worker | 0·74 (0·66 to 0·83) | <0·0001 |
| Homemaker | 0·99 (0·89 to 1·09) | 0·82 |
| Retired | 0·99 (0·9 to 1·1) | 0·86 |
| Student (non-health field) | 0·73 (0·64 to 0·84) | <0·0001 |
| Student (health field) | 0·63 (0·51 to 0·78) | <0·0001 |
| Job seeker | 1·03 (0·9 to 1·17) | 0·71 |
| Unemployed | 0·98 (0·87 to 1·11) | 0·77 |
| Other | 0·79 (0·44 to 1·43) | 0·43 |
| Continent of citizenship |  |  |
| Italy | Ref. |  |
| Europe (non-Italy) | 1·01 (0·79 to 1·28) | 0·94 |
| Africa | 1·51 (0·86 to 2·64) | 0·15 |
| America | 1·19 (0·68 to 2·09) | 0·55 |
| Asia | 0·95 (0·52 to 1·75) | 0·88 |
| Oceania |  |  |
| Self-identified ethnicity |  |  |
| European | Ref. |  |
| Multi-ethnic | 1·22 (0·92 to 1·63) | 0·17 |
| North American / Australian | 1·25 (0·78 to 2·01) | 0·36 |
| Arab-Middle Eastern | 0·94 (0·62 to 1·43) | 0·78 |
| North African | 1·2 (0·8 to 1·78) | 0·38 |
| Latino-American | 0·81 (0·55 to 1·19) | 0·28 |
| African American | 1·71 (0·6 to 4·86) | 0·31 |
| Black African | 0·75 (0·42 to 1·35) | 0·34 |
| Asian | 1·13 (0·66 to 1·96) | 0·66 |
| Pacific Islands | 0·47 (0·16 to 1·38) | 0·17 |
| Material deprivation |  |  |
| No deprivation | Ref. |  |
| Severe deprivation | 0·89 (0·78 to 1) | 0·055 |
| Chronic conditions |  |  |
| No chronic disease | Ref. |  |
| One chronic disease | 0·95 (0·9 to 1·01) | 0·088 |
| More than one chronic disease | 0·82 (0·76 to 0·88) | <0·0001 |
| Living with a person with disability |  |  |
| No | Ref. |  |
| Yes | 1·03 (0·96 to 1·1) | 0·41 |
| Inadequate health literacy |  |  |
| No | Ref. |  |
| Yes | 1·08 (1·02 to 1·13) | 0·0059 |
| Knowing someone who had AEFI |  |  |
| No | Ref. |  |
| Yes | 1·98 (1·87 to 2·1) | <0·0001 |
| Knowing someone who had VPD |  |  |
| No | Ref. |  |
| Yes | 0·62 (0·58 to 0·66) | <0·0001 |
| Reported barriers to vaccination |  |  |
| No | Ref. |  |
| Yes | 1·17 (1·11 to 1·23) | <0·0001 |
| Information source cluster |  |  |
| Diversified sources | Ref. |  |
| Professional-only sources | 0·77 (0·73 to 0·82) | <0·0001 |
| Trust in sources | 0·54 (0·51 to 0·56) | <0·0001 |
| By religious leaders |  |  |
| Yes | Ref. |  |
| No | 1·18 (1·08 to 1·29) | 0·00021 |
| Don’t know | 1·01 (0·94 to 1·08) | 0·81 |
| By political leaders |  |  |
| Yes | Ref. |  |
| No | 1·1 (1·01 to 1·2) | 0·037 |
| Don’t know | 0·83 (0·77 to 0·89) | <0·0001 |
| By teachers |  |  |
| Yes | Ref. |  |
| No | 1·24 (1·13 to 1·36) | <0·0001 |
| Don’t know | 1·28 (1·19 to 1·37) | <0·0001 |
| By health professionals |  |  |
| Yes | Ref. |  |
| No | 1·36 (1·23 to 1·49) | <0·0001 |
| Don’t know | 1·51 (1·41 to 1·62) | <0·0001 |
| Use of non-conventional medicine |  |  |
| No | Ref. |  |
| Yes, integrated with conventional medicine | 1·06 (1 to 1·13) | 0·057 |
| Yes, as alternative to conventional medicine | 1·56 (1·42 to 1·72) | <0·0001 |
| Political orientation |  |  |
| Right (7–9) | Ref. |  |
| Centre (4–6) | 1·11 (1·03 to 1·19) | 0·0032 |
| Extreme left (0) | 0·88 (0·76 to 1·01) | 0·065 |
| Left (1–3) | 0·8 (0·73 to 0·87) | <0·0001 |
| Extreme right (10) | 0·82 (0·71 to 0·95) | 0·0082 |
| Non-aligned with traditional parties | 1·16 (1·07 to 1·25) | 0·00038 |
| Prefer not to answer | 1·2 (1·07 to 1·34) | 0·0012 |
| Religion |  |  |
| Catholic | Ref. |  |
| Orthodox | 0·92 (0·79 to 1·07) | 0·28 |
| Protestant | 1·32 (1·01 to 1·72) | 0·039 |
| Jewish | 2·29 (1·41 to 3·71) | 0·00076 |
| Muslim | 1·08 (0·84 to 1·39) | 0·55 |
| Jehovah’s Witness | 1·67 (1·29 to 2·16) | <0·0001 |
| Atheist | 0·99 (0·89 to 1·09) | 0·81 |
| Agnostic | 1·08 (0·95 to 1·24) | 0·25 |
| Buddhist | 1·24 (0·89 to 1·74) | 0·204 |
| Hindu | 1·45 (0·72 to 2·94) | 0·302 |
| Other | 1·34 (1·11 to 1·61) | 0·0022 |
| Prefer not to answer | 1·23 (1·09 to 1·38) | 0·00093 |
| Importance of religion |  |  |
| Not at all (0) | Ref. |  |
| Slightly (1–3) | 1·07 (0·97 to 1·18) | 0·19 |
| Somewhat important (4–6) | 1·13 (1·02 to 1·25) | 0·016 |
| Very (7–9) | 1 (0·9 to 1·1) | 0·94 |
| Extremely (10) | 0·75 (0·66 to 0·86) | <0·0001 |
| Prefer not to answer | 1·09 (0·93 to 1·27) | 0·31 |
| Perceived NHS quality | 0·93 (0·91 to 0·95) | <0·0001 |
| Perceived NHS access | 0·89 (0·87 to 0·9) | <0·0001 |
| Survey mode |  |  |
| CAWI | Ref. |  |
| CATI | 1·28 (1·18 to 1·37) | <0·0001 |
| Vaccine Conspiracy Belief Scale | 2·08 (2·04 to 2·12) | <0·0001 |

Abbreviations: adjOR adjusted Odds Ratio, AEFI Adverse Event Following Immunisation, CATI Computer-Assisted Telephone Interviewing, CAWI Computer-Assisted Web Interviewing, CI Confidence Interval, NHS National Health Service, VPD Vaccine Preventable Disease.

Note: Regression estimates should not be interpreted as causal effects.

## Table S17. Comparison of unweighted and post-stratification weighted estimates logistic regression model for vaccine hesitancy adjusted for Vaccine Conspiracy Belief Scale (Block 7)

|  | **Unweighted** | | **Weighted** | |  |
| --- | --- | --- | --- | --- | --- |
|  | **adjOR** | **SE** | **adjOR** | **SE** | **Percentage difference adjOR** |
| Age group |  |  |  |  |  |
| 18–29 | Ref. |  | Ref. |  |  |
| 30–44 | 1·362*** | -0·062 | 1·343*** | -0·0633 | -1·395 |
| 45–59 | 1·413*** | -0·0684 | 1·393*** | -0·0698 | -1·415 |
| 60–74 | 1·511*** | -0·093 | 1·467*** | -0·0933 | -2·912 |
| 75+ | 1·650*** | -0·117 | 1·609*** | -0·123 | -2·485 |
| Gender |  |  |  |  |  |
| Male | Ref. |  | Ref. |  |  |
| Female | 1·045 | -0·0263 | 1·051 | -0·0276 | 0·574 |
| Non-binary/Other | 1·676*** | -0·225 | 1·658*** | -0·217 | -1·074 |
| Prefer not to answer | 0·98 | -0·421 | 1·021 | -0·403 | 4·184 |
| Marital status |  |  |  |  |  |
| Single | Ref. |  | Ref. |  |  |
| Married | 0·903* | -0·0364 | 0·899* | -0·0378 | -0·443 |
| Separated/Divorced | 1·08 | -0·0629 | 1·084 | -0·0655 | 0·370 |
| Cohabiting | 1·017 | -0·0453 | 1·008 | -0·0456 | -0·885 |
| Widowed | 0·771*** | -0·0546 | 0·785** | -0·0607 | 1·816 |
| Children |  |  |  |  |  |
| No children | Ref. |  | Ref. |  |  |
| Only children ≤11 years | 0·927 | -0·0413 | 0·94 | -0·0432 | 1·402 |
| Only children 12-18 years | 0·831*** | -0·0453 | 0·841** | -0·0479 | 1·203 |
| Only children >18 years | 0·749*** | -0·0319 | 0·758*** | -0·0338 | 1·202 |
| Children of various ages | 0·726*** | -0·0444 | 0·736*** | -0·046 | 1·377 |
| Sexual orientation |  |  |  |  |  |
| Heterosexual | Ref. |  | Ref. |  |  |
| Homosexual | 0·829* | -0·0776 | 0·822* | -0·08 | -0·844 |
| Bisexual | 0·862 | -0·0732 | 0·877 | -0·073 | 1·740 |
| Pansexual | 1·065 | -0·16 | 1·069 | -0·15 | 0·376 |
| Ace spectrum | 1·031 | -0·133 | 0·985 | -0·139 | -4·462 |
| Prefer not to answer | 0·674*** | -0·0371 | 0·693*** | -0·0411 | 2·819 |
| Municipality size (inhabitants) |  |  |  |  |  |
| ≤10,000 | Ref. |  | Ref. |  |  |
| 10,001–25,000 | 0·994 | -0·0343 | 0·976 | -0·0344 | -1·811 |
| 25,001–50,000 | 0·973 | -0·0411 | 0·97 | -0·0418 | -0·308 |
| 50,001–100,000 | 1·054 | -0·0608 | 1·049 | -0·0626 | -0·474 |
| 100,001–250,000 | 1·163* | -0·0814 | 1·161* | -0·0831 | -0·172 |
| >250,000 | 0·978 | -0·0618 | 0·957 | -0·0629 | -2·147 |
| Geographic macro-area |  |  |  |  |  |
| North-West | Ref. |  | Ref. |  |  |
| North-East | 1 | -0·0361 | 1·026 | -0·0377 | 2·600 |
| Centre | 1·031 | -0·0368 | 1·044 | -0·0389 | 1·261 |
| South | 0·894** | -0·032 | 0·899** | -0·0335 | 0·559 |
| Islands | 0·835*** | -0·0376 | 0·839*** | -0·041 | 0·479 |
| Degree of urbanisation |  |  |  |  |  |
| Pole | Ref. |  | Ref. |  |  |
| Intermunicipal pole | 0·978 | -0·0804 | 0·997 | -0·0843 | 1·943 |
| Belt | 1·129* | -0·0572 | 1·122* | -0·0581 | -0·620 |
| Intermediate | 1·189** | -0·0686 | 1·199** | -0·0716 | 0·841 |
| Peripheral | 1·044 | -0·0695 | 1·057 | -0·0725 | 1·245 |
| Ultra-peripheral | 0·983 | -0·113 | 0·973 | -0·114 | -1·017 |
| Education level |  |  |  |  |  |
| Upper secondary | Ref. |  | Ref. |  |  |
| Primary/None | 0·564*** | -0·0459 | 0·516*** | -0·0468 | -8·511 |
| Lower secondary | 0·989 | -0·0357 | 1·007 | -0·0391 | 1·820 |
| University | 0·889*** | -0·027 | 0·876*** | -0·0274 | -1·462 |
| Postgraduate | 0·733*** | -0·0389 | 0·730*** | -0·0395 | -0·409 |
| Occupational status |  |  |  |  |  |
| Non-healthcare worker | Ref. |  | Ref. |  |  |
| Healthcare worker | 0·748*** | -0·0435 | 0·739*** | -0·0439 | -1·203 |
| Homemaker | 0·995 | -0·0487 | 0·989 | -0·0502 | -0·603 |
| Retired | 1·001 | -0·0479 | 0·991 | -0·0505 | -0·999 |
| Student (non-health field) | 0·736*** | -0·0505 | 0·730*** | -0·0514 | -0·815 |
| Student (health field) | 0·636*** | -0·065 | 0·634*** | -0·0672 | -0·314 |
| Job seeker | 1·021 | -0·0665 | 1·025 | -0·0693 | 0·392 |
| Unemployed | 0·994 | -0·059 | 0·981 | -0·062 | -1·308 |
| Other | 0·758 | -0·225 | 0·788 | -0·238 | 3·958 |
| Continent of citizenship |  |  |  |  |  |
| Italy | Ref. |  | Ref. |  |  |
| Europe (non-Italy) | 1·085 | -0·123 | 1·009 | -0·124 | -7·005 |
| Africa | 1·545 | -0·383 | 1·506 | -0·43 | -2·524 |
| America | 1·146 | -0·309 | 1·19 | -0·343 | 3·839 |
| Asia | 0·861 | -0·258 | 0·954 | -0·294 | 10·801 |
| Oceania | - | - | - | - | - |
| Self-identified ethnicity |  |  |  |  |  |
| European | Ref. |  | Ref. |  |  |
| Multi-ethnic | 1·321 | -0·19 | 1·222 | -0·18 | -7·494 |
| North American / Australian | 1·306 | -0·298 | 1·25 | -0·303 | -4·288 |
| Arab-Middle Eastern | 1·038 | -0·198 | 0·943 | -0·2 | -9·152 |
| North African | 1·204 | -0·234 | 1·195 | -0·244 | -0·748 |
| Latino-American | 0·808 | -0·14 | 0·809 | -0·16 | 0·124 |
| African American | 1·01 | -0·468 | 1·71 | -0·911 | 69·307 |
| Black African | 0·769 | -0·217 | 0·751 | -0·226 | -2·341 |
| Asian | 1·096 | -0·272 | 1·133 | -0·317 | 3·376 |
| Pacific Islands | 0·463 | -0·187 | 0·473 | -0·259 | 2·160 |
| Material deprivation |  |  |  |  |  |
| No deprivation | Ref. |  | Ref. |  |  |
| Severe deprivation | 0·886* | -0·0545 | 0·885 | -0·0561 | -0·113 |
| Chronic conditions |  |  |  |  |  |
| No chronic disease | Ref. |  | Ref. |  |  |
| One chronic disease | 0·963 | -0·0276 | 0·951 | -0·0282 | -1·246 |
| More than one chronic disease | 0·817*** | -0·0296 | 0·818*** | -0·0312 | 0·122 |
| Living with a person with disability |  |  |  |  |  |
| No | Ref. |  | Ref. |  |  |
| Yes | 1·033 | -0·0332 | 1·029 | -0·035 | -0·387 |
| Inadequate health literacy |  |  |  |  |  |
| No | Ref. |  | Ref. |  |  |
| Yes | 1·065* | -0·0267 | 1·075** | -0·0282 | 0·939 |
| Knowing someone who had AEFI |  |  |  |  |  |
| No | Ref. |  | Ref. |  |  |
| Yes | 1·940*** | -0·0548 | 1·982*** | -0·0582 | 2·165 |
| Knowing someone who had VPD |  |  |  |  |  |
| No | Ref. |  | Ref. |  |  |
| Yes | 0·624*** | -0·0198 | 0·622*** | -0·0205 | -0·321 |
| Reported barriers to vaccination |  |  |  |  |  |
| No | Ref. |  | Ref. |  |  |
| Yes | 1·164*** | -0·0303 | 1·168*** | -0·0312 | 0·344 |
| Information source cluster |  |  |  |  |  |
| Diversified sources | Ref. |  | Ref. |  |  |
| Professional-only sources | 0·762*** | -0·0201 | 0·775*** | -0·0212 | 1·706 |
| Trust in sources | 0·534*** | -0·0119 | 0·538*** | -0·0127 | 0·749 |
| By religious leaders |  |  |  |  |  |
| Yes | Ref. |  | Ref. |  |  |
| No | 1·175*** | -0·0495 | 1·180*** | -0·0526 | 0·426 |
| Don’t know | 1·011 | -0·0358 | 1·008 | -0·0362 | -0·297 |
| By political leaders |  |  |  |  |  |
| Yes | Ref. |  | Ref. |  |  |
| No | 1·095* | -0·0465 | 1·097* | -0·0489 | 0·183 |
| Don’t know | 0·837*** | -0·0305 | 0·832*** | -0·0308 | -0·597 |
| By teachers |  |  |  |  |  |
| Yes | Ref. |  | Ref. |  |  |
| No | 1·257*** | -0·0566 | 1·237*** | -0·0591 | -1·591 |
| Don’t know | 1·256*** | -0·0451 | 1·277*** | -0·046 | 1·672 |
| By health professionals |  |  |  |  |  |
| Yes | Ref. |  | Ref. |  |  |
| No | 1·324*** | -0·0608 | 1·356*** | -0·0656 | 2·417 |
| Don’t know | 1·476*** | -0·053 | 1·509*** | -0·0546 | 2·236 |
| Use of non-conventional medicine |  |  |  |  |  |
| No | Ref. |  | Ref. |  |  |
| Yes, integrated with conventional medicine | 1·049 | -0·0316 | 1·061 | -0·0331 | 1·144 |
| Yes, as alternative to conventional medicine | 1·573*** | -0·0697 | 1·562*** | -0·0752 | -0·699 |
| Political orientation |  |  |  |  |  |
| Right (7–9) | Ref. |  | Ref. |  |  |
| Centre (4–6) | 1·114** | -0·0372 | 1·108** | -0·0385 | -0·539 |
| Extreme left (0) | 0·897 | -0·061 | 0·876 | -0·063 | -2·341 |
| Left (1–3) | 0·790*** | -0·0339 | 0·795*** | -0·0355 | 0·633 |
| Extreme right (10) | 0·817** | -0·0544 | 0·819** | -0·0619 | 0·245 |
| Non-aligned with traditional parties | 1·178*** | -0·0466 | 1·157*** | -0·0476 | -1·783 |
| Prefer not to answer | 1·259*** | -0·0679 | 1·200** | -0·0675 | -4·686 |
| Religion |  |  |  |  |  |
| Catholic | Ref. |  | Ref. |  |  |
| Orthodox | 0·917 | -0·069 | 0·92 | -0·0709 | 0·327 |
| Protestant | 1·385* | -0·183 | 1·319* | -0·177 | -4·765 |
| Jewish | 2·348*** | -0·551 | 2·290*** | -0·564 | -2·470 |
| Muslim | 1·036 | -0·122 | 1·08 | -0·14 | 4·247 |
| Jehovah’s Witness | 1·566*** | -0·195 | 1·671*** | -0·22 | 6·705 |
| Atheist | 0·963 | -0·0487 | 0·988 | -0·0506 | 2·596 |
| Agnostic | 1·048 | -0·0758 | 1·084 | -0·0749 | 3·435 |
| Buddhist | 1·238 | -0·203 | 1·244 | -0·214 | 0·485 |
| Hindu | 1·441 | -0·54 | 1·45 | -0·522 | 0·625 |
| Other | 1·317** | -0·124 | 1·336** | -0·126 | 1·443 |
| Prefer not to answer | 1·198** | -0·0702 | 1·226*** | -0·0754 | 2·337 |
| Importance of religion |  |  |  |  |  |
| Not at all (0) | Ref. |  | Ref. |  |  |
| Slightly (1–3) | 1·053 | -0·0533 | 1·067 | -0·0533 | 1·330 |
| Somewhat important (4–6) | 1·103 | -0·0552 | 1·130* | -0·0569 | 2·448 |
| Very (7–9) | 0·965 | -0·0501 | 0·996 | -0·0526 | 3·212 |
| Extremely (10) | 0·780*** | -0·0497 | 0·753*** | -0·0505 | -3·462 |
| Prefer not to answer | 1·078 | -0·0788 | 1·085 | -0·0867 | 0·649 |
| Perceived NHS quality | 0·930*** | -0·00763 | 0·931*** | -0·00833 | 0·108 |
| Perceived NHS access | 0·892*** | -0·00706 | 0·888*** | -0·00773 | -0·448 |
| Survey mode |  |  |  |  |  |
| CAWI | Ref. |  | Ref. |  |  |
| CATI | 1·266*** | -0·0473 | 1·275*** | -0·0489 | 0·711 |
| VCBS | 2·084*** | -0·0183 | 2·081*** | -0·02 | -0·144 |

Abbreviations: adjOR adjusted Odds Ratio, AEFI Adverse Event Following Immunisation, CATI Computer-Assisted Telephone Interviewing, CAWI Computer-Assisted Web Interviewing, CI Confidence Interval, NHS National Health Service, VPD Vaccine Preventable Disease.

* p<0.05, ** p<0.01, *** p<0.001

## Table S18. Post-stratification weighted analysis: Sensitivity analysis: linear regression models with adult Vaccine Hesitancy Scale (aVHS) score as outcome.

| **Variable** | **BLOCK 1[F(105, 51040)=346·03, p<0·0001; adjR2=0·3586]** | | **BLOCK 2 [F(106, 51040)=726·54; p<0·0001; adjR2=0·5672]** | |
| --- | --- | --- | --- | --- |
|  | **adjCoef (95% CI)** | **p** | **adjCoef (95% CI)** | **p** |
| Age group |  |  |  |  |
| 18–29 | Ref. |  | Ref. |  |
| 30–44 | 1·52 (1·28; 1·77) | <0·0001 | 0·93 (0·71; 1·14) | <0·0001 |
| 45–59 | 1·95 (1·68; 2·21) | <0·0001 | 1·11 (0·88; 1·33) | <0·0001 |
| 60–74 | 2·35 (2·01; 2·69) | <0·0001 | 1·21 (0·93; 1·49) | <0·0001 |
| 75+ | 1·88 (1·48; 2·28) | <0·0001 | 1·71 (1·38; 2·05) | <0·0001 |
| Gender |  |  |  |  |
| Male | Ref. |  | Ref. |  |
| Female | 0·23 (0·09; 0·37) | 0·0012 | 0·27 (0·15; 0·38) | <0·0001 |
| Non-binary/Other | 1·2 (0·53; 1·87) | 0·00042 | 0·38 (-0·21; 0·98) | 0·21 |
| Prefer not to answer | -0·05 (-1·85; 1·75) | 0·96 | -0·24 (-1·85; 1·37) | 0·77 |
| Marital status |  |  |  |  |
| Single | Ref. |  | Ref. |  |
| Married | -0·34 (-0·57; -0·11) | 0·0034 | -0·31 (-0·5; -0·12) | 0·0016 |
| Separated/Divorced | 0·56 (0·21; 0·91) | 0·0018 | 0·27 (-0·02; 0·56) | 0·067 |
| Cohabiting | 0·25 (-0·01; 0·51) | 0·058 | 0·07 (-0·14; 0·27) | 0·52 |
| Widowed | -0·51 (-0·91; -0·12) | 0·011 | -0·44 (-0·77; -0·11) | 0·0082 |
| Children |  |  |  |  |
| No children | Ref. |  | Ref. |  |
| Only children ≤11 years | -0·27 (-0·52; -0·03) | 0·0302 | -0·18 (-0·39; 0·02) | 0·084 |
| Only children 12-18 years | -0·44 (-0·74; -0·13) | 0·00497 | -0·49 (-0·75; -0·24) | 0·00015 |
| Only children >18 years | -0·89 (-1·13; -0·64) | <0·0001 | -0·82 (-1·02; -0·61) | <0·0001 |
| Children of various ages | -0·72 (-1·06; -0·38) | <0·0001 | -0·64 (-0·91; -0·36) | <0·0001 |
| Sexual orientation |  |  |  |  |
| Heterosexual | Ref. |  | Ref. |  |
| Homosexual | -0·66 (-1·22; -0·1) | 0·0201 | -0·52 (-0·95; -0·08) | 0·0198 |
| Bisexual | -0·93 (-1·4; -0·46) | 0·00012 | -0·35 (-0·73; 0·04) | 0·078 |
| Pansexual | -1·13 (-1·78; -0·47) | 0·00074 | -0·14 (-0·75; 0·48) | 0·67 |
| Ace spectrum | -0·92 (-1·59; -0·24) | 0·00074 | 0·02 (-0·63; 0·67) | 0·95 |
| Prefer not to answer | -0·39 (-0·68; -0·1) | 0·0076 | -0·01 (-0·27; 0·26) | 0·96 |
| Municipality size (inhabitants) |  |  |  |  |
| ≤10,000 | Ref. |  | Ref. |  |
| 10,001–25,000 | -0·08 (-0·27; 0·11) | 0·39 | 0 (-0·15; 0·15) | 0·997 |
| 25,001–50,000 | -0·11 (-0·34; 0·11) | 0·33 | -0·07 (-0·26; 0·11) | 0·44 |
| 50,001–100,000 | 0·23 (-0·09; 0·54) | 0·16 | 0·11 (-0·15; 0·36) | 0·42 |
| 100,001–250,000 | 0·24 (-0·14; 0·61) | 0·22 | 0·31 (-0·01; 0·62) | 0·055 |
| >250,000 | -0·02 (-0·37; 0·32) | 0·91 | -0·12 (-0·41; 0·17) | 0·403 |
| Geographic macro-area |  |  |  |  |
| North-West | Ref. |  | Ref. |  |
| North-East | 0·2 (0·01; 0·4) | 0·041 | 0·12 (-0·04; 0·29) | 0·13 |
| Centre | 0·15 (-0·05; 0·35) | 0·13 | 0·21 (0·05; 0·38) | 0·0103 |
| South | -0·35 (-0·54; -0·15) | 0·00046 | -0·36 (-0·52; -0·2) | <0·0001 |
| Islands | -0·86 (-1·12; -0·6) | <0·0001 | -0·85 (-1·07; -0·64) | <0·0001 |
| Degree of urbanisation |  |  |  |  |
| Pole | Ref. |  | Ref. |  |
| Intermunicipal pole | -0·08 (-0·51; 0·36) | 0·73 | -0·01 (-0·38; 0·35) | 0·95 |
| Belt | 0·22 (-0·05; 0·49) | 0·11 | 0·25 (0·02; 0·47) | 0·032 |
| Intermediate | 0·08 (-0·23; 0·39) | 0·62 | 0·27 (0·01; 0·53) | 0·044 |
| Peripheral | 0·15 (-0·2; 0·51) | 0·402 | 0·3 (0; 0·59) | 0·047 |
| Ultra-peripheral | 0·05 (-0·57; 0·67) | 0·87 | 0·15 (-0·34; 0·64) | 0·54 |
| Education level |  |  |  |  |
| Upper secondary | Ref. |  | Ref. |  |
| Primary/None | -1·45 (-1·85; -1·05) | <0·0001 | -1·08 (-1·45; -0·72) | <0·0001 |
| Lower secondary | 0·59 (0·39; 0·8) | <0·0001 | 0·33 (0·16; 0·5) | 0·00014 |
| University | -0·77 (-0·93; -0·6) | <0·0001 | -0·43 (-0·56; -0·29) | <0·0001 |
| Postgraduate | -0·94 (-1·24; -0·64) | <0·0001 | -0·54 (-0·77; -0·31) | <0·0001 |
| Occupational status |  |  |  |  |
| Non-healthcare worker | Ref. |  | Ref. |  |
| Healthcare worker | -1·5 (-1·79; -1·21) | <0·0001 | -0·98 (-1·22; -0·73) | <0·0001 |
| Homemaker | 0·14 (-0·14; 0·42) | 0·31 | -0·12 (-0·36; 0·11) | 0·295 |
| Retired | -0·44 (-0·72; -0·16) | 0·0019 | 0·29 (0·06; 0·51) | 0·013 |
| Student (non-health field) | -1·43 (-1·79; -1·06) | <0·0001 | -0·87 (-1·17; -0·56) | <0·0001 |
| Student (health field) | -2·25 (-2·74; -1·76) | <0·0001 | -1·25 (-1·65; -0·85) | <0·0001 |
| Job seeker | 0·08 (-0·29; 0·46) | 0·66 | 0·08 (-0·22; 0·39) | 0·595 |
| Unemployed | 0·21 (-0·13; 0·55) | 0·23 | 0·07 (-0·22; 0·35) | 0·64 |
| Other | 0·32 (-1·45; 2·09) | 0·72 | 0·53 (-0·94; 2) | 0·48 |
| Continent of citizenship |  |  |  |  |
| Italy | Ref. |  | Ref. |  |
| Europe (non-Italy) | -0·3 (-0·93; 0·33) | 0·35 | -0·09 (-0·63; 0·46) | 0·75 |
| Africa | 1·73 (0·42; 3·03) | 0·0095 | 2·38 (1·07; 3·69) | 0·00038 |
| America | -0·45 (-1·82; 0·93) | 0·53 | 0·34 (-0·84; 1·53) | 0·57 |
| Asia | 1·61 (0·08; 3·14) | 0·039 | 1·42 (0·1; 2·74) | 0·035 |
| Oceania | -11·18 (-12·76; -9·6) | <0·0001 | -9·63 (-11·01; -8·25) | <0·0001 |
| Self-identified ethnicity |  |  |  |  |
| European | Ref. |  | Ref. |  |
| Multi-ethnic | -0·32 (-1·03; 0·39) | 0·37 | -0·15 (-0·82; 0·52) | 0·66 |
| North American / Australian | 0·64 (-0·54; 1·81) | 0·39 | 0·25 (-0·77; 1·27) | 0·63 |
| Arab-Middle Eastern | -0·94 (-1·93; 0·04) | 0·061 | -0·64 (-1·59; 0·3) | 0·18 |
| North African | -1 (-1·99; -0·01) | 0·047 | -1·07 (-1·93; -0·22) | 0·014 |
| Latino-American | -0·42 (-1·34; 0·5) | 0·37 | -0·38 (-1·21; 0·45) | 0·37 |
| African American | -0·46 (-2·58; 1·66) | 0·67 | 0·01 (-2·01; 2·03) | 0·99 |
| Black African | -1·12 (-2·63; 0·38) | 0·14 | -1·49 (-2·95; -0·03) | 0·046 |
| Asian | -1·48 (-2·67; -0·3) | 0·014 | -1·16 (-2·25; -0·07) | 0·036 |
| Pacific Islands | -2·09 (-4·5; 0·32) | 0·089 | -1·72 (-4·2; 0·76) | 0·78 |
| Material deprivation |  |  |  |  |
| No deprivation | Ref. |  | Ref. |  |
| Severe deprivation | 0·24 (-0·14; 0·61) | 0·22 | -0·26 (-0·55; 0·04) | 0·088 |
| Chronic conditions |  |  |  |  |
| No chronic disease | Ref. |  | Ref. |  |
| One chronic disease | -0·32 (-0·48; -0·17) | <0·0001 | -0·25 (-0·38; -0·12) | 0·00015 |
| More than one chronic disease | 0·06 (-0·15; 0·27) | 0·57 | 0 (-0·17; 0·17) | 0·99 |
| Living with a person with disability |  |  |  |  |
| No | Ref. |  | Ref. |  |
| Yes | -0·14 (-0·32; 0·04) | 0·14 | -0·06 (-0·21; 0·09) | 0·45 |
| Inadequate health literacy |  |  |  |  |
| No | Ref. |  | Ref. |  |
| Yes | 0·2 (0·06; 0·33) | 0·0051 | -0·07 (-0·19; 0·04) | 0·21 |
| Knowing someone who had AEFI |  |  |  |  |
| No | Ref. |  | Ref. |  |
| Yes | 5·64 (5·47; 5·8) | <0·0001 | 2·58 (2·44; 2·72) | <0·0001 |
| Knowing someone who had VPD |  |  |  |  |
| No | Ref. |  | Ref. |  |
| Yes | -2·36 (-2·53; -2·2) | <0·0001 | -1·21 (-1·35; -1·07) | <0·0001 |
| Reported barriers to vaccination |  |  |  |  |
| No |  |  |  |  |
| Yes | 0·78 (0·64; 0·93) | <0·0001 | 0·13 (0·01; 0·25) | 0·297 |
| Information source cluster |  |  |  |  |
| Diversified sources | Ref. |  | Ref. |  |
| Professional-only sources | -0·97 (-1·12; -0·82) | <0·0001 | -0·57 (-0·69; -0·45) | <0·0001 |
| Trust in sources | -2·86 (-2·98; -2·73) | <0·0001 | -1·68 (-1·78; -1·57) | <0·0001 |
| By religious leaders |  |  |  |  |
| Yes | Ref. |  | Ref. |  |
| No | 0·59 (0·36; 0·81) | <0·0001 | 0·08 (-0·12; 0·27) | 0·43 |
| Don’t know | 0·04 (-0·15; 0·24) | 0·67 | -0·03 (-0·19; 0·12) | 0·68 |
| By political leaders |  |  |  |  |
| Yes | Ref. |  | Ref. |  |
| No | -0·64 (-0·87; -0·41) | <0·0001 | -0·36 (-0·55; -0·16) | 0·00045 |
| Don’t know | -1·66 (-1·86; -1·46) | <0·0001 | -0·7 (-0·86; -0·54) | <0·0001 |
| By teachers |  |  |  |  |
| Yes | Ref. |  | Ref. |  |
| No | 0·88 (0·63; 1·12) | <0·0001 | 0·27 (0·06; 0·48) | 0·013 |
| Don’t know | 0·77 (0·57; 0·97) | <0·0001 | 0·42 (0·27; 0·58) | <0·0001 |
| By health professionals |  |  |  |  |
| Yes | Ref. |  | Ref. |  |
| No | 1·53 (1·28; 1·77) | <0·0001 | 0·63 (0·41; 0·84) | <0·0001 |
| Don’t know | 2·22 (2·02; 2·42) | <0·0001 | 1·01 (0·85; 1·17) | <0·0001 |
| Use of non-conventional medicine |  |  |  |  |
| No | Ref. |  | Ref. |  |
| Yes, integrated with conventional medicine | 0·24 (0·07; 0·41) | 0·0051 | -0·26 (-0·4; -0·12) | 0·00022 |
| Yes, as alternative to conventional medicine | 3·07 (2·81; 3·33) | <0·0001 | 1·31 (1·09; 1·52) | <0·0001 |
| Political orientation |  |  |  |  |
| Right (7–9) | Ref. |  | Ref. |  |
| Centre (4–6) | -0·44 (-0·63; -0·26) | <0·0001 | 0·1 (-0·05; 0·25) | 0·21 |
| Extreme left (0) | -1·55 (-1·96; -1·14) | <0·0001 | -0·23 (-0·57; 0·1) | 0·17 |
| Left (1–3) | -2·01 (-2·25; -1·78) | <0·0001 | -0·45 (-0·64; -0·26) | <0·0001 |
| Extreme right (10) | 1·45 (1·02; 1·88) | <0·0001 | 0·27 (-0·09; 0·62) | 0·14 |
| Non-aligned with traditional parties | 1 (0·77; 1·23) | <0·0001 | 1·15 (0·97; 1·33) | <0·0001 |
| Prefer not to answer | -0·46 (-0·73; -0·19) | 0·00072 | 0·53 (0·3; 0·75) | <0·0001 |
| Religion |  |  |  |  |
| Catholic | Ref. |  | Ref. |  |
| Orthodox | 0·02 (-0·35; 0·39) | 0·91 | 0 (-0·32; 0·32) | 0·99 |
| Protestant | 0·64 (-0·08; 1·36) | 0·082 | 0·58 (-0·05; 1·21) | 0·073 |
| Jewish | 0·96 (-0·17; 2·08) | 0·097 | 1·41 (0·41; 2·41) | 0·0056 |
| Muslim | 1·13 (0·51; 1·75) | <0·0001 | 0·55 (-0·02; 1·13) | 0·059 |
| Jehovah’s Witness | 0·64 (-0·03; 1·32) | 0·062 | 0·85 (0·28; 1·43) | 0·0037 |
| Atheist | -0·17 (-0·47; 0·12) | 0·25 | -0·06 (-0·3; 0·18) | 0·63 |
| Agnostic | -0·25 (-0·65; 0·16) | 0·23 | 0·13 (-0·19; 0·44) | 0·43 |
| Buddhist | 1·37 (0·43; 2·32) | 0·0045 | 0·86 (0·1; 1·61) | 0·027 |
| Hindu | 2·8 (1·23; 4·38) | 0·00047 | 1·13 (-0·34; 2·6) | 0·13 |
| Other | 2·04 (1·47; 2·6) | <0·0001 | 1·39 (0·95; 1·82) | <0·0001 |
| Prefer not to answer | 0·73 (0·4; 1·05) | <0·0001 | 0·34 (0·06; 0·61) | 0·017 |
| Importance of religion |  |  |  |  |
| Not at all (0) | Ref. |  | Ref. |  |
| Slightly (1–3) | 0·46 (0·16; 0·75) | 0·0023 | 0·12 (-0·11; 0·35) | 0·304 |
| Somewhat important (4–6) | 0·38 (0·09; 0·68) | 0·0101 | -0·1 (-0·34; 0·13) | 0·39 |
| Very (7–9) | 0·28 (-0·02; 0·58) | 0·067 | -0·5 (-0·74; -0·26) | <0·0001 |
| Extremely (10) | 0·29 (-0·09; 0·67) | 0·13 | -0·84 (-1·15; -0·53) | <0·0001 |
| Prefer not to answer | 1·58 (1·16; 2·01) | <0·0001 | 0·16 (-0·21; 0·52) | 0·399 |
| Perceived NHS quality | -0·5 (-0·54; -0·45) | <0·0001 | -0·27 (-0·32; -0·23) | <0·0001 |
| Perceived NHS access | -0·27 (-0·32; -0·23) | <0·0001 | -0·29 (-0·33; -0·24) | <0·0001 |
| Survey mode |  |  |  |  |
| CAWI | Ref. |  | Ref. |  |
| CATI | -1·06 (-1·27; -0·85) | <0·0001 | 0·74 (0·57; 0·91) | <0·0001 |
| Vaccine Conspiracy Belief Scale |  |  | 2·81 (2·77; 2·86) | <0·0001 |

Abbreviations: adjCoef adjusted coefficient, CATI Computer Assisted Telephone Interviewing, CAWI Computer Assisted Web Interviewing, CI Confidence Interval, NHS National Health Service, VPD Vaccine Preventable Disease.

Note: Regression estimates should not be interpreted as causal effects.

## Table S19. Unweighted analysis: Sensitivity analysis: linear regression models with adult Vaccine Hesitancy Scale (aVHS) score as outcome.

| **Variable** | **BLOCK 1 ( R-squared=0·3582)** | | | **BLOCK 2 (R-squared=0·5669)** | | |
| --- | --- | --- | --- | --- | --- | --- |
|  | **adjCoef (95%CI)** | **p** | **Effect size*** | **adjCoef (95%CI)** | **p** | **Effect size*** |
| Age group |  |  | 0·0055 |  |  | 0·0035 |
| 18–29 | Ref. |  |  | Ref. |  |  |
| 30–44 | 1·55 (1·31; 1·79) | <0·0001 |  | 0·97 (0·76; 1·18) | <0·001 |  |
| 45–59 | 1·98 (1·72; 2·24) | <0·0001 |  | 1·16 (0·94; 1·38) | <0·001 |  |
| 60–74 | 2·45 (2·12; 2·78) | <0·0001 |  | 1·32 (1·04; 1·6) | <0·001 |  |
| 75+ | 1·88 (1·51; 2·26) | <0·0001 |  | 1·94 (1·62; 2·26) | <0·001 |  |
| Gender |  |  | 0·0004 |  |  | 0·0004 |
| Male | Ref. |  |  | Ref. |  |  |
| Female | 0·21 (0·08; 0·34) | 0·0019 |  | 0·25 (0·14; 0·36) | <0·001 |  |
| Non-binary/Other | 1·13 (0·47; 1·78) | 0·00072 |  | 0·18 (-0·39; 0·75) | 0·54 |  |
| Prefer not to answer | 0·21 (-1·86; 2·28) | 0·84 |  | -0·17 (-1·83; 1·49) | 0·84 |  |
| Marital status |  |  | 0·0015 |  |  | 0·0009 |
| Single | Ref. |  |  | Ref. |  |  |
| Married | -0·4 (-0·62; -0·17) | 0·00051 |  | -0·31 (-0·49; -0·12) | 0·0012 |  |
| Separated/Divorced | 0·52 (0·18; 0·86) | 0·0026 |  | 0·26 (-0·01; 0·54) | 0·059 |  |
| Cohabiting | 0·2 (-0·05; 0·45) | 0·12 |  | 0·07 (-0·13; 0·27) | 0·47 |  |
| Widowed | -0·72 (-1·09; -0·36) | 0·00012 |  | -0·55 (-0·86; -0·24) | 0·00041 |  |
| Children |  |  | 0·0013 |  |  | 0·0017 |
| No children | Ref. |  |  | Ref. |  |  |
| Only children ≤11 years | -0·3 (-0·54; -0·06) | 0·014 |  | -0·26 (-0·46; -0·06) | 0·013 |  |
| Only children 12-18 years | -0·44 (-0·74; -0·14) | 0·00401 |  | -0·54 (-0·79; -0·29) | <0·001 |  |
| Only children >18 years | -0·92 (-1·16; -0·68) | <0·0001 |  | -0·85 (-1·05; -0·65) | <0·001 |  |
| Children of various ages | -0·72 (-1·06; -0·38) | <0·0001 |  | -0·68 (-0·95; -0·41) | <0·001 |  |
| Sexual orientation |  |  | 0·0009 |  |  | 0·0002 |
| Heterosexual | Ref. |  |  | Ref. |  |  |
| Homosexual | -0·79 (-1·31; -0·27) | 0·0028 |  | -0·51 (-0·94; -0·09) | 0·018 |  |
| Bisexual | -0·98 (-1·44; -0·52) | <0·0001 |  | -0·36 (-0·73; 0·02) | 0·066 |  |
| Pansexual | -1·18 (-1·84; -0·52) | 0·00043 |  | -0·22 (-0·84; 0·39) | 0·47 |  |
| Ace spectrum | -0·95 (-1·6; -0·3) | 0·0041 |  | 0·05 (-0·57; 0·67) | 0·87 |  |
| Prefer not to answer | -0·43 (-0·71; -0·15) | 0·0028 |  | -0·09 (-0·34; 0·16) | 0·46 |  |
| Municipality size (inhabitants) |  |  | 0·0002 |  |  | 0·0003 |
| ≤10,000 | Ref. |  |  | Ref. |  |  |
| 10,001–25,000 | -0·07 (-0·26; 0·11) | 0·43 |  | 0·03 (-0·12; 0·18) | 0·71 |  |
| 25,001–50,000 | -0·12 (-0·34; 0·1) | 0·28 |  | -0·09 (-0·28; 0·09) | 0·32 |  |
| 50,001–100,000 | 0·22 (-0·08; 0·53) | 0·16 |  | 0·13 (-0·12; 0·39) | 0·2997 |  |
| 100,001–250,000 | 0·2 (-0·17; 0·56) | 0·29 |  | 0·32 (0·02; 0·63) | 0·038 |  |
| >250,000 | -0·01 (-0·34; 0·32) | 0·96 |  | -0·02 (-0·3; 0·25) | 0·86 |  |
| Geographic macro-area |  |  | 0·0020 |  |  | 0·0026 |
| North-West | Ref. |  |  | Ref. |  |  |
| North-East | 0·16 (-0·03; 0·35) | 0·097 |  | 0·07 (-0·09; 0·23) | 0·38 |  |
| Centre | 0·13 (-0·06; 0·32) | 0·17 |  | 0·22 (0·07; 0·38) | 0·0049 |  |
| South | -0·42 (-0·61; -0·24) | <0·0001 |  | -0·38 (-0·53; -0·22) | <0·001 |  |
| Islands | -0·88 (-1·13; -0·64) | <0·0001 |  | -0·82 (-1·02; -0·63) | <0·001 |  |
| Degree of urbanisation |  |  | 0·0001 |  |  | 0·0001 |
| Pole | Ref. |  |  | Ref. |  |  |
| Intermunicipal pole | -0·03 (-0·46; 0·4) | 0·89 |  | 0·08 (-0·27; 0·44) | 0·64 |  |
| Belt | 0·25 (-0·01; 0·52) | 0·0598 |  | 0·27 (0·05; 0·5) | 0·015 |  |
| Intermediate | 0·07 (-0·23; 0·38) | 0·64 |  | 0·27 (0·01; 0·52) | 0·038 |  |
| Peripheral | 0·15 (-0·2; 0·49) | 0·4004 |  | 0·3 (0·02; 0·59) | 0·038 |  |
| Ultra-peripheral | 0·1 (-0·49; 0·7) | 0·74 |  | 0·18 (-0·29; 0·65) | 0·45 |  |
| Education level |  |  | 0·0039 |  |  | 0·0021 |
| Upper secondary | Ref. |  |  | Ref. |  |  |
| Primary/None | -1·29 (-1·67; -0·91) | <0·0001 |  | -1 (-1·33; -0·66) | <0·001 |  |
| Lower secondary | 0·52 (0·32; 0·71) | <0·0001 |  | 0·25 (0·09; 0·42) | 0·0025 |  |
| University | -0·72 (-0·88; -0·56) | <0·0001 |  | -0·4 (-0·53; -0·27) | <0·001 |  |
| Postgraduate | -0·95 (-1·23; -0·67) | <0·0001 |  | -0·55 (-0·78; -0·33) | <0·001 |  |
| Occupational status |  |  | 0·0043 |  |  | 0·0025 |
| Non-healthcare worker | Ref. |  |  | Ref. |  |  |
| Healthcare worker | -1·49 (-1·77; -1·2) | <0·0001 |  | -0·97 (-1·21; -0·73) | <0·001 |  |
| Homemaker | 0·16 (-0·11; 0·44) | 0·24 |  | -0·12 (-0·35; 0·1) | 0·28 |  |
| Retired | -0·49 (-0·75; -0·22) | 0·00029 |  | 0·27 (0·05; 0·48) | 0·015 |  |
| Student (non-health field) | -1·43 (-1·78; -1·08) | <0·0001 |  | -0·88 (-1·17; -0·58) | <0·001 |  |
| Student (health field) | -2·3 (-2·78; -1·82) | <0·0001 |  | -1·3 (-1·7; -0·9) | <0·001 |  |
| Job seeker | 0·06 (-0·3; 0·43) | 0·73 |  | 0·06 (-0·24; 0·37) | 0·68 |  |
| Unemployed | 0·22 (-0·11; 0·55) | 0·195 |  | 0·1 (-0·18; 0·38) | 0·499 |  |
| Other | 0·13 (-1·66; 1·92) | 0·89 |  | 0·38 (-1·02; 1·77) | 0·597 |  |
| Continent of citizenship |  |  | 0·0002 |  |  | 0·0005 |
| Italy | Ref. |  |  | Ref. |  |  |
| Europe (non-Italy) | -0·21 (-0·82; 0·41) | 0·51 |  | 0·09 (-0·42; 0·6) | 0·74 |  |
| Africa | 1·61 (0·29; 2·93) | 0·017 |  | 2·43 (1·08; 3·77) | 0·0004 |  |
| America | -0·4 (-1·77; 0·97) | 0·56 |  | 0·31 (-0·89; 1·51) | 0·61 |  |
| Asia | 1·5 (-0·05; 3·06) | 0·059 |  | 1·21 (-0·12; 2·55) | 0·075 |  |
| Oceania | -11·6 (-13·1; -10·1) | <0·0001 |  | -9·99 (-11·34; -8·64) | <0·001 |  |
| Self-identified ethnicity |  |  | 0·0003 |  |  | 0·0004 |
| European | Ref. |  |  | Ref. |  |  |
| Multi-ethnic | -0·02 (-0·72; 0·69) | 0·97 |  | 0·07 (-0·59; 0·74) | 0·83 |  |
| North American / Australian | 0·71 (-0·38; 1·8) | 0·201 |  | 0·48 (-0·55; 1·5) | 0·36 |  |
| Arab-Middle Eastern | -0·72 (-1·68; 0·25) | 0·15 |  | -0·42 (-1·33; 0·49) | 0·37 |  |
| North African | -0·89 (-1·84; 0·06) | 0·068 |  | -1·05 (-1·87; -0·22) | 0·013 |  |
| Latino-American | -0·5 (-1·42; 0·41) | 0·28 |  | -0·47 (-1·31; 0·38) | 0·28 |  |
| African American | -1·19 (-3·54; 1·16) | 0·32 |  | -0·79 (-3·05; 1·46) | 0·49 |  |
| Black African | -1·17 (-2·75; 0·41) | 0·15 |  | -1·52 (-3·02; -0·02) | 0·047 |  |
| Asian | -1·56 (-2·7; -0·43) | 0·0069 |  | -1·15 (-2·17; -0·12) | 0·029 |  |
| Pacific Islands | -2·26 (-4·6; 0·08) | 0·058 |  | -1·9 (-4·36; 0·56) | 0·13 |  |
| Material deprivation |  |  | 0·0000 |  |  | 0·0001 |
| No deprivation | Ref. |  |  | Ref. |  |  |
| Severe deprivation | 0·24 (-0·13; 0·61) | 0·201 |  | -0·28 (-0·57; 0·01) | 0·061 |  |
| Chronic conditions |  |  | 0·0003 |  |  | 0·0003 |
| No chronic disease | Ref. |  |  | Ref. |  |  |
| One chronic disease | -0·25 (-0·4; -0·09) | 0·0016 |  | -0·22 (-0·35; -0·1) | 0·00048 |  |
| More than one chronic disease | 0·08 (-0·12; 0·28) | 0·42 |  | -0·03 (-0·19; 0·13) | 0·72 |  |
| Living with a person with disability |  |  | 0·0001 |  |  | 0·0000 |
| No |  |  |  |  |  |  |
| Yes | -0·15 (-0·32; 0·02) | 0·094 |  | -0·02 (-0·16; 0·13) | 0·83 |  |
| Inadequate health literacy |  |  |  |  |  |  |
| No |  |  | 0·0001 |  |  | 0·0001 |
| Yes | 0·17 (0·04; 0·3) | 0·012 |  | -0·1 (-0·21; 0·01) | 0·075 |  |
| Knowing someone who had AEFI |  |  | 0·0986 |  |  | 0·0298 |
| No | Ref. |  |  | Ref. |  |  |
| Yes | 5·58 (5·42; 5·74) | <0·0001 |  | 2·55 (2·41; 2·68) | <0·001 |  |
| Knowing someone who had VPD |  |  | 0·0151 |  |  | 0·0056 |
| No | Ref. |  |  | Ref. |  |  |
| Yes | -2·28 (-2·44; -2·12) | <0·0001 |  | -1·14 (-1·28; -1·01) | <0·001 |  |
| Reported barriers to vaccination |  |  | 0·0024 |  |  | 0·0002 |
| No | Ref. |  |  | Ref. |  |  |
| Yes | 0·78 (0·64; 0·92) | <0·0001 |  | 0·17 (0·06; 0·29) | 0·0027 |  |
| Information source cluster |  |  | 0·0043 |  |  | 0·0026 |
| Diversified sources | Ref. |  |  | Ref. |  |  |
| Professional-only sources | -1·06 (-1·2; -0·91) | <0·0001 |  | -0·67 (-0·79; -0·56) | <0·001 |  |
| Trust in sources | -2·87 (-2·99; -2·75) | <0·0001 | 0·0499 | -1·68 (-1·78; -1·58) | <0·001 | 0·0252 |
| By religious leaders |  |  | 0·0005 |  |  | 0·0000 |
| Yes | Ref. |  |  | Ref. |  |  |
| No | 0·52 (0·3; 0·74) | <0·0001 |  | 0·12 (-0·07; 0·31) | 0·22 |  |
| Don’t know | 0 (-0·19; 0·19) | 0·99 |  | 0·01 (-0·14; 0·16) | 0·87 |  |
| By political leaders |  |  | 0·0057 |  |  | 0·0013 |
| Yes | Ref. |  |  | Ref. |  |  |
| No | -0·59 (-0·81; -0·37) | <0·0001 |  | -0·32 (-0·51; -0·13) | 0·00099 |  |
| Don’t know | -1·65 (-1·84; -1·46) | <0·0001 |  | -0·64 (-0·79; -0·49) | <0·001 |  |
| By teachers |  |  | 0·0019 |  |  | 0·0005 |
| Yes | Ref. |  |  | Ref. |  |  |
| No | 0·9 (0·67; 1·14) | <0·0001 |  | 0·28 (0·08; 0·49) | 0·0074 |  |
| Don’t know | 0·77 (0·59; 0·96) | <0·0001 |  | 0·37 (0·22; 0·51) | <0·001 |  |
| By health professionals |  |  | 0·0105 |  |  | 0·0030 |
| Yes | Ref. |  |  | Ref. |  |  |
| No | 1·52 (1·28; 1·76) | <0·0001 |  | 0·58 (0·37; 0·79) | <0·001 |  |
| Don’t know | 2·13 (1·94; 2·33) | <0·0001 |  | 0·96 (0·8; 1·11) | <0·001 |  |
| Use of non-conventional medicine |  |  | 0·0137 |  |  | 0·0048 |
| No | Ref. |  |  | Ref. |  |  |
| Yes, integrated with conventional medicine | 0·22 (0·05; 0·38) | 0·0089 |  | -0·32 (-0·45; -0·18) | <0·001 |  |
| Yes, as alternative to conventional medicine | 3·06 (2·81; 3·31) | <0·0001 |  | 1·28 (1·08; 1·49) | <0·001 |  |
| Political orientation |  |  | 0·0159 |  |  | 0·0066 |
| Right (7–9) | Ref. |  |  | Ref. |  |  |
| Centre (4–6) | -0·44 (-0·61; -0·26) | <0·0001 |  | 0·1 (-0·04; 0·24) | 0·17 |  |
| Extreme left (0) | -1·45 (-1·84; -1·05) | <0·0001 |  | -0·16 (-0·48; 0·17) | 0·35 |  |
| Left (1–3) | -2·02 (-2·25; -1·8) | <0·0001 |  | -0·46 (-0·64; -0·28) | <0·001 |  |
| Extreme right (10) | 1·39 (0·99; 1·79) | <0·0001 |  | 0·23 (-0·1; 0·57) | 0·18 |  |
| Non-aligned with traditional parties | 0·99 (0·77; 1·21) | <0·0001 |  | 1·19 (1·02; 1·36) | <0·001 |  |
| Prefer not to answer | -0·4 (-0·66; -0·14) | 0·0027 |  | 0·54 (0·33; 0·76) | <0·001 |  |
| Religion |  |  | 0·0027 |  |  | 0·0016 |
| Catholic | Ref. |  |  | Ref. |  |  |
| Orthodox | 0·03 (-0·32; 0·37) | 0·88 |  | -0·01 (-0·31; 0·29) | 0·96 |  |
| Protestant | 0·87 (0·16; 1·58) | 0·016 |  | 0·74 (0·13; 1·35) | 0·017 |  |
| Jewish | 0·86 (-0·27; 1·99) | 0·14 |  | 1·28 (0·28; 2·28) | 0·013 |  |
| Muslim | 1·05 (0·44; 1·65) | 0·00077 |  | 0·47 (-0·09; 1·03) | 0·097 |  |
| Jehovah’s Witness | 0·45 (-0·19; 1·09) | 0·17 |  | 0·73 (0·16; 1·29) | 0·012 |  |
| Atheist | -0·28 (-0·57; 0) | 0·054 |  | -0·12 (-0·35; 0·11) | 0·31 |  |
| Agnostic | -0·3 (-0·7; 0·1) | 0·14 |  | 0·09 (-0·21; 0·4) | 0·56 |  |
| Buddhist | 1·32 (0·39; 2·24) | 0·0052 |  | 0·78 (0·02; 1·54) | 0·043 |  |
| Hindu | 2·88 (1·25; 4·51) | 0·00053 |  | 1·28 (-0·2; 2·77) | 0·0903 |  |
| Other | 2·11 (1·56; 2·66) | <0·0001 |  | 1·42 (0·99; 1·84) | <0·001 |  |
| Prefer not to answer | 0·68 (0·37; 1) | <0·0001 |  | 0·3 (0·03; 0·57) | 0·027 |  |
| Importance of religion |  |  | 0·0014 |  |  | 0·0017 |
| Not at all (0) | Ref. |  |  | Ref. |  |  |
| Slightly (1–3) | 0·44 (0·16; 0·73) | 0·0022 |  | 0·13 (-0·09; 0·35) | 0·26 |  |
| Somewhat important (4–6) | 0·34 (0·06; 0·63) | 0·018 |  | -0·13 (-0·35; 0·1) | 0·28 |  |
| Very (7–9) | 0·24 (-0·05; 0·53) | 0·11 |  | -0·51 (-0·75; -0·28) | <0·001 |  |
| Extremely (10) | 0·39 (0·03; 0·76) | 0·034 |  | -0·73 (-1·03; -0·43) | <0·001 |  |
| Prefer not to answer | 1·54 (1·13; 1·95) | <0·0001 |  | 0·12 (-0·23; 0·46) | 0·497 |  |
| Perceived NHS quality | -0·5 (-0·54; -0·45) | <0·0001 | 0·0099 | -0·28 (-0·32; -0·24) | <0·001 | 0·0046 |
| Perceived NHS access | -0·28 (-0·32; -0·23) | <0·0001 | 0·0033 | -0·28 (-0·32; -0·24) | <0·001 | 0·0051 |
| Survey mode |  |  | 0·0022 |  |  | 0·0015 |
| CAWI | Ref. |  |  | Ref. |  |  |
| CATI | -1·04 (-1·24; -0·84) | <0·0001 |  | 0·72 (0·55; 0·88) | <0·001 |  |
| Vaccine Conspiracy Belief Scale |  |  |  | 2·81 (2·77; 2·86) | <0·001 | 0·3252 |

*partial eta-squared

Abbreviations: adjCoef adjusted coefficient, AEFI Adverse Event Following, Immunization, CATI Computer Assisted Telephone Interviewing, CAWI Computer Assisted Web Interviewing, CI Confidence Interval, NHS National Health Service, VPD Vaccine Preventable Disease.

Note: Regression estimates should not be interpreted as causal effects.

All models were estimated using robust variance–covariance estimators to account for potential heteroskedasticity. Effect sizes (partial η²) were derived from the corresponding non-robust models.

## Table S20. Comparison of unweighted and post-stratification weighted estimates linear regression model for adult Vaccine Hesitancy Scale (aVHS) score.

|  | **Block 1** | | | | | **Block 2** | | | | |
| --- | --- | --- | --- | --- | --- | --- | --- | --- | --- | --- |
|  | **Unweighted** | | **Weighted** | |  | **Unweighted** | | **Weighted** | |  |
|  | **adjCoef** | **SE** | **adjCoef** | **SE** | **Percentage difference adjCoef** | **adjCoef** | **SE** | **adjCoef** | **SE** | **Percentage difference adjCoef** |
| Age group |  |  |  |  |  |  |  |  |  |  |
| 18–29 | Ref. |  | Ref. |  |  | Ref. |  | Ref. |  |  |
| 30–44 | 1·552*** | -0·123 | 1·523*** | -0·125 | -1·869 | 0·969*** | -0·106 | 0·926*** | -0·108 | -4·438 |
| 45–59 | 1·978*** | -0·132 | 1·945*** | -0·134 | -1·668 | 1·160*** | -0·113 | 1·107*** | -0·115 | -4·569 |
| 60–74 | 2·452*** | -0·17 | 2·353*** | -0·173 | -4·038 | 1·320*** | -0·141 | 1·211*** | -0·144 | -8·258 |
| 75+ | 1·885*** | -0·192 | 1·878*** | -0·206 | -0·371 | 1·940*** | -0·161 | 1·712*** | -0·171 | -11·753 |
| Gender |  |  |  |  |  |  |  |  |  |  |
| Male | Ref. |  | Ref. |  |  | Ref. |  | Ref. |  |  |
| Female | 0·210** | -0·0676 | 0·229** | -0·0708 | 9·048 | 0·251*** | -0·0557 | 0·268*** | -0·0582 | 6·773 |
| Non-binary/Other | 1·128*** | -0·333 | 1·200*** | -0·34 | 6·383 | 0·178 | -0·292 | 0·38 | -0·304 | 113·483 |
| Prefer not to answer | 0·21 | -1·058 | -0·0472 | -0·918 | -122·476 | -0·17 | -0·849 | -0·242 | -0·821 | 42·353 |
| Marital status |  |  |  |  |  |  |  |  |  |  |
| Single | Ref. |  | Ref. |  |  | Ref. |  | Ref. |  |  |
| Married | -0·397*** | -0·114 | -0·344** | -0·117 | -13·350 | -0·307** | -0·0947 | -0·306** | -0·0967 | -0·326 |
| Separated/Divorced | 0·521** | -0·173 | 0·559** | -0·179 | 7·294 | 0·264 | -0·14 | 0·269 | -0·147 | 1·894 |
| Cohabiting | 0·197 | -0·126 | 0·251 | -0·132 | 27·411 | 0·0739 | -0·102 | 0·0675 | -0·106 | -8·660 |
| Widowed | -0·725*** | -0·188 | -0·514* | -0·201 | -29·103 | -0·550*** | -0·156 | -0·441** | -0·167 | -19·818 |
| Children |  |  |  |  |  |  |  |  |  |  |
| No children | Ref. |  | Ref. |  |  | Ref. |  | Ref. |  |  |
| Only children ≤11 years | -0·300* | -0·123 | -0·272* | -0·126 | -9·333 | -0·258* | -0·103 | -0·182 | -0·105 | -29·457 |
| Only children 12-18 years | -0·438** | -0·152 | -0·437** | -0·155 | -0·228 | -0·544*** | -0·128 | -0·494*** | -0·13 | -9·191 |
| Only children >18 years | -0·923*** | -0·123 | -0·885*** | -0·127 | -4·117 | -0·850*** | -0·0996 | -0·815*** | -0·104 | -4·118 |
| Children of various ages | -0·722*** | -0·172 | -0·719*** | -0·175 | -0·416 | -0·681*** | -0·139 | -0·636*** | -0·14 | -6·608 |
| Sexual orientation |  |  |  |  |  |  |  |  |  |  |
| Heterosexual | Ref. |  | Ref. |  |  | Ref. |  | Ref. |  |  |
| Homosexual | -0·791** | -0·264 | -0·664* | -0·286 | -16·056 | -0·513* | -0·217 | -0·517* | -0·222 | 0·780 |
| Bisexual | -0·980*** | -0·234 | -0·929*** | -0·241 | -5·204 | -0·356 | -0·193 | -0·345 | -0·196 | -3·090 |
| Pansexual | -1·182*** | -0·336 | -1·125*** | -0·333 | -4·822 | -0·224 | -0·313 | -0·135 | -0·313 | -39·732 |
| Ace spectrum | -0·950** | -0·331 | -0·916** | -0·343 | -3·579 | 0·052 | -0·315 | 0·02 | -0·33 | -61·538 |
| Prefer not to answer | -0·432** | -0·144 | -0·389** | -0·149 | -9·954 | -0·0933 | -0·127 | -0·00643 | -0·134 | -93·108 |
| Municipality size (inhabitants) |  |  |  |  |  |  |  |  |  |  |
| ≤10,000 | Ref. |  | Ref. |  |  | Ref. |  | Ref. |  |  |
| 10,001–25,000 | -0·0741 | -0·0939 | -0·0819 | -0·0961 | 10·526 | 0·0286 | -0·0766 | -0·000324 | -0·0785 | -101·133 |
| 25,001–50,000 | -0·121 | -0·113 | -0·112 | -0·115 | -7·438 | -0·0932 | -0·0935 | -0·0739 | -0·095 | -20·708 |
| 50,001–100,000 | 0·221 | -0·156 | 0·226 | -0·159 | 2·262 | 0·134 | -0·13 | 0·106 | -0·132 | -20·896 |
| 100,001–250,000 | 0·196 | -0·187 | 0·236 | -0·191 | 20·408 | 0·323* | -0·156 | 0·307 | -0·16 | -4·954 |
| >250,000 | -0·00937 | -0·17 | -0·0206 | -0·176 | 119·851 | -0·025 | -0·142 | -0·123 | -0·147 | 392·000 |
| Geographic macro-area |  |  |  |  |  |  |  |  |  |  |
| North-West | Ref. |  | Ref. |  |  | Ref. |  | Ref. |  |  |
| North-East | 0·162 | -0·0976 | 0·205* | -0·1 | 26·543 | 0·0705 | -0·08 | 0·125 | -0·0819 | 77·305 |
| Centre | 0·132 | -0·0968 | 0·154 | -0·102 | 16·667 | 0·223** | -0·0791 | 0·213* | -0·083 | -4·484 |
| South | -0·424*** | -0·0961 | -0·346*** | -0·0988 | -18·396 | -0·376*** | -0·0795 | -0·358*** | -0·082 | -4·787 |
| Islands | -0·883*** | -0·124 | -0·859*** | -0·133 | -2·718 | -0·822*** | -0·1 | -0·853*** | -0·11 | 3·771 |
| Degree of urbanisation |  |  |  |  |  |  |  |  |  |  |
| Pole | Ref. |  | Ref. |  |  | Ref. |  | Ref. |  |  |
| Intermunicipal pole | -0·0311 | -0·218 | -0·0758 | -0·22 | 143·730 | 0·0847 | -0·183 | -0·0124 | -0·185 | -114·640 |
| Belt | 0·253 | -0·135 | 0·217 | -0·137 | -14·229 | 0·275* | -0·113 | 0·246* | -0·115 | -10·545 |
| Intermediate | 0·0715 | -0·155 | 0·0801 | -0·159 | 12·028 | 0·269* | -0·13 | 0·268* | -0·133 | -0·372 |
| Peripheral | 0·148 | -0·176 | 0·152 | -0·181 | 2·703 | 0·301* | -0·145 | 0·298* | -0·15 | -0·997 |
| Ultra-peripheral | 0·102 | -0·303 | 0·05 | -0·317 | -50·980 | 0·18 | -0·238 | 0·152 | -0·249 | -15·556 |
| Education level |  |  |  |  |  |  |  |  |  |  |
| Upper secondary | Ref. |  | Ref. |  |  | Ref. |  | Ref. |  |  |
| Primary/None | -1·294*** | -0·194 | -1·450*** | -0·206 | 12·056 | -0·998*** | -0·17 | -1·084*** | -0·186 | 8·617 |
| Lower secondary | 0·517*** | -0·0994 | 0·594*** | -0·103 | 14·894 | 0·253** | -0·0837 | 0·333*** | -0·0875 | 31·621 |
| University | -0·719*** | -0·0819 | -0·766*** | -0·0853 | 6·537 | -0·400*** | -0·0663 | -0·426*** | -0·0696 | 6·500 |
| Postgraduate | -0·948*** | -0·142 | -0·940*** | -0·151 | -0·844 | -0·554*** | -0·115 | -0·536*** | -0·118 | -3·249 |
| Occupational status |  |  |  |  |  |  |  |  |  |  |
| Non-healthcare worker | Ref. |  | Ref. |  |  | Ref. |  | Ref. |  |  |
| Healthcare worker | -1·486*** | -0·146 | -1·498*** | -0·148 | 0·808 | -0·972*** | -0·124 | -0·978*** | -0·126 | 0·617 |
| Homemaker | 0·165 | -0·139 | 0·145 | -0·143 | -12·121 | -0·124 | -0·114 | -0·124 | -0·118 | 0·000 |
| Retired | -0·487*** | -0·135 | -0·443** | -0·143 | -9·035 | 0·267* | -0·11 | 0·288* | -0·116 | 7·865 |
| Student (non-health field) | -1·429*** | -0·181 | -1·426*** | -0·186 | -0·210 | -0·876*** | -0·152 | -0·867*** | -0·156 | -1·027 |
| Student (health field) | -2·299*** | -0·244 | -2·250*** | -0·251 | -2·131 | -1·298*** | -0·204 | -1·251*** | -0·205 | -3·621 |
| Job seeker | 0·0631 | -0·185 | 0·0848 | -0·19 | 34·390 | 0·0649 | -0·155 | 0·0837 | -0·157 | 28·968 |
| Unemployed | 0·219 | -0·169 | 0·208 | -0·173 | -5·023 | 0·0974 | -0·144 | 0·0677 | -0·146 | -30·493 |
| Other | 0·13 | -0·915 | 0·321 | -0·903 | 146·923 | 0·376 | -0·711 | 0·529 | -0·751 | 40·691 |
| Continent of citizenship |  |  |  |  |  |  |  |  |  |  |
| Italy | Ref. |  | Ref. |  |  | Ref. |  | Ref. |  |  |
| Europe (non-Italy) | -0·206 | -0·312 | -0·298 | -0·321 | 44·660 | 0·0885 | -0·262 | -0·0896 | -0·278 | -201·243 |
| Africa | 1·609* | -0·675 | 1·725** | -0·665 | 7·209 | 2·429*** | -0·686 | 2·378*** | -0·669 | -2·100 |
| America | -0·403 | -0·699 | -0·445 | -0·701 | 10·422 | 0·31 | -0·612 | 0·342 | -0·605 | 10·323 |
| Asia | 1·501 | -0·794 | 1·609* | -0·781 | 7·195 | 1·212 | -0·682 | 1·420* | -0·674 | 17·162 |
| Oceania | -11·60*** | -0·764 | -11·18*** | -0·806 | -3·621 | -9·992*** | -0·689 | -9·629*** | -0·703 | -3·633 |
| Self-identified ethnicity |  |  |  |  |  |  |  |  |  |  |
| European | Ref. |  | Ref. |  |  | Ref. |  | Ref. |  |  |
| Multi-ethnic | -0·0151 | -0·36 | -0·324 | -0·362 | 2045·695 | 0·0744 | -0·339 | -0·151 | -0·342 | -302·957 |
| North American / Australian | 0·711 | -0·556 | 0·638 | -0·6 | -10·267 | 0·477 | -0·523 | 0·253 | -0·521 | -46·960 |
| Arab-Middle Eastern | -0·717 | -0·493 | -0·944 | -0·504 | 31·660 | -0·417 | -0·465 | -0·644 | -0·481 | 54·436 |
| North African | -0·888 | -0·486 | -0·998* | -0·504 | 12·387 | -1·048* | -0·421 | -1·071* | -0·436 | 2·195 |
| Latino-American | -0·504 | -0·469 | -0·419 | -0·468 | -16·865 | -0·468 | -0·431 | -0·381 | -0·422 | -18·590 |
| African American | -1·191 | -1·2 | -0·462 | -1·082 | -61·209 | -0·793 | -1·151 | 0·0109 | -1·031 | -101·375 |
| Black African | -1·172 | -0·807 | -1·124 | -0·769 | -4·096 | -1·520* | -0·766 | -1·488* | -0·745 | -2·105 |
| Asian | -1·563** | -0·578 | -1·485* | -0·603 | -4·990 | -1·145* | -0·525 | -1·163* | -0·556 | 1·572 |
| Pacific Islands | -2·263 | -1·194 | -2·092 | -1·228 | -7·556 | -1·902 | -1·255 | -1·717 | -1·266 | -9·727 |
| Material deprivation |  |  |  |  |  |  |  |  |  |  |
| No deprivation | Ref. |  | Ref. |  |  | Ref. |  | Ref. |  |  |
| Severe deprivation | 0·241 | -0·188 | 0·237 | -0·192 | -1·660 | -0·277 | -0·148 | -0·258 | -0·151 | -6·859 |
| Chronic conditions |  |  |  |  |  |  |  |  |  |  |
| No chronic disease | Ref. |  | Ref. |  |  | Ref. |  | Ref. |  |  |
| One chronic disease | -0·245** | -0·0778 | -0·324*** | -0·0805 | 32·245 | -0·223*** | -0·064 | -0·251*** | -0·0662 | 12·556 |
| More than one chronic disease | 0·08 | -0·1 | 0·0599 | -0·106 | -25·125 | -0·0295 | -0·081 | -0·00154 | -0·0864 | -94·780 |
| Living with a person with disability |  |  |  |  |  |  |  |  |  |  |
| No | Ref. |  | Ref. |  |  | Ref. |  | Ref. |  |  |
| Yes | -0·146 | -0·087 | -0·136 | -0·0916 | -6·849 | -0·0158 | -0·0724 | -0·0574 | -0·0755 | 263·291 |
| Inadequate health literacy |  |  |  |  |  |  |  |  |  |  |
| No | Ref. |  | Ref. |  |  | Ref. |  | Ref. |  |  |
| Yes | 0·168* | -0·0672 | 0·196** | -0·0699 | 16·667 | -0·1 | -0·0561 | -0·0732 | -0·0584 | -26·800 |
| Knowing someone who had AEFI |  |  |  |  |  |  |  |  |  |  |
| No | Ref. |  | Ref. |  |  | Ref. |  | Ref. |  |  |
| Yes | 5·583*** | -0·081 | 5·637*** | -0·0844 | 0·967 | 2·548*** | -0·0688 | 2·584*** | -0·0715 | 1·413 |
| Knowing someone who had VPD |  |  |  |  |  |  |  |  |  |  |
| No | Ref. |  | Ref. |  |  | Ref. |  | Ref. |  |  |
| Yes | -2·281*** | -0·0824 | -2·364*** | -0·0854 | 3·639 | -1·141*** | -0·0682 | -1·212*** | -0·0704 | 6·223 |
| Reported barriers to vaccination |  |  |  |  |  |  |  |  |  |  |
| No | Ref. |  | Ref. |  |  | Ref. |  | Ref. |  |  |
| Yes | 0·783*** | -0·0722 | 0·782*** | -0·0747 | -0·128 | 0·175** | -0·0582 | 0·132* | -0·0607 | -24·571 |
| Information source cluster |  |  |  |  |  |  |  |  |  |  |
| Diversified sources | Ref. |  | Ref. |  |  | Ref. |  | Ref. |  |  |
| Professional-only sources | -1·058*** | -0·0733 | -0·974*** | -0·0763 | -7·940 | -0·673*** | -0·0585 | -0·573*** | -0·061 | -14·859 |
| Trust in sources | -2·870*** | -0·0598 | -2·856*** | -0·0619 | -0·488 | -1·677*** | -0·0505 | -1·675*** | -0·0526 | -0·119 |
| By religious leaders |  |  |  |  |  |  |  |  |  |  |
| Yes | Ref. |  | Ref. |  |  | Ref. |  | Ref. |  |  |
| No | 0·520*** | -0·112 | 0·588*** | -0·115 | 13·077 | 0·119 | -0·0964 | 0·0792 | -0·0997 | -33·445 |
| Don’t know | 0·000905 | -0·0964 | 0·0427 | -0·1 | 4618·232 | 0·0127 | -0·0764 | -0·0326 | -0·0799 | -356·693 |
| By political leaders |  |  |  |  |  |  |  |  |  |  |
| Yes | Ref. |  | Ref. |  |  | Ref. |  | Ref. |  |  |
| No | -0·587*** | -0·113 | -0·639*** | -0·117 | 8·859 | -0·321*** | -0·0973 | -0·355*** | -0·101 | 10·592 |
| Don’t know | -1·648*** | -0·0973 | -1·663*** | -0·101 | 0·910 | -0·637*** | -0·0773 | -0·699*** | -0·08 | 9·733 |
| By teachers |  |  |  |  |  |  |  |  |  |  |
| Yes | Ref. |  | Ref. |  |  | Ref. |  | Ref. |  |  |
| No | 0·904*** | -0·121 | 0·877*** | -0·125 | -2·987 | 0·282** | -0·105 | 0·269* | -0·109 | -4·610 |
| Don’t know | 0·775*** | -0·0964 | 0·767*** | -0·102 | -1·032 | 0·366*** | -0·0757 | 0·421*** | -0·0788 | 15·027 |
| By health professionals |  |  |  |  |  |  |  |  |  |  |
| Yes | Ref. |  | Ref. |  |  | Ref. |  | Ref. |  |  |
| No | 1·522*** | -0·122 | 1·528*** | -0·125 | 0·394 | 0·580*** | -0·107 | 0·627*** | -0·11 | 8·103 |
| Don’t know | 2·135*** | -0·0982 | 2·218*** | -0·102 | 3·888 | 0·956*** | -0·0793 | 1·008*** | -0·0825 | 5·439 |
| Use of non-conventional medicine |  |  |  |  |  |  |  |  |  |  |
| No | Ref. |  | Ref. |  |  | Ref. |  | Ref. |  |  |
| Yes, integrated with conventional medicine | 0·217** | -0·083 | 0·239** | -0·0854 | 10·138 | -0·317*** | -0·0686 | -0·261*** | -0·0708 | -17·666 |
| Yes, as alternative to conventional medicine | 3·060*** | -0·127 | 3·068*** | -0·133 | 0·261 | 1·282*** | -0·105 | 1·308*** | -0·11 | 2·028 |
| Political orientation |  |  |  |  |  |  |  |  |  |  |
| Right (7–9) | Ref. |  | Ref. |  |  | Ref. |  | Ref. |  |  |
| Centre (4–6) | -0·436*** | -0·0904 | -0·443*** | -0·0942 | 1·606 | 0·101 | -0·0734 | 0·0955 | -0·0766 | -5·446 |
| Extreme left (0) | -1·448*** | -0·202 | -1·551*** | -0·209 | 7·113 | -0·156 | -0·166 | -0·234 | -0·172 | 50·000 |
| Left (1–3) | -2·023*** | -0·115 | -2·012*** | -0·119 | -0·544 | -0·461*** | -0·0931 | -0·450*** | -0·0963 | -2·386 |
| Extreme right (10) | 1·394*** | -0·204 | 1·452*** | -0·218 | 4·161 | 0·232 | -0·171 | 0·267 | -0·182 | 15·086 |
| Non-aligned with traditional parties | 0·990*** | -0·111 | 0·999*** | -0·116 | 0·909 | 1·190*** | -0·0888 | 1·147*** | -0·0922 | -3·613 |
| Prefer not to answer | -0·397** | -0·132 | -0·463*** | -0·137 | 16·625 | 0·543*** | -0·111 | 0·528*** | -0·115 | -2·762 |
| Religion |  |  |  |  |  |  |  |  |  |  |
| Catholic | Ref. |  | Ref. |  |  | Ref. |  | Ref. |  |  |
| Orthodox | 0·0256 | -0·176 | 0·0211 | -0·187 | -17·578 | -0·0075 | -0·154 | -0·00217 | -0·164 | -71·067 |
| Protestant | 0·873* | -0·362 | 0·642 | -0·369 | -26·460 | 0·741* | -0·309 | 0·578 | -0·323 | -21·997 |
| Jewish | 0·859 | -0·575 | 0·955 | -0·575 | 11·176 | 1·279* | -0·512 | 1·410** | -0·509 | 10·242 |
| Muslim | 1·045*** | -0·311 | 1·131*** | -0·316 | 8·230 | 0·474 | -0·286 | 0·555 | -0·294 | 17·089 |
| Jehovah’s Witness | 0·45 | -0·329 | 0·643 | -0·344 | 42·889 | 0·727* | -0·288 | 0·853** | -0·294 | 17·331 |
| Atheist | -0·285 | -0·148 | -0·173 | -0·152 | -39·298 | -0·119 | -0·118 | -0·0582 | -0·122 | -51·092 |
| Agnostic | -0·301 | -0·202 | -0·248 | -0·207 | -17·608 | 0·0913 | -0·156 | 0·126 | -0·159 | 38·007 |
| Buddhist | 1·317** | -0·472 | 1·373** | -0·483 | 4·252 | 0·780* | -0·386 | 0·856* | -0·387 | 9·744 |
| Hindu | 2·883*** | -0·832 | 2·804*** | -0·802 | -2·740 | 1·283 | -0·757 | 1·128 | -0·75 | -12·081 |
| Other | 2·108*** | -0·281 | 2·035*** | -0·286 | -3·463 | 1·419*** | -0·217 | 1·387*** | -0·224 | -2·255 |
| Prefer not to answer | 0·684*** | -0·161 | 0·728*** | -0·166 | 6·433 | 0·300* | -0·136 | 0·337* | -0·141 | 12·333 |
| Importance of religion |  |  |  |  |  |  |  |  |  |  |
| Not at all (0) | Ref. |  | Ref. |  |  | Ref. |  | Ref. |  |  |
| Slightly (1–3) | 0·444** | -0·145 | 0·456** | -0·15 | 2·703 | 0·13 | -0·114 | 0·121 | -0·118 | -6·923 |
| Somewhat important (4–6) | 0·342* | -0·144 | 0·385* | -0·15 | 12·573 | -0·125 | -0·116 | -0·103 | -0·12 | -17·600 |
| Very (7–9) | 0·241 | -0·149 | 0·282 | -0·154 | 17·012 | -0·511*** | -0·12 | -0·498*** | -0·124 | -2·544 |
| Extremely (10) | 0·393* | -0·185 | 0·293 | -0·195 | -25·445 | -0·729*** | -0·151 | -0·839*** | -0·157 | 15·089 |
| Prefer not to answer | 1·536*** | -0·209 | 1·582*** | -0·216 | 2·995 | 0·119 | -0·176 | 0·157 | -0·186 | 31·933 |
| Perceived NHS quality | -0·497*** | -0·024 | -0·495*** | -0·025 | -0·402 | -0·279*** | -0·02 | -0·275*** | -0·0213 | -1·434 |
| Perceived NHS access | -0·276*** | -0·0231 | -0·275*** | -0·0243 | -0·362 | -0·282*** | -0·0193 | -0·286*** | -0·0211 | 1·418 |
| Survey mode |  |  |  |  |  |  |  |  |  |  |
| CAWI | Ref. |  | Ref. |  |  | Ref. |  | Ref. |  |  |
| CATI | -1·041*** | -0·104 | -1·059*** | -0·107 | 1·729 | 0·718*** | -0·0835 | 0·738*** | -0·0862 | 2·786 |
| Vaccine Conspiracy Belief Scale |  |  |  |  |  | 2.813*** | -0.0219 | 2.813*** | -0.0231 | 0 |

Abbreviations: adjCoef adjusted coefficient, CATI Computer Assisted Telephone Interviewing, CAWI Computer Assisted Web Interviewing, CI Confidence Interval, NHS National Health Service, VPD Vaccine Preventable Disease.

* p<0.05, ** p<0.01, *** p<0.001
