# Supplementary material for: Prevalence of vaccine hesitancy in Italy: a cross-sectional study
Source: Lancet Reg Health Eur. 2026 Jan 31;63:101603. doi: 10.1016/j.lanepe.2026.101603 (PMC12882703; doi:10.1016/j.lanepe.2026.101603)
Supplement: Translated Abstract [file mmc2.docx]

**This translation in Italian was submitted by the authors and we reproduce it as supplied. It has not been peer reviewed. Our editorial processes have only been applied to the original abstract in English, which should serve as reference for this manuscript.**

**Prevalence of vaccine hesitancy in Italy: a cross-sectional study**

**Authors**

Giuseppina Lo Moro, MD*^a^, Prof Fabrizio Bert, MD*°^a^, Giovanna Elisa Calabrò, PhD^b^, Prof Mauro Giovanni Carta, MD^c^, Giulia Cossu, PhD^c^, Prof Corrado De Vito, PhD^d^, Manuela Martella, MD^a^, Azzurra Massimi, PhD^d^, Prof Anna Odone, PhD^e,f^, Paolo Ragusa, MD^a^, Giacomo Pietro Vigezzi, PhD^e^, Prof Walter Ricciardi, MPH^g^, Prof Roberta Siliquini, MD^a,h^

* Joint first authorship.

**Affiliations**

^a^ Department of Public Health and Pediatric Sciences, University of Turin, Turin, Italy

^b^ Department of Human Sciences, Society and Health, University of Cassino and Southern Lazio, Cassino, Italy

^c^ Department of Medical Sciences and Public Health, University of Cagliari, Cagliari, Italy

^d^ Department of Public Health and Infectious Diseases, Sapienza University of Rome, Rome, Italy

^e^ Department of Public Health, Experimental and Forensic Medicine. University of Pavia, Pavia, Italy

^f^ Medical Direction, Fondazione IRCCS Policlinico San Matteo, Pavia, Italy

^g^ Section of Hygiene, Department of Life Sciences and Public Health, Università Cattolica del Sacro Cuore, Rome, Italy

^h^ AOU City of Health and Sciences, Turin, Italy.

°**Correspondence to: Prof Fabrizio Bert**

Department of Public Health and Pediatric Sciences, University of Turin, Turin, Italy

Via Santena 5 bis, 10126, Turin, Italy

Email: [fabrizio.bert@unito.it](mailto:fabrizio.bert@unito.it)

Tel. +390116705816; Fax. +390116705889

**Summary**

**English version**

**Background:** Vaccine hesitancy (VH) remains a global threat, exacerbated by socio-political uncertainty. We aimed primarily to estimate VH prevalence in Italy, identifying the most susceptible subgroups, and secondarily to assess whether these patterns varied across VH dimensions.

**Methods:** Cross-sectional survey (web/telephone) among adults in Italy (September 2024-March 2025). The sample (n=52,094) was nationally representative by age, gender, education, area, municipality size. The primary outcome was VH (score≥25, adult Vaccine Hesitancy Scale, aVHS). The secondary outcomes were aVHS subscales “Lack of trust” and “Risk perception”. Post-stratification weighting for age, area, and municipality size was applied.

**Findings:** VH prevalence was 46·09% (95%CI: 45·65-46·53%). Multivariable models showed several associations with VH, e.g., gender, sexual orientation, ethnicity, health literacy, political and religious orientation, personal experiences, and vaccination support from community figures. Among many subgroups significant after multiple-comparison correction, the strongest differences in VH predicted probability (PP) were estimated among individuals using complementary/alternative medicine (PP=58·5%), right-aligned (PP=47·0%) or politically unaffiliated participants (PP=48·4%), individuals with middle school education (PP=48·3%), people aged 60–74 (PP=49·0%), and participants uncertain about healthcare workers' pro-vaccination support (PP=52·8%). While some groups, e.g., individuals with chronic conditions, inadequate health literacy, or religious participants reported higher perceived risk, others, e.g., non-binary respondents, showed higher lack of trust.

**Interpretation:** This study highlighted the importance of granular data to inform inclusive strategies. Key figures and politics emerged as relevant, deserving further exploration. Future research should evaluate tailored interventions for identified at-risk groups.

**Funding:** NextGenerationEU funding within the Italian Ministry of University and Research PNRR Extended Partnership initiative on Emerging Infectious Diseases.

**Italian version**

**Background:** L’esitazione vaccinale (*vaccine hesitancy*, VH) rimane una minaccia globale, aggravata dall’incertezza socio-politica. L’obiettivo primario dello studio è stato stimare la prevalenza della VH in Italia e identificare i sottogruppi maggiormente suscettibili; secondariamente, lo studio ha avuto l’obiettivo di valutare se tali pattern variassero tra le diverse dimensioni della VH.

**Metodi:** Indagine trasversale (online/telefonica) condotta tra adulti residenti in Italia (settembre 2024-marzo 2025). Il campione (n=52.094) era rappresentativo a livello nazionale per età, genere, istruzione, area geografica e popolosità del comune. L’outcome primario è stato la VH (punteggio ≥25 alla Adult Vaccine Hesitancy Scale, aVHS). Gli outcome secondari sono stati le sottoscale aVHS “Mancanza di fiducia” e “Percezione del rischio”. È stata applicata una ponderazione post-stratificazione per età, area geografica e popolosità del comune.

**Risultati:** La prevalenza di VH è risultata pari al 46,09% (IC95%: 45,65-46,53%). I modelli multivariabile hanno mostrato diverse associazioni con la VH, tra cui genere, orientamento sessuale, etnia, alfabetizzazione sanitaria, orientamento politico e religioso, esperienze personali e supporto alla vaccinazione da parte di figure chiave nella comunità. Tra i numerosi sottogruppi che hanno mantenuto un’associazione significativa dopo correzione per confronti multipli, le maggiori differenze nella probabilità predetta (PP) di VH sono state osservate tra gli utilizzatori di medicina complementare/alternativa (PP=58,5%), i partecipanti politicamente orientati a destra (PP=47,0%) o non affiliati politicamente (PP=48,4%), le persone con istruzione fino alla scuola media (PP=48,3%), gli individui di età 60-74 anni (PP=49,0%) e i partecipanti incerti sul supporto pro-vaccinazione degli operatori sanitari (PP=52,8%). Mentre alcuni gruppi (ad esempio persone con patologie croniche, con alfabetizzazione sanitaria inadeguata o partecipanti religiosi) riportavano una maggiore percezione del rischio, altri (ad esempio i rispondenti con genere non binario) mostravano livelli più elevati di mancanza di fiducia.

**Interpretazione:** Lo studio evidenzia l’importanza di disporre di dati maggiormente granulari per informare strategie realmente inclusive. Figure chiave e fattori politici emergono come elementi rilevanti e meritevoli di ulteriore approfondimento. Studi futuri dovrebbero valutare interventi mirati per i gruppi identificati come maggiormente a rischio.

**Finanziamento:** Finanziamento NextGenerationEU nell’ambito dell’iniziativa PNRR Extended Partnership del Ministero dell’Università e della Ricerca sulle Malattie Infettive Emergenti.
